# Characterization of hypoxia-responsive states in ovarian cancer to identify hot tumors and aid adjuvant therapy

**DOI:** 10.1007/s12672-024-00859-8

**Published:** 2024-01-31

**Authors:** Minghui Cao, Liwei Xiao, Shuo Chen, Jiaming Huang

**Affiliations:** 1 Department of Radiology, Sun Yat-Sen Memorial Hospital, Sun Yat-Sen University, Guangzhou, 510120 China; 2 Guangdong Provincial Key Laboratory of Malignant Tumor Epigenetics and Gene Regulation, Medical Research Center, Sun Yat-Sen Memorial Hospital, Sun Yat-Sen University, Guangzhou, 510120 China; 3 Department of Obstetrics and Gynecology, The First Affiliated Hospital of Sun Yat-sen University, Guangzhou, Guangzhou, 510080 Guangdong China

**Keywords:** Hypoxia, Ovarian cancer, Immunotherapy, Hot tumor, Hypoxic score

## Abstract

**Backgrounds:**

The hypoxia-responsive state of cancer is a complex pathophysiological process involving numerous genes playing different roles. Due to the rapid proliferation of cancer cells and chaotic angiogenesis, the clinical features of hypoxia-responsive states are not yet clear in patients with ovarian cancer.

**Methods:**

Based on the RNA expression levels of 14 hypoxic markers, our study screened out hypoxia-related genes and construct a hypoxic score pattern to quantify the hypoxia-responsive states of a single tumor. Combining clinical prognosis, tumor mutation burden, microsatellite instability, the expression level of the immune checkpoint, IC50, and other indicators to evaluate the impact of different hypoxia-responsive states on clinical prognosis and therapeutic sensitivity.

**Results:**

Our study identified a subgroup with an active hypoxia-responsive state and they have a worse clinical prognosis but exhibit higher immunogenicity and higher sensitivity to immunotherapy.

**Conclusions:**

This work revealed that hypoxia-responsive states played an important role in formation of tumor immunogenicity. Evaluating the hypoxia-responsive state will contribute to guiding more effective immunotherapy strategies.

**Supplementary Information:**

The online version contains supplementary material available at 10.1007/s12672-024-00859-8.

## Introduction

Ovarian cancer (OC) is the most lethal malignancy of the female reproductive tract, with 239,000 new cases and 152,000 deaths annually [1]. Considering the lack of effective early detection methods and delayed symptoms, more than 75% of OC patients are at an advanced stage when they were first diagnosed [2]. Surgical resection and platinum-based chemotherapy are still the first-line therapy [3], but a considerable number of OC patients are reported to experience disease recurrence and chemoresistance [4]. Although newly developed technologies such as immunotherapy provide new opportunities for the treatment of OC, they are not sufficient to overcome the disease. More important issues should be taken into consideration that how to address for these therapies to be more effective.

A recent breakthrough in cancer research is the clinical efficacy of immune checkpoint inhibitors (ICIs) in selected cancer indications [5]. With the increase of platinum-based chemotherapy drug resistance groups, the successful application of ICIs have well filled this gap. It was reported that nivolumab, an anti-PD-1 therapy, has encouraged safety and clinical efficacy in patients with platinum-resistant OC [6], and it can significantly prolong the survival time in patients with advanced tumor stage [7]. Unfortunately, the efficacy of this treatment in specific OC patients was found to be low, most likely due to the presence of multiple immune inhibitory mechanisms in the TME and a lower number of somatic mutations. Scientists have created the perception that this kind of OC patient would be a poorly immunogenic, ‘cold’ tumor. Indeed, previous data has shown that a large number of patients only experience short-term benefits or no benefits at all from immune checkpoint blockade therapies, which is not in line with our pursuit of precision medicine [8]. Identifying ‘hot’ tumors in OC patients will, therefore, help us circumvent potential challenges in the treatment of cancer and reach the goal of precision medicine. The diversity of immune evasion mechanisms remains a key obstacle in turning nonresponsive ‘cold’ tumors into responsive ‘hot’ ones. Therefore, exploring the mechanisms of such transitions and tumor immunotyping can provide significant insights into designing effective therapeutic strategies against cancer.

Hypoxia is a complex, dynamically pathophysiological process that commonly exists in tumor tissue [9], which regulates almost all signal pathways known to tumor cells through interaction [10, 11]. When the tumor tissue is in a low oxygen level, the tumor cells will show a corresponding hypoxia-responsive state, among which high expression of hypoxia-inducible factors (HIFs) and promotion of transcription is one of the typical characteristics [12, 13]. Hypoxic tumor cells obtain malignant phenotypes such as angiogenesis, immune escape, and metastasis by secreting a variety of inflammatory factors [14, 15]. Emerging evidence reveals that hypoxia plays a prominent role in tumor resistance to immunotherapy because of its effects on immune suppression [16–18]. Targeting the transcription activity of HIFs can switch the microenvironment of tumors from cold non-inflamed/not-infiltrated into hot inflamed and infiltrated by cytotoxic immune cells. A better understanding of hypoxia will help us yield detailed insights into the mechanisms by which tumor cells resist immunotherapy. HIFs are significantly highly expressed in hypoxic tumor cells [19] and have been verified in OC [20, 21]. However, simply using HIFs family proteins to reflect the complex manifestations and mechanisms of the hypoxia-responsive state in solid tumors is not accurate enough. Ye et al. found that several hypoxic markers in tumor cells can characterize the hypoxia-responsive state of tumors from multiple dimensions such as cell cycle, metabolism, angiogenesis, and inflammation [22]. This will help us better understand the molecular biological mechanisms of the hypoxia-responsive state. At present, targeting hypoxia in the tumor microenvironment is a potential strategy to improve cancer immunotherapy. Unraveling the characteristics of the hypoxia-responsive state of OC will help to evaluate the clinical prognosis and identify patients who are potentially sensitive to immunotherapy and chemotherapy.

In this study, using 14 hypoxic markers, we identified different hypoxia response states in bulk-seq of OC and screened out hypoxia-related genes (HRGs). Based on the expression level of HRGs, we characterized hypoxia-responsive patterns, which can ultimately be used to identify hypoxic subgroups of OC that are sensitive to adjuvant therapy.

## Materials and methods

### Collection and preprocessing of OC data

RNA-seq data of 7 OC cohorts and associated clinical data were collected from Gene-Expression Omnibus (GEO), The Cancer Genome Atlas (TCGA) database, and International Cancer Genome Consortium (ICGC) (Additional file 1: Table S1). Besides, RNA-seq data of 88 normal ovarian tissues were downloaded from Genotype-Tissue Expression (GTEx) database and used as a control group. The collected RNA-seq data were all transformed into transcripts per kilobase million (TPM) values. The R package “limma” and “combat” algorithms were employed to correct batch effects between the 7 cohorts and merged into one cohort. The somatic mutation data, Copy Number Variation (CNV) data, and Microsatellite Instability (MSI) data of TCGA-OV were acquired from the University of California Santa Cruz (https://xena.ucsc.edu).

### Unsupervised clustering of 14 hypoxic markers

Based on the study by Ye et. al [22], 15 hypoxia-associated molecular features (PGAM1, TPI1, SLC2A1, TUBB6, VEGFA, ACOT7, ENO1, ADM, ALDOA, CDKN3, LDHA, MIF, MRPS17, NDRG1, P4HA1) can well reflect hypoxia status. Since the ACOT7 gene was not included in the sequences of the cohort, the remaining 14 markers were used to identify different hypoxia response states using unsupervised clustering method. The analysis was performed using the ConsensuClusterPlus package and the analysis was repeated 1000 times to guarantee the stability of the classification [23].

### Gene set variation analysis (GSVA) and functional annotation

To demonstrate the difference in biological processes (BP) between the hypoxia-responsive states, we performed GSVA enrichment analysis using “GSVA” R packages. The gene sets of “c2.cp.kegg.v7.2.- symbols” were downloaded from the MSigDB database for the GSVA analysis. In addition, we used R package “clusterprofiler” to perform functional annotation for the hypoxia-related genes. False discovery rate (FDR) < 0.05 was considered to be statistically significant.

### Tumour-infiltrating Immune cells

Single sample gene set enrichment analysis (ssGSEA) was performed to investigate the relative abundance of immune cells infiltration. The enrichment scores, which represent the immune cells infiltration level in the samples, were quantified using the ssGSEA analysis. Besides, the ESTIMATE algorithm was employed to evaluate the immune and stromal contents in each patient.

### Obtained HRGs and constructed hypoxia-responsive patterns

Differentially expressed genes (DEGs) between different hypoxia response states were used to conduct prognostic analysis by the univariate Cox regression method. The genes with significant positive or negative prognoses were defined as HRGs. HRGs were further used to identify different hypoxia-responsive patterns. For the sake of more accurately assessing the hypoxia response pattern of each case, the Boruta algorithm was used to reduce the dimension of the hypoxia gene signatures. Both principal components (PC) 1 and 2 were extracted as the signature score. We then defined the hypoxic score of each patient using a method like the previous studies [24, 25]:

$${\text{Hypoxic score}} = \sqrt {\sum {\left( {PC1i + PC2i} \right)} }$$

$i$ represent the expression of hypoxia phenotype-associated genes. The analysis of DEGs was performed by the R package limma, DEGs with FDR < 0.05 and |logFC|>0 were considered to be statistically significant. Finally, according to the optimal cutoff value, a group of patients with high hypoxic scores was defined as actively hypoxia-responsive state, while a group of patients with low hypoxia scores was defined as silently hypoxia-responsive state. Those tumors presenting with significant lymphocyte infiltration are referred to as “hot” tumors. To further explore the relationship between hypoxia score and tumor immune status, we evaluated the expression of TIL, immune checkpoint markers and the status of TMB and MSI in different subgroups

### Assessment of the prognosis and the efficacy of immunotherapy between different hypoxia-responsive patterns

We use the R package “maftool” to identify gene mutations between different hypoxia-responsive states. Differences in tumor mutation burden (TMB) and MSI between different hypoxia-responsive states have been used to evaluate the efficacy and prognosis and immunotherapy respectively. Data from two independent immunotherapy cohorts, IMvigor210 [26] (advanced urothelial cancer patients under atezolizumab) and GSE78220 [27] (metastatic melanoma treated with pembrolizumab), were used as external verification to evaluate the predictive effect of hypoxia-responsive states in immunotherapy.

### Assessment of the efficacy of chemotherapy between different hypoxia-responsive patterns

To evaluate the value of the hypoxic score in the clinical treatment of ovarian carcinoma, we used CMAP (https://clue.io) to screen the target components based on differentially expressed genes between high and low hypoxia scores groups. IC50 is commonly used as a measure of antagonist drug potency in pharmacological research. Components with |CMAP score|>90 are considered potentially highly sensitive drugs, Use R package “pRRophetic” to analyze the IC50 of highly sensitive components in the OC cohort as verification of CMAP results [28].

### Statistical analysis

All statistical analyses were performed using R version 4.0.3 and GraphPad Prism Version 8.0 for macOS. The Wilcoxon test and one-way analysis of variance (ANOVA) were applied to compare two independent non-parametric samples or among multiple groups. Using the univariate and multivariate Cox analyses, independent prognostic factors closely related to prognosis were identified. The Kruskal–Wallis test was used to compare more than two groups while the Wilcoxon test was used to compare two groups. Kaplan–Meier (K–M) analysis method and log-rank test were employed for survival curves and evaluation of the patient prognosis. Generally considered *p* < 0.05 was considered statistically significant.

## Results

### The landscape of genetic variation of hypoxia genes in OC

**Fig. 1:**
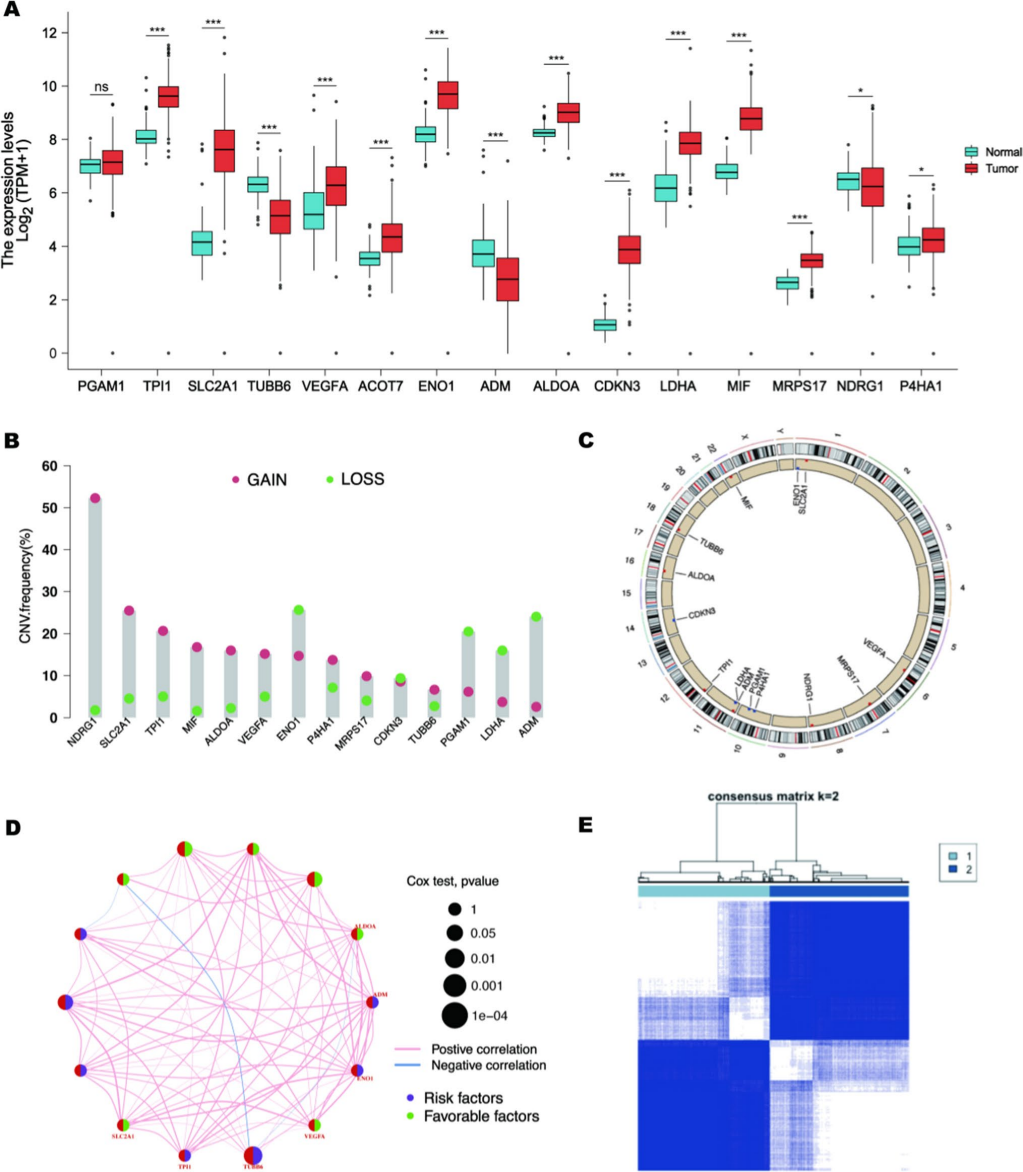
The landscape of genetic variation of hypoxia genes in OC. **A** The RNA expression of 15 hypoxic markers in normal tissues and OC tumor tissues were obtained from GTEx and TCGA databases respectively. The red color indicates tumor tissue; the blue color indicates normal tissue. Lines in the boxes represent the median value, and black dots show outliers. Asterisks represent the p-value (*p < 0.05; **p < 0.01; ***P < 0.001). **B** The CNV variation frequency of hypoxic markers in TCGA-OC cohorts. The height of the column represents the alteration frequency. Green dots represent deletion frequency; Red dots represent amplification frequency. **C** The location of CNV alteration of hypoxic markers on 23 chromosomes in OC cohort. **D** Interactions among hypoxic markers in OC. The circle size represents the *p-value* of each hypoxia-related gene on the prognosis. The range of values calculated using the Log-rank test was *p* < 0.001, *p* < 0.01, *p* < 0.05, and *p* < 0.1. The green half of the circle depicts favorable prognostic factors; the purple half of the circle stands for risk prognostic factors. The lines linking regulators indicate interactions, and their thickness shows the correlation strength between regulators. The blue color represents negative correlation whereas the red color represents positive correlation. **E** Consensus matrices of the meta-cohort for suitable *k* value (*k* = 2)

The whole study was conducted as the flow chart (Additional file 2: Figure S1). We compared the expression of 15 hypoxic markers in 427 OC tissues and 88 normal ovarian tissues. All 14 hypoxic markers except PGAM1 have significant expression differences (Fig. 1A). 7 OC bulk-seq datasets with corresponding clinical information were recruited into one meta-cohort (Additional file 1: Table S1), OC patients with high and low expression levels of these hypoxic markers showed different overall survival rates as demonstrated by the K–M curve (Additional file 2: Figure S2). The investigation of CNV alteration frequency showed a prevalent CNV alteration in 15 hypoxic markers and most were focused on the amplification in copy number, while ENO1, PGAM1, LDHA, and ADM had a widespread frequency of CNV deletion (Fig. 1B). The location of CNV alteration of hypoxic markers on chromosomes was shown in Fig. 1C. The comprehensive landscape of hypoxic markers interactions and their prognostic significance for OC patients was depicted with the hypoxic regulator network (Fig. 1D). We found that genes related to angiogenesis, inflammatory response, and metabolic regulation presented a remarkable correlation in expression. These results suggest that the independent function of hypoxic markers, as well as the regulatory network formed by each other, may play important roles in OC progression.

### Hypocluster mediated by 14 hypoxia genes

**Fig. 2:**
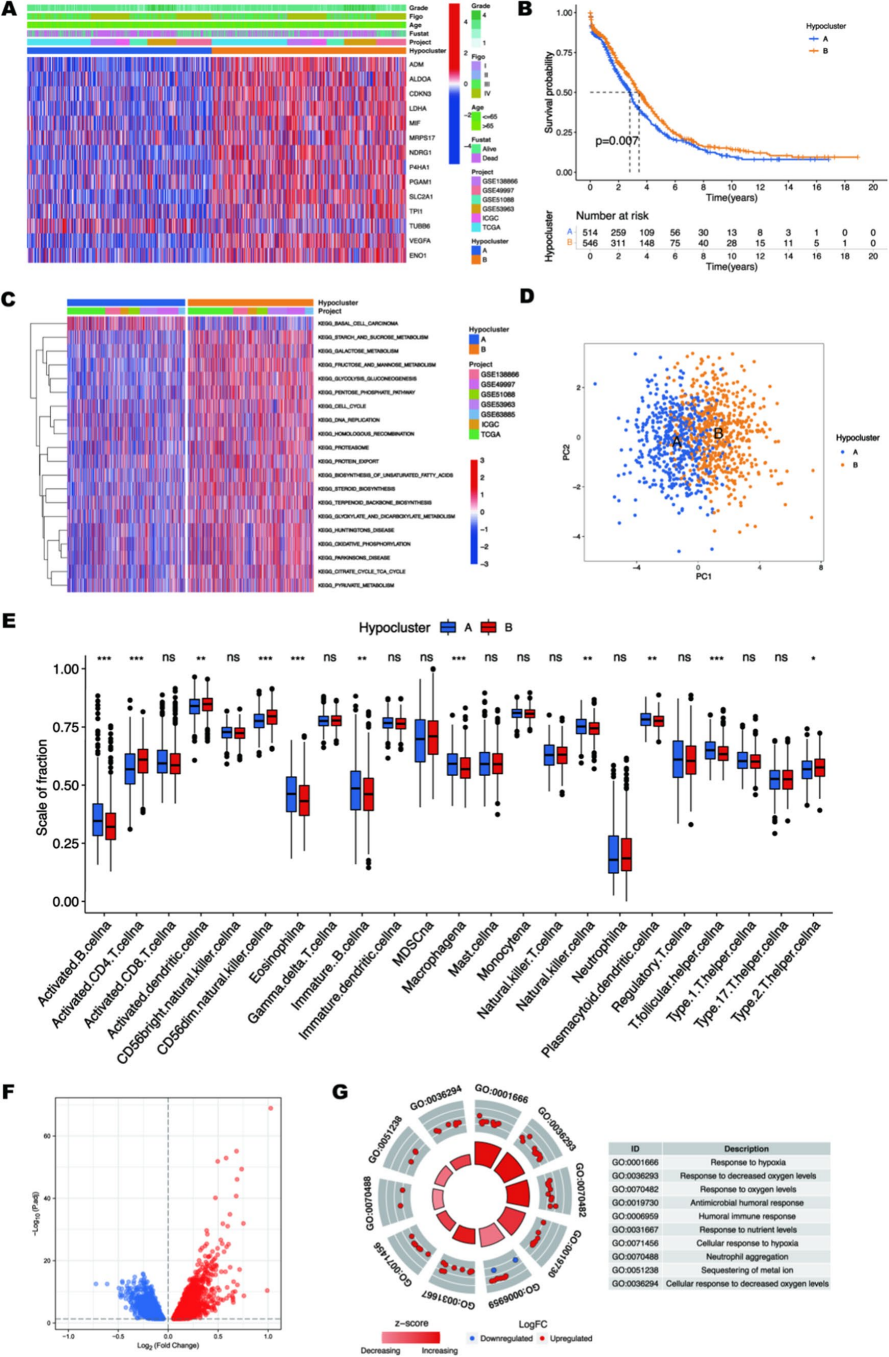
Biological characteristics and immune infiltration for various hypoxic clusters. **A** Heatmap showing the expression level of hypoxic markers and clinical information in two hypoclusters. **B** The survival curve of A and B hyposclusters. **C** Heatmap showing the biological pathways for two hyposclusters. **D** Principal component analysis for the transcriptome profiles of two hyposclusters. **E** The distribution of immune infiltrating cells in two hyposclusters. Lines in the boxes represent the median value, and black dots show outliers. The asterisks show the p-value (*p < 0.05; **p < 0.01; ***p < 0.001). **F** The volcano plot shows all significantly differential HRGs. The red dots indicate upregulated genes; the blue dot indicates downregulated genes. **G** GO enrichment analysis between two hypoclusters. Higher z-scores represent the higher significant activity of biological functions

To identify distinct hypoxic responsive states, meta-cohort was divided into two subgroups based on the expression of the 14 hypoxic markers using the unsupervised clustering method, with 514 patients in group A and 546 patients in group B. These two subgroups were defined as hypocluster A and B (Fig. 1E and Additional file 1: Table S2). We found the generally high expression of many hypoxic markers in hypocluster B, so hypocluster B was considered to be in an active hypoxia-responsive state (Fig. 2A). We originally planned to indicate that hypocluster B was associated with significantly worse survival probability using K–M analysis. Unexpectedly, patients in this hypocluster did not show a matching survival disadvantage (Fig. 2B). To explore the biological functions of the two hypoxia modification patterns, we conducted the GSVA analysis. Among the Top 20 significantly enriched signaling pathways, metabolism-related signaling pathways were significantly enriched in hypoclusterB, including glycolysis and gluconeogenesis metabolism and biosynthesis of unsaturated fatty acids (Fig. 2C and D). It was well known that the metabolism of hypoxic tumor cells would be significantly enhanced, and this result is in line with our previous consideration that hypoclusterB belongs to an active hypoxia-responsive state. Previous studies have shown that hypoxia can lead to T cell exhaustion and immunosuppression [29]. The results of ssGSEA analysis have revealed that there was no significant difference in tumor microenvironment (TME) between the two hypoclusters except for a few differences in innate immune cells (Fig. 2E). The above results show that these 14 hypoxic markers seem to only reflect the hypoxia response state of OC, but they do not seem to be of good value for the evaluation of clinical prognosis and TME infiltration. To further clarify our hypothesis, we identified 4879 DEGs, with 2536 downregulated and 2343 upregulated genes, between two hypoclusters (Fig. 2F and Additional file 1: Table S3). DEGs upregulated in hypocluster B were used for GO enrichment analysis, and the results showed that the genes were mainly enriched in hypoxia-related biological functions such as response to hypoxia, response to decreased oxygen levels, and humoral immune response (Fig. 2G). Thus, the classification of hypoxia clusters could precisely reflect the hypoxia status of the analyzed OC patients.

### Identified hypoxia modification patterns

**Fig. 3:**
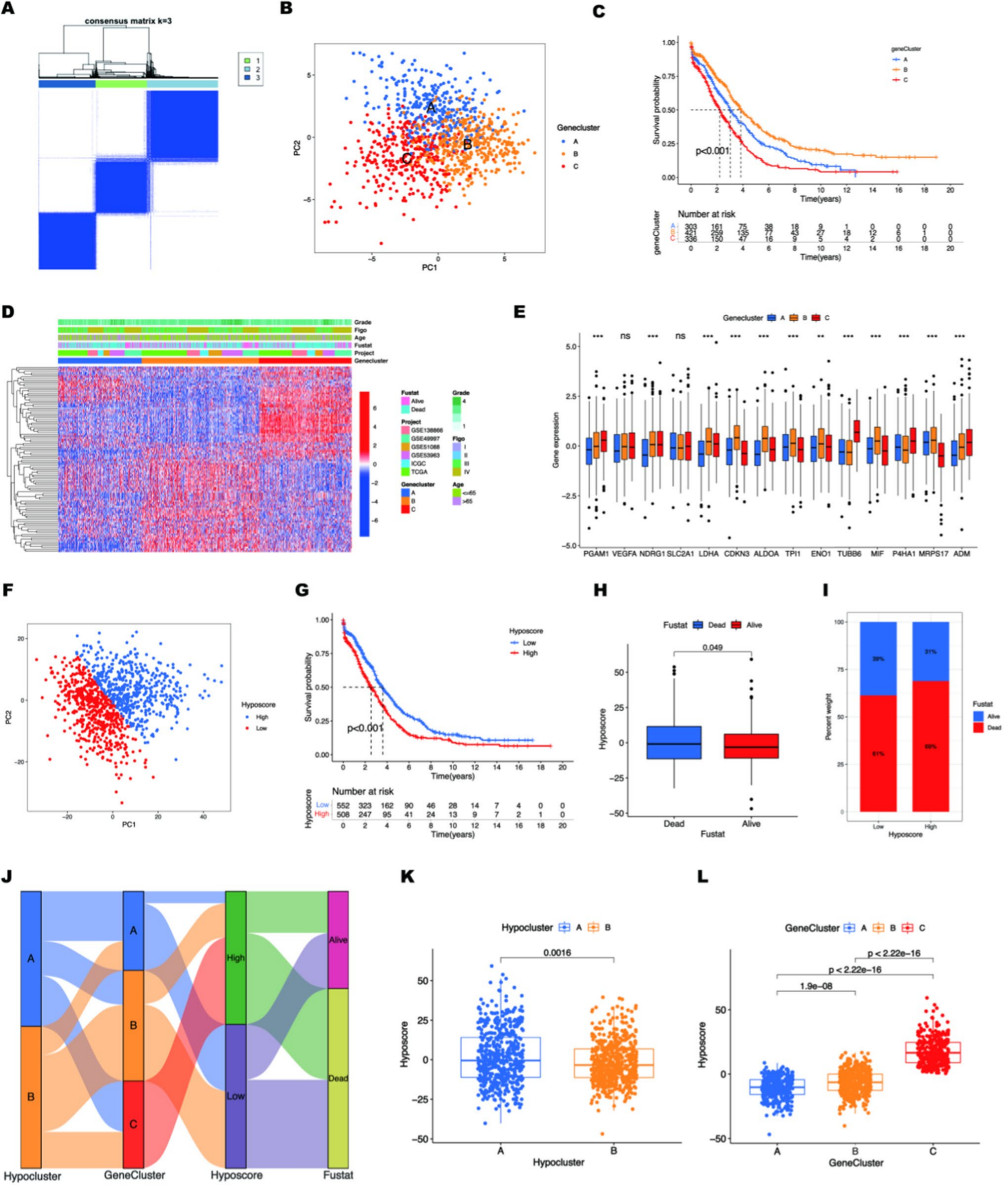
Establishment of the hypoxic score patterns. **A** Consensus matrices of the OC cancer cohort for suitable *k* value (*k* = 3). **B** Principal component analysis for the transcriptome profiles of three geneclusters. **C** The survival curves of three hypoxia gene clusters. **D** Heatmap showing the expression level of top 100 significantly differential HRGs and clinical information in three genenclusters. **E** Expression landscape of hypoxic markers in different geneclusters. **F** PCA for the transcriptome profiles of two hypoxic score clusters. **G** Survival curve of patients in the high and low hypoxic score subgroups. **H** Analysis of hypoxic scores in different survival statuses. **I** The proportion of death and alive in different hypoxic score clusters. **J** Alluvial diagram showing changes in hypoclusters, geneclusters, hypoxic score, and survival status. **K** Comparison of hypoxic scores in different hypoclusters. **L** Analysis of hypoxic scores in different geneclusters

Based on our findings, GO enrichment analysis results showed that the biological function of response to hypoxia was significantly activated in hypocluster B, which Indicated the DEGs would undoubtedly outperform the 14 hypoxic markers in identifying the hypoxia-responsive state of OC. So, we defined these DEGs as hypoxic genes. To more accurately evaluate clinical prognosis and TME infiltration by use of hypoxic genes, we filtered 4879 DEGs by univariate independent prognostic analysis and screened out 1203 HRGs associated with the prognosis of OC patients (Additional file 1: Table S4). To unravel the underlying biological characteristics of the distinct hypoxia subtypes, we performed unsupervised clustering analysis again at meta-cohort but based on the expression levels of 1203 HRGs. This time, the meta-cohort was divided into 3 more precise subgroups, and we named it genecluster (Fig. 3A and Additional file 1: Table S5). Compared with 14 hypoxic markers, unsupervised clustering analysis and principle-component analysis (PCA) of geneclusters both delineated more precise subgroups (Fig. 3B). Prognostic analysis for the three main geneclusters revealed a particularly prominent survival advantage in genecluster B (Fig. 3C). A better tumor differentiation and lower proportion of FIGO-IV stage were observed in genecluster B, and a better survival outcome **(**Fig. 3D). We found that most hypoxic markers showed significant expression differences in the gene cluster, indicating that the gene cluster can not only evaluate the clinical characteristics and prognosis of OC patients but also reflect the hypoxia response state well (Fig. 3E).

### Establishment of the hypoxic score pattern

However, genecluster can only identify hypoxic subtypes with poor clinical prognosis, and cannot accurately assess hypoxic-responsive state in individual patients. To acquire quantitative indicators of hypoxic-responsive state in the OC patients, we applied the principal component analysis to calculate the PCA score of a single case as their hypoxic score (hypoxia scores) (Fig. 3F). Based on the obtained hypoxic score, we performed survival analysis on meta-cohort and categorized the patients into high and low hypoxic score subgroups according to whether the hypoxic score was higher than the optimal cut-off of − 1.107829 (Additional file 1: Table S6). Survival analysis showed that patients with higher hypoxic scores had worse clinical outcomes, and were positively associated with clinical outcomes of death(Fig. 3G and I). We considered patients with high hypoxic scores to represent a more active hypoxia-responsive state than those with low hypoxia scores. The alluvial diagram was used to visualize the attribute changes of individual patients (Fig. 3J). Kruskal–Wallis test revealed a difference in hypoxic score between Hypoclusters. Hypocluster A showed the higher median score while Hypocluster B had the lower median score, which indicated that a higher hypoxic score could be closely linked to worse survival but activated hypoxia-responsive state combined with the above analysis results of hypocluster (Fig. 3K). The difference in the median hypoxic score between the two hypoclusters was not significant, which is consistent with our belief that the 14 hypoxic markers have imprecise titers for predicting clinical outcomes (Fig. 2B). No surprise, Kruskal-Wallis test revealed a significant difference between three geneclusters(Fig. 3L), especially genecluster C has 10 times higher hypoxia scores than genecluster B. On the contrary, survival analysis showed that genecluster B had a 50% survival probability that was nearly 2 times better than genecluster C (Fig. 2C). In general, quantitative hypoxia scores were accurate and reliable for assessing hypoxia-responsive states and clinical prognosis in individual cases.

### Hypoxic score pattern identified hot tumors in OC

**Fig. 4:**
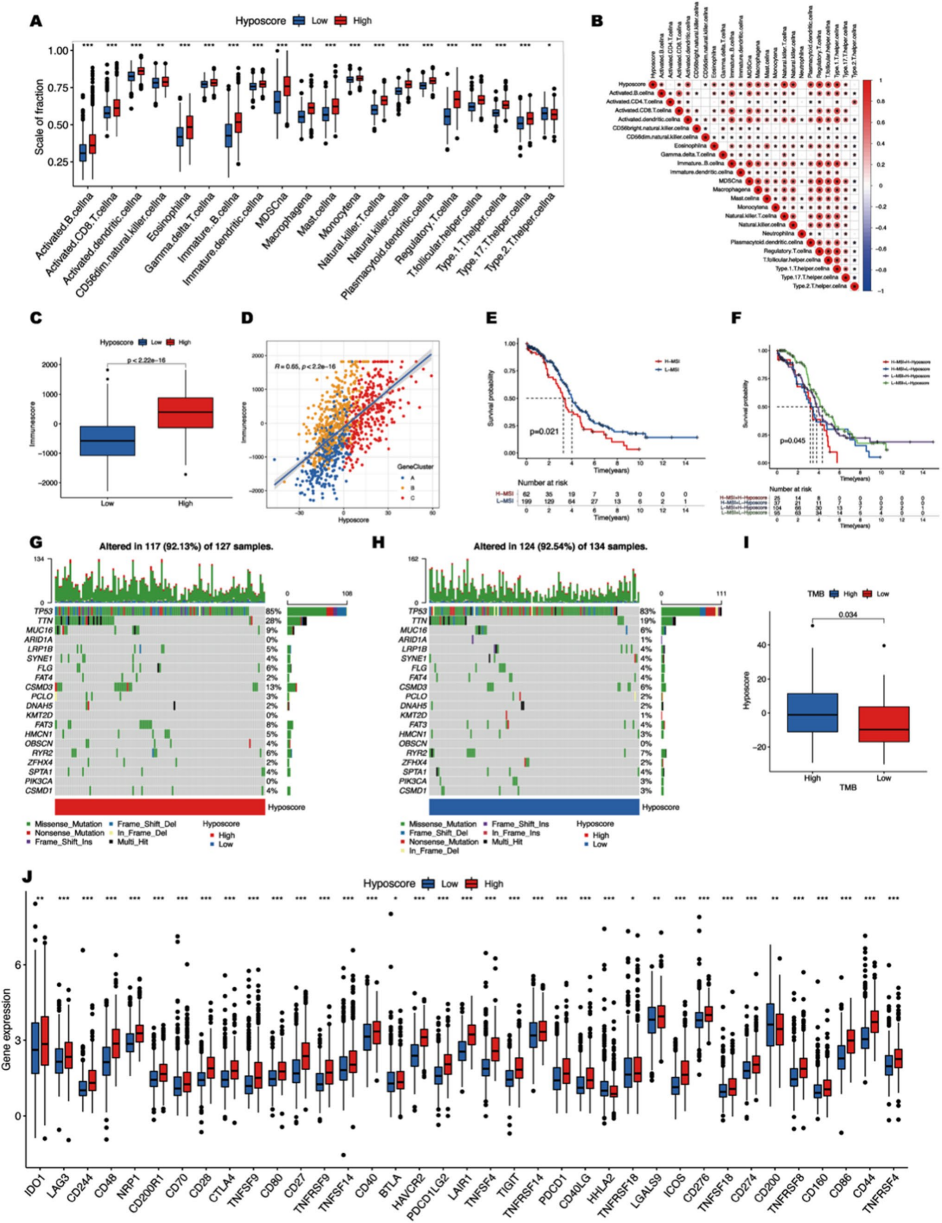
Hypoxic score pattern identified hot tumors in OC. **A** The distribution of immune infiltrating cells in two hypoxic score subgroups. Lines in the boxes represent the median value, and black dots show outliers. The asterisks show the p-value (*p < 0.05; **p < 0.01; ***p < 0.001). **B** The correlation of immune infiltrating cells. (C) Comparisons of immunescores in two hypoxic score subgroups. **D** Scatterplots depicting the positive correlation between hypoxic scores and immunescores in the TCGA-OV cohort. The Spearman correlation between hypoxic scores and immunscores. **E** The survival curve shows survival rates of OC patients in H-MSI and L-MSI groups. **F** The survival curve shows the survival of OC patients in different MSI and hypoxic score subgroups. **G**, **H** The waterfall plot of tumor somatic mutations was constructed using high hypoxic scores (**G**) and low hypoxic scores (**H**). Each column represents an individual patient. The upper barplot shows TMB, the number on the right indicates the mutation frequency for each gene. The right barplot shows the proportion of each variant type. **I** Analysis of hypoxic scores in high and low TMB groups, lines in the boxes represent the median value. **J** Expression of immune checkpoints in high and low hypoxic score subgroups

In this study, we aim to unravel the characteristics of the hypoxia-responsive state to identify two major immunophenotypes—‘hot and cold tumors’ in OC. It was previously reported that TILs and TAMs infiltration are distinguishing features for immunotherapy sensitivity [30]. Kruskal–Wallis test revealed the infiltration degree of all immune cells was significantly different between high and low hypoxia scores groups. Among them, activated CD8^+^ T cells infiltrated more in patients with high hypoxia scores, suggesting that patients with high hypoxia scores may be hot tumors (Fig. 4A). Activated B cells, natural killer T cells, macrophages, etc. also showed a significant positive correlation with hypoxia scores, suggesting that patients with high hypoxia scores had better immunogenicity (Fig. 4B). Some kinds of immunophenotypes were considered to be more responsive to immunotherapies because of a higher number of TILs [31]. We further used the ratio of immune cells and stromal cells as the important indicators for evaluating tumor immunogenicity and hot tumors. Kruskal–Wallis test revealed that patients with high hypoxia scores had higher median immunescore (Fig. 4C). The results of Spearman correlation analysis suggest that there was a significant positive correlation between immunnescore and hypoxia scores (Fig. 4D). It is generally believed that cancer patients with high MSI tend to be hot tumors, although they have a poor prognosis. Survival analysis suggested that patients with high MSI in the TCGA-OV cohort had worse OS (Fig. 4E). The results of the multi-group survival analysis of MSI combined with hypoxia scores indicated that the difference in OS between patients with high MSI plus high hypoxia scores and patients with low MSI plus low hypoxia scores was significantly widened (Fig. 4F). On the other hand, the distribution of somatic mutation between the high and low hypoxic score subgroups in the TCGA-OV cohort revealed that the mutation frequency of top20 frequently mutated genes increased overall in patients with high hypoxia scores (Fig. 4G, H). Kruskal–Wallis test revealed that patients with high hypoxia scores had higher median TMB (Fig. 4I). Hot tumors have a higher potential to benefit from PD-1/PD-L1 blockade or other immunotherapies due to a higher number of TILs [32]. It must be mentioned that considerable expression of immune checkpoints is crucial for defining hot tumors. Further analysis in meta-cohort demonstrated that the hypoxic score pattern was highly correlated with immune characteristics in solid tumors. The results showed that most immune checkpoints, including PD-1, PD-L1, and CTLA4, were highly expressed in the high hypoxia scores group (Fig. 4J). In conclusion, because OC patients with high hypoxia scores had abundant immune cell infiltration, worse synergistic MSI lethality, higher TMB, and higher expression levels of immune checkpoints, we preliminarily consider them as highly immunogenic hot tumors.

### Hypoxic score pattern in the prediction of immunotherapeutic response

**Fig. 5:**
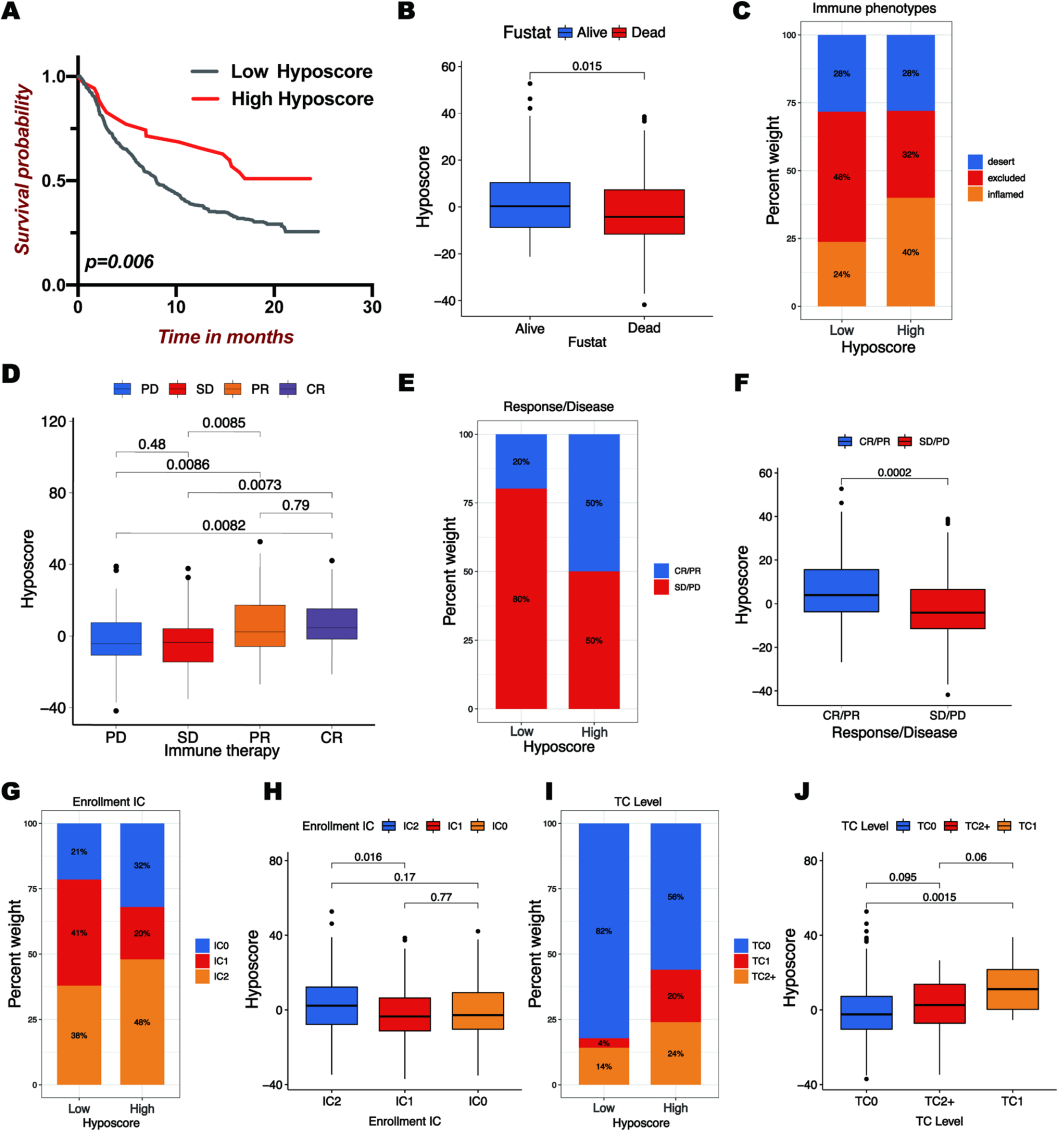
Hypoxic score pattern in the prediction of immunotherapeutic response. **A** The survival curves of patients with low and high hypoxic scores in the IMvigor210 cohort. **B** Comparison of hypoxic scores in different survival statuses. **C** The proportion of patients in various hypoxic score subgroups with different immune phenotypes. **D** Analysis of hypoxic scores in different clinical response outcomes. SD, stable disease; PD, progressive disease; CR, complete response; PR, partial response. **E** The proportion of patients showing response to PD-L1 blockade immunotherapy in low or high hypoxic score subgroups. **F** Analysis of hypoxic scores in CR/PR or SD/PD patients. **G** The proportion of IC0, IC1, and IC2 in low or high hypoxic score subgroups. IC: percentages of PD-L1-positive immune cells. IC0:<1%, IC1: ≥1% but < 5%, IC2:>5%. **H** Analysis of hypoxic scores in different IC0, IC1, or IC2 groups. **I** The proportion of TC0, TC1, and TC2 in low or high hypoxic score subgroups. TC: PD-L1 expression on tumor cells. TC0:<1%; TC1: ≥1% but < 5%; TC2+: >5%. **J** Analysis of hypoxic scores in different TC0, TC1, and TC2 + groups.

**Fig. 6:**
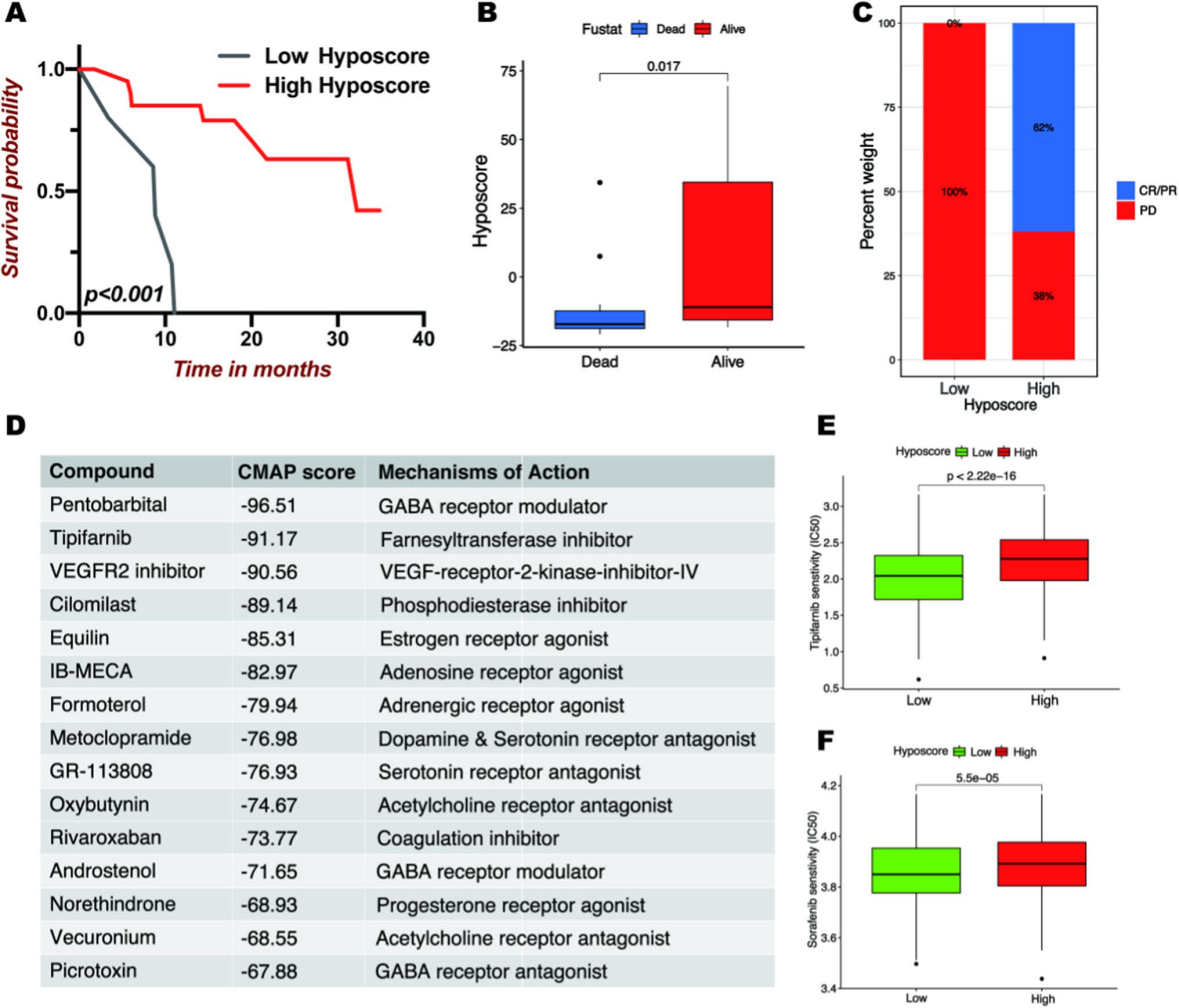
The impact of hypoxia modification patterns on anti-PD-1 immunotherapy and drug sensitivity. **A** The survival curves for the low and high hypoxic score subgroups in GSE78220 cohort. **B** Comparison of hypoxic scores of different survival statuses. **C** The proportion of patients showing response to PD-1 blockade immunotherapy in low or high hypoxic score subgroups. **D** The top 15 chemical compounds in different hypoxic score subgroups were obtained from CMAP analysis based on HRGs. **E**, **F** IC50 of Tipifarnib and Sorafenib in high or low hypoxic score subgroups

The above analysis indicated that patients with high hypoxia scores were considered highly immunogenic hot tumors, suggesting that although these OC patients had a worse clinical prognosis, they may be highly sensitive to immune checkpoint inhibitor therapy. To further evaluate the predictive power of the hypoxic score pattern, we compared the efficacy of immunotherapy with different hypoxic scores in two immunotherapy cohorts, IMvigor210 and GSE78220. As same as the above method, we calculated the hypoxia scores of each case in the two immunotherapy cohorts and then classified them into low or high hypoxia scores groups according to the optimal cutoff value. Unexpectedly, patients in the high hypoxia scores group had significantly prolonged overall survival compared to those in the low hypoxia scores group in both two cohorts (Figs. 5A and 6A). Kruskal-Wallis test revealed that alive cases had higher median hypoxia scores than dead cases (Figs. 5B and 6B). Surprisingly, we found that higher hypoxia scores were remarkably associated with inflamed immune phenotype, which had higher TILs and ICIs were easier to exert an antitumor effect in this phenotype (Fig. 5C). This further illustrated that tumor with high hypoxia scores was a hot tumor. The significant therapeutic advantages and clinical response to anti-PD-L1 immunotherapy in patients with high hypoxia scores compared to those with low hypoxic scores were confirmed (Fig. 5D–F). In the anti-PD-1 cohort, 62% of patients with high hypoxic scores showed complete response or partial response (Fig. 6C). Given the devastating survival rate of patients with high hypoxia scores, this could be a life-saving strategy. The clinical data corresponding to the IMvigor210 cohort included the expression of PD-L1 on immune cells (IC) and tumor cells (TC). We found that the proportion of the IC2 population in the high hypoxic scores subgroup was higher, but the combined proportion of IC2 and IC1 was less than that in the low hypoxic scores subgroup (Fig. 5G). There were only minor differences in hypoxic scores between the three IC groups (Fig. 5H). In contrast, the proportion of total cases of TC1 and TC2 + in the high hypoxic scores subgroup was more than twice that of the low hypoxic scores subgroup (Fig. 5I). The median hyposcores of the TC1 and TC2 + groups with more PD-L1 positive expression were also significantly higher than those of the TC1 group with less PD-L1 positive expression(Fig. 5J).

### Correlation between hypoxic score and chemotherapeutics

Chemotherapy remains the primary treatment strategy in OC other than radical surgical resection. Besides immunotherapy, we attempted to investigate the association between the hypoxic score pattern and chemotherapy. According to the analysis results of CMAP, we obtained and presented the top 15 components, which included two chemotherapeutic drugs Tipifarnib and VEGFR2 inhibitor with a CMAP score lower than − 90 (Fig. 6D). A negative CMAP score indicates insensitivity to chemotherapy, suggesting that tipifarnib and VEGFR2 inhibitors may be less sensitive to chemotherapy in patients with high ss. To verify the results of CMAP, we calculated the half inhibitory centration (IC50) of tipifarnib and VEGFR2 inhibitors for each case in meta-cohort. In this study, we selected Sorafenib as a VEGFR2 inhibitor for further analysis. Kruskal-Wallis test revealed the high hypoxic score was associated with a higher half inhibitory centration (IC_50_) of chemotherapeutics such as Tipifarnib and Sorafenib (Fig. 6E, F). Thus, patients in the low hypoxic score group were more sensitive to chemotherapy.

## Discussion

A recent breakthrough in cancer research is the clinical efficacy of ICIs in selected cancer indications. Unfortunately, the efficacy of immune checkpoint blockade treatment in OC was found to be low, most likely due to the presence of multiple immune inhibitory mechanisms in the TME and a lower TILs level. Although this has created the perception that OC would be a poorly immunogenic, cold tumor, our study has shown that a subgroup of OC patients exhibited the immunophenotype of hot tumors, and they can be identified according to hypoxic characteristics. Due to the rapid growth of tumor cells and disordered angiogenesis, partial pressure of oxygen in cancer tissues is often at low levels, thus showing a hypoxia phenotype. A fertile network of vessels is one of the main characteristics of malignant tumors, but its oxygen supply efficiency is lower than the oxygen demand consumed by the vigorous proliferation and metabolic activities of cancer cells [33]. Every OC solid tumor will respond differently to tolerate a hypoxic microenvironment due to tumor size, different gene expression profiles, etc. However, most studies focus on a single hypoxic marker that can only be functioned in a specific phenotype of a single cell and cannot represent the hypoxia-responsive state and TME infiltration characteristics of the whole tumors.

In this work, we identified two subgroups with markedly different hypoxia-responsive states based on 14 hypoxic markers in OC. There are active metabolic and hypoxia responses in hypocluster B, pointing out that the 14 hypoxic markers in Ye’s study can well reflect the hypoxia-responsive state. However, the nonsense of survival analysis and immune infiltration results showed the limitations of the 14 hypoxic markers. We consider that the hypoxic microenvironment in solid tumors is complex, and only 14 hypoxic markers are insufficient to elucidate the hypoxic characteristics of OC. We further screened HRGs in two hypoclusters and identified 3 geneclusters, which could well evaluate the prognosis and hypoxia-responsive status of OC patients. Considering the individual heterogeneity, it was urgently demanded to quantify the hypoxic environment of individual tumors. We quantified the hypoxia-responsive states by establishing a hypoxic score pattern that could be applied in individual cases. Using the hypoxic score pattern, The hypoxia scores of each OC patient are accurately quantified, and the patient’s clinical prognosis can be assessed based on the hypoxia scores. As demonstrated, the hypoxic score was a robust tool to evaluate hypoxia modification patterns in individual tumors.

Furthermore, we found that the hypoxic score pattern can reveal the immune infiltration signature of OC patients and identify the immunophenotype of hot tumors. Surprisingly, the hypoxic score pattern showed that patients with an active hypoxic response, despite poor prognosis, displayed a highly immunogenic hot tumor immunophenotype. And two independent immunotherapy cohorts showed predictive outcomes, and patients with high hypoxia scores who had a poor overall prognosis had better clinical outcomes in the immunotherapy cohort. We believe the reason for this result is that patients with high hypoxia scores show potential for immunotherapy, and they have higher levels of immune checkpoint expression and immune infiltration abundance. So when receiving immunotherapy, the number of cases in clinical remission is greatly increased. This is exciting because it may provide a life-saving treatment strategy for this subset of OC patients. Although our analysis showed no significant difference in PD-L1 positive lymphocytes between the two hypoxic scores subgroups (Fig. 5G), PD-L1 blockade often acts on tumor cells. The higher content of PD-L1 positive tumor cells in high hypoxic scores subgroups means that they are more sensitive to PD-L1 blockade treatment (Fig. 5I). On the contrary, patients with cold tumors identified by the hypoxia score pattern were unexpectedly found to be sensitive to both tipifarnib and VEGFR2 inhibitors. Our hypoxic score pattern can effectively characterize the hypoxia characteristics of OC patients and identify tumor immunogenicity to distinguish between patients who are sensitive to immunotherapy and those who are sensitive to specific targeted drug therapy. Thus, systematic evaluation of hypoxia-responsive states has crucial clinical implications. Also, it can facilitate the identification of ideal candidates for tailoring optimal immunotherapeutic and chemotherapeutic strategies.

However, we acknowledge some shortcomings and limitations of this study. For example, the cohorts used for analysis were all obtained from public databases, and evaluation of hypoxia-responsive states requires external validation due to the different expression levels of each sample, which may make the final results unreliable. We included more than 1000 OC patients and eliminated batch differences between different cohorts before analysis to minimize sample errors due to expression changes. Based on these results, we hypothesize that our hypoxic score pattern is acceptable despite the lack of external data validation. In addition, we selected IMvigor210 and GSE78220 as external validation to validate the value of the hypoxic score pattern in the identification of hot tumors. Different pathological types of tumors have different immunogenicity, and that would cause certain discrepancies and inaccuracies. Likewise, further in vivo and in vitro experiments are needed to evaluate the sensitivity of OC patients with different hypoxia scores to chemical compound treatment. Therefore, in future work, we will re-collect clinical samples and expand the sample size for further validation, whose evaluation will be time-consuming.

## Conclusion

In conclusion, this work constructed a hypoxic score pattern based on hypoxic genes to accurately assess the hypoxia-responsive state of OC patients. The hypoxic score pattern can be used to assess the clinical prognosis and immune infiltration, and to identify hot tumor immunophenotypes sensitive to immunotherapy and cold tumor immunophenotypes sensitive to chemotherapy. The comprehensive evaluation of individual tumor hypoxia-responsive patterns will contribute to providing precision treatment strategies for OC patients.

### Supplementary Information

**Additional file 1: Table S1.** Basic information of datasets included in this study for identifying distinct hypoxia modification patterns. **Table S2.** The group of 1060 ovarian cancer patients according to 14 hypogenes. **Table S3.** Identification of 4879 DEGs between hypocluster A and B. **Table S4.** Identification of 1203 candidate genes according to Cox univariate analysis. **Table S5.** Identification of hypogene clusters. **Table S6.** The survival information and the hyposcore of 1060 ovarian cancer patients.

**Additional file 2: Figure S1.** K-M survival curves of these fifteen hypoxia-genes.

## Data Availability

The data used in the study can be freely downloaded in TCGA (https://www.cancer.gov/about-nci/organization/ccg/research/structural-genomics/tcga), ICGC (https://dcc.icgc.org/) and GEO (https://www.ncbi.nlm.nih.gov/geo/), GTEx (www.gtexportal.org). The complete expression data and detailed clinical annotations of IMvigor210 were obtained from http://research-pub.Gene.com/imvigor210corebiologies.
