# Supplementary material for: Characterization of hypoxia-responsive states in ovarian cancer to identify hot tumors and aid adjuvant therapy
Source: Discov Oncol. 2024 Jan 31;15:23. doi: 10.1007/s12672-024-00859-8 (PMC10831007; doi:10.1007/s12672-024-00859-8)
Supplement: Supplementary file 1 — Additional file 1: Table S1. Basic information of datasets included in this study for identifying distinct hypoxia modification patterns. Table S2. The group of 1060 ovarian cancer patients according to 14 hypogenes. Table S3. Identification of 4879 DEGs between hypocluster A and B. Table S4. Identification of 1203 candidate genes according to Cox univariate analysis. Table S5. Identification of hypogene clusters. Table S6. The survival information and the hyposcore of 1060 ovarian cancer patients. [file 12672_2024_859_MOESM1_ESM.doc]

Table-S1: Basic information of datasets included in this study for identifying distinct hypoxia modification patterns

| **Accession number /Source** | **Platform** | **Number of  patients** | **Stage** | **Histology** | **Survival data** |
| --- | --- | --- | --- | --- | --- |
| GEO: GSE138866 | HiSeq X Ten | 130 | III/IV:130 | G3:130 | OS/PFS |
| GEO: GSE49997 | ABI Human Genome Survey Microarray Version 2 | 170 | II:5 III/IV:165 | G1&2:36 G3:134 | OS/PFS |
| GEO: GSE51088 | Agilent-012097 Human 1A Microarray (V2) G4110B | 96 | II:10 III/IV:86 | G1&2:5 G3:91 | OS |
| GEO: GSE53963 | Agilent-014850 Whole Human Genome Microarray 4x44K G4112F | 160 | II:7 III/IV:153 | G1&2:3 G3&4:157 | OS |
| GEO: GSE63885 | Affymetrix Human Genome U133 Plus 2.0 Array | 66 | II:7 III&IV:59 | G2:7 G3&4:59 | OS/PFS |
| TCGA:OV | Illumina RNAseq | 363 | I&II:22 III&IV:341 | G1&2:43 G3&4:320 | OS |
| ICGC:OV | Illumina RNAseq | 75 | III/IV:75 | G2:14 G3:61 | OS |

Table-S2: The group of 1060 ovarian cancer patients according to 14 hypogenes.

| ID | hypocluster |
| --- | --- |
| TCGA_TCGA-24-1544 | A |
| TCGA_TCGA-13-0795 | A |
| TCGA_TCGA-13-1411 | A |
| TCGA_TCGA-23-1030 | A |
| TCGA_TCGA-57-1586 | A |
| TCGA_TCGA-36-1568 | A |
| TCGA_TCGA-29-1701 | A |
| TCGA_TCGA-36-1571 | A |
| TCGA_TCGA-24-1426 | A |
| TCGA_TCGA-09-1661 | A |
| TCGA_TCGA-29-A5NZ | A |
| TCGA_TCGA-29-1774 | A |
| TCGA_TCGA-13-0724 | A |
| TCGA_TCGA-23-2077 | A |
| TCGA_TCGA-09-1670 | A |
| TCGA_TCGA-13-1505 | A |
| TCGA_TCGA-25-1632 | A |
| TCGA_TCGA-04-1347 | A |
| TCGA_TCGA-61-1900 | A |
| TCGA_TCGA-24-1930 | A |
| TCGA_TCGA-61-1741 | A |
| TCGA_TCGA-24-1846 | A |
| TCGA_TCGA-29-1690 | A |
| TCGA_TCGA-13-1409 | A |
| TCGA_TCGA-25-1628 | A |
| TCGA_TCGA-13-0888 | A |
| TCGA_TCGA-36-1580 | A |
| TCGA_TCGA-61-1918 | A |
| TCGA_TCGA-13-1485 | A |
| TCGA_TCGA-23-1114 | A |
| TCGA_TCGA-09-1673 | A |
| TCGA_TCGA-24-1565 | A |
| TCGA_TCGA-23-1111 | A |
| TCGA_TCGA-24-2261 | A |
| TCGA_TCGA-31-1959 | A |
| TCGA_TCGA-30-1866 | A |
| TCGA_TCGA-61-2088 | A |
| TCGA_TCGA-25-2396 | A |
| TCGA_TCGA-61-2002 | A |
| TCGA_TCGA-24-2271 | A |
| TCGA_TCGA-25-1633 | A |
| TCGA_TCGA-09-1666 | A |
| TCGA_TCGA-25-2400 | A |
| TCGA_TCGA-13-1404 | A |
| TCGA_TCGA-24-2254 | A |
| TCGA_TCGA-30-1861 | A |
| TCGA_TCGA-13-0730 | A |
| TCGA_TCGA-04-1343 | A |
| TCGA_TCGA-25-1316 | A |
| TCGA_TCGA-25-2401 | A |
| TCGA_TCGA-24-2289 | A |
| TCGA_TCGA-29-1768 | A |
| TCGA_TCGA-61-1733 | A |
| TCGA_TCGA-09-1662 | A |
| TCGA_TCGA-04-1648 | A |
| TCGA_TCGA-24-1928 | A |
| TCGA_TCGA-24-2027 | A |
| TCGA_TCGA-29-2425 | A |
| TCGA_TCGA-23-1024 | A |
| TCGA_TCGA-29-1710 | A |
| TCGA_TCGA-13-0804 | A |
| TCGA_TCGA-25-1312 | A |
| TCGA_TCGA-61-2102 | A |
| TCGA_TCGA-09-2056 | A |
| TCGA_TCGA-09-1668 | A |
| TCGA_TCGA-23-1109 | A |
| TCGA_TCGA-09-2048 | A |
| TCGA_TCGA-25-1313 | A |
| TCGA_TCGA-23-1023 | A |
| TCGA_TCGA-25-1328 | A |
| TCGA_TCGA-13-0884 | A |
| TCGA_TCGA-61-1736 | A |
| TCGA_TCGA-04-1514 | A |
| TCGA_TCGA-29-1763 | A |
| TCGA_TCGA-24-1464 | A |
| TCGA_TCGA-24-1923 | A |
| TCGA_TCGA-24-2036 | A |
| TCGA_TCGA-24-1422 | A |
| TCGA_TCGA-25-1634 | A |
| TCGA_TCGA-29-1695 | A |
| TCGA_TCGA-30-1714 | A |
| TCGA_TCGA-24-1424 | A |
| TCGA_TCGA-24-1474 | A |
| TCGA_TCGA-24-1427 | A |
| TCGA_TCGA-61-2111 | A |
| TCGA_TCGA-10-0927 | A |
| TCGA_TCGA-09-2051 | A |
| TCGA_TCGA-13-2060 | A |
| TCGA_TCGA-13-0924 | A |
| TCGA_TCGA-57-1584 | A |
| TCGA_TCGA-24-1553 | A |
| TCGA_TCGA-WR-A838 | A |
| TCGA_TCGA-24-2280 | A |
| TCGA_TCGA-13-1499 | A |
| TCGA_TCGA-61-1995 | A |
| TCGA_TCGA-25-2398 | A |
| TCGA_TCGA-61-1738 | A |
| TCGA_TCGA-13-1496 | A |
| TCGA_TCGA-29-1711 | A |
| TCGA_TCGA-59-2354 | A |
| TCGA_TCGA-61-1721 | A |
| TCGA_TCGA-59-2352 | A |
| TCGA_TCGA-29-1785 | A |
| TCGA_TCGA-24-1567 | A |
| TCGA_TCGA-24-2024 | A |
| TCGA_TCGA-09-0364 | A |
| TCGA_TCGA-10-0936 | A |
| TCGA_TCGA-13-0885 | A |
| TCGA_TCGA-04-1655 | A |
| TCGA_TCGA-36-1569 | A |
| TCGA_TCGA-29-1778 | A |
| TCGA_TCGA-24-1471 | A |
| TCGA_TCGA-13-0920 | A |
| TCGA_TCGA-24-0968 | A |
| TCGA_TCGA-24-1843 | A |
| TCGA_TCGA-04-1332 | A |
| TCGA_TCGA-OY-A56Q | A |
| TCGA_TCGA-29-1703 | A |
| TCGA_TCGA-23-1123 | A |
| TCGA_TCGA-09-2054 | A |
| TCGA_TCGA-61-2009 | A |
| TCGA_TCGA-25-2393 | A |
| TCGA_TCGA-09-2045 | A |
| TCGA_TCGA-29-1691 | A |
| TCGA_TCGA-25-1870 | A |
| TCGA_TCGA-13-0800 | A |
| TCGA_TCGA-24-1431 | A |
| TCGA_TCGA-04-1361 | A |
| TCGA_TCGA-13-0916 | A |
| TCGA_TCGA-61-2003 | A |
| TCGA_TCGA-25-2409 | A |
| TCGA_TCGA-30-1853 | A |
| TCGA_TCGA-25-1626 | A |
| TCGA_TCGA-13-0714 | A |
| TCGA_TCGA-25-1321 | A |
| TCGA_TCGA-57-1993 | A |
| TCGA_TCGA-13-0886 | A |
| TCGA_TCGA-09-0369 | A |
| TCGA_TCGA-24-1434 | A |
| TCGA_TCGA-23-1022 | A |
| TCGA_TCGA-13-0727 | A |
| TCGA_TCGA-59-2351 | A |
| TCGA_TCGA-24-1470 | A |
| TCGA_TCGA-25-2042 | A |
| TCGA_TCGA-13-0923 | A |
| TCGA_TCGA-13-1477 | A |
| TCGA_TCGA-09-2053 | A |
| TCGA_TCGA-29-1776 | A |
| TCGA_TCGA-29-1783 | A |
| TCGA_TCGA-10-0933 | A |
| TCGA_TCGA-24-0966 | A |
| TCGA_TCGA-36-1576 | A |
| TCGA_TCGA-23-1021 | A |
| TCGA_TCGA-24-1467 | A |
| TCGA_TCGA-13-0883 | A |
| TCGA_TCGA-24-1604 | A |
| TCGA_TCGA-57-1582 | A |
| TCGA_TCGA-13-0762 | A |
| TCGA_TCGA-04-1542 | A |
| TCGA_TCGA-24-2035 | A |
| TCGA_TCGA-13-0908 | A |
| TCGA_TCGA-24-0970 | A |
| TCGA_TCGA-04-1364 | A |
| TCGA_TCGA-20-1687 | A |
| TCGA_TCGA-25-1877 | A |
| TCGA_TCGA-29-2414 | A |
| GSE138866_GSM4120700 | A |
| GSE138866_GSM4120699 | A |
| GSE138866_GSM4120698 | A |
| GSE138866_GSM4120694 | A |
| GSE138866_GSM4120692 | A |
| GSE138866_GSM4120690 | A |
| GSE138866_GSM4120720 | A |
| GSE138866_GSM4120686 | A |
| GSE138866_GSM4120718 | A |
| GSE138866_GSM4120717 | A |
| GSE138866_GSM4120716 | A |
| GSE138866_GSM4120715 | A |
| GSE138866_GSM4120713 | A |
| GSE138866_GSM4120680 | A |
| GSE138866_GSM4120712 | A |
| GSE138866_GSM4120678 | A |
| GSE138866_GSM4120673 | A |
| GSE138866_GSM4120672 | A |
| GSE138866_GSM4120670 | A |
| GSE138866_GSM4120669 | A |
| GSE138866_GSM4120667 | A |
| GSE138866_GSM4120664 | A |
| GSE138866_GSM4120663 | A |
| GSE138866_GSM4120662 | A |
| GSE138866_GSM4120661 | A |
| GSE138866_GSM4120660 | A |
| GSE138866_GSM4120659 | A |
| GSE138866_GSM4120658 | A |
| GSE138866_GSM4120654 | A |
| GSE138866_GSM4120653 | A |
| GSE138866_GSM4120652 | A |
| GSE138866_GSM4120651 | A |
| GSE138866_GSM4120650 | A |
| GSE138866_GSM4120649 | A |
| GSE138866_GSM4120648 | A |
| GSE138866_GSM4120647 | A |
| GSE138866_GSM4120641 | A |
| GSE138866_GSM4120638 | A |
| GSE138866_GSM4120637 | A |
| GSE138866_GSM4120633 | A |
| GSE138866_GSM4120631 | A |
| GSE138866_GSM4120629 | A |
| GSE138866_GSM4120628 | A |
| GSE138866_GSM4120627 | A |
| GSE138866_GSM4120626 | A |
| GSE138866_GSM4120625 | A |
| GSE138866_GSM4120621 | A |
| GSE138866_GSM4120617 | A |
| GSE138866_GSM4120616 | A |
| GSE138866_GSM4120611 | A |
| GSE138866_GSM4120609 | A |
| GSE138866_GSM4120608 | A |
| GSE138866_GSM4120707 | A |
| GSE138866_GSM4120706 | A |
| GSE138866_GSM4120606 | A |
| GSE138866_GSM4120703 | A |
| GSE138866_GSM4120708 | A |
| GSE138866_GSM4120702 | A |
| GSE138866_GSM4120604 | A |
| GSE138866_GSM4120709 | A |
| GSE138866_GSM4120602 | A |
| GSE138866_GSM4120600 | A |
| GSE138866_GSM4120710 | A |
| GSE138866_GSM4120596 | A |
| GSE138866_GSM4120594 | A |
| GSE138866_GSM4120593 | A |
| ICGC_DO46326 | A |
| ICGC_DO46328 | A |
| ICGC_DO46331 | A |
| ICGC_DO46333 | A |
| ICGC_DO46334 | A |
| ICGC_DO46338 | A |
| ICGC_DO46340 | A |
| ICGC_DO46350 | A |
| ICGC_DO46352 | A |
| ICGC_DO46356 | A |
| ICGC_DO46364 | A |
| ICGC_DO46370 | A |
| ICGC_DO46378 | A |
| ICGC_DO46384 | A |
| ICGC_DO46386 | A |
| ICGC_DO46388 | A |
| ICGC_DO46390 | A |
| ICGC_DO46392 | A |
| ICGC_DO46394 | A |
| ICGC_DO46396 | A |
| ICGC_DO46398 | A |
| ICGC_DO46400 | A |
| ICGC_DO46412 | A |
| ICGC_DO46438 | A |
| ICGC_DO46443 | A |
| ICGC_DO46458 | A |
| ICGC_DO46463 | A |
| ICGC_DO46468 | A |
| ICGC_DO46518 | A |
| ICGC_DO46560 | A |
| ICGC_DO46571 | A |
| ICGC_DO46576 | A |
| ICGC_DO46586 | A |
| ICGC_DO46588 | A |
| ICGC_DO46597 | A |
| ICGC_DO46602 | A |
| GSE51088_GSM1238147 | A |
| GSE51088_GSM1238148 | A |
| GSE51088_GSM1238149 | A |
| GSE51088_GSM1238151 | A |
| GSE51088_GSM1238154 | A |
| GSE51088_GSM1238156 | A |
| GSE51088_GSM1238158 | A |
| GSE51088_GSM1238163 | A |
| GSE51088_GSM1238165 | A |
| GSE51088_GSM1238169 | A |
| GSE51088_GSM1238171 | A |
| GSE51088_GSM1238173 | A |
| GSE51088_GSM1238174 | A |
| GSE51088_GSM1238179 | A |
| GSE51088_GSM1238184 | A |
| GSE51088_GSM1238188 | A |
| GSE51088_GSM1238190 | A |
| GSE51088_GSM1238191 | A |
| GSE51088_GSM1238195 | A |
| GSE51088_GSM1238197 | A |
| GSE51088_GSM1238200 | A |
| GSE51088_GSM1238201 | A |
| GSE51088_GSM1238208 | A |
| GSE51088_GSM1238211 | A |
| GSE51088_GSM1238213 | A |
| GSE51088_GSM1238216 | A |
| GSE51088_GSM1238223 | A |
| GSE51088_GSM1238224 | A |
| GSE51088_GSM1238226 | A |
| GSE51088_GSM1238227 | A |
| GSE51088_GSM1238236 | A |
| GSE51088_GSM1238245 | A |
| GSE51088_GSM1238246 | A |
| GSE51088_GSM1238257 | A |
| GSE51088_GSM1238260 | A |
| GSE51088_GSM1238261 | A |
| GSE51088_GSM1238263 | A |
| GSE51088_GSM1238270 | A |
| GSE51088_GSM1238272 | A |
| GSE51088_GSM1238273 | A |
| GSE51088_GSM1238278 | A |
| GSE51088_GSM1238283 | A |
| GSE51088_GSM1238290 | A |
| GSE51088_GSM1238292 | A |
| GSE51088_GSM1238297 | A |
| GSE51088_GSM1238304 | A |
| GSE51088_GSM1238305 | A |
| GSE51088_GSM1238314 | A |
| GSE51088_GSM1238315 | A |
| GSE53963_GSM1304248 | A |
| GSE53963_GSM1304249 | A |
| GSE53963_GSM1304250 | A |
| GSE53963_GSM1304251 | A |
| GSE53963_GSM1304252 | A |
| GSE53963_GSM1304254 | A |
| GSE53963_GSM1304255 | A |
| GSE53963_GSM1304257 | A |
| GSE53963_GSM1304258 | A |
| GSE53963_GSM1304259 | A |
| GSE53963_GSM1304260 | A |
| GSE53963_GSM1304262 | A |
| GSE53963_GSM1304266 | A |
| GSE53963_GSM1304267 | A |
| GSE53963_GSM1304270 | A |
| GSE53963_GSM1304271 | A |
| GSE53963_GSM1304278 | A |
| GSE53963_GSM1304279 | A |
| GSE53963_GSM1304280 | A |
| GSE53963_GSM1304281 | A |
| GSE53963_GSM1304283 | A |
| GSE53963_GSM1304284 | A |
| GSE53963_GSM1304289 | A |
| GSE53963_GSM1304290 | A |
| GSE53963_GSM1304292 | A |
| GSE53963_GSM1304294 | A |
| GSE53963_GSM1304297 | A |
| GSE53963_GSM1304299 | A |
| GSE53963_GSM1304303 | A |
| GSE53963_GSM1304305 | A |
| GSE53963_GSM1304311 | A |
| GSE53963_GSM1304312 | A |
| GSE53963_GSM1304317 | A |
| GSE53963_GSM1304318 | A |
| GSE53963_GSM1304323 | A |
| GSE53963_GSM1304327 | A |
| GSE53963_GSM1304329 | A |
| GSE53963_GSM1304331 | A |
| GSE53963_GSM1304334 | A |
| GSE53963_GSM1304335 | A |
| GSE53963_GSM1304336 | A |
| GSE53963_GSM1304337 | A |
| GSE53963_GSM1304339 | A |
| GSE53963_GSM1304343 | A |
| GSE53963_GSM1304344 | A |
| GSE53963_GSM1304345 | A |
| GSE53963_GSM1304346 | A |
| GSE53963_GSM1304347 | A |
| GSE53963_GSM1304348 | A |
| GSE53963_GSM1304350 | A |
| GSE53963_GSM1304351 | A |
| GSE53963_GSM1304353 | A |
| GSE53963_GSM1304358 | A |
| GSE53963_GSM1304359 | A |
| GSE53963_GSM1304360 | A |
| GSE53963_GSM1304363 | A |
| GSE53963_GSM1304364 | A |
| GSE53963_GSM1304365 | A |
| GSE53963_GSM1304366 | A |
| GSE53963_GSM1304367 | A |
| GSE53963_GSM1304369 | A |
| GSE53963_GSM1304370 | A |
| GSE53963_GSM1304371 | A |
| GSE53963_GSM1304374 | A |
| GSE53963_GSM1304376 | A |
| GSE53963_GSM1304378 | A |
| GSE53963_GSM1304382 | A |
| GSE53963_GSM1304385 | A |
| GSE53963_GSM1304389 | A |
| GSE53963_GSM1304395 | A |
| GSE53963_GSM1304397 | A |
| GSE53963_GSM1304398 | A |
| GSE53963_GSM1304402 | A |
| GSE53963_GSM1304404 | A |
| GSE53963_GSM1304410 | A |
| GSE53963_GSM1304411 | A |
| GSE49997_GSM1211536 | A |
| GSE49997_GSM1211537 | A |
| GSE49997_GSM1211539 | A |
| GSE49997_GSM1211540 | A |
| GSE49997_GSM1211541 | A |
| GSE49997_GSM1211550 | A |
| GSE49997_GSM1211553 | A |
| GSE49997_GSM1211555 | A |
| GSE49997_GSM1211556 | A |
| GSE49997_GSM1211558 | A |
| GSE49997_GSM1211561 | A |
| GSE49997_GSM1211565 | A |
| GSE49997_GSM1211566 | A |
| GSE49997_GSM1211569 | A |
| GSE49997_GSM1211570 | A |
| GSE49997_GSM1211571 | A |
| GSE49997_GSM1211573 | A |
| GSE49997_GSM1211574 | A |
| GSE49997_GSM1211576 | A |
| GSE49997_GSM1211577 | A |
| GSE49997_GSM1211579 | A |
| GSE49997_GSM1211582 | A |
| GSE49997_GSM1211583 | A |
| GSE49997_GSM1211585 | A |
| GSE49997_GSM1211586 | A |
| GSE49997_GSM1211593 | A |
| GSE49997_GSM1211594 | A |
| GSE49997_GSM1211595 | A |
| GSE49997_GSM1211596 | A |
| GSE49997_GSM1211597 | A |
| GSE49997_GSM1211598 | A |
| GSE49997_GSM1211602 | A |
| GSE49997_GSM1211603 | A |
| GSE49997_GSM1211604 | A |
| GSE49997_GSM1211607 | A |
| GSE49997_GSM1211608 | A |
| GSE49997_GSM1211610 | A |
| GSE49997_GSM1211611 | A |
| GSE49997_GSM1211612 | A |
| GSE49997_GSM1211614 | A |
| GSE49997_GSM1211616 | A |
| GSE49997_GSM1211617 | A |
| GSE49997_GSM1211620 | A |
| GSE49997_GSM1211621 | A |
| GSE49997_GSM1211623 | A |
| GSE49997_GSM1211624 | A |
| GSE49997_GSM1211628 | A |
| GSE49997_GSM1211630 | A |
| GSE49997_GSM1211632 | A |
| GSE49997_GSM1211634 | A |
| GSE49997_GSM1211635 | A |
| GSE49997_GSM1211638 | A |
| GSE49997_GSM1211640 | A |
| GSE49997_GSM1211641 | A |
| GSE49997_GSM1211652 | A |
| GSE49997_GSM1211655 | A |
| GSE49997_GSM1211657 | A |
| GSE49997_GSM1211658 | A |
| GSE49997_GSM1211660 | A |
| GSE49997_GSM1211662 | A |
| GSE49997_GSM1211663 | A |
| GSE49997_GSM1211669 | A |
| GSE49997_GSM1211670 | A |
| GSE49997_GSM1211671 | A |
| GSE49997_GSM1211672 | A |
| GSE49997_GSM1211673 | A |
| GSE49997_GSM1211674 | A |
| GSE49997_GSM1211675 | A |
| GSE49997_GSM1211676 | A |
| GSE49997_GSM1211677 | A |
| GSE49997_GSM1211681 | A |
| GSE49997_GSM1211684 | A |
| GSE49997_GSM1211686 | A |
| GSE49997_GSM1211688 | A |
| GSE49997_GSM1211689 | A |
| GSE49997_GSM1211691 | A |
| GSE49997_GSM1211693 | A |
| GSE49997_GSM1211694 | A |
| GSE49997_GSM1211697 | A |
| GSE49997_GSM1211698 | A |
| GSE49997_GSM1211701 | A |
| GSE49997_GSM1211702 | A |
| GSE49997_GSM1211704 | A |
| GSE49997_GSM1211707 | A |
| GSE49997_GSM1211708 | A |
| GSE49997_GSM1211711 | A |
| GSE49997_GSM1211717 | A |
| GSE49997_GSM1211720 | A |
| GSE49997_GSM1211729 | A |
| GSE49997_GSM1211730 | A |
| GSE49997_GSM1211733 | A |
| GSE49997_GSM1211736 | A |
| GSE63885_GSM1559299 | A |
| GSE63885_GSM1559301 | A |
| GSE63885_GSM1559302 | A |
| GSE63885_GSM1559304 | A |
| GSE63885_GSM1559306 | A |
| GSE63885_GSM1559307 | A |
| GSE63885_GSM1559308 | A |
| GSE63885_GSM1559312 | A |
| GSE63885_GSM1559316 | A |
| GSE63885_GSM1559320 | A |
| GSE63885_GSM1559321 | A |
| GSE63885_GSM1559322 | A |
| GSE63885_GSM1559323 | A |
| GSE63885_GSM1559326 | A |
| GSE63885_GSM1559327 | A |
| GSE63885_GSM1559328 | A |
| GSE63885_GSM1559329 | A |
| GSE63885_GSM1559335 | A |
| GSE63885_GSM1559336 | A |
| GSE63885_GSM1559337 | A |
| GSE63885_GSM1559341 | A |
| GSE63885_GSM1559343 | A |
| GSE63885_GSM1559344 | A |
| GSE63885_GSM1559345 | A |
| GSE63885_GSM1559346 | A |
| GSE63885_GSM1559347 | A |
| GSE63885_GSM1559348 | A |
| GSE63885_GSM1559354 | A |
| GSE63885_GSM1559369 | A |
| TCGA_TCGA-24-2267 | B |
| TCGA_TCGA-25-1329 | B |
| TCGA_TCGA-24-2288 | B |
| TCGA_TCGA-13-1511 | B |
| TCGA_TCGA-29-2428 | B |
| TCGA_TCGA-30-1862 | B |
| TCGA_TCGA-09-2044 | B |
| TCGA_TCGA-24-1603 | B |
| TCGA_TCGA-13-1407 | B |
| TCGA_TCGA-25-1635 | B |
| TCGA_TCGA-25-2392 | B |
| TCGA_TCGA-09-1669 | B |
| TCGA_TCGA-29-1696 | B |
| TCGA_TCGA-24-1413 | B |
| TCGA_TCGA-24-1560 | B |
| TCGA_TCGA-23-1028 | B |
| TCGA_TCGA-61-1737 | B |
| TCGA_TCGA-61-1914 | B |
| TCGA_TCGA-09-1659 | B |
| TCGA_TCGA-25-1318 | B |
| TCGA_TCGA-04-1536 | B |
| TCGA_TCGA-61-1910 | B |
| TCGA_TCGA-24-0979 | B |
| TCGA_TCGA-61-2104 | B |
| TCGA_TCGA-20-0987 | B |
| TCGA_TCGA-31-1944 | B |
| TCGA_TCGA-29-1766 | B |
| TCGA_TCGA-24-1425 | B |
| TCGA_TCGA-04-1362 | B |
| TCGA_TCGA-29-1769 | B |
| TCGA_TCGA-25-1623 | B |
| TCGA_TCGA-59-2355 | B |
| TCGA_TCGA-29-2427 | B |
| TCGA_TCGA-23-1026 | B |
| TCGA_TCGA-24-1419 | B |
| TCGA_TCGA-24-1469 | B |
| TCGA_TCGA-13-0887 | B |
| TCGA_TCGA-13-1506 | B |
| TCGA_TCGA-23-1118 | B |
| TCGA_TCGA-24-1550 | B |
| TCGA_TCGA-24-2026 | B |
| TCGA_TCGA-13-0797 | B |
| TCGA_TCGA-23-1116 | B |
| TCGA_TCGA-25-1322 | B |
| TCGA_TCGA-24-1842 | B |
| TCGA_TCGA-24-1558 | B |
| TCGA_TCGA-25-1323 | B |
| TCGA_TCGA-09-0366 | B |
| TCGA_TCGA-13-1408 | B |
| TCGA_TCGA-13-0906 | B |
| TCGA_TCGA-25-2391 | B |
| TCGA_TCGA-24-1435 | B |
| TCGA_TCGA-29-1762 | B |
| TCGA_TCGA-61-2113 | B |
| TCGA_TCGA-20-0991 | B |
| TCGA_TCGA-25-1319 | B |
| TCGA_TCGA-24-1104 | B |
| TCGA_TCGA-23-1120 | B |
| TCGA_TCGA-24-1552 | B |
| TCGA_TCGA-25-1631 | B |
| TCGA_TCGA-13-0905 | B |
| TCGA_TCGA-24-1924 | B |
| TCGA_TCGA-31-1953 | B |
| TCGA_TCGA-23-1809 | B |
| TCGA_TCGA-61-2092 | B |
| TCGA_TCGA-29-1777 | B |
| TCGA_TCGA-30-1892 | B |
| TCGA_TCGA-61-2098 | B |
| TCGA_TCGA-13-1405 | B |
| TCGA_TCGA-30-1860 | B |
| TCGA_TCGA-36-1581 | B |
| TCGA_TCGA-13-0893 | B |
| TCGA_TCGA-13-0913 | B |
| TCGA_TCGA-04-1651 | B |
| TCGA_TCGA-20-1683 | B |
| TCGA_TCGA-24-1557 | B |
| TCGA_TCGA-29-1770 | B |
| TCGA_TCGA-25-1320 | B |
| TCGA_TCGA-23-1027 | B |
| TCGA_TCGA-61-2012 | B |
| TCGA_TCGA-24-1546 | B |
| TCGA_TCGA-24-1423 | B |
| TCGA_TCGA-13-0900 | B |
| TCGA_TCGA-29-1705 | B |
| TCGA_TCGA-10-0938 | B |
| TCGA_TCGA-09-1667 | B |
| TCGA_TCGA-24-1418 | B |
| TCGA_TCGA-29-1761 | B |
| TCGA_TCGA-24-1847 | B |
| TCGA_TCGA-24-2033 | B |
| TCGA_TCGA-20-1686 | B |
| TCGA_TCGA-24-1844 | B |
| TCGA_TCGA-24-2262 | B |
| TCGA_TCGA-61-2000 | B |
| TCGA_TCGA-31-1956 | B |
| TCGA_TCGA-61-2109 | B |
| TCGA_TCGA-24-1549 | B |
| TCGA_TCGA-04-1350 | B |
| TCGA_TCGA-57-1583 | B |
| TCGA_TCGA-10-0937 | B |
| TCGA_TCGA-30-1891 | B |
| TCGA_TCGA-23-1119 | B |
| TCGA_TCGA-10-0928 | B |
| TCGA_TCGA-25-1315 | B |
| TCGA_TCGA-31-1946 | B |
| TCGA_TCGA-30-1718 | B |
| TCGA_TCGA-36-1570 | B |
| TCGA_TCGA-24-1850 | B |
| TCGA_TCGA-24-1103 | B |
| TCGA_TCGA-24-2020 | B |
| TCGA_TCGA-30-1857 | B |
| TCGA_TCGA-61-1998 | B |
| TCGA_TCGA-29-1784 | B |
| TCGA_TCGA-23-1107 | B |
| TCGA_TCGA-24-2290 | B |
| TCGA_TCGA-57-1585 | B |
| TCGA_TCGA-24-2297 | B |
| TCGA_TCGA-25-1326 | B |
| TCGA_TCGA-31-1950 | B |
| TCGA_TCGA-13-1488 | B |
| TCGA_TCGA-36-1574 | B |
| TCGA_TCGA-23-2084 | B |
| TCGA_TCGA-13-0901 | B |
| TCGA_TCGA-25-2404 | B |
| TCGA_TCGA-04-1338 | B |
| TCGA_TCGA-13-0765 | B |
| TCGA_TCGA-24-1616 | B |
| TCGA_TCGA-24-2023 | B |
| TCGA_TCGA-13-1512 | B |
| TCGA_TCGA-13-1497 | B |
| TCGA_TCGA-61-2097 | B |
| TCGA_TCGA-13-0891 | B |
| TCGA_TCGA-24-1430 | B |
| TCGA_TCGA-23-1122 | B |
| TCGA_TCGA-04-1365 | B |
| TCGA_TCGA-13-1492 | B |
| TCGA_TCGA-13-0720 | B |
| TCGA_TCGA-61-1907 | B |
| TCGA_TCGA-29-1693 | B |
| TCGA_TCGA-61-2101 | B |
| TCGA_TCGA-13-0911 | B |
| TCGA_TCGA-13-1483 | B |
| TCGA_TCGA-29-1781 | B |
| TCGA_TCGA-23-1029 | B |
| TCGA_TCGA-13-1495 | B |
| TCGA_TCGA-13-A5FT | B |
| TCGA_TCGA-24-0982 | B |
| TCGA_TCGA-31-1951 | B |
| TCGA_TCGA-13-1501 | B |
| TCGA_TCGA-13-0897 | B |
| TCGA_TCGA-61-1728 | B |
| TCGA_TCGA-04-1331 | B |
| TCGA_TCGA-29-1697 | B |
| TCGA_TCGA-25-2399 | B |
| TCGA_TCGA-23-2078 | B |
| TCGA_TCGA-61-1725 | B |
| TCGA_TCGA-29-1688 | B |
| TCGA_TCGA-09-1665 | B |
| TCGA_TCGA-13-0766 | B |
| TCGA_TCGA-13-1498 | B |
| TCGA_TCGA-04-1519 | B |
| TCGA_TCGA-59-2363 | B |
| TCGA_TCGA-23-1113 | B |
| TCGA_TCGA-24-1428 | B |
| TCGA_TCGA-04-1530 | B |
| TCGA_TCGA-25-1317 | B |
| TCGA_TCGA-24-1417 | B |
| TCGA_TCGA-09-0367 | B |
| TCGA_TCGA-59-2350 | B |
| TCGA_TCGA-13-1403 | B |
| TCGA_TCGA-13-1510 | B |
| TCGA_TCGA-13-1507 | B |
| TCGA_TCGA-24-1416 | B |
| TCGA_TCGA-10-0931 | B |
| TCGA_TCGA-36-1577 | B |
| TCGA_TCGA-04-1356 | B |
| TCGA_TCGA-24-1105 | B |
| TCGA_TCGA-61-1911 | B |
| TCGA_TCGA-24-1551 | B |
| TCGA_TCGA-24-2298 | B |
| TCGA_TCGA-61-1919 | B |
| TCGA_TCGA-23-1110 | B |
| TCGA_TCGA-24-1845 | B |
| TCGA_TCGA-59-2348 | B |
| TCGA_TCGA-61-1724 | B |
| TCGA_TCGA-25-1630 | B |
| TCGA_TCGA-13-0768 | B |
| TCGA_TCGA-59-A5PD | B |
| TCGA_TCGA-13-1509 | B |
| TCGA_TCGA-24-1562 | B |
| TCGA_TCGA-13-0725 | B |
| TCGA_TCGA-29-1707 | B |
| TCGA_TCGA-24-1563 | B |
| TCGA_TCGA-25-1627 | B |
| TCGA_TCGA-24-2281 | B |
| TCGA_TCGA-13-1489 | B |
| TCGA_TCGA-61-2008 | B |
| GSE138866_GSM4120701 | B |
| GSE138866_GSM4120697 | B |
| GSE138866_GSM4120696 | B |
| GSE138866_GSM4120695 | B |
| GSE138866_GSM4120693 | B |
| GSE138866_GSM4120691 | B |
| GSE138866_GSM4120689 | B |
| GSE138866_GSM4120719 | B |
| GSE138866_GSM4120688 | B |
| GSE138866_GSM4120687 | B |
| GSE138866_GSM4120685 | B |
| GSE138866_GSM4120684 | B |
| GSE138866_GSM4120683 | B |
| GSE138866_GSM4120682 | B |
| GSE138866_GSM4120714 | B |
| GSE138866_GSM4120681 | B |
| GSE138866_GSM4120679 | B |
| GSE138866_GSM4120677 | B |
| GSE138866_GSM4120676 | B |
| GSE138866_GSM4120675 | B |
| GSE138866_GSM4120674 | B |
| GSE138866_GSM4120671 | B |
| GSE138866_GSM4120668 | B |
| GSE138866_GSM4120666 | B |
| GSE138866_GSM4120665 | B |
| GSE138866_GSM4120657 | B |
| GSE138866_GSM4120656 | B |
| GSE138866_GSM4120655 | B |
| GSE138866_GSM4120645 | B |
| GSE138866_GSM4120644 | B |
| GSE138866_GSM4120643 | B |
| GSE138866_GSM4120642 | B |
| GSE138866_GSM4120640 | B |
| GSE138866_GSM4120639 | B |
| GSE138866_GSM4120636 | B |
| GSE138866_GSM4120635 | B |
| GSE138866_GSM4120634 | B |
| GSE138866_GSM4120632 | B |
| GSE138866_GSM4120630 | B |
| GSE138866_GSM4120624 | B |
| GSE138866_GSM4120623 | B |
| GSE138866_GSM4120622 | B |
| GSE138866_GSM4120620 | B |
| GSE138866_GSM4120619 | B |
| GSE138866_GSM4120618 | B |
| GSE138866_GSM4120615 | B |
| GSE138866_GSM4120614 | B |
| GSE138866_GSM4120613 | B |
| GSE138866_GSM4120612 | B |
| GSE138866_GSM4120610 | B |
| GSE138866_GSM4120607 | B |
| GSE138866_GSM4120704 | B |
| GSE138866_GSM4120705 | B |
| GSE138866_GSM4120605 | B |
| GSE138866_GSM4120711 | B |
| GSE138866_GSM4120646 | B |
| GSE138866_GSM4120603 | B |
| GSE138866_GSM4120601 | B |
| GSE138866_GSM4120599 | B |
| GSE138866_GSM4120598 | B |
| GSE138866_GSM4120597 | B |
| GSE138866_GSM4120595 | B |
| GSE138866_GSM4120592 | B |
| GSE138866_GSM4120591 | B |
| ICGC_DO46325 | B |
| ICGC_DO46327 | B |
| ICGC_DO46329 | B |
| ICGC_DO46330 | B |
| ICGC_DO46332 | B |
| ICGC_DO46336 | B |
| ICGC_DO46342 | B |
| ICGC_DO46344 | B |
| ICGC_DO46346 | B |
| ICGC_DO46348 | B |
| ICGC_DO46354 | B |
| ICGC_DO46358 | B |
| ICGC_DO46360 | B |
| ICGC_DO46362 | B |
| ICGC_DO46374 | B |
| ICGC_DO46376 | B |
| ICGC_DO46382 | B |
| ICGC_DO46402 | B |
| ICGC_DO46404 | B |
| ICGC_DO46408 | B |
| ICGC_DO46428 | B |
| ICGC_DO46432 | B |
| ICGC_DO46436 | B |
| ICGC_DO46448 | B |
| ICGC_DO46453 | B |
| ICGC_DO46493 | B |
| ICGC_DO46508 | B |
| ICGC_DO46513 | B |
| ICGC_DO46523 | B |
| ICGC_DO46528 | B |
| ICGC_DO46533 | B |
| ICGC_DO46538 | B |
| ICGC_DO46546 | B |
| ICGC_DO46551 | B |
| ICGC_DO46561 | B |
| ICGC_DO46566 | B |
| ICGC_DO46568 | B |
| ICGC_DO46581 | B |
| ICGC_DO46591 | B |
| GSE51088_GSM1238146 | B |
| GSE51088_GSM1238153 | B |
| GSE51088_GSM1238159 | B |
| GSE51088_GSM1238166 | B |
| GSE51088_GSM1238167 | B |
| GSE51088_GSM1238172 | B |
| GSE51088_GSM1238176 | B |
| GSE51088_GSM1238181 | B |
| GSE51088_GSM1238182 | B |
| GSE51088_GSM1238189 | B |
| GSE51088_GSM1238192 | B |
| GSE51088_GSM1238193 | B |
| GSE51088_GSM1238196 | B |
| GSE51088_GSM1238199 | B |
| GSE51088_GSM1238203 | B |
| GSE51088_GSM1238207 | B |
| GSE51088_GSM1238215 | B |
| GSE51088_GSM1238220 | B |
| GSE51088_GSM1238222 | B |
| GSE51088_GSM1238225 | B |
| GSE51088_GSM1238228 | B |
| GSE51088_GSM1238229 | B |
| GSE51088_GSM1238231 | B |
| GSE51088_GSM1238238 | B |
| GSE51088_GSM1238241 | B |
| GSE51088_GSM1238243 | B |
| GSE51088_GSM1238253 | B |
| GSE51088_GSM1238255 | B |
| GSE51088_GSM1238256 | B |
| GSE51088_GSM1238262 | B |
| GSE51088_GSM1238264 | B |
| GSE51088_GSM1238268 | B |
| GSE51088_GSM1238269 | B |
| GSE51088_GSM1238275 | B |
| GSE51088_GSM1238276 | B |
| GSE51088_GSM1238277 | B |
| GSE51088_GSM1238279 | B |
| GSE51088_GSM1238284 | B |
| GSE51088_GSM1238285 | B |
| GSE51088_GSM1238288 | B |
| GSE51088_GSM1238291 | B |
| GSE51088_GSM1238300 | B |
| GSE51088_GSM1238301 | B |
| GSE51088_GSM1238303 | B |
| GSE51088_GSM1238308 | B |
| GSE51088_GSM1238312 | B |
| GSE51088_GSM1238316 | B |
| GSE53963_GSM1304246 | B |
| GSE53963_GSM1304253 | B |
| GSE53963_GSM1304256 | B |
| GSE53963_GSM1304263 | B |
| GSE53963_GSM1304264 | B |
| GSE53963_GSM1304265 | B |
| GSE53963_GSM1304268 | B |
| GSE53963_GSM1304269 | B |
| GSE53963_GSM1304272 | B |
| GSE53963_GSM1304273 | B |
| GSE53963_GSM1304274 | B |
| GSE53963_GSM1304275 | B |
| GSE53963_GSM1304276 | B |
| GSE53963_GSM1304277 | B |
| GSE53963_GSM1304282 | B |
| GSE53963_GSM1304285 | B |
| GSE53963_GSM1304286 | B |
| GSE53963_GSM1304287 | B |
| GSE53963_GSM1304288 | B |
| GSE53963_GSM1304291 | B |
| GSE53963_GSM1304293 | B |
| GSE53963_GSM1304295 | B |
| GSE53963_GSM1304296 | B |
| GSE53963_GSM1304298 | B |
| GSE53963_GSM1304300 | B |
| GSE53963_GSM1304301 | B |
| GSE53963_GSM1304302 | B |
| GSE53963_GSM1304304 | B |
| GSE53963_GSM1304306 | B |
| GSE53963_GSM1304307 | B |
| GSE53963_GSM1304308 | B |
| GSE53963_GSM1304309 | B |
| GSE53963_GSM1304310 | B |
| GSE53963_GSM1304313 | B |
| GSE53963_GSM1304314 | B |
| GSE53963_GSM1304315 | B |
| GSE53963_GSM1304316 | B |
| GSE53963_GSM1304319 | B |
| GSE53963_GSM1304320 | B |
| GSE53963_GSM1304321 | B |
| GSE53963_GSM1304322 | B |
| GSE53963_GSM1304324 | B |
| GSE53963_GSM1304325 | B |
| GSE53963_GSM1304326 | B |
| GSE53963_GSM1304328 | B |
| GSE53963_GSM1304330 | B |
| GSE53963_GSM1304332 | B |
| GSE53963_GSM1304333 | B |
| GSE53963_GSM1304338 | B |
| GSE53963_GSM1304340 | B |
| GSE53963_GSM1304341 | B |
| GSE53963_GSM1304342 | B |
| GSE53963_GSM1304349 | B |
| GSE53963_GSM1304352 | B |
| GSE53963_GSM1304354 | B |
| GSE53963_GSM1304355 | B |
| GSE53963_GSM1304356 | B |
| GSE53963_GSM1304357 | B |
| GSE53963_GSM1304361 | B |
| GSE53963_GSM1304362 | B |
| GSE53963_GSM1304368 | B |
| GSE53963_GSM1304372 | B |
| GSE53963_GSM1304373 | B |
| GSE53963_GSM1304375 | B |
| GSE53963_GSM1304377 | B |
| GSE53963_GSM1304379 | B |
| GSE53963_GSM1304380 | B |
| GSE53963_GSM1304383 | B |
| GSE53963_GSM1304386 | B |
| GSE53963_GSM1304387 | B |
| GSE53963_GSM1304388 | B |
| GSE53963_GSM1304391 | B |
| GSE53963_GSM1304392 | B |
| GSE53963_GSM1304393 | B |
| GSE53963_GSM1304394 | B |
| GSE53963_GSM1304401 | B |
| GSE53963_GSM1304403 | B |
| GSE53963_GSM1304406 | B |
| GSE53963_GSM1304409 | B |
| GSE53963_GSM1304412 | B |
| GSE53963_GSM1304413 | B |
| GSE53963_GSM1304415 | B |
| GSE53963_GSM1304416 | B |
| GSE53963_GSM1304419 | B |
| GSE49997_GSM1211538 | B |
| GSE49997_GSM1211544 | B |
| GSE49997_GSM1211546 | B |
| GSE49997_GSM1211547 | B |
| GSE49997_GSM1211549 | B |
| GSE49997_GSM1211551 | B |
| GSE49997_GSM1211552 | B |
| GSE49997_GSM1211557 | B |
| GSE49997_GSM1211559 | B |
| GSE49997_GSM1211563 | B |
| GSE49997_GSM1211567 | B |
| GSE49997_GSM1211572 | B |
| GSE49997_GSM1211575 | B |
| GSE49997_GSM1211578 | B |
| GSE49997_GSM1211581 | B |
| GSE49997_GSM1211589 | B |
| GSE49997_GSM1211590 | B |
| GSE49997_GSM1211591 | B |
| GSE49997_GSM1211592 | B |
| GSE49997_GSM1211599 | B |
| GSE49997_GSM1211601 | B |
| GSE49997_GSM1211605 | B |
| GSE49997_GSM1211606 | B |
| GSE49997_GSM1211609 | B |
| GSE49997_GSM1211615 | B |
| GSE49997_GSM1211619 | B |
| GSE49997_GSM1211622 | B |
| GSE49997_GSM1211625 | B |
| GSE49997_GSM1211627 | B |
| GSE49997_GSM1211631 | B |
| GSE49997_GSM1211633 | B |
| GSE49997_GSM1211637 | B |
| GSE49997_GSM1211643 | B |
| GSE49997_GSM1211644 | B |
| GSE49997_GSM1211645 | B |
| GSE49997_GSM1211646 | B |
| GSE49997_GSM1211647 | B |
| GSE49997_GSM1211648 | B |
| GSE49997_GSM1211650 | B |
| GSE49997_GSM1211651 | B |
| GSE49997_GSM1211654 | B |
| GSE49997_GSM1211659 | B |
| GSE49997_GSM1211661 | B |
| GSE49997_GSM1211665 | B |
| GSE49997_GSM1211666 | B |
| GSE49997_GSM1211667 | B |
| GSE49997_GSM1211678 | B |
| GSE49997_GSM1211679 | B |
| GSE49997_GSM1211680 | B |
| GSE49997_GSM1211683 | B |
| GSE49997_GSM1211690 | B |
| GSE49997_GSM1211692 | B |
| GSE49997_GSM1211695 | B |
| GSE49997_GSM1211696 | B |
| GSE49997_GSM1211699 | B |
| GSE49997_GSM1211700 | B |
| GSE49997_GSM1211703 | B |
| GSE49997_GSM1211705 | B |
| GSE49997_GSM1211706 | B |
| GSE49997_GSM1211709 | B |
| GSE49997_GSM1211710 | B |
| GSE49997_GSM1211712 | B |
| GSE49997_GSM1211713 | B |
| GSE49997_GSM1211715 | B |
| GSE49997_GSM1211716 | B |
| GSE49997_GSM1211719 | B |
| GSE49997_GSM1211721 | B |
| GSE49997_GSM1211722 | B |
| GSE49997_GSM1211723 | B |
| GSE49997_GSM1211724 | B |
| GSE49997_GSM1211726 | B |
| GSE49997_GSM1211727 | B |
| GSE49997_GSM1211728 | B |
| GSE49997_GSM1211732 | B |
| GSE49997_GSM1211734 | B |
| GSE49997_GSM1211735 | B |
| GSE49997_GSM1211737 | B |
| GSE49997_GSM1211739 | B |
| GSE63885_GSM1559300 | B |
| GSE63885_GSM1559303 | B |
| GSE63885_GSM1559305 | B |
| GSE63885_GSM1559309 | B |
| GSE63885_GSM1559311 | B |
| GSE63885_GSM1559313 | B |
| GSE63885_GSM1559314 | B |
| GSE63885_GSM1559315 | B |
| GSE63885_GSM1559317 | B |
| GSE63885_GSM1559318 | B |
| GSE63885_GSM1559324 | B |
| GSE63885_GSM1559325 | B |
| GSE63885_GSM1559330 | B |
| GSE63885_GSM1559331 | B |
| GSE63885_GSM1559332 | B |
| GSE63885_GSM1559333 | B |
| GSE63885_GSM1559334 | B |
| GSE63885_GSM1559338 | B |
| GSE63885_GSM1559339 | B |
| GSE63885_GSM1559342 | B |
| GSE63885_GSM1559349 | B |
| GSE63885_GSM1559350 | B |
| GSE63885_GSM1559351 | B |
| GSE63885_GSM1559355 | B |
| GSE63885_GSM1559356 | B |
| GSE63885_GSM1559357 | B |
| GSE63885_GSM1559358 | B |
| GSE63885_GSM1559359 | B |
| GSE63885_GSM1559360 | B |
| GSE63885_GSM1559361 | B |
| GSE63885_GSM1559362 | B |
| GSE63885_GSM1559364 | B |
| GSE63885_GSM1559365 | B |
| GSE63885_GSM1559366 | B |
| GSE63885_GSM1559367 | B |
| GSE63885_GSM1559368 | B |
| GSE63885_GSM1559370 | B |

Table-S3:Identification of 4879 DEGs between hypocluster A and B.

| genesymbol | logFC | AveExpr | t | P.Value | adj.P.Val | ID |
| --- | --- | --- | --- | --- | --- | --- |
| ADM | 1.027072876 | 2.938357386 | 19.67379737 | 9.75E-74 | 1.08E-69 | 156.3360004 |
| VEGFA | 0.684338218 | 4.4489437 | 17.35313652 | 1.36E-59 | 7.53E-56 | 124.2726947 |
| LDHA | 0.57993259 | 5.767919361 | 16.94562445 | 3.31E-57 | 1.22E-53 | 118.8644097 |
| PGK1 | 0.496140405 | 5.288530106 | 16.74161969 | 5.04E-56 | 1.39E-52 | 116.1844186 |
| NDRG1 | 0.735730253 | 4.996012327 | 16.29132286 | 1.92E-53 | 4.24E-50 | 110.3359696 |
| SLC2A1 | 0.689711238 | 4.838810206 | 15.69143688 | 4.51E-50 | 8.32E-47 | 102.6937159 |
| CDKN3 | 0.680785213 | 3.247331604 | 14.69559547 | 1.19E-44 | 1.88E-41 | 90.40679561 |
| ALDOA | 0.473263202 | 6.264819712 | 14.57612321 | 5.15E-44 | 7.12E-41 | 88.96790505 |
| BNIP3 | 0.618566748 | 3.983245505 | 13.54415986 | 1.13E-38 | 1.38E-35 | 76.87188125 |
| EGLN3 | 0.753273878 | 3.028615427 | 12.9455805 | 1.06E-35 | 1.17E-32 | 70.14165928 |
| MIF | 0.547304959 | 5.464311343 | 12.92050404 | 1.40E-35 | 1.41E-32 | 69.86448289 |
| SPAG4 | 0.595709298 | 2.895093277 | 12.74824224 | 9.61E-35 | 8.86E-32 | 67.9710095 |
| PDK1 | 0.385201197 | 3.073973434 | 12.45261343 | 2.51E-33 | 2.13E-30 | 64.7650625 |
| ENO1 | 0.392327041 | 6.161782615 | 12.19503651 | 4.11E-32 | 3.24E-29 | 62.01740575 |
| DLGAP5 | 0.527084806 | 3.183739833 | 11.78950706 | 3.07E-30 | 2.26E-27 | 57.77952167 |
| PTTG1 | 0.541349889 | 4.026109598 | 11.5159206 | 5.29E-29 | 3.66E-26 | 54.98257858 |
| P4HA1 | 0.386560968 | 4.18202353 | 11.50309904 | 6.04E-29 | 3.93E-26 | 54.85274668 |
| RNF183 | 0.604742442 | 2.355312284 | 11.46839416 | 8.63E-29 | 5.30E-26 | 54.50188634 |
| PFKFB4 | 0.369252707 | 2.738565382 | 11.33226565 | 3.48E-28 | 2.02E-25 | 53.13362766 |
| STC1 | 0.584876275 | 2.78829057 | 11.27311175 | 6.35E-28 | 3.51E-25 | 52.54304011 |
| TPI1 | 0.339525201 | 6.019183941 | 11.17978753 | 1.63E-27 | 8.59E-25 | 51.61623359 |
| STC2 | 0.494489657 | 2.894701138 | 11.14818024 | 2.24E-27 | 1.13E-24 | 51.30371504 |
| GPI | 0.322548069 | 5.055505586 | 11.00925397 | 9.01E-27 | 4.33E-24 | 49.93836752 |
| KPNA2 | 0.443637664 | 4.692037833 | 10.9069589 | 2.49E-26 | 1.15E-23 | 48.94171196 |
| SCD | 0.559645484 | 4.064089322 | 10.88715724 | 3.03E-26 | 1.34E-23 | 48.74964098 |
| CDC20 | 0.495933408 | 4.229292807 | 10.66027764 | 2.79E-25 | 1.19E-22 | 46.56891321 |
| FTH1 | 0.419450027 | 7.172252648 | 10.56113781 | 7.29E-25 | 2.99E-22 | 45.62759681 |
| HK2 | 0.370597665 | 3.642224213 | 10.54065444 | 8.88E-25 | 3.51E-22 | 45.433996 |
| PFKP | 0.404803734 | 4.361852498 | 10.49187527 | 1.42E-24 | 5.42E-22 | 44.97417896 |
| KIF4A | 0.402700456 | 3.044446389 | 10.48073616 | 1.58E-24 | 5.82E-22 | 44.86941848 |
| BIRC5 | 0.470147536 | 3.634724465 | 10.36724105 | 4.67E-24 | 1.67E-21 | 43.80717913 |
| TK1 | 0.456039745 | 4.085349108 | 10.24729921 | 1.45E-23 | 5.02E-21 | 42.69485175 |
| FAM162A | 0.310113559 | 4.175063114 | 10.18952928 | 2.50E-23 | 8.37E-21 | 42.16287878 |
| KIF20A | 0.439006513 | 3.468647328 | 10.08627694 | 6.54E-23 | 2.13E-20 | 41.21823516 |
| INSIG1 | 0.379080409 | 3.476139867 | 10.05179866 | 9.01E-23 | 2.85E-20 | 40.90456121 |
| MELK | 0.432940432 | 3.35895752 | 9.932502177 | 2.71E-22 | 8.32E-20 | 39.82608162 |
| EBP | 0.388306574 | 3.841009834 | 9.903762377 | 3.53E-22 | 1.05E-19 | 39.56785768 |
| MRPL13 | 0.333379143 | 3.809296353 | 9.871576308 | 4.73E-22 | 1.38E-19 | 39.27940623 |
| DERL1 | 0.295225042 | 4.161380394 | 9.829780007 | 6.93E-22 | 1.96E-19 | 38.90599205 |
| CKS2 | 0.476970938 | 4.875340764 | 9.811537382 | 8.18E-22 | 2.26E-19 | 38.74342282 |
| HMMR | 0.40973479 | 3.024321093 | 9.738163808 | 1.59E-21 | 4.29E-19 | 38.09209514 |
| CEP55 | 0.467598699 | 3.577351381 | 9.722846295 | 1.83E-21 | 4.81E-19 | 37.95663812 |
| DHCR24 | 0.484162099 | 5.580938914 | 9.705003553 | 2.14E-21 | 5.52E-19 | 37.79907435 |
| AURKA | 0.391191466 | 3.432648203 | 9.672104256 | 2.88E-21 | 7.25E-19 | 37.50918504 |
| ATAD2 | 0.374488694 | 3.634319102 | 9.636627986 | 3.96E-21 | 9.74E-19 | 37.19751204 |
| ESM1 | 0.449016237 | 2.517039659 | 9.625684865 | 4.37E-21 | 1.05E-18 | 37.10156599 |
| SPP1 | 0.620498781 | 5.40474087 | 9.595352372 | 5.73E-21 | 1.35E-18 | 36.83609744 |
| ANGPT2 | 0.343482049 | 2.336299162 | 9.591273673 | 5.94E-21 | 1.37E-18 | 36.80045446 |
| SPC25 | 0.431020945 | 2.865386365 | 9.503296995 | 1.30E-20 | 2.93E-18 | 36.03474279 |
| CDCA8 | 0.434861693 | 3.601114552 | 9.464730086 | 1.83E-20 | 4.04E-18 | 35.70094513 |
| BSPRY | 0.374249838 | 3.160784282 | 9.410361481 | 2.95E-20 | 6.40E-18 | 35.23232805 |
| CA9 | 0.714868489 | 2.604946455 | 9.397377881 | 3.30E-20 | 7.03E-18 | 35.12075622 |
| PLOD2 | 0.448000106 | 3.914091992 | 9.387339952 | 3.61E-20 | 7.53E-18 | 35.03458663 |
| MCM4 | 0.323996823 | 3.940491721 | 9.375710971 | 4.00E-20 | 8.19E-18 | 34.93485617 |
| UBE2C | 0.474558404 | 4.715239711 | 9.356244945 | 4.74E-20 | 9.53E-18 | 34.768149 |
| ANLN | 0.36624186 | 3.322213668 | 9.332387153 | 5.84E-20 | 1.15E-17 | 34.56423074 |
| HPRT1 | 0.309303076 | 3.827766753 | 9.265144289 | 1.05E-19 | 2.03E-17 | 33.99186516 |
| CYC1 | 0.388999452 | 5.370028712 | 9.228684992 | 1.44E-19 | 2.74E-17 | 33.682996 |
| ANGPTL4 | 0.532560095 | 2.609931859 | 9.197239359 | 1.88E-19 | 3.53E-17 | 33.41743239 |
| STXBP6 | 0.573446313 | 2.910746178 | 9.192015235 | 1.97E-19 | 3.63E-17 | 33.37338852 |
| GPSM2 | 0.295634795 | 2.818564486 | 9.167146625 | 2.44E-19 | 4.43E-17 | 33.1640168 |
| HCCS | 0.274459849 | 3.301916509 | 9.163419697 | 2.52E-19 | 4.50E-17 | 33.13268099 |
| NCAPG | 0.37129835 | 2.930290096 | 9.1298972 | 3.36E-19 | 5.90E-17 | 32.85131396 |
| TFRC | 0.393725366 | 4.218304729 | 9.100245993 | 4.33E-19 | 7.48E-17 | 32.6031735 |
| TTK | 0.403803046 | 3.157218715 | 9.041876481 | 7.12E-19 | 1.21E-16 | 32.1167153 |
| ECT2 | 0.365516453 | 3.873018192 | 9.002667117 | 9.93E-19 | 1.66E-16 | 31.79144419 |
| CREBL2 | -0.251691778 | 3.574433588 | -8.99978267 | 1.02E-18 | 1.68E-16 | 31.76756332 |
| FAM83D | 0.429862188 | 3.521088288 | 8.960963472 | 1.41E-18 | 2.30E-16 | 31.44681092 |
| CIRBP | -0.249213298 | 4.203083835 | -8.9398393 | 1.69E-18 | 2.70E-16 | 31.27276756 |
| SEC61G | 0.282669765 | 4.363475637 | 8.920414309 | 1.99E-18 | 3.14E-16 | 31.11303519 |
| DDIT4 | 0.474019636 | 4.198194009 | 8.903531776 | 2.29E-18 | 3.57E-16 | 30.97445209 |
| ELF3 | 0.44212346 | 4.860570669 | 8.875169944 | 2.90E-18 | 4.46E-16 | 30.74214752 |
| FEN1 | 0.302090787 | 3.623446246 | 8.867465833 | 3.10E-18 | 4.69E-16 | 30.67915527 |
| BUB1 | 0.351962294 | 3.221253677 | 8.826826462 | 4.35E-18 | 6.50E-16 | 30.34764946 |
| UPP1 | 0.349581272 | 2.754525808 | 8.801645178 | 5.36E-18 | 7.90E-16 | 30.14289798 |
| COL4A6 | -0.33398622 | 1.720420133 | -8.70677838 | 1.17E-17 | 1.71E-15 | 29.37606501 |
| NSDHL | 0.28178652 | 3.524386484 | 8.699876361 | 1.24E-17 | 1.77E-15 | 29.32055468 |
| CCNA2 | 0.396621995 | 3.458302537 | 8.699174907 | 1.25E-17 | 1.77E-15 | 29.3149153 |
| GPRASP1 | -0.236348255 | 2.178736975 | -8.6662063 | 1.64E-17 | 2.29E-15 | 29.0503058 |
| CCNB1 | 0.354827128 | 4.081956262 | 8.659638817 | 1.73E-17 | 2.39E-15 | 28.99769852 |
| NDUFA8 | 0.262190725 | 4.403568871 | 8.591298294 | 3.02E-17 | 4.13E-15 | 28.45232641 |
| FAM91A1 | 0.248662416 | 3.796034474 | 8.571071282 | 3.56E-17 | 4.81E-15 | 28.29163035 |
| CDCA5 | 0.344676089 | 3.473412592 | 8.56410326 | 3.77E-17 | 5.02E-15 | 28.23634827 |
| PRC1 | 0.336812634 | 3.623686565 | 8.559218402 | 3.92E-17 | 5.16E-15 | 28.19761667 |
| RRM2 | 0.380983189 | 3.524558735 | 8.542626435 | 4.49E-17 | 5.84E-15 | 28.066204 |
| MTCH2 | 0.220995108 | 4.35300176 | 8.48516616 | 7.14E-17 | 9.18E-15 | 27.61282 |
| SMS | 0.277941025 | 4.531606049 | 8.479117076 | 7.49E-17 | 9.44E-15 | 27.56524552 |
| CENPA | 0.380126562 | 3.1070756 | 8.478817952 | 7.51E-17 | 9.44E-15 | 27.56289375 |
| COX7B | 0.318291447 | 4.563617934 | 8.472160065 | 7.93E-17 | 9.85E-15 | 27.510567 |
| BACH2 | -0.245258124 | 1.912608495 | -8.46907872 | 8.12E-17 | 9.98E-15 | 27.48636177 |
| SMC4 | 0.290113828 | 3.866841185 | 8.419593282 | 1.21E-16 | 1.47E-14 | 27.09868561 |
| NCAPG2 | 0.270948534 | 3.238553893 | 8.41286342 | 1.28E-16 | 1.53E-14 | 27.04611618 |
| TRIP13 | 0.328906341 | 3.483506033 | 8.410228673 | 1.30E-16 | 1.55E-14 | 27.0255452 |
| PBK | 0.42134646 | 3.068741803 | 8.405912805 | 1.35E-16 | 1.59E-14 | 26.99186093 |
| SDHA | 0.242757643 | 4.171558513 | 8.401432391 | 1.40E-16 | 1.63E-14 | 26.9569084 |
| GYS1 | 0.238686058 | 4.019316144 | 8.39687085 | 1.45E-16 | 1.67E-14 | 26.92133972 |
| VDAC1 | 0.213150851 | 4.717621491 | 8.39249201 | 1.50E-16 | 1.71E-14 | 26.88721154 |
| TCF4 | -0.269392577 | 3.130817174 | -8.38306436 | 1.62E-16 | 1.83E-14 | 26.81378633 |
| MRPL36 | 0.308722054 | 3.816900786 | 8.373914905 | 1.74E-16 | 1.94E-14 | 26.74259688 |
| ASF1B | 0.399040315 | 3.825856941 | 8.368588719 | 1.82E-16 | 2.01E-14 | 26.70118656 |
| KIF23 | 0.322795718 | 3.109356126 | 8.34581008 | 2.18E-16 | 2.38E-14 | 26.52434623 |
| CDK2AP2 | 0.28529987 | 4.6041077 | 8.339453138 | 2.29E-16 | 2.48E-14 | 26.4750699 |
| PGM2L1 | 0.335611302 | 2.897094776 | 8.337838253 | 2.32E-16 | 2.49E-14 | 26.46255724 |
| CCNB2 | 0.349661338 | 3.533632833 | 8.326350219 | 2.54E-16 | 2.70E-14 | 26.37360539 |
| ARPC5L | 0.245825188 | 3.833151013 | 8.316117225 | 2.75E-16 | 2.90E-14 | 26.29446183 |
| MRPS15 | 0.271977928 | 4.261600702 | 8.284598672 | 3.53E-16 | 3.68E-14 | 26.05122878 |
| PAPLN | -0.342555213 | 2.603297841 | -8.28358569 | 3.56E-16 | 3.68E-14 | 26.0434249 |
| LTBP4 | -0.459121165 | 3.764007312 | -8.25987264 | 4.29E-16 | 4.40E-14 | 25.86098195 |
| C6orf223 | 0.40409244 | 2.311350544 | 8.248943297 | 4.68E-16 | 4.75E-14 | 25.7770486 |
| DISP1 | -0.207414764 | 2.493959943 | -8.23111767 | 5.38E-16 | 5.41E-14 | 25.64036387 |
| RANBP1 | 0.259223025 | 4.152183997 | 8.223941742 | 5.69E-16 | 5.64E-14 | 25.58541309 |
| MMP1 | 0.654923064 | 1.772125783 | 8.223611634 | 5.71E-16 | 5.64E-14 | 25.58288625 |
| SLIT3 | -0.395254115 | 2.907244286 | -8.20423519 | 6.64E-16 | 6.50E-14 | 25.43472397 |
| FRY | -0.224117073 | 2.369465813 | -8.20221203 | 6.75E-16 | 6.50E-14 | 25.4192715 |
| TMEM165 | 0.244883624 | 3.993593641 | 8.202119463 | 6.76E-16 | 6.50E-14 | 25.41856461 |
| RCBTB2 | -0.252210939 | 2.861193846 | -8.18936033 | 7.46E-16 | 7.12E-14 | 25.3211917 |
| PLK1 | 0.310073108 | 3.50126539 | 8.17945032 | 8.06E-16 | 7.62E-14 | 25.24565429 |
| NDC80 | 0.348105765 | 2.972582252 | 8.170402383 | 8.65E-16 | 8.11E-14 | 25.1767582 |
| NUSAP1 | 0.349164815 | 3.923081447 | 8.162845784 | 9.18E-16 | 8.53E-14 | 25.11926948 |
| DTX3 | -0.278332373 | 3.462613769 | -8.13310806 | 1.16E-15 | 1.07E-13 | 24.89348746 |
| FMOD | -0.457744377 | 4.31217185 | -8.12410662 | 1.24E-15 | 1.13E-13 | 24.82528779 |
| ENO2 | 0.399202798 | 3.727590272 | 8.096269804 | 1.54E-15 | 1.40E-13 | 24.61480285 |
| ABCA6 | -0.232390889 | 1.8702527 | -8.07688285 | 1.79E-15 | 1.61E-13 | 24.46858736 |
| TRAF5 | -0.246171949 | 2.848699288 | -8.06632845 | 1.94E-15 | 1.73E-13 | 24.38911674 |
| COL4A5 | -0.345015052 | 2.97791744 | -8.05587645 | 2.10E-15 | 1.86E-13 | 24.31050758 |
| YWHAZ | 0.244079834 | 5.68553359 | 8.025804216 | 2.65E-15 | 2.33E-13 | 24.08483801 |
| KIF18A | 0.299106945 | 2.647502041 | 8.003573676 | 3.14E-15 | 2.74E-13 | 23.91849446 |
| C7 | -0.722715207 | 2.958958919 | -7.99384267 | 3.39E-15 | 2.93E-13 | 23.8458092 |
| MFAP4 | -0.610607984 | 3.386333474 | -7.98236371 | 3.70E-15 | 3.17E-13 | 23.76016831 |
| MRPL47 | 0.259914399 | 4.215643716 | 7.976965962 | 3.85E-15 | 3.28E-13 | 23.71993511 |
| KRT19 | 0.404693078 | 5.76333201 | 7.972958255 | 3.97E-15 | 3.36E-13 | 23.69007845 |
| ST3GAL5 | -0.236081235 | 2.584298698 | -7.9547063 | 4.57E-15 | 3.83E-13 | 23.55427301 |
| CDC6 | 0.285874309 | 2.642286993 | 7.949143899 | 4.77E-15 | 3.96E-13 | 23.51294033 |
| RACGAP1 | 0.285670465 | 3.672266888 | 7.941294033 | 5.06E-15 | 4.18E-13 | 23.45465368 |
| MRPS17 | 0.239338118 | 2.979433208 | 7.932336074 | 5.42E-15 | 4.42E-13 | 23.38820166 |
| CENPI | 0.227031324 | 2.229634518 | 7.931758212 | 5.44E-15 | 4.42E-13 | 23.38391724 |
| POLR2K | 0.2683169 | 4.508865194 | 7.909649446 | 6.43E-15 | 5.19E-13 | 23.22020518 |
| ARHGAP25 | -0.219849901 | 2.643927662 | -7.90159383 | 6.84E-15 | 5.48E-13 | 23.16065542 |
| CDKN2A | 0.69299406 | 4.329039746 | 7.89051967 | 7.44E-15 | 5.92E-13 | 23.07887949 |
| CPT2 | 0.228754011 | 3.252353543 | 7.834472327 | 1.14E-14 | 8.97E-13 | 22.66656776 |
| HIGD1A | 0.251127113 | 4.56285988 | 7.822805676 | 1.24E-14 | 9.72E-13 | 22.5810709 |
| CDC25C | 0.29292686 | 2.56562179 | 7.800609722 | 1.46E-14 | 1.14E-12 | 22.41872521 |
| ZNF521 | -0.412208227 | 2.591883299 | -7.78507913 | 1.64E-14 | 1.27E-12 | 22.30537567 |
| CDCP1 | 0.306921845 | 3.408698489 | 7.783134162 | 1.67E-14 | 1.28E-12 | 22.29119453 |
| ARL6IP1 | 0.266076676 | 4.967157594 | 7.755418859 | 2.05E-14 | 1.57E-12 | 22.08946049 |
| PGAM1 | 0.246102795 | 4.339989411 | 7.749736248 | 2.14E-14 | 1.62E-12 | 22.0481772 |
| CENPE | 0.245126684 | 2.522902997 | 7.745026965 | 2.22E-14 | 1.66E-12 | 22.01398544 |
| COX8A | 0.259449726 | 5.691676514 | 7.744971867 | 2.22E-14 | 1.66E-12 | 22.01358551 |
| WDYHV1 | 0.237057795 | 3.32869759 | 7.739473738 | 2.31E-14 | 1.72E-12 | 21.97369005 |
| MBTPS1 | -0.166247846 | 3.913351167 | -7.73421844 | 2.40E-14 | 1.77E-12 | 21.93558027 |
| AURKB | 0.339237287 | 3.261561065 | 7.727928142 | 2.52E-14 | 1.85E-12 | 21.88999532 |
| NCAPH | 0.292418507 | 3.077016989 | 7.721783422 | 2.64E-14 | 1.92E-12 | 21.84549734 |
| APOO | 0.271209748 | 3.531058049 | 7.7197791 | 2.68E-14 | 1.93E-12 | 21.83098955 |
| TUB | -0.266135191 | 2.452176524 | -7.71864103 | 2.70E-14 | 1.94E-12 | 21.82275344 |
| NUF2 | 0.3317606 | 3.061632001 | 7.716587485 | 2.74E-14 | 1.96E-12 | 21.80789477 |
| ABCA8 | -0.291976471 | 1.710889176 | -7.70657489 | 2.95E-14 | 2.09E-12 | 21.7354982 |
| SLC25A5 | 0.246587727 | 5.584383611 | 7.705058035 | 2.99E-14 | 2.10E-12 | 21.72453784 |
| BCL10 | 0.205987035 | 3.267277727 | 7.703409005 | 3.02E-14 | 2.12E-12 | 21.71262459 |
| NDUFS6 | 0.263108963 | 4.638332758 | 7.693892743 | 3.24E-14 | 2.26E-12 | 21.64391984 |
| SH3D19 | -0.244734369 | 3.280252841 | -7.68678872 | 3.42E-14 | 2.36E-12 | 21.59268023 |
| VRK1 | 0.241822543 | 3.274976907 | 7.67412659 | 3.75E-14 | 2.58E-12 | 21.50145628 |
| GAB1 | -0.17762138 | 2.574996078 | -7.62851468 | 5.25E-14 | 3.59E-12 | 21.17396158 |
| RECQL4 | 0.33928096 | 3.499401773 | 7.626863007 | 5.32E-14 | 3.61E-12 | 21.16213534 |
| HLF | -0.311094969 | 1.439441065 | -7.62158814 | 5.53E-14 | 3.73E-12 | 21.12438169 |
| ACAT2 | 0.250823086 | 2.89861533 | 7.618382815 | 5.66E-14 | 3.79E-12 | 21.10145174 |
| CDCA2 | 0.274600563 | 2.558754213 | 7.615533958 | 5.78E-14 | 3.85E-12 | 21.08107911 |
| S100A2 | 0.590584433 | 3.437914174 | 7.613739061 | 5.86E-14 | 3.88E-12 | 21.06824701 |
| SHCBP1 | 0.264664173 | 2.438948961 | 7.584974941 | 7.23E-14 | 4.76E-12 | 20.862976 |
| ASNS | 0.261505537 | 3.420360811 | 7.57712839 | 7.66E-14 | 5.01E-12 | 20.8071011 |
| DBI | 0.245035763 | 4.385629342 | 7.574547549 | 7.80E-14 | 5.08E-12 | 20.78873439 |
| TREM1 | 0.332247056 | 2.028885264 | 7.572647105 | 7.91E-14 | 5.09E-12 | 20.77521335 |
| TMEM98 | -0.331279482 | 3.74527199 | -7.57254128 | 7.92E-14 | 5.09E-12 | 20.77446055 |
| RAD51 | 0.273446555 | 2.695901521 | 7.569573379 | 8.09E-14 | 5.17E-12 | 20.75335124 |
| SFN | 0.496276572 | 3.599447039 | 7.568688187 | 8.15E-14 | 5.18E-12 | 20.74705672 |
| TOP2A | 0.342698207 | 3.905656773 | 7.564928651 | 8.37E-14 | 5.27E-12 | 20.72033035 |
| SLC31A1 | 0.228866413 | 3.254739862 | 7.564615893 | 8.39E-14 | 5.27E-12 | 20.7181075 |
| ERCC6L | 0.268663701 | 2.237939346 | 7.562159883 | 8.54E-14 | 5.31E-12 | 20.70065492 |
| ZNF25 | -0.183534696 | 2.651480881 | -7.56211261 | 8.55E-14 | 5.31E-12 | 20.70031904 |
| CROCC | -0.231041172 | 3.072187183 | -7.56082008 | 8.63E-14 | 5.31E-12 | 20.69113633 |
| EGLN1 | 0.200711077 | 3.5328631 | 7.56061672 | 8.64E-14 | 5.31E-12 | 20.68969169 |
| MAL2 | 0.345739163 | 5.46203518 | 7.557772306 | 8.82E-14 | 5.39E-12 | 20.66948913 |
| BAZ2B | -0.211899961 | 3.260219915 | -7.5414421 | 9.94E-14 | 6.04E-12 | 20.55363521 |
| TPX2 | 0.347779367 | 4.359107619 | 7.534662928 | 1.04E-13 | 6.31E-12 | 20.50560672 |
| SESN1 | -0.201913936 | 2.810661524 | -7.52870671 | 1.09E-13 | 6.54E-12 | 20.46344061 |
| C8orf76 | 0.237160769 | 2.889926446 | 7.527795174 | 1.10E-13 | 6.54E-12 | 20.4569902 |
| SSBP1 | 0.195735404 | 4.119395141 | 7.527398926 | 1.10E-13 | 6.54E-12 | 20.45418639 |
| BOC | -0.373662736 | 3.456796327 | -7.52540747 | 1.12E-13 | 6.60E-12 | 20.44009705 |
| CXCL12 | -0.493308992 | 3.494671449 | -7.52440968 | 1.12E-13 | 6.62E-12 | 20.43303907 |
| FHL1 | -0.404353378 | 3.057608027 | -7.52213232 | 1.14E-13 | 6.69E-12 | 20.41693303 |
| RPN1 | 0.194659586 | 5.541359166 | 7.515527053 | 1.20E-13 | 6.98E-12 | 20.37024378 |
| CENPM | 0.314877815 | 2.898494865 | 7.509679243 | 1.25E-13 | 7.25E-12 | 20.32893934 |
| UBE2S | 0.315838045 | 4.033631053 | 7.48497296 | 1.50E-13 | 8.62E-12 | 20.15475196 |
| APOL1 | 0.449265445 | 4.387964075 | 7.477654569 | 1.58E-13 | 9.04E-12 | 20.10325396 |
| BDH2 | -0.225718922 | 3.059822535 | -7.47260057 | 1.64E-13 | 9.33E-12 | 20.06771644 |
| SQLE | 0.304886079 | 3.879259018 | 7.466520347 | 1.71E-13 | 9.70E-12 | 20.02499163 |
| CCND2 | -0.462954905 | 3.103108205 | -7.46299394 | 1.75E-13 | 9.90E-12 | 20.00022645 |
| NR2F1 | -0.387763863 | 2.811797942 | -7.44621523 | 1.98E-13 | 1.11E-11 | 19.88253724 |
| MRPL37 | 0.215652034 | 4.501150128 | 7.440658156 | 2.06E-13 | 1.15E-11 | 19.8436114 |
| TUBA4A | 0.372327787 | 3.580365701 | 7.439855786 | 2.07E-13 | 1.15E-11 | 19.83799317 |
| PRDX4 | 0.241223037 | 4.613809926 | 7.4331827 | 2.17E-13 | 1.20E-11 | 19.79128904 |
| BOLA3 | 0.240203692 | 3.379488969 | 7.428928142 | 2.24E-13 | 1.23E-11 | 19.76153159 |
| EXO1 | 0.274201006 | 2.682504273 | 7.418148028 | 2.42E-13 | 1.32E-11 | 19.68620144 |
| NEK2 | 0.333701369 | 3.198800063 | 7.418115996 | 2.42E-13 | 1.32E-11 | 19.68597775 |
| GLI2 | -0.308488419 | 2.346237317 | -7.41356209 | 2.50E-13 | 1.36E-11 | 19.65418532 |
| CHST10 | -0.236496478 | 2.852320973 | -7.40988264 | 2.57E-13 | 1.39E-11 | 19.62851066 |
| EXOSC4 | 0.286368865 | 4.109376805 | 7.407553557 | 2.61E-13 | 1.40E-11 | 19.61226454 |
| MAN1C1 | -0.233358758 | 2.343390352 | -7.40754711 | 2.61E-13 | 1.40E-11 | 19.61221959 |
| DNAJA1 | 0.220377379 | 5.160257326 | 7.396566147 | 2.83E-13 | 1.50E-11 | 19.53568594 |
| PHLDA2 | 0.436826608 | 3.142904063 | 7.393413638 | 2.89E-13 | 1.53E-11 | 19.51373292 |
| SNTB1 | 0.31827792 | 3.189999566 | 7.384994102 | 3.07E-13 | 1.62E-11 | 19.45514341 |
| KIAA1324L | -0.319169918 | 2.443163815 | -7.37692316 | 3.25E-13 | 1.71E-11 | 19.39903614 |
| PDHA1 | 0.228528118 | 4.215180622 | 7.372972558 | 3.35E-13 | 1.75E-11 | 19.37159271 |
| HJURP | 0.296925056 | 3.298270827 | 7.370057361 | 3.42E-13 | 1.77E-11 | 19.35135035 |
| BNC2 | -0.27210497 | 2.702765911 | -7.36840615 | 3.46E-13 | 1.79E-11 | 19.33988794 |
| FAF1 | 0.176790284 | 3.607827219 | 7.362147727 | 3.62E-13 | 1.86E-11 | 19.2964643 |
| ARMCX1 | -0.27374583 | 2.93812877 | -7.36130078 | 3.64E-13 | 1.86E-11 | 19.29059034 |
| PKIG | -0.236464519 | 3.771599459 | -7.33572364 | 4.36E-13 | 2.22E-11 | 19.11348948 |
| S100A6 | 0.432705324 | 6.267734245 | 7.317735362 | 4.96E-13 | 2.52E-11 | 18.98926855 |
| CEP290 | -0.168568554 | 2.575781709 | -7.31515314 | 5.05E-13 | 2.55E-11 | 18.9714592 |
| C15orf48 | 0.511601909 | 2.731625129 | 7.299587257 | 5.64E-13 | 2.84E-11 | 18.86422309 |
| ZNF219 | -0.317621053 | 3.343959091 | -7.29560106 | 5.80E-13 | 2.90E-11 | 18.83679467 |
| TSTA3 | 0.278482803 | 4.534363319 | 7.293753253 | 5.88E-13 | 2.92E-11 | 18.82408478 |
| ECE2 | 0.276432916 | 2.766106662 | 7.29346641 | 5.89E-13 | 2.92E-11 | 18.82211203 |
| MYBL2 | 0.381341675 | 4.066071746 | 7.284189642 | 6.29E-13 | 3.11E-11 | 18.75834929 |
| S100A16 | 0.351639176 | 5.16081 | 7.271934875 | 6.86E-13 | 3.37E-11 | 18.67423011 |
| NEK1 | -0.151883598 | 2.389457852 | -7.26644655 | 7.13E-13 | 3.49E-11 | 18.63659867 |
| SST | 0.991727232 | 3.406550459 | 7.253831231 | 7.79E-13 | 3.80E-11 | 18.55019747 |
| OIP5 | 0.309893571 | 2.459725171 | 7.248969243 | 8.06E-13 | 3.91E-11 | 18.51693441 |
| PAICS | 0.224780063 | 4.092207125 | 7.239769267 | 8.60E-13 | 4.15E-11 | 18.45404844 |
| SFXN1 | 0.182252312 | 3.099939432 | 7.234273936 | 8.94E-13 | 4.30E-11 | 18.41651986 |
| GCLM | 0.240645975 | 3.064268496 | 7.232207781 | 9.07E-13 | 4.32E-11 | 18.4024164 |
| EZH2 | 0.273474152 | 3.391832642 | 7.23218512 | 9.07E-13 | 4.32E-11 | 18.40226174 |
| ZDHHC17 | -0.164936741 | 2.894480346 | -7.21157657 | 1.05E-12 | 4.97E-11 | 18.26178899 |
| PBXIP1 | -0.223726362 | 4.297149544 | -7.19148811 | 1.21E-12 | 5.70E-11 | 18.12521074 |
| PDCD10 | 0.222375931 | 4.197231355 | 7.174486085 | 1.36E-12 | 6.39E-11 | 18.00988654 |
| CDO1 | -0.383220528 | 1.485913168 | -7.16603077 | 1.44E-12 | 6.75E-11 | 17.95262652 |
| LACTB2 | 0.280668327 | 3.66475368 | 7.15996834 | 1.50E-12 | 7.01E-11 | 17.91160903 |
| FOXP1 | -0.171582848 | 3.113317511 | -7.15688584 | 1.54E-12 | 7.13E-11 | 17.89076536 |
| GBE1 | 0.188176842 | 3.167334538 | 7.148411684 | 1.63E-12 | 7.54E-11 | 17.83350563 |
| CX3CR1 | -0.420735761 | 2.322986254 | -7.14147003 | 1.71E-12 | 7.87E-11 | 17.78664687 |
| NSMCE2 | 0.210903764 | 3.533738854 | 7.14033931 | 1.72E-12 | 7.90E-11 | 17.77901798 |
| UQCRC1 | 0.205599751 | 4.940789238 | 7.139304571 | 1.73E-12 | 7.93E-11 | 17.77203764 |
| HTR3A | 0.67005212 | 2.673995665 | 7.135725483 | 1.78E-12 | 8.09E-11 | 17.74790025 |
| MCM10 | 0.277525582 | 2.593945237 | 7.130763169 | 1.84E-12 | 8.34E-11 | 17.71445253 |
| ZWINT | 0.286797582 | 3.832293042 | 7.122738044 | 1.95E-12 | 8.78E-11 | 17.66040512 |
| ZFPM2 | -0.304111042 | 2.597474321 | -7.09928209 | 2.29E-12 | 1.03E-10 | 17.50275143 |
| PHC1 | -0.249533834 | 3.207618488 | -7.09788655 | 2.31E-12 | 1.03E-10 | 17.4933865 |
| AK2 | 0.195383419 | 4.49276132 | 7.079943523 | 2.62E-12 | 1.17E-10 | 17.37312718 |
| SLCO4A1 | 0.334632598 | 1.966904321 | 7.078322871 | 2.64E-12 | 1.17E-10 | 17.36227873 |
| NAP1L1 | -0.221647867 | 4.773745765 | -7.07505021 | 2.70E-12 | 1.20E-10 | 17.34037879 |
| UBE2T | 0.310168961 | 3.766013197 | 7.068286524 | 2.83E-12 | 1.25E-10 | 17.29514689 |
| STOML2 | 0.199236698 | 4.74438199 | 7.067045651 | 2.86E-12 | 1.25E-10 | 17.28685287 |
| CRABP2 | 0.463824828 | 6.043694872 | 7.065628802 | 2.89E-12 | 1.26E-10 | 17.27738424 |
| ATP6V0B | 0.249339507 | 4.6304588 | 7.058300875 | 3.04E-12 | 1.32E-10 | 17.22844014 |
| KRT6A | 0.649116434 | 2.630157921 | 7.05663604 | 3.07E-12 | 1.33E-10 | 17.21732695 |
| WNT4 | -0.352526985 | 2.044825316 | -7.04204448 | 3.39E-12 | 1.47E-10 | 17.1200267 |
| E2F8 | 0.28946238 | 2.778607728 | 7.037913966 | 3.49E-12 | 1.50E-10 | 17.09251664 |
| OGN | -0.475625185 | 1.785851055 | -7.03712137 | 3.51E-12 | 1.50E-10 | 17.08723947 |
| GMPS | 0.188689759 | 3.943313897 | 7.01724051 | 4.02E-12 | 1.72E-10 | 16.95504812 |
| ARHGAP11A | 0.260780491 | 2.877304856 | 7.016286443 | 4.05E-12 | 1.72E-10 | 16.94871292 |
| THRA | -0.211517219 | 3.34622573 | -7.01030202 | 4.22E-12 | 1.78E-10 | 16.90899303 |
| ELN | -0.401716572 | 3.267203052 | -7.00988473 | 4.23E-12 | 1.78E-10 | 16.90622454 |
| SNRPD3 | 0.192186844 | 4.436841208 | 7.009443417 | 4.24E-12 | 1.78E-10 | 16.90329684 |
| TK2 | -0.171360172 | 2.866447278 | -7.00919324 | 4.25E-12 | 1.78E-10 | 16.9016372 |
| RGS12 | -0.172209213 | 2.851439541 | -7.00381483 | 4.41E-12 | 1.84E-10 | 16.86597108 |
| ISCU | -0.157977347 | 4.214697827 | -7.00219246 | 4.46E-12 | 1.85E-10 | 16.85521741 |
| CDKN2D | 0.320253404 | 2.909448169 | 6.99207967 | 4.78E-12 | 1.98E-10 | 16.78823756 |
| APPL2 | -0.190187934 | 3.387224979 | -6.97820333 | 5.25E-12 | 2.17E-10 | 16.69647422 |
| SDF2L1 | 0.285756045 | 3.621485206 | 6.977132018 | 5.29E-12 | 2.17E-10 | 16.68939662 |
| ASPM | 0.266495161 | 3.011750679 | 6.973557873 | 5.42E-12 | 2.22E-10 | 16.66579125 |
| PIAS2 | 0.171911777 | 2.916566114 | 6.959998161 | 5.94E-12 | 2.42E-10 | 16.57633667 |
| COX6C | 0.277736748 | 5.006239324 | 6.957519308 | 6.04E-12 | 2.46E-10 | 16.56000063 |
| HMGB3 | 0.268428175 | 3.928353519 | 6.955819942 | 6.11E-12 | 2.48E-10 | 16.54880461 |
| PSMA3 | 0.165245784 | 3.990625947 | 6.948123408 | 6.44E-12 | 2.60E-10 | 16.49812834 |
| PPA1 | 0.243595088 | 4.883829349 | 6.9468239 | 6.50E-12 | 2.61E-10 | 16.48957704 |
| LRP8 | 0.289942566 | 2.962502588 | 6.946436322 | 6.51E-12 | 2.61E-10 | 16.4870269 |
| IL13RA1 | 0.24305694 | 4.223533423 | 6.935110715 | 7.03E-12 | 2.81E-10 | 16.41256533 |
| PSMD10 | 0.204542209 | 3.896152652 | 6.931651106 | 7.20E-12 | 2.86E-10 | 16.38984182 |
| PLS1 | 0.269782309 | 2.97178372 | 6.925431631 | 7.51E-12 | 2.98E-10 | 16.3490169 |
| RDH11 | 0.198618935 | 3.526833192 | 6.924442728 | 7.56E-12 | 2.99E-10 | 16.34252878 |
| SOD2 | 0.334379774 | 4.016835436 | 6.91946694 | 7.82E-12 | 3.08E-10 | 16.30989582 |
| TMEM119 | -0.357746915 | 2.577951294 | -6.91720132 | 7.94E-12 | 3.11E-10 | 16.29504421 |
| KLF2 | -0.345586207 | 3.210055015 | -6.91287205 | 8.17E-12 | 3.19E-10 | 16.2666772 |
| VBP1 | 0.212566439 | 3.890822527 | 6.902845349 | 8.74E-12 | 3.41E-10 | 16.2010409 |
| CA12 | 0.425227294 | 2.714246036 | 6.895095364 | 9.21E-12 | 3.58E-10 | 16.15036793 |
| GAPDH | 0.284940229 | 7.42809883 | 6.891338179 | 9.45E-12 | 3.65E-10 | 16.12582044 |
| KLHDC1 | -0.177483 | 1.896870962 | -6.88628441 | 9.78E-12 | 3.77E-10 | 16.09282103 |
| ALG3 | 0.205597576 | 4.404493192 | 6.883342147 | 9.97E-12 | 3.83E-10 | 16.07361923 |
| NDUFB6 | 0.23138108 | 3.820251012 | 6.881567796 | 1.01E-11 | 3.86E-10 | 16.06204309 |
| PSMB2 | 0.193775972 | 4.332484441 | 6.87839587 | 1.03E-11 | 3.93E-10 | 16.04135574 |
| EEF2 | -0.19658005 | 6.611468167 | -6.87394503 | 1.06E-11 | 4.04E-10 | 16.01234202 |
| APBB2 | -0.204947619 | 3.064638692 | -6.87299319 | 1.07E-11 | 4.05E-10 | 16.00613943 |
| CORO2A | 0.265011044 | 3.160039894 | 6.871997398 | 1.08E-11 | 4.06E-10 | 15.99965133 |
| CENPN | 0.237193321 | 2.666701498 | 6.870713723 | 1.09E-11 | 4.08E-10 | 15.99128877 |
| RMI1 | 0.240709407 | 2.982775595 | 6.869475343 | 1.09E-11 | 4.10E-10 | 15.98322265 |
| PSRC1 | 0.271214733 | 3.334866297 | 6.867587479 | 1.11E-11 | 4.14E-10 | 15.97092871 |
| PALM | -0.355085156 | 3.410845748 | -6.85888215 | 1.17E-11 | 4.38E-10 | 15.91427876 |
| SLC6A8 | 0.380225807 | 3.585360768 | 6.84758816 | 1.27E-11 | 4.70E-10 | 15.84088102 |
| CDCA7 | 0.361375759 | 2.874590963 | 6.846814583 | 1.27E-11 | 4.71E-10 | 15.83585773 |
| CRY2 | -0.167615178 | 3.037800519 | -6.84360927 | 1.30E-11 | 4.80E-10 | 15.81504923 |
| TBC1D4 | -0.195716102 | 2.753852202 | -6.81238272 | 1.60E-11 | 5.89E-10 | 15.61279708 |
| SYNE1 | -0.152891592 | 2.537477293 | -6.81165891 | 1.61E-11 | 5.90E-10 | 15.60811903 |
| MND1 | 0.311097111 | 2.541635831 | 6.805283356 | 1.68E-11 | 6.14E-10 | 15.56693309 |
| LSM12 | 0.171222499 | 3.213249793 | 6.799410619 | 1.75E-11 | 6.36E-10 | 15.5290266 |
| PGM1 | 0.231564602 | 3.978251773 | 6.787954824 | 1.89E-11 | 6.84E-10 | 15.45516966 |
| BUB1B | 0.263419268 | 3.189854578 | 6.78046051 | 1.98E-11 | 7.17E-10 | 15.40691462 |
| NDUFS3 | 0.192014387 | 4.222506537 | 6.778477035 | 2.01E-11 | 7.22E-10 | 15.39415142 |
| ABCA9 | -0.210652743 | 1.735002922 | -6.7784248 | 2.01E-11 | 7.22E-10 | 15.39381535 |
| GTSE1 | 0.254962871 | 2.749469627 | 6.777944971 | 2.02E-11 | 7.22E-10 | 15.3907283 |
| TPD52 | 0.20953041 | 3.870140879 | 6.774937435 | 2.06E-11 | 7.34E-10 | 15.37138342 |
| RAB6A | 0.190107634 | 4.31888985 | 6.77269573 | 2.09E-11 | 7.42E-10 | 15.3569696 |
| CDCA4 | 0.232119739 | 3.720372661 | 6.771398534 | 2.11E-11 | 7.46E-10 | 15.34863082 |
| DPEP2 | -0.208030007 | 1.815040173 | -6.77025993 | 2.12E-11 | 7.50E-10 | 15.3413127 |
| CHCHD3 | 0.159463021 | 4.033209854 | 6.767471363 | 2.16E-11 | 7.61E-10 | 15.32339467 |
| IFT140 | -0.199818943 | 3.169950928 | -6.76238285 | 2.23E-11 | 7.85E-10 | 15.29071562 |
| RHEB | 0.185940551 | 4.600100435 | 6.760889293 | 2.26E-11 | 7.90E-10 | 15.28112813 |
| BCL11B | -0.199112358 | 1.762879523 | -6.74811646 | 2.46E-11 | 8.57E-10 | 15.1992154 |
| FNDC3A | -0.187939181 | 3.456197432 | -6.74469245 | 2.51E-11 | 8.74E-10 | 15.17728123 |
| MTHFD2 | 0.266054358 | 4.247812132 | 6.737114855 | 2.64E-11 | 9.16E-10 | 15.12877552 |
| RABGAP1L | -0.153719921 | 2.991139274 | -6.73424963 | 2.69E-11 | 9.30E-10 | 15.1104477 |
| ANKMY2 | -0.193021152 | 2.992949528 | -6.72905477 | 2.78E-11 | 9.57E-10 | 15.0772362 |
| DOCK1 | -0.208650428 | 3.343721885 | -6.72901031 | 2.79E-11 | 9.57E-10 | 15.07695207 |
| LDLR | 0.310422948 | 3.190710567 | 6.728048573 | 2.80E-11 | 9.60E-10 | 15.07080615 |
| ENY2 | 0.195237676 | 3.904568192 | 6.726987585 | 2.82E-11 | 9.64E-10 | 15.06402693 |
| CHCHD2 | 0.185867193 | 5.940078692 | 6.725600134 | 2.85E-11 | 9.70E-10 | 15.05516324 |
| ATP6V0A1 | -0.177830219 | 3.131916404 | -6.72183681 | 2.92E-11 | 9.91E-10 | 15.03112978 |
| LAPTM4B | 0.296071609 | 5.794785955 | 6.720852411 | 2.94E-11 | 9.94E-10 | 15.02484524 |
| EIF2S1 | 0.162494023 | 3.710246724 | 6.713286716 | 3.09E-11 | 1.04E-09 | 14.97657283 |
| ENPP2 | -0.293478449 | 2.644218663 | -6.69334753 | 3.52E-11 | 1.18E-09 | 14.84959131 |
| FMO4 | -0.184302226 | 2.407107905 | -6.6881694 | 3.64E-11 | 1.22E-09 | 14.81667145 |
| C18orf25 | 0.213531599 | 3.083498478 | 6.687063108 | 3.67E-11 | 1.23E-09 | 14.80964126 |
| MFAP2 | -0.4807248 | 4.139624801 | -6.68505499 | 3.72E-11 | 1.24E-09 | 14.79688289 |
| SLC25A1 | 0.234478147 | 4.341834337 | 6.676254703 | 3.94E-11 | 1.31E-09 | 14.74101285 |
| CD22 | -0.435718381 | 2.523880008 | -6.67548342 | 3.96E-11 | 1.31E-09 | 14.73611943 |
| ANGPTL1 | -0.362818297 | 1.664641431 | -6.67530368 | 3.96E-11 | 1.31E-09 | 14.73497919 |
| STIP1 | 0.178398755 | 4.668445566 | 6.673462744 | 4.01E-11 | 1.32E-09 | 14.72330177 |
| ATP1A2 | -0.392293252 | 1.553594209 | -6.66431676 | 4.26E-11 | 1.40E-09 | 14.66533095 |
| ANKRD9 | 0.239764152 | 2.779289556 | 6.658645096 | 4.42E-11 | 1.45E-09 | 14.62941841 |
| COL9A3 | -0.461342229 | 1.995434514 | -6.65780341 | 4.44E-11 | 1.45E-09 | 14.62409133 |
| KIF15 | 0.231229268 | 2.755004552 | 6.656388614 | 4.49E-11 | 1.46E-09 | 14.61513837 |
| NRIP2 | -0.205525463 | 1.935502236 | -6.65288495 | 4.59E-11 | 1.49E-09 | 14.59297442 |
| VTCN1 | 0.611193405 | 4.211556172 | 6.648620461 | 4.72E-11 | 1.53E-09 | 14.56601197 |
| NLRP1 | -0.225534591 | 2.595745636 | -6.6413833 | 4.95E-11 | 1.60E-09 | 14.52029104 |
| MAD2L1 | 0.29168778 | 3.097603382 | 6.639874276 | 5.00E-11 | 1.61E-09 | 14.51076354 |
| KCNJ5 | -0.223477688 | 2.25943937 | -6.63697892 | 5.09E-11 | 1.63E-09 | 14.4924887 |
| SMC2 | 0.220569369 | 3.159590713 | 6.635545151 | 5.14E-11 | 1.64E-09 | 14.48344181 |
| CKAP2L | 0.238346169 | 2.703625837 | 6.635308711 | 5.15E-11 | 1.64E-09 | 14.48195007 |
| SETBP1 | -0.226736909 | 2.576965578 | -6.63062711 | 5.31E-11 | 1.69E-09 | 14.45242325 |
| CSTF2 | 0.169991989 | 3.15016201 | 6.62093707 | 5.65E-11 | 1.79E-09 | 14.39136907 |
| AGPAT4 | -0.203609483 | 2.493023284 | -6.61766695 | 5.77E-11 | 1.82E-09 | 14.37078351 |
| TSPYL4 | -0.22517659 | 3.458450246 | -6.6155621 | 5.85E-11 | 1.84E-09 | 14.35753834 |
| PRELID1 | 0.188705861 | 4.72462993 | 6.614308453 | 5.90E-11 | 1.85E-09 | 14.34965138 |
| CXCL17 | 0.758208602 | 3.325548523 | 6.605797087 | 6.23E-11 | 1.95E-09 | 14.29614086 |
| FAM50A | 0.230914396 | 4.103458801 | 6.605575919 | 6.24E-11 | 1.95E-09 | 14.29475124 |
| OSR1 | -0.316285079 | 1.876370016 | -6.60306666 | 6.35E-11 | 1.98E-09 | 14.27898823 |
| BLMH | -0.185680934 | 2.955976769 | -6.60111034 | 6.43E-11 | 2.00E-09 | 14.26670259 |
| GAS7 | -0.239770167 | 2.875187701 | -6.59415817 | 6.72E-11 | 2.08E-09 | 14.22307023 |
| LAGE3 | 0.285011375 | 3.924951635 | 6.592066684 | 6.81E-11 | 2.11E-09 | 14.2099522 |
| IDH3G | 0.19079387 | 4.138350092 | 6.582556871 | 7.25E-11 | 2.23E-09 | 14.15035387 |
| NMU | 0.457164508 | 3.350148547 | 6.578862518 | 7.42E-11 | 2.28E-09 | 14.12722259 |
| SOX6 | -0.218074453 | 2.316095399 | -6.57222552 | 7.75E-11 | 2.37E-09 | 14.08569676 |
| AOX1 | -0.302046276 | 1.971225677 | -6.56949553 | 7.88E-11 | 2.41E-09 | 14.06862711 |
| CSTB | 0.237021588 | 4.698499148 | 6.565241774 | 8.10E-11 | 2.47E-09 | 14.04204305 |
| PLXDC2 | -0.229394621 | 3.659884701 | -6.56393753 | 8.17E-11 | 2.47E-09 | 14.03389529 |
| DHCR7 | 0.232637103 | 3.811694306 | 6.563758358 | 8.18E-11 | 2.47E-09 | 14.03277608 |
| HSPA12B | -0.225540182 | 2.118195431 | -6.56367819 | 8.19E-11 | 2.47E-09 | 14.0322753 |
| HMGCS1 | 0.231038806 | 3.440957619 | 6.563317325 | 8.20E-11 | 2.47E-09 | 14.0300213 |
| KLK13 | 0.412061245 | 2.153073332 | 6.558366028 | 8.47E-11 | 2.55E-09 | 13.99910622 |
| ACTL6A | 0.198908356 | 4.17764221 | 6.554902458 | 8.66E-11 | 2.60E-09 | 13.97749304 |
| GLA | 0.201007249 | 3.298923296 | 6.552833958 | 8.78E-11 | 2.62E-09 | 13.96459031 |
| ITIH3 | -0.26660694 | 1.547545333 | -6.54951785 | 8.97E-11 | 2.67E-09 | 13.94391319 |
| USP1 | 0.220101783 | 4.026546593 | 6.52984135 | 1.02E-10 | 3.03E-09 | 13.82142165 |
| RNASEH2A | 0.269643365 | 3.894131301 | 6.529366923 | 1.02E-10 | 3.03E-09 | 13.81847241 |
| PSMA5 | 0.167302385 | 3.986945869 | 6.523651456 | 1.06E-10 | 3.13E-09 | 13.78295816 |
| S100A9 | 0.643709239 | 4.019014419 | 6.522192632 | 1.07E-10 | 3.15E-09 | 13.77389804 |
| CACNA1G | -0.246714622 | 1.759341773 | -6.52187665 | 1.07E-10 | 3.15E-09 | 13.77193588 |
| UBQLN1 | 0.166578281 | 4.055371775 | 6.520659209 | 1.08E-10 | 3.17E-09 | 13.76437664 |
| MAGT1 | 0.192045565 | 4.160911939 | 6.519132241 | 1.09E-10 | 3.19E-09 | 13.75489737 |
| SAT1 | 0.282775338 | 5.490733042 | 6.515341326 | 1.12E-10 | 3.26E-09 | 13.73137259 |
| SELP | -0.261759031 | 1.586052238 | -6.51127485 | 1.15E-10 | 3.34E-09 | 13.70615185 |
| BCAP31 | 0.227137898 | 4.925311165 | 6.507949545 | 1.17E-10 | 3.40E-09 | 13.68553869 |
| FAM122B | 0.207126073 | 3.732385297 | 6.503968711 | 1.20E-10 | 3.48E-09 | 13.6608748 |
| GLB1L | -0.166927312 | 2.580513893 | -6.49598515 | 1.26E-10 | 3.65E-09 | 13.61145334 |
| EXTL2 | -0.171027304 | 3.148383339 | -6.4895111 | 1.32E-10 | 3.79E-09 | 13.57141751 |
| IQCA1 | -0.266919121 | 2.911155456 | -6.48785198 | 1.33E-10 | 3.83E-09 | 13.56116331 |
| TRIB3 | 0.31602256 | 2.923899142 | 6.486881106 | 1.34E-10 | 3.84E-09 | 13.55516399 |
| LAMA2 | -0.255303447 | 2.150654922 | -6.4757873 | 1.44E-10 | 4.11E-09 | 13.48667071 |
| METTL7A | -0.24682847 | 3.649392115 | -6.4742387 | 1.45E-10 | 4.14E-09 | 13.4771182 |
| EIF2S2 | 0.162005799 | 4.601753016 | 6.467734403 | 1.51E-10 | 4.30E-09 | 13.4370198 |
| CNRIP1 | -0.248171025 | 2.173982639 | -6.45079638 | 1.69E-10 | 4.78E-09 | 13.33277307 |
| ST6GAL2 | -0.305400491 | 2.141427914 | -6.44957827 | 1.70E-10 | 4.81E-09 | 13.32528584 |
| S100A14 | 0.402777533 | 4.251801155 | 6.447328927 | 1.72E-10 | 4.86E-09 | 13.31146346 |
| FXYD6 | -0.362749704 | 3.022529163 | -6.44478323 | 1.75E-10 | 4.93E-09 | 13.29582533 |
| ZBED3 | -0.222436215 | 2.937029045 | -6.44233113 | 1.78E-10 | 4.99E-09 | 13.28076753 |
| TMEM38B | 0.278688302 | 2.353609494 | 6.43513334 | 1.86E-10 | 5.21E-09 | 13.23659818 |
| ATP6V1C1 | 0.19985909 | 3.83031606 | 6.43444329 | 1.87E-10 | 5.22E-09 | 13.23236607 |
| ADH1B | -0.473342937 | 1.997404136 | -6.43056968 | 1.92E-10 | 5.34E-09 | 13.20861684 |
| BARD1 | 0.189917295 | 2.813957279 | 6.421281637 | 2.03E-10 | 5.65E-09 | 13.15172539 |
| IL4I1 | 0.423655979 | 3.892819407 | 6.411292181 | 2.16E-10 | 6.00E-09 | 13.09062249 |
| HSPB11 | 0.208429842 | 3.54852058 | 6.41088231 | 2.17E-10 | 6.00E-09 | 13.08811729 |
| STAT6 | -0.200963547 | 4.477066513 | -6.40874441 | 2.20E-10 | 6.07E-09 | 13.07505251 |
| CKS1B | 0.25659287 | 4.20711993 | 6.408118747 | 2.21E-10 | 6.08E-09 | 13.07122983 |
| OSTF1 | 0.199656829 | 3.625535419 | 6.401034697 | 2.31E-10 | 6.34E-09 | 13.02797168 |
| MT1X | 0.348604907 | 3.872629335 | 6.40054687 | 2.32E-10 | 6.34E-09 | 13.02499444 |
| BTG2 | -0.295098264 | 4.385014902 | -6.39754502 | 2.36E-10 | 6.44E-09 | 13.00667854 |
| CRISPLD1 | -0.372711256 | 2.334062022 | -6.39732683 | 2.36E-10 | 6.44E-09 | 13.00534758 |
| GALK1 | 0.196458261 | 2.911475316 | 6.396927507 | 2.37E-10 | 6.44E-09 | 13.00291175 |
| TUBA1B | 0.204550916 | 5.814840259 | 6.395634395 | 2.39E-10 | 6.48E-09 | 12.99502493 |
| CXorf38 | 0.191803595 | 3.027067179 | 6.393039057 | 2.43E-10 | 6.57E-09 | 12.97920017 |
| DSCC1 | 0.232763135 | 2.980665655 | 6.39050017 | 2.47E-10 | 6.66E-09 | 12.96372535 |
| POLQ | 0.202983548 | 2.576785306 | 6.386381569 | 2.53E-10 | 6.82E-09 | 12.93863409 |
| RIPK2 | 0.23016421 | 3.40387034 | 6.384742213 | 2.56E-10 | 6.87E-09 | 12.92865101 |
| FOXM1 | 0.303492728 | 3.923620183 | 6.379739668 | 2.64E-10 | 7.07E-09 | 12.89820197 |
| TAGLN2 | 0.188172445 | 5.833441809 | 6.37805798 | 2.67E-10 | 7.13E-09 | 12.88797099 |
| GRASP | -0.234359625 | 2.760790758 | -6.3767867 | 2.69E-10 | 7.17E-09 | 12.88023847 |
| IRS1 | -0.254501321 | 3.01843604 | -6.37573925 | 2.71E-10 | 7.20E-09 | 12.87386849 |
| HDHD3 | 0.209129617 | 3.585784694 | 6.3727674 | 2.76E-10 | 7.32E-09 | 12.85580067 |
| SVIL | -0.228522142 | 3.579271739 | -6.37137689 | 2.78E-10 | 7.37E-09 | 12.84734951 |
| DKK3 | -0.307241511 | 3.085980391 | -6.36995853 | 2.81E-10 | 7.41E-09 | 12.83873084 |
| ZBTB4 | -0.192265606 | 4.057182647 | -6.3692588 | 2.82E-10 | 7.43E-09 | 12.8344796 |
| OCIAD2 | 0.217990755 | 4.134449696 | 6.368973933 | 2.83E-10 | 7.43E-09 | 12.83274902 |
| ZFHX3 | -0.167916567 | 3.188068244 | -6.36538831 | 2.89E-10 | 7.58E-09 | 12.81097207 |
| PSMA7 | 0.1731996 | 5.122069095 | 6.362242199 | 2.95E-10 | 7.71E-09 | 12.79187377 |
| KIF11 | 0.247255482 | 3.430325284 | 6.348546385 | 3.21E-10 | 8.38E-09 | 12.70883603 |
| EFNA1 | 0.24532309 | 4.221396576 | 6.344226351 | 3.30E-10 | 8.59E-09 | 12.68267804 |
| CLEC10A | -0.314432854 | 1.608761708 | -6.34312883 | 3.32E-10 | 8.63E-09 | 12.67603516 |
| SETDB2 | -0.128160131 | 2.439808867 | -6.33649229 | 3.46E-10 | 8.97E-09 | 12.63588916 |
| CLPB | 0.177887229 | 3.093168266 | 6.333139651 | 3.54E-10 | 9.14E-09 | 12.6156231 |
| DAPK2 | 0.241294944 | 2.211867558 | 6.330945312 | 3.59E-10 | 9.23E-09 | 12.60236411 |
| NAALAD2 | -0.246479608 | 1.439404215 | -6.33086631 | 3.59E-10 | 9.23E-09 | 12.60188684 |
| ADCK5 | 0.242991515 | 3.087889132 | 6.330329485 | 3.60E-10 | 9.24E-09 | 12.59864383 |
| PI3 | 0.648888659 | 3.479766539 | 6.329549029 | 3.62E-10 | 9.26E-09 | 12.59392948 |
| IGF1 | -0.298346239 | 2.79728121 | -6.32582985 | 3.70E-10 | 9.46E-09 | 12.57147118 |
| TACC2 | -0.203543455 | 2.801062433 | -6.32204165 | 3.79E-10 | 9.66E-09 | 12.54860865 |
| COX6A1 | 0.228099667 | 4.911940783 | 6.316412187 | 3.93E-10 | 9.98E-09 | 12.5146572 |
| PHLDB1 | -0.209052841 | 3.433611171 | -6.31608594 | 3.93E-10 | 9.98E-09 | 12.51269043 |
| ASS1 | 0.300671755 | 5.808922706 | 6.313641155 | 4.00E-10 | 1.01E-08 | 12.49795532 |
| ZCCHC24 | -0.252817965 | 2.971588442 | -6.31299166 | 4.01E-10 | 1.01E-08 | 12.49404158 |
| IGDCC4 | -0.215030656 | 2.049182344 | -6.31137115 | 4.05E-10 | 1.02E-08 | 12.48427836 |
| FKBP9 | -0.193138042 | 4.408729978 | -6.30887136 | 4.12E-10 | 1.03E-08 | 12.46922218 |
| CCDC102A | -0.206552008 | 2.979606018 | -6.30827902 | 4.13E-10 | 1.04E-08 | 12.46565535 |
| MAPRE2 | -0.189902322 | 2.770851147 | -6.30371864 | 4.25E-10 | 1.06E-08 | 12.43820496 |
| ABHD5 | 0.177665639 | 2.746383504 | 6.303637326 | 4.25E-10 | 1.06E-08 | 12.43771567 |
| PPWD1 | -0.156919148 | 3.0327755 | -6.30289081 | 4.27E-10 | 1.06E-08 | 12.43322399 |
| TACC3 | 0.249319312 | 3.708913119 | 6.298286356 | 4.40E-10 | 1.09E-08 | 12.40553031 |
| MRPL21 | 0.201474022 | 4.073186287 | 6.295762099 | 4.47E-10 | 1.11E-08 | 12.39035604 |
| SPARCL1 | -0.303524751 | 4.176600492 | -6.29146194 | 4.59E-10 | 1.13E-08 | 12.36451915 |
| RRAGD | 0.28364578 | 2.839899907 | 6.289937015 | 4.63E-10 | 1.14E-08 | 12.35536079 |
| PIGA | 0.162661548 | 2.523890975 | 6.287395447 | 4.70E-10 | 1.16E-08 | 12.34010126 |
| HSPA5 | 0.183187366 | 5.522198676 | 6.283814053 | 4.81E-10 | 1.18E-08 | 12.31860836 |
| LCN2 | 0.584313815 | 4.96876095 | 6.282522027 | 4.85E-10 | 1.19E-08 | 12.31085736 |
| MRPS22 | 0.155581264 | 3.571277595 | 6.280672158 | 4.90E-10 | 1.20E-08 | 12.29976236 |
| ZNF608 | -0.229231437 | 3.256881508 | -6.28062721 | 4.91E-10 | 1.20E-08 | 12.29949285 |
| IDO1 | 0.499766725 | 2.876052088 | 6.27836831 | 4.97E-10 | 1.21E-08 | 12.28594877 |
| SESN3 | -0.236678569 | 3.13050359 | -6.27708119 | 5.01E-10 | 1.22E-08 | 12.27823343 |
| RIC3 | -0.344494982 | 2.342223314 | -6.27280848 | 5.15E-10 | 1.25E-08 | 12.25263205 |
| CDKN1C | -0.34162089 | 3.347093878 | -6.27269414 | 5.15E-10 | 1.25E-08 | 12.25194717 |
| AKAP13 | -0.155142019 | 3.436386544 | -6.26853648 | 5.29E-10 | 1.28E-08 | 12.22705119 |
| GPM6A | -0.316157997 | 1.318762171 | -6.26777017 | 5.31E-10 | 1.28E-08 | 12.2224642 |
| INPP5F | -0.162282446 | 2.890588126 | -6.26523475 | 5.40E-10 | 1.30E-08 | 12.20729135 |
| PPME1 | 0.149211772 | 3.610532059 | 6.262850646 | 5.48E-10 | 1.31E-08 | 12.19302927 |
| BTRC | -0.135183583 | 2.859964836 | -6.25999043 | 5.57E-10 | 1.33E-08 | 12.17592565 |
| ARTN | 0.276459277 | 2.421743627 | 6.256422871 | 5.70E-10 | 1.36E-08 | 12.15460238 |
| PAM | -0.281630242 | 4.228380512 | -6.25567824 | 5.72E-10 | 1.36E-08 | 12.15015315 |
| APOD | -0.369887066 | 2.713538545 | -6.25335541 | 5.81E-10 | 1.38E-08 | 12.1362772 |
| C8orf33 | 0.249083772 | 4.287315016 | 6.249899468 | 5.93E-10 | 1.41E-08 | 12.11564125 |
| DCP1B | -0.181648737 | 3.188217575 | -6.24680793 | 6.05E-10 | 1.43E-08 | 12.09719014 |
| ANKRD13B | -0.242956362 | 2.440189699 | -6.24212276 | 6.22E-10 | 1.47E-08 | 12.06924402 |
| KHDRBS3 | 0.302586028 | 2.694769824 | 6.236792569 | 6.43E-10 | 1.52E-08 | 12.03747417 |
| SOX11 | -0.424298257 | 1.993592486 | -6.23524325 | 6.49E-10 | 1.53E-08 | 12.02824438 |
| RAD21 | 0.211225256 | 4.927176095 | 6.227798 | 6.80E-10 | 1.60E-08 | 11.98392044 |
| POP7 | 0.177473414 | 4.033065447 | 6.226848073 | 6.84E-10 | 1.60E-08 | 11.97826876 |
| LIMS2 | -0.234741236 | 2.967344183 | -6.2232979 | 6.99E-10 | 1.63E-08 | 11.95715377 |
| TUBA1C | 0.219023113 | 4.960126768 | 6.222807198 | 7.01E-10 | 1.64E-08 | 11.95423613 |
| SLC43A3 | 0.248333148 | 3.492342501 | 6.221296846 | 7.08E-10 | 1.65E-08 | 11.94525722 |
| COPB2 | 0.150303309 | 4.380947419 | 6.22024771 | 7.12E-10 | 1.65E-08 | 11.93902139 |
| ARF6 | 0.186942289 | 4.60200308 | 6.217781343 | 7.23E-10 | 1.68E-08 | 11.92436569 |
| PSME1 | 0.207126289 | 5.38801548 | 6.21360116 | 7.42E-10 | 1.72E-08 | 11.89953845 |
| OPRL1 | -0.206195829 | 1.875664417 | -6.2132609 | 7.43E-10 | 1.72E-08 | 11.89751822 |
| TGFB3 | -0.276632288 | 2.729533151 | -6.2053928 | 7.80E-10 | 1.80E-08 | 11.85083191 |
| DCHS1 | -0.249810989 | 2.691271755 | -6.20514089 | 7.81E-10 | 1.80E-08 | 11.84933812 |
| ENDOG | 0.238062878 | 3.081506877 | 6.201383879 | 8.00E-10 | 1.84E-08 | 11.82706567 |
| DLC1 | -0.193609751 | 2.373574214 | -6.19982039 | 8.07E-10 | 1.85E-08 | 11.81780064 |
| PPP1R12B | -0.15134969 | 2.645011411 | -6.18803148 | 8.68E-10 | 1.98E-08 | 11.74801104 |
| LTBP3 | -0.210021689 | 4.509651049 | -6.18766076 | 8.70E-10 | 1.98E-08 | 11.74581836 |
| RFK | 0.22092394 | 3.406189021 | 6.183564845 | 8.92E-10 | 2.03E-08 | 11.72160108 |
| AXL | -0.25054694 | 3.371284207 | -6.18339319 | 8.93E-10 | 2.03E-08 | 11.72058647 |
| CNIH4 | 0.165488563 | 3.515409278 | 6.182967405 | 8.95E-10 | 2.03E-08 | 11.71806993 |
| MTBP | 0.166430502 | 2.492624412 | 6.179513955 | 9.14E-10 | 2.07E-08 | 11.69766465 |
| PRKD1 | -0.202572071 | 2.349494807 | -6.17680045 | 9.30E-10 | 2.10E-08 | 11.6816389 |
| PTGES | 0.375284026 | 2.296074052 | 6.176584192 | 9.31E-10 | 2.10E-08 | 11.68036198 |
| NEIL3 | 0.226433633 | 2.213660413 | 6.173358829 | 9.49E-10 | 2.13E-08 | 11.66132237 |
| MMS19 | -0.164715324 | 3.941143671 | -6.16878484 | 9.76E-10 | 2.19E-08 | 11.63433759 |
| SRP54 | 0.139317435 | 3.804945196 | 6.1682684 | 9.79E-10 | 2.19E-08 | 11.63129193 |
| OLFML3 | -0.32560084 | 3.83848839 | -6.16556355 | 9.96E-10 | 2.22E-08 | 11.61534431 |
| EPC1 | -0.144047804 | 3.202022428 | -6.16255255 | 1.01E-09 | 2.26E-08 | 11.59759932 |
| GRPEL1 | 0.177010934 | 3.310524642 | 6.151025915 | 1.09E-09 | 2.42E-08 | 11.52974297 |
| ABCB1 | -0.197369027 | 1.551451599 | -6.14506471 | 1.13E-09 | 2.51E-08 | 11.49469622 |
| GHR | -0.219994983 | 1.873130718 | -6.14015796 | 1.16E-09 | 2.58E-08 | 11.4658725 |
| ROBO3 | -0.26170978 | 2.633335331 | -6.13871385 | 1.17E-09 | 2.59E-08 | 11.45739342 |
| CDK6 | -0.271410637 | 2.823331519 | -6.13273228 | 1.22E-09 | 2.68E-08 | 11.42229259 |
| CYCS | 0.206339138 | 4.414527324 | 6.130552338 | 1.23E-09 | 2.71E-08 | 11.40950821 |
| UST | -0.203720312 | 2.170007803 | -6.12772269 | 1.25E-09 | 2.75E-08 | 11.39291997 |
| TRERF1 | -0.193043634 | 2.604372017 | -6.12757799 | 1.25E-09 | 2.75E-08 | 11.39207184 |
| CHRAC1 | 0.211958725 | 4.034490116 | 6.122896322 | 1.29E-09 | 2.83E-08 | 11.36464276 |
| APLN | 0.266365449 | 2.372599411 | 6.121985527 | 1.30E-09 | 2.84E-08 | 11.35930883 |
| DBF4 | 0.182129687 | 2.980407923 | 6.120391289 | 1.31E-09 | 2.86E-08 | 11.34997421 |
| CSRNP3 | -0.181786586 | 2.191881206 | -6.11830994 | 1.33E-09 | 2.89E-08 | 11.33779085 |
| FLNC | -0.309099748 | 2.742305885 | -6.11796319 | 1.33E-09 | 2.89E-08 | 11.3357615 |
| WDR6 | -0.208320566 | 4.343378401 | -6.11451237 | 1.36E-09 | 2.95E-08 | 11.31557138 |
| FLI1 | -0.17838179 | 2.347956928 | -6.11138421 | 1.38E-09 | 3.00E-08 | 11.29727823 |
| IGF1R | -0.249263007 | 3.56320891 | -6.1096104 | 1.40E-09 | 3.02E-08 | 11.28690909 |
| CXCL10 | 0.528204972 | 4.161461678 | 6.108608336 | 1.41E-09 | 3.03E-08 | 11.28105256 |
| RNF181 | 0.162719896 | 4.747735894 | 6.10827847 | 1.41E-09 | 3.03E-08 | 11.27912487 |
| N4BP2L1 | -0.173361017 | 2.677788905 | -6.1036393 | 1.45E-09 | 3.11E-08 | 11.25202442 |
| NDUFA6 | 0.195466812 | 4.155941746 | 6.095652962 | 1.52E-09 | 3.26E-08 | 11.20541593 |
| SLC2A6 | 0.247435026 | 2.421854647 | 6.093594271 | 1.54E-09 | 3.30E-08 | 11.19341057 |
| RHOBTB1 | -0.212520235 | 2.754003387 | -6.09113062 | 1.56E-09 | 3.34E-08 | 11.1790486 |
| MEX3B | -0.2323703 | 1.939656449 | -6.08355231 | 1.64E-09 | 3.49E-08 | 11.13490452 |
| GRINA | 0.235838398 | 5.516346053 | 6.08063757 | 1.67E-09 | 3.54E-08 | 11.11793964 |
| GGH | 0.32732845 | 3.376346164 | 6.077075266 | 1.70E-09 | 3.61E-08 | 11.09721597 |
| GSTM2 | -0.245225301 | 3.113943927 | -6.06754057 | 1.80E-09 | 3.82E-08 | 11.04180368 |
| COX5B | 0.224252927 | 5.623787591 | 6.064067625 | 1.84E-09 | 3.89E-08 | 11.0216403 |
| BMP6 | -0.295079774 | 1.935348691 | -6.05989498 | 1.89E-09 | 3.99E-08 | 10.99742882 |
| LGI4 | -0.218805546 | 1.673369998 | -6.05590822 | 1.93E-09 | 4.07E-08 | 10.97431042 |
| CDT1 | 0.245735492 | 3.087141872 | 6.049215641 | 2.01E-09 | 4.23E-08 | 10.93553348 |
| PDZK1IP1 | 0.548877173 | 3.906791456 | 6.048802989 | 2.02E-09 | 4.24E-08 | 10.93314387 |
| MXRA8 | -0.389912202 | 4.07988719 | -6.04652209 | 2.05E-09 | 4.29E-08 | 10.9199383 |
| MT1G | 0.648307483 | 3.926107122 | 6.045676022 | 2.06E-09 | 4.30E-08 | 10.91504102 |
| IFFO1 | -0.190378823 | 2.49785014 | -6.04395074 | 2.08E-09 | 4.34E-08 | 10.90505664 |
| TRAM1 | 0.214645452 | 5.041601599 | 6.042619316 | 2.09E-09 | 4.36E-08 | 10.89735337 |
| MBOAT7 | 0.173909811 | 4.175979483 | 6.04143688 | 2.11E-09 | 4.38E-08 | 10.89051343 |
| GOT1 | 0.191203639 | 3.835972789 | 6.041278931 | 2.11E-09 | 4.38E-08 | 10.88959985 |
| CCNE2 | 0.241113415 | 2.104235189 | 6.040606446 | 2.12E-09 | 4.39E-08 | 10.88571044 |
| CNIH3 | -0.193170299 | 2.054870181 | -6.03143425 | 2.24E-09 | 4.63E-08 | 10.83270203 |
| PHACTR2 | -0.173430278 | 2.818266397 | -6.03120311 | 2.24E-09 | 4.63E-08 | 10.83136721 |
| FDFT1 | 0.221153691 | 4.229145075 | 6.027218464 | 2.30E-09 | 4.72E-08 | 10.80836318 |
| PDK2 | -0.1959879 | 3.281678447 | -6.02719761 | 2.30E-09 | 4.72E-08 | 10.80824282 |
| VASH1 | -0.187151273 | 2.960630723 | -6.01712068 | 2.44E-09 | 5.00E-08 | 10.75013057 |
| VEGFC | -0.246452909 | 2.490172012 | -6.01706388 | 2.44E-09 | 5.00E-08 | 10.74980326 |
| IFI27 | 0.450701683 | 5.550544305 | 6.016085141 | 2.45E-09 | 5.02E-08 | 10.74416389 |
| BUB3 | 0.152195692 | 3.789001675 | 6.00820474 | 2.57E-09 | 5.25E-08 | 10.69878925 |
| FAM124A | -0.262847874 | 2.475272237 | -6.00395183 | 2.64E-09 | 5.38E-08 | 10.67432448 |
| LMNB1 | 0.242945453 | 3.904349485 | 6.00076079 | 2.69E-09 | 5.47E-08 | 10.65597866 |
| ING5 | -0.159905906 | 2.961428865 | -6.00046819 | 2.69E-09 | 5.47E-08 | 10.65429694 |
| NOP16 | 0.189250013 | 3.351520926 | 5.985447628 | 2.95E-09 | 5.97E-08 | 10.56806765 |
| KLF15 | -0.260681898 | 2.366543317 | -5.97677484 | 3.10E-09 | 6.27E-08 | 10.51837129 |
| COX17 | 0.197692431 | 3.63087569 | 5.976425378 | 3.11E-09 | 6.27E-08 | 10.51637025 |
| PREX1 | -0.210361779 | 3.224289102 | -5.97450536 | 3.14E-09 | 6.33E-08 | 10.50537796 |
| TMBIM4 | -0.151601779 | 3.672681459 | -5.97211058 | 3.19E-09 | 6.41E-08 | 10.49167224 |
| NDUFA1 | 0.213786683 | 5.266600971 | 5.971127965 | 3.21E-09 | 6.44E-08 | 10.48605007 |
| ARNT2 | -0.266535094 | 2.995008724 | -5.96986347 | 3.23E-09 | 6.48E-08 | 10.47881634 |
| CHCHD4 | 0.180755987 | 3.187461877 | 5.963610668 | 3.35E-09 | 6.69E-08 | 10.44306734 |
| SCP2 | 0.157555796 | 4.121586132 | 5.96343315 | 3.36E-09 | 6.69E-08 | 10.44205292 |
| C5orf22 | 0.159391383 | 3.438978963 | 5.963325802 | 3.36E-09 | 6.69E-08 | 10.44143951 |
| PTK2 | 0.165651418 | 4.053912337 | 5.955418873 | 3.52E-09 | 6.99E-08 | 10.39628555 |
| UQCRQ | 0.212104967 | 4.996647122 | 5.955385441 | 3.52E-09 | 6.99E-08 | 10.39609475 |
| UBR1 | -0.126847557 | 2.777408764 | -5.95478503 | 3.53E-09 | 7.00E-08 | 10.39266832 |
| ECM2 | -0.246962706 | 2.312910371 | -5.95442652 | 3.54E-09 | 7.01E-08 | 10.39062251 |
| PLEKHF2 | 0.216651331 | 3.508276443 | 5.949810168 | 3.64E-09 | 7.19E-08 | 10.36428997 |
| CTDSP1 | -0.139126183 | 4.59527954 | -5.94854744 | 3.67E-09 | 7.23E-08 | 10.35709046 |
| TFPT | 0.188915146 | 3.697951376 | 5.945094189 | 3.74E-09 | 7.36E-08 | 10.33740887 |
| COX7A2 | 0.215209699 | 4.977361561 | 5.940428769 | 3.85E-09 | 7.56E-08 | 10.31083557 |
| FKBP7 | -0.24423507 | 2.626267552 | -5.93918945 | 3.87E-09 | 7.60E-08 | 10.30377992 |
| EXOSC3 | 0.146991002 | 3.302947762 | 5.937766499 | 3.91E-09 | 7.65E-08 | 10.29568056 |
| RAD54B | 0.201301029 | 2.396846878 | 5.935107766 | 3.97E-09 | 7.75E-08 | 10.28055202 |
| ARHGAP10 | -0.152335873 | 2.308500554 | -5.93486142 | 3.97E-09 | 7.75E-08 | 10.27915061 |
| NDUFB9 | 0.239653838 | 5.438339698 | 5.931099312 | 4.06E-09 | 7.91E-08 | 10.25775537 |
| PRDM2 | -0.151979495 | 3.429615525 | -5.92596381 | 4.19E-09 | 8.14E-08 | 10.2285701 |
| CHST12 | -0.132592643 | 2.548888346 | -5.92295721 | 4.26E-09 | 8.27E-08 | 10.21149444 |
| POMP | 0.173814551 | 4.544494699 | 5.922588041 | 4.27E-09 | 8.28E-08 | 10.20939833 |
| ACOT7 | 0.21491396 | 3.09532765 | 5.921563166 | 4.30E-09 | 8.31E-08 | 10.20357985 |
| SLPI | 0.440951252 | 6.688768791 | 5.908385442 | 4.65E-09 | 8.97E-08 | 10.1288505 |
| YBX2 | 0.296001722 | 2.682416232 | 5.904579337 | 4.75E-09 | 9.15E-08 | 10.10729551 |
| SCAPER | -0.134996048 | 2.713689251 | -5.90313006 | 4.79E-09 | 9.21E-08 | 10.09909126 |
| HSPB1 | 0.232048957 | 6.567608588 | 5.902714229 | 4.80E-09 | 9.21E-08 | 10.09673765 |
| GNG5 | 0.170581816 | 5.188707585 | 5.902585851 | 4.81E-09 | 9.21E-08 | 10.09601105 |
| BCL2 | -0.196900955 | 2.505937326 | -5.90080491 | 4.86E-09 | 9.29E-08 | 10.08593278 |
| PDZD11 | 0.166474025 | 4.18663331 | 5.900493153 | 4.87E-09 | 9.30E-08 | 10.08416883 |
| TBC1D2B | -0.16376078 | 3.03908673 | -5.89905535 | 4.91E-09 | 9.35E-08 | 10.07603484 |
| SDHB | 0.176594368 | 4.248604324 | 5.898875924 | 4.91E-09 | 9.35E-08 | 10.0750199 |
| TRIM13 | -0.142701004 | 2.870798757 | -5.89125644 | 5.14E-09 | 9.76E-08 | 10.03194678 |
| DAD1 | 0.176374986 | 5.456531404 | 5.889778116 | 5.18E-09 | 9.83E-08 | 10.02359584 |
| PPP1CB | 0.160117629 | 5.119688761 | 5.889139868 | 5.20E-09 | 9.85E-08 | 10.01999103 |
| ETFB | 0.175997385 | 3.253307669 | 5.884468066 | 5.35E-09 | 1.01E-07 | 9.993615887 |
| SLC24A1 | -0.15940512 | 2.558980295 | -5.88292934 | 5.39E-09 | 1.02E-07 | 9.984933132 |
| PRPF4 | 0.173770164 | 3.617948474 | 5.882541262 | 5.41E-09 | 1.02E-07 | 9.982743625 |
| HHAT | -0.187644197 | 2.162543878 | -5.87427067 | 5.67E-09 | 1.07E-07 | 9.936113547 |
| KLRG1 | -0.194241124 | 1.882535147 | -5.8736215 | 5.70E-09 | 1.07E-07 | 9.932456084 |
| NAGPA | -0.140023303 | 2.704573234 | -5.87239521 | 5.74E-09 | 1.08E-07 | 9.925548097 |
| RASA1 | -0.154977215 | 3.3209847 | -5.87102438 | 5.78E-09 | 1.08E-07 | 9.917827534 |
| RAD54L | 0.221701928 | 2.929703048 | 5.869615483 | 5.83E-09 | 1.09E-07 | 9.909894317 |
| ALDH3B2 | 0.428753878 | 3.529603915 | 5.863564659 | 6.04E-09 | 1.13E-07 | 9.875843625 |
| APOL2 | 0.253144989 | 3.855954927 | 5.858850664 | 6.21E-09 | 1.15E-07 | 9.849338665 |
| KIFC2 | 0.287522724 | 3.788482411 | 5.858844072 | 6.21E-09 | 1.15E-07 | 9.849301617 |
| POLA2 | 0.170181492 | 3.207770351 | 5.858707001 | 6.21E-09 | 1.15E-07 | 9.848531218 |
| CDCA3 | 0.226683752 | 3.15144931 | 5.854952154 | 6.35E-09 | 1.18E-07 | 9.827433953 |
| IFT88 | -0.149110405 | 2.676069915 | -5.85489473 | 6.35E-09 | 1.18E-07 | 9.827111389 |
| WDR5 | 0.149321507 | 3.661402606 | 5.853186757 | 6.42E-09 | 1.18E-07 | 9.817519136 |
| MRPL49 | 0.154913288 | 4.150654593 | 5.851649365 | 6.47E-09 | 1.19E-07 | 9.808887126 |
| PRELID2 | 0.145790518 | 2.279383004 | 5.846470093 | 6.67E-09 | 1.23E-07 | 9.779822652 |
| COMTD1 | 0.297241985 | 3.547041607 | 5.839372243 | 6.95E-09 | 1.28E-07 | 9.740030878 |
| MXD3 | 0.187721547 | 2.851697661 | 5.838427541 | 6.99E-09 | 1.28E-07 | 9.734738132 |
| CYBA | 0.248751768 | 4.990261927 | 5.835115424 | 7.13E-09 | 1.31E-07 | 9.716188153 |
| ADH1C | -0.267449883 | 1.091294129 | -5.83440651 | 7.16E-09 | 1.31E-07 | 9.712219036 |
| NUP155 | 0.154089501 | 3.407923512 | 5.831941715 | 7.26E-09 | 1.33E-07 | 9.698422604 |
| PARL | 0.151825236 | 4.146068574 | 5.830558382 | 7.32E-09 | 1.33E-07 | 9.69068192 |
| RAB42 | 0.19883801 | 2.327136951 | 5.82805823 | 7.43E-09 | 1.35E-07 | 9.676696245 |
| DIAPH3 | 0.179607149 | 2.523846032 | 5.825369418 | 7.54E-09 | 1.37E-07 | 9.661661499 |
| RNF19A | 0.199438286 | 3.838300708 | 5.821037619 | 7.74E-09 | 1.40E-07 | 9.637453517 |
| SLC39A4 | 0.2935075 | 4.016209759 | 5.820890561 | 7.74E-09 | 1.40E-07 | 9.63663199 |
| SATB1 | -0.216323687 | 2.550238653 | -5.82087306 | 7.74E-09 | 1.40E-07 | 9.6365342 |
| KLK8 | 0.353826595 | 4.115514884 | 5.816269795 | 7.95E-09 | 1.43E-07 | 9.610828357 |
| TSPAN32 | -0.223639985 | 1.858059292 | -5.81542524 | 7.99E-09 | 1.44E-07 | 9.606114181 |
| SERP2 | -0.234436212 | 1.645367452 | -5.81538645 | 7.99E-09 | 1.44E-07 | 9.605897686 |
| ZBTB20 | -0.149317818 | 2.504256646 | -5.81380037 | 8.07E-09 | 1.45E-07 | 9.597046292 |
| TNXB | -0.25388477 | 2.236084342 | -5.81379983 | 8.07E-09 | 1.45E-07 | 9.59704328 |
| UBE2H | 0.170141054 | 4.758885559 | 5.812693055 | 8.12E-09 | 1.45E-07 | 9.590868043 |
| RAB11FIP2 | -0.185446662 | 3.328654562 | -5.80564907 | 8.46E-09 | 1.51E-07 | 9.551592115 |
| SLC26A6 | 0.156950381 | 2.706120916 | 5.802824898 | 8.60E-09 | 1.53E-07 | 9.535857646 |
| ACRBP | -0.227092295 | 2.139990397 | -5.79948116 | 8.76E-09 | 1.56E-07 | 9.51723772 |
| GCH1 | 0.206496179 | 2.575835262 | 5.79834179 | 8.82E-09 | 1.57E-07 | 9.510895365 |
| CLDN15 | -0.195309724 | 2.790125581 | -5.79760063 | 8.86E-09 | 1.57E-07 | 9.506770281 |
| SREBF1 | 0.213824892 | 3.624436976 | 5.796932535 | 8.89E-09 | 1.58E-07 | 9.503052262 |
| PRDX1 | 0.161740094 | 5.550012793 | 5.79420544 | 9.03E-09 | 1.60E-07 | 9.487879908 |
| MN1 | -0.251855862 | 2.363861631 | -5.79163605 | 9.17E-09 | 1.62E-07 | 9.473591054 |
| CCNE1 | 0.332283299 | 3.567837241 | 5.791468481 | 9.18E-09 | 1.62E-07 | 9.472659405 |
| PLD2 | -0.170126925 | 3.216316423 | -5.7900975 | 9.25E-09 | 1.63E-07 | 9.465037776 |
| SNRPG | 0.182255953 | 4.354121276 | 5.786721151 | 9.43E-09 | 1.66E-07 | 9.446275032 |
| EIF4EBP1 | 0.261471757 | 4.422163463 | 5.786184111 | 9.46E-09 | 1.66E-07 | 9.443291584 |
| CYP27A1 | -0.22169238 | 3.098845334 | -5.78566036 | 9.49E-09 | 1.66E-07 | 9.440382197 |
| SULT1C4 | -0.299163879 | 1.921009138 | -5.78419194 | 9.57E-09 | 1.68E-07 | 9.432226643 |
| PLXNC1 | -0.186188003 | 2.700783902 | -5.78289045 | 9.64E-09 | 1.69E-07 | 9.424999827 |
| C1QTNF7 | -0.251408744 | 1.845082777 | -5.78197967 | 9.69E-09 | 1.69E-07 | 9.419943417 |
| SEMA6C | -0.239983855 | 2.797166861 | -5.78021751 | 9.79E-09 | 1.71E-07 | 9.410162537 |
| GFM1 | 0.145683073 | 3.391690167 | 5.778428406 | 9.90E-09 | 1.72E-07 | 9.400234912 |
| TROAP | 0.25165073 | 3.491100533 | 5.774625695 | 1.01E-08 | 1.76E-07 | 9.379143534 |
| PRELP | -0.374044694 | 3.267718589 | -5.77319509 | 1.02E-08 | 1.77E-07 | 9.371212181 |
| MFN1 | 0.15229195 | 3.895915208 | 5.770914388 | 1.03E-08 | 1.79E-07 | 9.358571687 |
| DCTPP1 | 0.195906819 | 4.292773732 | 5.768075242 | 1.05E-08 | 1.82E-07 | 9.342842627 |
| AASS | -0.171908538 | 2.103464878 | -5.76308153 | 1.08E-08 | 1.87E-07 | 9.315194737 |
| RCSD1 | -0.185667243 | 2.321846928 | -5.76276784 | 1.08E-08 | 1.87E-07 | 9.313458759 |
| EMCN | -0.197913071 | 1.958580149 | -5.76035051 | 1.10E-08 | 1.89E-07 | 9.300083853 |
| PFKFB3 | 0.242502142 | 3.958636421 | 5.758330969 | 1.11E-08 | 1.91E-07 | 9.288913958 |
| RASSF2 | -0.202316448 | 2.882660351 | -5.75814251 | 1.11E-08 | 1.91E-07 | 9.287871773 |
| SURF2 | 0.182234787 | 3.408543673 | 5.757727631 | 1.11E-08 | 1.91E-07 | 9.285577662 |
| ATOH8 | -0.263442506 | 1.886141445 | -5.74889792 | 1.17E-08 | 2.00E-07 | 9.2367893 |
| GABBR1 | -0.25651482 | 2.942131918 | -5.74629741 | 1.19E-08 | 2.03E-07 | 9.222433648 |
| ANKS1B | -0.163824777 | 2.075270072 | -5.74567007 | 1.19E-08 | 2.04E-07 | 9.218971448 |
| SLC25A10 | 0.193534329 | 3.051034236 | 5.737504391 | 1.25E-08 | 2.13E-07 | 9.173938511 |
| SLC25A39 | 0.153837875 | 4.849966246 | 5.736327628 | 1.26E-08 | 2.14E-07 | 9.167453734 |
| ADAM33 | -0.279762493 | 2.464344352 | -5.73469124 | 1.27E-08 | 2.16E-07 | 9.158438208 |
| HAPLN1 | 0.265828362 | 1.488987689 | 5.733868395 | 1.28E-08 | 2.16E-07 | 9.15390571 |
| KCNIP3 | -0.225836766 | 2.383349601 | -5.73086033 | 1.30E-08 | 2.20E-07 | 9.137341549 |
| PDGFD | -0.24778053 | 2.585736977 | -5.72305058 | 1.36E-08 | 2.30E-07 | 9.094374682 |
| MPDZ | -0.164411136 | 2.889116914 | -5.71827306 | 1.40E-08 | 2.36E-07 | 9.068117329 |
| TSPAN4 | -0.187793381 | 3.35633171 | -5.71590248 | 1.42E-08 | 2.38E-07 | 9.055096271 |
| ANAPC11 | 0.192604802 | 3.936310941 | 5.709865534 | 1.47E-08 | 2.46E-07 | 9.021959489 |
| SUFU | -0.139221755 | 2.940230732 | -5.70966641 | 1.47E-08 | 2.46E-07 | 9.020867084 |
| ZFP2 | -0.197272834 | 1.769396645 | -5.70862714 | 1.48E-08 | 2.47E-07 | 9.015166014 |
| DEFB1 | 0.652124865 | 3.856049535 | 5.708163721 | 1.48E-08 | 2.48E-07 | 9.012624194 |
| BRI3BP | 0.186374038 | 3.318530259 | 5.705077175 | 1.51E-08 | 2.52E-07 | 8.995699618 |
| GLI3 | -0.229428416 | 2.789520965 | -5.70455331 | 1.51E-08 | 2.52E-07 | 8.992827961 |
| HSPA9 | 0.150267435 | 4.815525278 | 5.704221183 | 1.51E-08 | 2.52E-07 | 8.991007453 |
| CDK5 | 0.191025993 | 3.067258328 | 5.702243512 | 1.53E-08 | 2.55E-07 | 8.980169267 |
| TFR2 | 0.251259952 | 1.669949811 | 5.700945209 | 1.54E-08 | 2.56E-07 | 8.973056128 |
| ADRB2 | -0.200160746 | 1.52032489 | -5.69348197 | 1.61E-08 | 2.67E-07 | 8.932196065 |
| NUDT22 | 0.161577651 | 3.320175291 | 5.692442062 | 1.62E-08 | 2.68E-07 | 8.926506756 |
| PLSCR4 | -0.267477303 | 3.227234085 | -5.68747977 | 1.66E-08 | 2.75E-07 | 8.899371531 |
| SULF2 | -0.307203011 | 3.453002342 | -5.68608399 | 1.68E-08 | 2.77E-07 | 8.891743008 |
| ARPC5 | 0.165143095 | 4.422065318 | 5.685640246 | 1.68E-08 | 2.77E-07 | 8.889318134 |
| DES | -0.372364943 | 2.456482447 | -5.68441835 | 1.69E-08 | 2.79E-07 | 8.882641909 |
| HTRA3 | -0.318209181 | 2.857375133 | -5.68123437 | 1.73E-08 | 2.84E-07 | 8.865251528 |
| RUVBL2 | 0.151416526 | 4.262555383 | 5.679962362 | 1.74E-08 | 2.85E-07 | 8.858306581 |
| LDB1 | -0.184077029 | 4.6492289 | -5.67057469 | 1.83E-08 | 3.00E-07 | 8.80709675 |
| RHOBTB2 | -0.165665846 | 2.796388149 | -5.66818491 | 1.86E-08 | 3.04E-07 | 8.794073218 |
| RAD17 | -0.125912696 | 3.211890135 | -5.66546331 | 1.89E-08 | 3.08E-07 | 8.779247697 |
| ASAP3 | -0.208646964 | 3.399235853 | -5.66318696 | 1.91E-08 | 3.12E-07 | 8.766852718 |
| GHRL | -0.19070511 | 1.608557872 | -5.66177688 | 1.93E-08 | 3.14E-07 | 8.759177007 |
| TMPRSS4 | 0.393125545 | 3.514205063 | 5.659456874 | 1.95E-08 | 3.17E-07 | 8.746552101 |
| TMEM69 | 0.153050648 | 3.94548037 | 5.659418605 | 1.95E-08 | 3.17E-07 | 8.746343889 |
| BUD31 | 0.152895251 | 4.21895307 | 5.659247228 | 1.95E-08 | 3.17E-07 | 8.745411498 |
| EMD | 0.16841417 | 4.267393579 | 5.658994329 | 1.96E-08 | 3.17E-07 | 8.744035622 |
| RASL11B | -0.289594065 | 1.9134627 | -5.65645544 | 1.99E-08 | 3.21E-07 | 8.730226238 |
| RABIF | 0.147027531 | 3.108475968 | 5.653703928 | 2.02E-08 | 3.26E-07 | 8.715266976 |
| PODN | -0.279151317 | 2.732531876 | -5.65321521 | 2.02E-08 | 3.26E-07 | 8.712610632 |
| TNFAIP2 | 0.336731094 | 5.190122147 | 5.650588702 | 2.05E-08 | 3.30E-07 | 8.698338548 |
| SEC61B | 0.15592862 | 4.501726207 | 5.650501648 | 2.05E-08 | 3.30E-07 | 8.697865617 |
| LETMD1 | -0.162333623 | 3.744058023 | -5.64148427 | 2.16E-08 | 3.47E-07 | 8.648914536 |
| IL16 | -0.176510046 | 2.462944671 | -5.63410451 | 2.25E-08 | 3.61E-07 | 8.608908107 |
| IRX3 | 0.466011147 | 3.00542721 | 5.633313683 | 2.26E-08 | 3.62E-07 | 8.604623872 |
| CKAP2 | 0.191629159 | 3.435828154 | 5.626627039 | 2.35E-08 | 3.75E-07 | 8.568422239 |
| CLUAP1 | -0.152549003 | 2.915498466 | -5.6213155 | 2.42E-08 | 3.86E-07 | 8.539694297 |
| MKI67 | 0.250804829 | 3.716121633 | 5.619464412 | 2.45E-08 | 3.90E-07 | 8.529688536 |
| DOCK10 | -0.160720432 | 2.354616199 | -5.61895778 | 2.45E-08 | 3.90E-07 | 8.526950538 |
| SPAG1 | 0.228902604 | 2.627765075 | 5.613906268 | 2.52E-08 | 4.01E-07 | 8.499663497 |
| CXCL1 | 0.490149688 | 2.793541207 | 5.61249816 | 2.54E-08 | 4.04E-07 | 8.492061355 |
| ARPC1B | 0.210067606 | 4.849896787 | 5.611957122 | 2.55E-08 | 4.04E-07 | 8.489140858 |
| TIMM8A | 0.151774809 | 2.663645628 | 5.611316166 | 2.56E-08 | 4.05E-07 | 8.485681347 |
| GAB3 | -0.146461947 | 2.029395573 | -5.61117837 | 2.56E-08 | 4.05E-07 | 8.484937665 |
| DHRS12 | -0.153938113 | 2.594473209 | -5.60600269 | 2.64E-08 | 4.16E-07 | 8.457016653 |
| LRRC42 | 0.158028805 | 3.874453577 | 5.60291766 | 2.68E-08 | 4.22E-07 | 8.440385504 |
| SPSB3 | -0.128518524 | 2.786930704 | -5.60285119 | 2.68E-08 | 4.22E-07 | 8.440027241 |
| MTDH | 0.173480087 | 4.458089461 | 5.602616488 | 2.69E-08 | 4.22E-07 | 8.438762375 |
| SSR4 | 0.204992237 | 4.765613342 | 5.600258965 | 2.72E-08 | 4.27E-07 | 8.426059625 |
| SORBS3 | -0.211173473 | 3.730503553 | -5.59908011 | 2.74E-08 | 4.30E-07 | 8.419709623 |
| FDPS | 0.132153685 | 4.6111806 | 5.598663472 | 2.75E-08 | 4.30E-07 | 8.41746569 |
| WFDC2 | 0.365549693 | 6.459002352 | 5.58981888 | 2.89E-08 | 4.51E-07 | 8.369867161 |
| S100A11 | 0.246797876 | 6.498363012 | 5.588832487 | 2.90E-08 | 4.53E-07 | 8.36456313 |
| DMD | -0.176866754 | 2.308638017 | -5.58843837 | 2.91E-08 | 4.53E-07 | 8.362444121 |
| FBLN1 | -0.302678292 | 4.034035911 | -5.5883616 | 2.91E-08 | 4.53E-07 | 8.362031374 |
| IER3IP1 | 0.179526193 | 3.497806367 | 5.586726245 | 2.94E-08 | 4.56E-07 | 8.353240407 |
| FAT4 | -0.138831315 | 2.121213903 | -5.58430743 | 2.98E-08 | 4.62E-07 | 8.340242306 |
| FRMD4A | -0.173491942 | 2.739050202 | -5.57909847 | 3.07E-08 | 4.75E-07 | 8.312268734 |
| PTGIS | -0.344252698 | 3.087504577 | -5.57546168 | 3.13E-08 | 4.84E-07 | 8.292752672 |
| SHPRH | -0.105398247 | 2.567213845 | -5.57491588 | 3.14E-08 | 4.84E-07 | 8.289824823 |
| AP2S1 | 0.184253029 | 4.391254476 | 5.574796079 | 3.14E-08 | 4.84E-07 | 8.2891822 |
| CXXC4 | -0.247681012 | 1.743782759 | -5.56600967 | 3.30E-08 | 5.07E-07 | 8.242086709 |
| PAQR8 | -0.217424193 | 2.543104171 | -5.56596255 | 3.30E-08 | 5.07E-07 | 8.24183435 |
| KANK2 | -0.180877975 | 3.702307224 | -5.56412859 | 3.33E-08 | 5.12E-07 | 8.232013165 |
| CHD4 | -0.122470355 | 4.819197406 | -5.55773827 | 3.45E-08 | 5.29E-07 | 8.197815721 |
| IPPK | 0.116296513 | 2.430028904 | 5.557618546 | 3.46E-08 | 5.29E-07 | 8.197175378 |
| TRAIP | 0.16964392 | 2.731075508 | 5.556912954 | 3.47E-08 | 5.31E-07 | 8.193401787 |
| RFC2 | 0.155992434 | 3.727005972 | 5.555795179 | 3.49E-08 | 5.33E-07 | 8.187424723 |
| MED30 | 0.176535538 | 3.48716181 | 5.551002586 | 3.59E-08 | 5.47E-07 | 8.161810192 |
| DIRAS2 | 0.289897872 | 1.841902361 | 5.54999981 | 3.61E-08 | 5.49E-07 | 8.156453386 |
| CLIP3 | -0.272546772 | 3.551548353 | -5.54981017 | 3.61E-08 | 5.49E-07 | 8.155440412 |
| SEMA6D | -0.206635811 | 2.58727829 | -5.5490346 | 3.62E-08 | 5.51E-07 | 8.151298106 |
| ADD1 | -0.135929934 | 4.148889488 | -5.54854196 | 3.63E-08 | 5.51E-07 | 8.148667232 |
| HSPA6 | 0.235105512 | 2.391608757 | 5.547332147 | 3.66E-08 | 5.54E-07 | 8.142207235 |
| TIMM10 | 0.183568697 | 4.033430497 | 5.547074611 | 3.66E-08 | 5.54E-07 | 8.140832252 |
| SRD5A3 | 0.254749919 | 3.385200055 | 5.546226003 | 3.68E-08 | 5.56E-07 | 8.136301973 |
| CAPN10 | -0.150941556 | 2.724843291 | -5.54406637 | 3.73E-08 | 5.62E-07 | 8.124775771 |
| FOXN3 | -0.144077544 | 3.136726625 | -5.54403394 | 3.73E-08 | 5.62E-07 | 8.124602684 |
| PLK4 | 0.181352945 | 2.605470065 | 5.543190045 | 3.74E-08 | 5.63E-07 | 8.12009992 |
| TMEM70 | 0.163980206 | 3.172360951 | 5.541389988 | 3.78E-08 | 5.68E-07 | 8.110497473 |
| KCNE3 | 0.241178684 | 3.060990622 | 5.540786515 | 3.79E-08 | 5.70E-07 | 8.10727889 |
| MRPS12 | 0.226428168 | 3.856655795 | 5.539716691 | 3.82E-08 | 5.72E-07 | 8.101573872 |
| ABHD11 | 0.232142268 | 4.462677343 | 5.539562386 | 3.82E-08 | 5.72E-07 | 8.100751096 |
| S100A8 | 0.502174687 | 2.540473369 | 5.53870039 | 3.84E-08 | 5.74E-07 | 8.096155231 |
| AXIN2 | -0.201775129 | 3.107981656 | -5.53679653 | 3.88E-08 | 5.79E-07 | 8.086006884 |
| CD1C | -0.270134156 | 1.473105262 | -5.5307107 | 4.01E-08 | 5.98E-07 | 8.05358907 |
| PCNA | 0.20199916 | 4.864288357 | 5.528556597 | 4.06E-08 | 6.05E-07 | 8.042122716 |
| OASL | 0.35211557 | 2.797947791 | 5.52818537 | 4.07E-08 | 6.05E-07 | 8.040147089 |
| CALCOCO1 | -0.166018322 | 3.898078061 | -5.52770268 | 4.08E-08 | 6.06E-07 | 8.037578466 |
| PHF14 | -0.153678086 | 3.357667556 | -5.52466264 | 4.15E-08 | 6.15E-07 | 8.021405767 |
| PDIA4 | 0.197929659 | 5.311487285 | 5.523473196 | 4.18E-08 | 6.18E-07 | 8.015080329 |
| SAR1B | 0.136502061 | 3.134448255 | 5.520554267 | 4.24E-08 | 6.27E-07 | 7.999562986 |
| RHOV | 0.30681685 | 2.341646623 | 5.520385013 | 4.25E-08 | 6.27E-07 | 7.998663448 |
| SPRY1 | -0.261548251 | 4.112994302 | -5.51558005 | 4.36E-08 | 6.44E-07 | 7.973137321 |
| PRR15 | 0.308320031 | 3.292677287 | 5.514762464 | 4.38E-08 | 6.46E-07 | 7.968796055 |
| SLC2A5 | 0.221277903 | 2.336553144 | 5.514362933 | 4.39E-08 | 6.46E-07 | 7.96667481 |
| C19orf44 | -0.180772777 | 2.742115938 | -5.51152912 | 4.46E-08 | 6.55E-07 | 7.951633282 |
| FKBP3 | 0.158536992 | 3.899376773 | 5.51006274 | 4.50E-08 | 6.60E-07 | 7.943852813 |
| PRRG4 | 0.230637571 | 2.917228972 | 5.509823944 | 4.50E-08 | 6.60E-07 | 7.942585969 |
| LSM4 | 0.194685942 | 4.711365993 | 5.509279804 | 4.52E-08 | 6.61E-07 | 7.939699421 |
| COX6B1 | 0.216830421 | 5.90431897 | 5.50893622 | 4.53E-08 | 6.61E-07 | 7.937876922 |
| PSMC1 | 0.13072601 | 3.573676775 | 5.506954603 | 4.58E-08 | 6.68E-07 | 7.927367763 |
| NDUFB5 | 0.172522475 | 4.360187528 | 5.506515346 | 4.59E-08 | 6.69E-07 | 7.925038725 |
| CRB3 | 0.205904913 | 3.45273098 | 5.506238506 | 4.59E-08 | 6.69E-07 | 7.923570947 |
| ACSS3 | -0.222564562 | 2.413659459 | -5.50390506 | 4.65E-08 | 6.76E-07 | 7.911202005 |
| GMNN | 0.227198981 | 3.387803352 | 5.503683527 | 4.66E-08 | 6.76E-07 | 7.910027998 |
| TXNDC17 | 0.168620891 | 3.453573324 | 5.501399495 | 4.72E-08 | 6.84E-07 | 7.897926256 |
| ATL3 | 0.143607012 | 3.925433576 | 5.500785718 | 4.73E-08 | 6.85E-07 | 7.894675019 |
| CLPTM1L | 0.16215468 | 4.234809249 | 5.499854412 | 4.76E-08 | 6.88E-07 | 7.889742459 |
| BBS9 | -0.128498786 | 2.663459936 | -5.4993357 | 4.77E-08 | 6.88E-07 | 7.886995514 |
| ANKRD44 | -0.143124969 | 2.266592161 | -5.49922334 | 4.78E-08 | 6.88E-07 | 7.886400484 |
| MPZL2 | 0.25075184 | 3.806615031 | 5.499109355 | 4.78E-08 | 6.88E-07 | 7.885796909 |
| CHST14 | -0.168837429 | 3.222519236 | -5.49727601 | 4.83E-08 | 6.94E-07 | 7.876090325 |
| LRG1 | 0.387628681 | 2.552507108 | 5.49501923 | 4.89E-08 | 7.02E-07 | 7.864146108 |
| PARVA | -0.147879353 | 3.680859896 | -5.49248424 | 4.96E-08 | 7.11E-07 | 7.850734969 |
| TC2N | 0.211394242 | 3.561385853 | 5.492303235 | 4.96E-08 | 7.11E-07 | 7.849777579 |
| LRP1 | -0.222809265 | 4.095463155 | -5.49099272 | 5.00E-08 | 7.15E-07 | 7.842846885 |
| PPIF | 0.181346533 | 4.484043555 | 5.490847669 | 5.00E-08 | 7.15E-07 | 7.842079901 |
| YIPF6 | 0.125439791 | 3.386984584 | 5.490194092 | 5.02E-08 | 7.16E-07 | 7.838624127 |
| PPP1CA | 0.160707519 | 5.043998392 | 5.483666062 | 5.20E-08 | 7.42E-07 | 7.804128597 |
| LIMD2 | -0.204537223 | 3.13701312 | -5.48328282 | 5.21E-08 | 7.42E-07 | 7.802104684 |
| BET1 | 0.145377547 | 3.22876396 | 5.483199303 | 5.22E-08 | 7.42E-07 | 7.801663626 |
| STARD4 | 0.17710819 | 2.49131152 | 5.481154773 | 5.28E-08 | 7.49E-07 | 7.790868729 |
| PRSS35 | -0.23851353 | 1.125197657 | -5.47870559 | 5.35E-08 | 7.58E-07 | 7.777942328 |
| TMED9 | 0.1691402 | 4.843991199 | 5.478034654 | 5.37E-08 | 7.60E-07 | 7.774402157 |
| TCF7L1 | -0.313515067 | 3.013649102 | -5.47646102 | 5.41E-08 | 7.66E-07 | 7.766100599 |
| THBS4 | -0.283229825 | 2.330123828 | -5.47608189 | 5.42E-08 | 7.66E-07 | 7.764100886 |
| TRMT12 | 0.169081112 | 3.502754831 | 5.474483661 | 5.47E-08 | 7.72E-07 | 7.755672413 |
| SHMT2 | 0.179884084 | 4.304463836 | 5.474377872 | 5.48E-08 | 7.72E-07 | 7.755114602 |
| NDUFB11 | 0.179774285 | 4.898622455 | 5.470438378 | 5.60E-08 | 7.87E-07 | 7.734349505 |
| SUV39H1 | 0.178868538 | 2.852532652 | 5.469887791 | 5.61E-08 | 7.89E-07 | 7.731448484 |
| EIF2B3 | 0.128066695 | 3.271001509 | 5.46833868 | 5.66E-08 | 7.94E-07 | 7.723287754 |
| GLRX5 | 0.149883223 | 4.141786628 | 5.467761973 | 5.68E-08 | 7.96E-07 | 7.720250211 |
| BLVRB | 0.218688177 | 3.980722945 | 5.465411159 | 5.75E-08 | 8.05E-07 | 7.707871513 |
| GPR160 | 0.31454934 | 2.881872576 | 5.464164782 | 5.79E-08 | 8.10E-07 | 7.701310492 |
| POLR3B | -0.124915732 | 2.671394944 | -5.4624248 | 5.85E-08 | 8.17E-07 | 7.692153453 |
| PTPN5 | -0.206800854 | 1.043675092 | -5.45630798 | 6.05E-08 | 8.43E-07 | 7.659984278 |
| ANTXR2 | -0.207382154 | 2.483660511 | -5.45459664 | 6.10E-08 | 8.50E-07 | 7.650990203 |
| SH3BGRL | -0.166942184 | 4.531856556 | -5.45027308 | 6.25E-08 | 8.69E-07 | 7.628279272 |
| TMEM59 | 0.162993204 | 4.494269159 | 5.449844477 | 6.26E-08 | 8.70E-07 | 7.626028838 |
| GINS4 | 0.172337957 | 2.495610497 | 5.448716452 | 6.30E-08 | 8.75E-07 | 7.620106756 |
| EBAG9 | 0.191354549 | 3.723595816 | 5.447954174 | 6.33E-08 | 8.77E-07 | 7.616105489 |
| HNRNPAB | 0.162379609 | 5.15068122 | 5.444931578 | 6.43E-08 | 8.91E-07 | 7.600244806 |
| STK33 | -0.181067197 | 2.393486443 | -5.4440111 | 6.47E-08 | 8.94E-07 | 7.595416377 |
| STAT5A | -0.175345316 | 3.172300387 | -5.43527584 | 6.78E-08 | 9.37E-07 | 7.549633299 |
| SLC3A2 | 0.158306374 | 4.375103761 | 5.427719222 | 7.07E-08 | 9.75E-07 | 7.510083808 |
| COMMD5 | 0.167657532 | 3.560164084 | 5.4247144 | 7.18E-08 | 9.89E-07 | 7.494371757 |
| SH3BP4 | -0.215470761 | 3.836063476 | -5.42459472 | 7.19E-08 | 9.89E-07 | 7.493746151 |
| NAMPT | 0.198961925 | 3.710168541 | 5.419068506 | 7.41E-08 | 1.02E-06 | 7.464871932 |
| GATM | -0.228524511 | 2.1800994 | -5.41834462 | 7.44E-08 | 1.02E-06 | 7.461091734 |
| EID1 | -0.16988654 | 4.486331922 | -5.41333954 | 7.64E-08 | 1.05E-06 | 7.434967743 |
| HERC1 | -0.116245625 | 2.949028473 | -5.41023321 | 7.77E-08 | 1.06E-06 | 7.418765801 |
| DNAJB5 | 0.232396507 | 2.791688349 | 5.410025625 | 7.78E-08 | 1.06E-06 | 7.417683371 |
| QPCT | 0.33770112 | 2.394545361 | 5.409088141 | 7.82E-08 | 1.07E-06 | 7.412795549 |
| OSMR | 0.227792565 | 3.457573606 | 5.407197051 | 7.90E-08 | 1.08E-06 | 7.402938297 |
| SDC4 | 0.212800434 | 5.082630707 | 5.40703243 | 7.91E-08 | 1.08E-06 | 7.402080369 |
| PEX13 | 0.12459772 | 3.242499569 | 5.404405318 | 8.02E-08 | 1.09E-06 | 7.388392427 |
| PLA2G4A | -0.28264293 | 2.484833911 | -5.39907909 | 8.26E-08 | 1.12E-06 | 7.360660711 |
| NEK9 | -0.14147999 | 3.286687864 | -5.38657535 | 8.84E-08 | 1.20E-06 | 7.295659916 |
| RFC3 | 0.18706838 | 3.39519168 | 5.384221685 | 8.95E-08 | 1.21E-06 | 7.283440333 |
| FCER1A | -0.260556564 | 1.271404063 | -5.38371018 | 8.98E-08 | 1.22E-06 | 7.280785424 |
| CNPY4 | -0.155932164 | 2.75086835 | -5.37003193 | 9.66E-08 | 1.31E-06 | 7.209878058 |
| NLRC3 | -0.162280339 | 1.960718825 | -5.36907746 | 9.71E-08 | 1.31E-06 | 7.204936541 |
| CREBBP | -0.154723957 | 3.846061247 | -5.36863206 | 9.74E-08 | 1.31E-06 | 7.202630873 |
| MCTS1 | 0.140659978 | 3.446893121 | 5.367190261 | 9.81E-08 | 1.32E-06 | 7.195168426 |
| ARHGAP6 | -0.168811976 | 1.726512378 | -5.36375649 | 1.00E-07 | 1.35E-06 | 7.177403659 |
| ADK | 0.174228431 | 3.330362329 | 5.362313502 | 1.01E-07 | 1.35E-06 | 7.169941502 |
| GSTA4 | -0.209108262 | 3.016824796 | -5.36055726 | 1.02E-07 | 1.37E-06 | 7.160861959 |
| NDUFA3 | 0.206254234 | 4.41898012 | 5.360334627 | 1.02E-07 | 1.37E-06 | 7.159711201 |
| ATP6V1E1 | 0.151697221 | 4.264513943 | 5.359612419 | 1.02E-07 | 1.37E-06 | 7.155978451 |
| TNF | 0.31921151 | 2.497630383 | 5.358745057 | 1.03E-07 | 1.37E-06 | 7.151496102 |
| S100A5 | 0.296598573 | 1.94777465 | 5.357939052 | 1.03E-07 | 1.38E-06 | 7.147331443 |
| AOAH | -0.225182378 | 2.467202969 | -5.35720611 | 1.04E-07 | 1.38E-06 | 7.143544809 |
| ATP6AP2 | 0.15459274 | 4.593532825 | 5.353516531 | 1.06E-07 | 1.41E-06 | 7.124490682 |
| GOLPH3 | 0.138447374 | 4.720997448 | 5.351169304 | 1.07E-07 | 1.42E-06 | 7.112375342 |
| ERMP1 | 0.202314923 | 3.572974896 | 5.351162759 | 1.07E-07 | 1.42E-06 | 7.112341564 |
| PDZRN3 | -0.237641818 | 2.829773841 | -5.35098583 | 1.07E-07 | 1.42E-06 | 7.111428534 |
| NDUFA4L2 | 0.346515396 | 3.254906665 | 5.35092289 | 1.07E-07 | 1.42E-06 | 7.111103753 |
| ANXA6 | -0.19350285 | 3.722298165 | -5.34988599 | 1.08E-07 | 1.43E-06 | 7.105753588 |
| SMG6 | -0.128238921 | 2.936893429 | -5.348984 | 1.08E-07 | 1.43E-06 | 7.101100279 |
| MDH2 | 0.169038611 | 5.039554681 | 5.347833074 | 1.09E-07 | 1.44E-06 | 7.09516386 |
| LYPLA1 | 0.17106704 | 4.16374207 | 5.347714322 | 1.09E-07 | 1.44E-06 | 7.09455141 |
| BBS4 | -0.135686031 | 2.924498092 | -5.346898 | 1.09E-07 | 1.44E-06 | 7.090341688 |
| UBE3C | 0.141034018 | 3.688211894 | 5.343985389 | 1.11E-07 | 1.46E-06 | 7.075326428 |
| KRT16 | 0.358811891 | 2.16486778 | 5.338876759 | 1.14E-07 | 1.50E-06 | 7.049008864 |
| MYT1 | -0.244924838 | 1.88740694 | -5.33883946 | 1.14E-07 | 1.50E-06 | 7.048816829 |
| ALAS1 | 0.127968872 | 4.000276117 | 5.336618834 | 1.16E-07 | 1.52E-06 | 7.037384562 |
| DISC1 | -0.116027271 | 2.092554128 | -5.33653806 | 1.16E-07 | 1.52E-06 | 7.036968807 |
| BID | 0.189491515 | 3.457984973 | 5.331768077 | 1.19E-07 | 1.55E-06 | 7.012427531 |
| TSEN34 | 0.148317334 | 3.857814429 | 5.331723528 | 1.19E-07 | 1.55E-06 | 7.012198429 |
| NAP1L3 | -0.245761655 | 1.845935766 | -5.33033502 | 1.20E-07 | 1.56E-06 | 7.005058585 |
| MBNL2 | -0.159615009 | 3.736620694 | -5.32713657 | 1.22E-07 | 1.59E-06 | 6.988618621 |
| SLC35A3 | 0.142075526 | 3.337384597 | 5.326854748 | 1.22E-07 | 1.59E-06 | 6.987170492 |
| GINS2 | 0.215753156 | 2.528230684 | 5.324850564 | 1.23E-07 | 1.60E-06 | 6.976874288 |
| GLRX2 | 0.173488401 | 2.848909685 | 5.321853518 | 1.25E-07 | 1.63E-06 | 6.961484253 |
| MRPL12 | 0.224600799 | 3.699232022 | 5.319324372 | 1.27E-07 | 1.65E-06 | 6.948503301 |
| PPP6C | 0.126153889 | 3.728277591 | 5.318045195 | 1.28E-07 | 1.66E-06 | 6.941940098 |
| CXADR | 0.205349088 | 3.723869853 | 5.316722617 | 1.29E-07 | 1.67E-06 | 6.935155784 |
| ZNF32 | -0.160422741 | 3.85419248 | -5.31640287 | 1.29E-07 | 1.67E-06 | 6.933515834 |
| REV3L | -0.14545117 | 2.881465136 | -5.30887195 | 1.34E-07 | 1.73E-06 | 6.894917827 |
| KLHL3 | -0.121073888 | 2.244682187 | -5.30535946 | 1.37E-07 | 1.77E-06 | 6.876933083 |
| TAOK1 | -0.119316566 | 3.217534967 | -5.30470242 | 1.37E-07 | 1.77E-06 | 6.873570127 |
| RAB37 | -0.184026371 | 1.334599852 | -5.30396505 | 1.38E-07 | 1.77E-06 | 6.869796512 |
| LRP6 | -0.183323346 | 3.513361492 | -5.29818647 | 1.42E-07 | 1.83E-06 | 6.840240838 |
| ADRA2A | -0.310539194 | 2.496876313 | -5.29560446 | 1.44E-07 | 1.85E-06 | 6.827044507 |
| SSR3 | 0.140290351 | 4.605446731 | 5.294400524 | 1.45E-07 | 1.86E-06 | 6.820893414 |
| MYO1D | -0.155745987 | 3.45100731 | -5.29405935 | 1.45E-07 | 1.86E-06 | 6.81915057 |
| IFITM1 | 0.313290928 | 5.859356683 | 5.289564169 | 1.49E-07 | 1.90E-06 | 6.796197142 |
| TUBG1 | 0.154251446 | 3.747984176 | 5.289262756 | 1.49E-07 | 1.91E-06 | 6.794658719 |
| LUC7L | -0.173677322 | 3.470591001 | -5.28803472 | 1.50E-07 | 1.92E-06 | 6.788391665 |
| RNF113A | 0.148414659 | 3.253572781 | 5.286788789 | 1.51E-07 | 1.93E-06 | 6.782034655 |
| ARHGAP22 | -0.128024705 | 2.176503046 | -5.28545678 | 1.52E-07 | 1.94E-06 | 6.775240046 |
| ABR | -0.194686908 | 3.015157886 | -5.28114079 | 1.56E-07 | 1.98E-06 | 6.753235252 |
| ROR1 | -0.230040795 | 2.642769671 | -5.28094739 | 1.56E-07 | 1.98E-06 | 6.752249625 |
| PLSCR1 | 0.226963966 | 4.290292743 | 5.279524694 | 1.57E-07 | 1.99E-06 | 6.745000083 |
| ALG6 | 0.134143247 | 3.00097748 | 5.276537582 | 1.59E-07 | 2.02E-06 | 6.729784889 |
| HINT2 | 0.193665568 | 3.56716604 | 5.276038125 | 1.60E-07 | 2.03E-06 | 6.727241645 |
| MAF1 | 0.182967472 | 5.109896077 | 5.273655408 | 1.62E-07 | 2.05E-06 | 6.715111948 |
| AOC3 | -0.246835932 | 2.158488561 | -5.27307397 | 1.62E-07 | 2.05E-06 | 6.712152788 |
| VAV3 | 0.377869337 | 2.814178203 | 5.271473228 | 1.64E-07 | 2.07E-06 | 6.704007685 |
| MAG | -0.354072053 | 1.867661876 | -5.26723291 | 1.68E-07 | 2.11E-06 | 6.682442802 |
| ATP10D | -0.146242238 | 2.525636966 | -5.26569434 | 1.69E-07 | 2.13E-06 | 6.674622259 |
| MAPK10 | -0.189142449 | 1.935128824 | -5.26421864 | 1.70E-07 | 2.14E-06 | 6.667123263 |
| TIGD5 | 0.196822884 | 3.10476697 | 5.263851971 | 1.71E-07 | 2.14E-06 | 6.66526028 |
| CAPS2 | -0.121694146 | 1.704816145 | -5.26364141 | 1.71E-07 | 2.14E-06 | 6.664190509 |
| PKMYT1 | 0.200592472 | 3.036396538 | 5.25833051 | 1.76E-07 | 2.20E-06 | 6.637221719 |
| ZNF367 | 0.194471292 | 2.543944984 | 5.252402564 | 1.81E-07 | 2.27E-06 | 6.607150068 |
| ATP8B2 | -0.190081299 | 3.059704315 | -5.24871431 | 1.85E-07 | 2.31E-06 | 6.588456302 |
| NUPR1 | 0.26305581 | 3.898970035 | 5.247893401 | 1.86E-07 | 2.32E-06 | 6.584297258 |
| RCE1 | 0.130749553 | 3.439985361 | 5.246631732 | 1.87E-07 | 2.33E-06 | 6.577906352 |
| SMOC2 | -0.288684295 | 3.117106951 | -5.24581849 | 1.88E-07 | 2.34E-06 | 6.573787689 |
| LTBP2 | -0.24903784 | 4.233612959 | -5.24454212 | 1.89E-07 | 2.35E-06 | 6.567324741 |
| MRPL17 | 0.140288652 | 3.838184672 | 5.242449621 | 1.91E-07 | 2.38E-06 | 6.55673255 |
| MAF | -0.201466862 | 2.920765739 | -5.23877897 | 1.95E-07 | 2.42E-06 | 6.53816147 |
| ZNF518A | -0.134049332 | 2.943268522 | -5.23801396 | 1.96E-07 | 2.43E-06 | 6.534292572 |
| CEP70 | 0.16484694 | 3.452881678 | 5.229917182 | 2.04E-07 | 2.53E-06 | 6.493377476 |
| COL16A1 | -0.276345563 | 3.363797669 | -5.2298144 | 2.04E-07 | 2.53E-06 | 6.492858458 |
| PDGFRB | -0.253338762 | 3.56877098 | -5.22966278 | 2.04E-07 | 2.53E-06 | 6.492092875 |
| LOXL3 | -0.152691291 | 2.624089174 | -5.22916944 | 2.05E-07 | 2.53E-06 | 6.489601985 |
| ZNF706 | 0.142447076 | 3.955874369 | 5.226194746 | 2.08E-07 | 2.57E-06 | 6.474587226 |
| GPLD1 | -0.14448227 | 1.659813782 | -5.22508877 | 2.09E-07 | 2.58E-06 | 6.469006908 |
| MRS2 | 0.158308412 | 3.539793586 | 5.224471164 | 2.10E-07 | 2.59E-06 | 6.465891161 |
| KCNMA1 | -0.213113302 | 2.8251362 | -5.22274 | 2.12E-07 | 2.61E-06 | 6.457159603 |
| AKT3 | -0.214396062 | 2.426464199 | -5.22144652 | 2.13E-07 | 2.62E-06 | 6.450637374 |
| GREM2 | -0.189807672 | 1.309558504 | -5.22013151 | 2.15E-07 | 2.64E-06 | 6.444008193 |
| FOSL1 | 0.241708002 | 2.075793951 | 5.217735678 | 2.18E-07 | 2.67E-06 | 6.431934454 |
| SYNGR2 | 0.196122852 | 4.793699778 | 5.217629466 | 2.18E-07 | 2.67E-06 | 6.43139932 |
| RPL8 | 0.214061264 | 7.205868831 | 5.217273092 | 2.18E-07 | 2.67E-06 | 6.429603868 |
| CD9 | 0.213142615 | 5.613548655 | 5.213650302 | 2.22E-07 | 2.72E-06 | 6.411358442 |
| DCUN1D1 | 0.146096158 | 3.334277894 | 5.212773267 | 2.23E-07 | 2.73E-06 | 6.406943249 |
| STK3 | 0.130259728 | 3.087745758 | 5.209353609 | 2.27E-07 | 2.77E-06 | 6.389734649 |
| PNMA3 | -0.292892745 | 2.547031645 | -5.2091948 | 2.28E-07 | 2.77E-06 | 6.38893575 |
| TSPAN12 | 0.303908068 | 3.435793175 | 5.206633476 | 2.31E-07 | 2.81E-06 | 6.376053899 |
| CCDC107 | 0.152873611 | 3.013820146 | 5.205628981 | 2.32E-07 | 2.82E-06 | 6.371003565 |
| ZNF462 | -0.176840535 | 2.846951688 | -5.20522266 | 2.32E-07 | 2.82E-06 | 6.368960934 |
| NFATC2 | -0.158539657 | 2.62510462 | -5.20359151 | 2.34E-07 | 2.84E-06 | 6.360762558 |
| NDUFA2 | 0.168690775 | 4.22567015 | 5.201425952 | 2.37E-07 | 2.87E-06 | 6.349881893 |
| CCDC80 | -0.329509479 | 3.53376517 | -5.20087446 | 2.38E-07 | 2.88E-06 | 6.347111642 |
| NELL2 | -0.220858365 | 2.071635614 | -5.19939997 | 2.40E-07 | 2.90E-06 | 6.339706445 |
| KPNA4 | 0.120139176 | 3.913665549 | 5.198707549 | 2.41E-07 | 2.90E-06 | 6.336229607 |
| TMEM130 | -0.274736864 | 1.984451097 | -5.19595277 | 2.44E-07 | 2.94E-06 | 6.322401542 |
| CCL20 | 0.452740633 | 2.324084839 | 5.195858592 | 2.44E-07 | 2.94E-06 | 6.321928936 |
| GIMAP8 | -0.158877548 | 2.101484747 | -5.19579243 | 2.44E-07 | 2.94E-06 | 6.321596903 |
| SRPRB | 0.128339377 | 3.671688984 | 5.193268107 | 2.47E-07 | 2.98E-06 | 6.308932155 |
| GSDMC | 0.255614845 | 2.065281867 | 5.19259168 | 2.48E-07 | 2.98E-06 | 6.305539456 |
| RLF | 0.15470515 | 3.307840452 | 5.192390028 | 2.49E-07 | 2.98E-06 | 6.304528128 |
| TERF2IP | -0.123971758 | 3.874945526 | -5.1917099 | 2.50E-07 | 2.99E-06 | 6.301117416 |
| RING1 | -0.143560407 | 4.322539819 | -5.19035109 | 2.51E-07 | 3.01E-06 | 6.294304521 |
| SLFN12 | -0.139163313 | 2.551533947 | -5.18980891 | 2.52E-07 | 3.01E-06 | 6.291586571 |
| DPYSL2 | -0.210130183 | 3.72555743 | -5.1867703 | 2.56E-07 | 3.06E-06 | 6.276358956 |
| FBN3 | -0.355255113 | 2.63399274 | -5.18427141 | 2.59E-07 | 3.10E-06 | 6.263842477 |
| P2RY6 | 0.229458851 | 2.514301495 | 5.184083486 | 2.60E-07 | 3.10E-06 | 6.262901423 |
| UCHL5 | 0.122744755 | 3.194465531 | 5.179233313 | 2.66E-07 | 3.17E-06 | 6.23862496 |
| USP28 | 0.165473909 | 3.475400799 | 5.179009608 | 2.67E-07 | 3.17E-06 | 6.23750577 |
| RTN1 | -0.202634284 | 2.086009171 | -5.17891415 | 2.67E-07 | 3.17E-06 | 6.237028199 |
| TRIM8 | -0.152693624 | 4.782896542 | -5.17535224 | 2.72E-07 | 3.23E-06 | 6.21921467 |
| CDR2L | 0.274329852 | 4.123676465 | 5.174434362 | 2.73E-07 | 3.24E-06 | 6.214626128 |
| NFIL3 | 0.186574339 | 3.506196341 | 5.173215421 | 2.75E-07 | 3.25E-06 | 6.208533753 |
| PSMD14 | 0.131213994 | 3.700790744 | 5.17317091 | 2.75E-07 | 3.25E-06 | 6.208311309 |
| NCBP1 | 0.125120022 | 3.25755633 | 5.171941401 | 2.77E-07 | 3.27E-06 | 6.202167548 |
| SPCS2 | 0.137542163 | 4.234751646 | 5.170547346 | 2.79E-07 | 3.29E-06 | 6.195203246 |
| MRPL14 | 0.168803633 | 4.486678947 | 5.168867867 | 2.81E-07 | 3.32E-06 | 6.186815412 |
| THOC7 | 0.140336112 | 4.203947584 | 5.168348483 | 2.82E-07 | 3.32E-06 | 6.184221977 |
| ATG4A | 0.141109269 | 2.649023521 | 5.168068744 | 2.82E-07 | 3.32E-06 | 6.182825257 |
| DRG1 | 0.151981715 | 4.16840388 | 5.165542558 | 2.86E-07 | 3.36E-06 | 6.170215441 |
| RPL39L | 0.218345785 | 3.935765134 | 5.164401033 | 2.88E-07 | 3.38E-06 | 6.164519283 |
| PRRG3 | -0.211542266 | 1.712550199 | -5.1623655 | 2.91E-07 | 3.41E-06 | 6.154365008 |
| DTNA | -0.208690237 | 2.226711193 | -5.1622303 | 2.91E-07 | 3.41E-06 | 6.153690713 |
| SLC28A3 | 0.261368501 | 2.408112107 | 5.16191503 | 2.92E-07 | 3.41E-06 | 6.152118372 |
| EFHC1 | -0.183756349 | 3.0670564 | -5.16176875 | 2.92E-07 | 3.41E-06 | 6.151388859 |
| MRPL11 | 0.162622391 | 4.138305946 | 5.159647355 | 2.95E-07 | 3.45E-06 | 6.1408115 |
| SLC9A6 | 0.135018441 | 3.024675422 | 5.158940641 | 2.96E-07 | 3.45E-06 | 6.137288711 |
| TMEM160 | 0.216569603 | 3.774586418 | 5.156245017 | 3.00E-07 | 3.50E-06 | 6.12385593 |
| UCHL3 | 0.183171769 | 2.875568886 | 5.155459281 | 3.02E-07 | 3.51E-06 | 6.119941722 |
| SENP7 | -0.137193116 | 2.926310666 | -5.15525794 | 3.02E-07 | 3.51E-06 | 6.118938813 |
| SLC25A27 | -0.196785843 | 2.723344866 | -5.1552312 | 3.02E-07 | 3.51E-06 | 6.118805642 |
| TCF19 | 0.203885788 | 3.318197981 | 5.154282931 | 3.03E-07 | 3.52E-06 | 6.114082701 |
| ISG15 | 0.395540519 | 5.149012525 | 5.151521135 | 3.08E-07 | 3.57E-06 | 6.100332073 |
| RGS9 | -0.220903368 | 1.830880644 | -5.15017976 | 3.10E-07 | 3.59E-06 | 6.093656071 |
| PURG | -0.183405608 | 1.38929656 | -5.14675387 | 3.15E-07 | 3.65E-06 | 6.076612941 |
| UBE2L6 | 0.221758493 | 4.830636015 | 5.146579896 | 3.16E-07 | 3.65E-06 | 6.075747769 |
| CLEC4F | -0.236812782 | 1.566000753 | -5.14519829 | 3.18E-07 | 3.67E-06 | 6.068877808 |
| CLEC2D | -0.142028828 | 2.448217576 | -5.14069333 | 3.26E-07 | 3.76E-06 | 6.046489406 |
| TNFRSF21 | 0.215796656 | 4.091412906 | 5.138561744 | 3.29E-07 | 3.79E-06 | 6.035902537 |
| CFL2 | 0.158722263 | 3.119706231 | 5.136598741 | 3.33E-07 | 3.83E-06 | 6.026156635 |
| PLAUR | 0.218163097 | 3.50253613 | 5.136414089 | 3.33E-07 | 3.83E-06 | 6.025240059 |
| ITFG2 | -0.153045241 | 3.421018347 | -5.13420378 | 3.37E-07 | 3.87E-06 | 6.014270925 |
| SMO | -0.217205963 | 3.546287441 | -5.13226664 | 3.40E-07 | 3.90E-06 | 6.004661184 |
| LSAMP | -0.188597295 | 1.956169449 | -5.13032322 | 3.44E-07 | 3.94E-06 | 5.995023722 |
| TRIM47 | 0.22154083 | 3.778304048 | 5.129697872 | 3.45E-07 | 3.95E-06 | 5.991923368 |
| CHMP2A | 0.148764686 | 4.503604733 | 5.126729431 | 3.50E-07 | 4.00E-06 | 5.977211234 |
| BCKDK | 0.137046758 | 3.875551775 | 5.126710467 | 3.50E-07 | 4.00E-06 | 5.977117271 |
| CSE1L | 0.145285834 | 4.641848403 | 5.12557177 | 3.52E-07 | 4.02E-06 | 5.971475851 |
| NEK3 | -0.151258059 | 2.492384154 | -5.12440748 | 3.54E-07 | 4.04E-06 | 5.965708868 |
| CCT5 | 0.157724359 | 4.757238889 | 5.123179589 | 3.57E-07 | 4.06E-06 | 5.959628209 |
| KDELR1 | 0.149227342 | 5.257102857 | 5.120580547 | 3.61E-07 | 4.11E-06 | 5.946762017 |
| CYBRD1 | -0.261793035 | 4.118424226 | -5.11951413 | 3.63E-07 | 4.13E-06 | 5.941484648 |
| SIVA1 | 0.171696155 | 3.835128457 | 5.118328835 | 3.66E-07 | 4.15E-06 | 5.935620257 |
| R3HDM2 | -0.117596651 | 3.674291856 | -5.11813876 | 3.66E-07 | 4.15E-06 | 5.934679965 |
| UBE2L3 | 0.134134419 | 4.190040952 | 5.117743751 | 3.67E-07 | 4.15E-06 | 5.932725948 |
| RRM1 | 0.163069881 | 3.642112972 | 5.117732945 | 3.67E-07 | 4.15E-06 | 5.932672496 |
| SLC10A3 | 0.171883333 | 3.93375784 | 5.1173971 | 3.67E-07 | 4.15E-06 | 5.931011278 |
| NAV3 | -0.139690648 | 2.000765634 | -5.11571826 | 3.71E-07 | 4.18E-06 | 5.922708641 |
| SMAD3 | -0.155026521 | 3.277992814 | -5.11565324 | 3.71E-07 | 4.18E-06 | 5.922387121 |
| GPBP1 | -0.122184859 | 3.948188113 | -5.11335072 | 3.75E-07 | 4.23E-06 | 5.911004442 |
| GCC2 | -0.111680019 | 3.145392994 | -5.11279937 | 3.76E-07 | 4.24E-06 | 5.908279531 |
| SPG11 | -0.117287856 | 3.37734097 | -5.11259603 | 3.77E-07 | 4.24E-06 | 5.907274658 |
| ITM2A | -0.270798141 | 2.289703857 | -5.11027199 | 3.81E-07 | 4.28E-06 | 5.895792124 |
| RAD1 | 0.125959707 | 3.191149677 | 5.109564923 | 3.83E-07 | 4.30E-06 | 5.892299687 |
| MICB | 0.246098069 | 2.588906822 | 5.106253839 | 3.89E-07 | 4.37E-06 | 5.875951173 |
| ASPRV1 | -0.174639642 | 1.78555034 | -5.10584512 | 3.90E-07 | 4.37E-06 | 5.8739338 |
| PACRG | -0.248285725 | 1.681471458 | -5.10569085 | 3.90E-07 | 4.37E-06 | 5.873172428 |
| PTPRB | -0.114201929 | 2.094284139 | -5.10345723 | 3.95E-07 | 4.41E-06 | 5.862150736 |
| JAM3 | -0.19234035 | 2.482608947 | -5.10336357 | 3.95E-07 | 4.41E-06 | 5.8616887 |
| THBS3 | -0.199822768 | 3.927099843 | -5.10034369 | 4.01E-07 | 4.48E-06 | 5.846794812 |
| RNASEH1 | 0.126112619 | 3.230970337 | 5.099005221 | 4.04E-07 | 4.50E-06 | 5.840196261 |
| LAYN | -0.217178039 | 2.644738702 | -5.0961778 | 4.10E-07 | 4.57E-06 | 5.826262677 |
| VASH2 | -0.166290801 | 2.02869479 | -5.09460508 | 4.13E-07 | 4.60E-06 | 5.818515473 |
| KLF12 | -0.14706995 | 2.626495903 | -5.09354539 | 4.15E-07 | 4.62E-06 | 5.813296754 |
| RBM18 | 0.136377665 | 3.045592601 | 5.087370889 | 4.29E-07 | 4.76E-06 | 5.782909216 |
| MEF2C | -0.158581043 | 2.547705046 | -5.0857583 | 4.32E-07 | 4.80E-06 | 5.774978717 |
| LSM3 | 0.148392992 | 3.792303569 | 5.085521166 | 4.33E-07 | 4.80E-06 | 5.773812725 |
| CDH11 | -0.305240463 | 3.318903762 | -5.07900334 | 4.48E-07 | 4.96E-06 | 5.741784729 |
| C9orf40 | 0.192243603 | 2.496787328 | 5.075556775 | 4.56E-07 | 5.04E-06 | 5.724864424 |
| PIK3R3 | 0.223444275 | 3.339107909 | 5.074510771 | 4.58E-07 | 5.06E-06 | 5.719731419 |
| HSPE1 | 0.172861398 | 4.750573813 | 5.074341865 | 4.59E-07 | 5.06E-06 | 5.718902646 |
| RUNX1T1 | -0.1917391 | 2.035596996 | -5.07314952 | 4.61E-07 | 5.09E-06 | 5.713052902 |
| B4GALT1 | 0.200664526 | 4.383254245 | 5.072970273 | 4.62E-07 | 5.09E-06 | 5.712173637 |
| FAM110B | -0.202251802 | 2.547142078 | -5.07265862 | 4.63E-07 | 5.09E-06 | 5.710644897 |
| SUCLG1 | 0.117562643 | 4.028120815 | 5.071519571 | 4.65E-07 | 5.12E-06 | 5.705058398 |
| FTSJ1 | 0.148742141 | 3.849851016 | 5.070300104 | 4.68E-07 | 5.14E-06 | 5.699078795 |
| SECTM1 | 0.308697351 | 3.285151144 | 5.068393658 | 4.73E-07 | 5.19E-06 | 5.689733367 |
| IPMK | 0.123012064 | 2.4227953 | 5.068239199 | 4.73E-07 | 5.19E-06 | 5.688976349 |
| APBB1 | -0.174864429 | 2.689126547 | -5.06685011 | 4.77E-07 | 5.22E-06 | 5.682169336 |
| CPVL | -0.257511421 | 3.335008395 | -5.06384187 | 4.84E-07 | 5.30E-06 | 5.667433978 |
| TYRO3 | -0.209225316 | 2.480236523 | -5.06336407 | 4.85E-07 | 5.30E-06 | 5.665094313 |
| CCDC124 | 0.157973343 | 4.381496488 | 5.063132263 | 4.86E-07 | 5.30E-06 | 5.663959281 |
| PDCD6 | 0.137897954 | 4.100075444 | 5.061758282 | 4.89E-07 | 5.34E-06 | 5.657232739 |
| GOSR1 | -0.096580289 | 3.190798494 | -5.06042285 | 4.93E-07 | 5.37E-06 | 5.650696589 |
| GAS6 | -0.260987842 | 4.919746934 | -5.05907648 | 4.96E-07 | 5.40E-06 | 5.644108579 |
| BCHE | -0.265856013 | 1.503180513 | -5.05856297 | 4.97E-07 | 5.41E-06 | 5.641596297 |
| WDR35 | -0.146653259 | 2.811789345 | -5.05823751 | 4.98E-07 | 5.41E-06 | 5.640004176 |
| ARF1 | 0.128245449 | 5.504991754 | 5.057674495 | 5.00E-07 | 5.42E-06 | 5.63725017 |
| TTF2 | 0.140338172 | 2.841818202 | 5.053497662 | 5.10E-07 | 5.53E-06 | 5.616828202 |
| ZNF287 | -0.111935268 | 2.096170552 | -5.05255618 | 5.13E-07 | 5.56E-06 | 5.612227206 |
| PLD4 | -0.226567588 | 1.823661612 | -5.05126934 | 5.16E-07 | 5.59E-06 | 5.60593973 |
| PFN1 | 0.160067989 | 6.317998385 | 5.049482519 | 5.21E-07 | 5.63E-06 | 5.597211948 |
| GLI1 | -0.229941541 | 1.41027139 | -5.04935168 | 5.21E-07 | 5.63E-06 | 5.59657299 |
| IMPG2 | -0.327310991 | 2.152495295 | -5.04872471 | 5.23E-07 | 5.64E-06 | 5.59351129 |
| LAMP3 | 0.31114393 | 3.005761412 | 5.047028543 | 5.27E-07 | 5.69E-06 | 5.585230193 |
| DCXR | 0.200875123 | 4.303656237 | 5.045321524 | 5.32E-07 | 5.73E-06 | 5.576898797 |
| LMAN2 | 0.147643786 | 4.700796532 | 5.045202365 | 5.32E-07 | 5.73E-06 | 5.57631732 |
| FGF2 | -0.187886945 | 1.59112287 | -5.04317252 | 5.38E-07 | 5.78E-06 | 5.566414016 |
| LMOD1 | -0.234289384 | 2.556464048 | -5.04203849 | 5.41E-07 | 5.81E-06 | 5.560882921 |
| PUF60 | 0.182932132 | 5.161119521 | 5.040697452 | 5.45E-07 | 5.85E-06 | 5.554343712 |
| PARS2 | 0.140918006 | 3.167570661 | 5.03739725 | 5.54E-07 | 5.94E-06 | 5.538258191 |
| TTC39B | 0.150162768 | 2.513652047 | 5.0357666 | 5.59E-07 | 5.98E-06 | 5.530313942 |
| DDIT3 | 0.185268047 | 3.937722416 | 5.035457642 | 5.60E-07 | 5.99E-06 | 5.528809025 |
| ABLIM1 | -0.218130339 | 4.297472929 | -5.03434568 | 5.63E-07 | 6.01E-06 | 5.523393443 |
| CHEK1 | 0.187989559 | 2.920738351 | 5.031192789 | 5.72E-07 | 6.11E-06 | 5.508044193 |
| MESP1 | 0.306049161 | 2.092443285 | 5.028889121 | 5.79E-07 | 6.17E-06 | 5.496835007 |
| CDON | -0.166505185 | 2.605475458 | -5.02627005 | 5.86E-07 | 6.25E-06 | 5.484097081 |
| POGZ | -0.146290785 | 3.952982566 | -5.02541632 | 5.89E-07 | 6.27E-06 | 5.479946308 |
| ZCCHC17 | 0.13617914 | 4.045537666 | 5.025008947 | 5.90E-07 | 6.28E-06 | 5.47796592 |
| RGMA | -0.218405395 | 2.668593349 | -5.02406315 | 5.93E-07 | 6.30E-06 | 5.473368657 |
| TAF1C | -0.142661598 | 3.355203701 | -5.02037262 | 6.04E-07 | 6.41E-06 | 5.455437885 |
| UBE2W | 0.129990835 | 3.084401916 | 5.019211115 | 6.08E-07 | 6.45E-06 | 5.449797226 |
| RHOBTB3 | -0.202191594 | 2.671023748 | -5.01724171 | 6.14E-07 | 6.50E-06 | 5.440235952 |
| ANO4 | -0.197130767 | 1.185552728 | -5.01504164 | 6.21E-07 | 6.57E-06 | 5.42955906 |
| BLM | 0.169335956 | 2.567330396 | 5.014902025 | 6.21E-07 | 6.57E-06 | 5.428881652 |
| CRIM1 | -0.168128161 | 3.718086142 | -5.01392286 | 6.24E-07 | 6.59E-06 | 5.424131339 |
| LACTB | 0.136846653 | 2.697094326 | 5.013793712 | 6.25E-07 | 6.59E-06 | 5.423504864 |
| ULK2 | -0.131606505 | 2.634765308 | -5.01364747 | 6.25E-07 | 6.59E-06 | 5.422795457 |
| DNAJB11 | 0.147281363 | 4.190905841 | 5.0120796 | 6.30E-07 | 6.64E-06 | 5.415191371 |
| ADAMTSL4 | -0.164955503 | 2.441252744 | -5.01122598 | 6.33E-07 | 6.66E-06 | 5.411052302 |
| SGPP2 | 0.25607978 | 3.393683062 | 5.009467895 | 6.39E-07 | 6.72E-06 | 5.402529746 |
| PORCN | 0.239374126 | 3.077592699 | 5.00738736 | 6.45E-07 | 6.78E-06 | 5.392447746 |
| LATS2 | -0.154215477 | 2.975233485 | -5.00655117 | 6.48E-07 | 6.80E-06 | 5.388396795 |
| THEM4 | -0.134534189 | 3.018872858 | -5.00596643 | 6.50E-07 | 6.82E-06 | 5.385564402 |
| MADD | -0.131988166 | 3.295167702 | -5.00454631 | 6.55E-07 | 6.85E-06 | 5.37868685 |
| CEBPG | 0.179895007 | 3.678452212 | 5.004303688 | 6.56E-07 | 6.85E-06 | 5.37751203 |
| EGR3 | -0.309947806 | 2.402180082 | -5.00429067 | 6.56E-07 | 6.85E-06 | 5.377448977 |
| SEMA4C | -0.156631426 | 3.882420552 | -5.00315798 | 6.59E-07 | 6.88E-06 | 5.37196504 |
| SYAP1 | 0.138342765 | 3.754066376 | 5.00307555 | 6.60E-07 | 6.88E-06 | 5.371566006 |
| CSF1R | -0.276817053 | 3.636063849 | -5.00290713 | 6.60E-07 | 6.88E-06 | 5.370750707 |
| GPR34 | -0.251718353 | 2.514470563 | -5.00067132 | 6.68E-07 | 6.96E-06 | 5.359929942 |
| FUT10 | -0.12522953 | 2.321915377 | -4.99784681 | 6.77E-07 | 7.05E-06 | 5.346266639 |
| LRRC59 | 0.144999357 | 4.106292039 | 4.997749294 | 6.78E-07 | 7.05E-06 | 5.345795063 |
| TFDP1 | 0.168838881 | 4.217190202 | 4.9974335 | 6.79E-07 | 7.05E-06 | 5.344267922 |
| GLT8D2 | -0.256362729 | 2.703942019 | -4.99665706 | 6.82E-07 | 7.07E-06 | 5.340513548 |
| OLFML1 | -0.222801508 | 2.496350815 | -4.99619432 | 6.83E-07 | 7.08E-06 | 5.338276323 |
| SLC22A17 | -0.241019657 | 2.79647886 | -4.99496315 | 6.87E-07 | 7.11E-06 | 5.332324816 |
| DEPDC1B | 0.235542401 | 2.595602484 | 4.994901349 | 6.88E-07 | 7.11E-06 | 5.332026098 |
| ISCA1 | 0.137387472 | 3.558748649 | 4.993411814 | 6.93E-07 | 7.16E-06 | 5.324827623 |
| SPR | 0.193767421 | 3.679573994 | 4.991936879 | 6.98E-07 | 7.21E-06 | 5.317701725 |
| MFNG | -0.179789103 | 2.363677178 | -4.99061985 | 7.03E-07 | 7.25E-06 | 5.311340437 |
| CP | 0.382825086 | 4.792483521 | 4.990432501 | 7.03E-07 | 7.25E-06 | 5.310435643 |
| UBE2V2 | 0.121729854 | 3.514430105 | 4.988776784 | 7.09E-07 | 7.30E-06 | 5.302441017 |
| PDZRN4 | -0.201010595 | 1.359527515 | -4.98876795 | 7.09E-07 | 7.30E-06 | 5.302398381 |
| CLGN | 0.399281057 | 2.919552762 | 4.986989254 | 7.16E-07 | 7.36E-06 | 5.293812773 |
| PIR | 0.25231316 | 2.569820407 | 4.985165976 | 7.22E-07 | 7.42E-06 | 5.285015012 |
| DEXI | -0.120654667 | 2.778573207 | -4.98434074 | 7.25E-07 | 7.44E-06 | 5.281034066 |
| ZNF407 | -0.088893638 | 2.394144341 | -4.98287759 | 7.31E-07 | 7.49E-06 | 5.273977344 |
| RTN2 | 0.220430334 | 2.635055672 | 4.98236633 | 7.33E-07 | 7.50E-06 | 5.271512015 |
| PIGB | -0.122232405 | 2.606732317 | -4.98202926 | 7.34E-07 | 7.51E-06 | 5.269886773 |
| GFRA1 | -0.239226655 | 1.964129889 | -4.9812923 | 7.37E-07 | 7.53E-06 | 5.266333782 |
| PLCL1 | -0.142162329 | 1.852021183 | -4.9809861 | 7.38E-07 | 7.53E-06 | 5.264857699 |
| CHAF1B | 0.180045496 | 2.714455683 | 4.97930061 | 7.44E-07 | 7.59E-06 | 5.256734014 |
| YWHAG | 0.116444943 | 4.804674787 | 4.978741479 | 7.46E-07 | 7.61E-06 | 5.254039713 |
| RPS23 | -0.173336098 | 5.624836589 | -4.97819796 | 7.48E-07 | 7.62E-06 | 5.251420936 |
| ATM | -0.111098281 | 2.963939305 | -4.97779945 | 7.50E-07 | 7.63E-06 | 5.249501008 |
| KLK11 | 0.408802461 | 3.906856364 | 4.977397992 | 7.51E-07 | 7.64E-06 | 5.247566995 |
| POLL | -0.127597843 | 3.378052374 | -4.97427801 | 7.63E-07 | 7.75E-06 | 5.232541824 |
| CLINT1 | 0.138792437 | 4.148746428 | 4.972561994 | 7.70E-07 | 7.81E-06 | 5.224281682 |
| ZNF438 | -0.102402883 | 2.659037765 | -4.97233361 | 7.71E-07 | 7.81E-06 | 5.223182546 |
| PPIA | 0.133579027 | 5.728613589 | 4.969478976 | 7.82E-07 | 7.92E-06 | 5.209448233 |
| FBLN5 | -0.262037467 | 3.517410137 | -4.96803431 | 7.88E-07 | 7.97E-06 | 5.202500495 |
| TST | 0.197651073 | 3.503559085 | 4.966673758 | 7.93E-07 | 8.02E-06 | 5.195958997 |
| MRPL40 | 0.166887916 | 3.725401442 | 4.964038644 | 8.04E-07 | 8.11E-06 | 5.183294351 |
| EMILIN1 | -0.290903977 | 3.583054983 | -4.96399249 | 8.04E-07 | 8.11E-06 | 5.183072591 |
| CLDN10 | 0.457000261 | 3.168676451 | 4.963652047 | 8.05E-07 | 8.12E-06 | 5.181436867 |
| MICAL1 | -0.16520839 | 2.868990916 | -4.96098018 | 8.16E-07 | 8.22E-06 | 5.168603076 |
| KCNAB3 | -0.137719846 | 1.954712353 | -4.95936981 | 8.23E-07 | 8.28E-06 | 5.160871153 |
| PSMC3 | 0.145047664 | 4.50595305 | 4.955556975 | 8.39E-07 | 8.43E-06 | 5.142574071 |
| CHMP5 | 0.150886864 | 4.307496258 | 4.953347449 | 8.48E-07 | 8.52E-06 | 5.131977103 |
| RAD18 | 0.134915868 | 2.808529641 | 4.950517306 | 8.60E-07 | 8.63E-06 | 5.118410226 |
| CMTM3 | -0.167166938 | 3.685056481 | -4.95034796 | 8.61E-07 | 8.63E-06 | 5.117598656 |
| ZWILCH | 0.143564221 | 2.980092709 | 4.945562188 | 8.82E-07 | 8.83E-06 | 5.094674656 |
| CUL9 | -0.155805636 | 3.138651224 | -4.9432174 | 8.92E-07 | 8.93E-06 | 5.083450778 |
| PATL1 | 0.130033136 | 3.923578511 | 4.943010328 | 8.93E-07 | 8.93E-06 | 5.082459815 |
| TCF7L2 | -0.138085233 | 3.664076727 | -4.93797708 | 9.16E-07 | 9.15E-06 | 5.058385094 |
| KLK6 | 0.407546575 | 4.813333225 | 4.934768172 | 9.31E-07 | 9.29E-06 | 5.043048676 |
| LAMB1 | -0.232203827 | 3.651423164 | -4.9320823 | 9.43E-07 | 9.41E-06 | 5.030219313 |
| CEP72 | 0.161611522 | 2.814928101 | 4.930727642 | 9.50E-07 | 9.46E-06 | 5.023751192 |
| UTP23 | 0.143853603 | 3.279907472 | 4.929870814 | 9.54E-07 | 9.49E-06 | 5.019660942 |
| RFTN2 | -0.147450556 | 1.73327773 | -4.92975141 | 9.54E-07 | 9.49E-06 | 5.019090993 |
| ORAI3 | -0.151945164 | 3.141074592 | -4.92888693 | 9.59E-07 | 9.53E-06 | 5.014964988 |
| TXNDC15 | -0.114910392 | 3.506608099 | -4.92671576 | 9.69E-07 | 9.62E-06 | 5.004605446 |
| TOMM22 | 0.147908824 | 4.157024946 | 4.926526983 | 9.70E-07 | 9.62E-06 | 5.003704943 |
| SIAH2 | 0.159682321 | 4.006445423 | 4.926213831 | 9.72E-07 | 9.63E-06 | 5.002211185 |
| MNT | -0.104578484 | 3.29349371 | -4.92372265 | 9.84E-07 | 9.74E-06 | 4.990331263 |
| B3GNT9 | -0.190082002 | 2.805191886 | -4.92310911 | 9.87E-07 | 9.76E-06 | 4.987406311 |
| RASAL2 | -0.116866406 | 2.789007849 | -4.9191564 | 1.01E-06 | 9.95E-06 | 4.968570708 |
| CNTN4 | -0.216688875 | 1.691832322 | -4.91620526 | 1.02E-06 | 1.01E-05 | 4.954517275 |
| GINS3 | 0.154355212 | 2.257850026 | 4.915888722 | 1.02E-06 | 1.01E-05 | 4.953010363 |
| PGRMC1 | 0.156407159 | 5.048190753 | 4.915404327 | 1.03E-06 | 1.01E-05 | 4.950704564 |
| NDUFAB1 | 0.145889544 | 4.529675641 | 4.913337449 | 1.04E-06 | 1.02E-05 | 4.940868324 |
| SLC39A13 | -0.121895288 | 3.80354696 | -4.91203258 | 1.04E-06 | 1.03E-05 | 4.934660488 |
| ST8SIA1 | -0.135547334 | 1.736601182 | -4.90973437 | 1.05E-06 | 1.04E-05 | 4.923730758 |
| PKIA | -0.256174641 | 1.888017939 | -4.90822832 | 1.06E-06 | 1.04E-05 | 4.91657103 |
| DUSP3 | 0.128193382 | 3.610420849 | 4.908220542 | 1.06E-06 | 1.04E-05 | 4.916534045 |
| BPTF | -0.128333317 | 3.717705226 | -4.90523478 | 1.08E-06 | 1.06E-05 | 4.902345972 |
| S100A1 | 0.435934645 | 4.834476991 | 4.903374807 | 1.09E-06 | 1.07E-05 | 4.893511702 |
| CAPN6 | -0.363659159 | 1.699156928 | -4.90047512 | 1.10E-06 | 1.08E-05 | 4.879745522 |
| P2RY13 | -0.219959834 | 1.778789522 | -4.89807288 | 1.12E-06 | 1.09E-05 | 4.868346832 |
| SFRP4 | -0.423545396 | 3.479465667 | -4.89806567 | 1.12E-06 | 1.09E-05 | 4.868312642 |
| SEMA6A | -0.210257449 | 2.280750867 | -4.8959547 | 1.13E-06 | 1.10E-05 | 4.858300496 |
| TRIM4 | -0.168654026 | 3.045729679 | -4.89515063 | 1.13E-06 | 1.11E-05 | 4.854487919 |
| GADD45GIP1 | 0.180521939 | 4.299971001 | 4.895117242 | 1.13E-06 | 1.11E-05 | 4.854329623 |
| TOMM40 | 0.150738909 | 4.109709228 | 4.892641349 | 1.15E-06 | 1.12E-05 | 4.84259383 |
| COPE | 0.147164502 | 4.808107957 | 4.8926361 | 1.15E-06 | 1.12E-05 | 4.842568958 |
| PCK2 | 0.160956103 | 3.190001544 | 4.89168162 | 1.15E-06 | 1.12E-05 | 4.838046217 |
| YRDC | 0.155677796 | 3.268668111 | 4.88705304 | 1.18E-06 | 1.15E-05 | 4.81612596 |
| GPAA1 | 0.169822307 | 4.878519072 | 4.885937574 | 1.19E-06 | 1.15E-05 | 4.810846248 |
| RHBDL2 | 0.191715883 | 2.253281249 | 4.885120893 | 1.19E-06 | 1.16E-05 | 4.806981469 |
| E2F1 | 0.199572021 | 3.692791219 | 4.885062431 | 1.19E-06 | 1.16E-05 | 4.806704835 |
| MRPL22 | 0.137873492 | 3.303785826 | 4.884412054 | 1.20E-06 | 1.16E-05 | 4.803627537 |
| CA2 | 0.260572178 | 2.572025906 | 4.883659207 | 1.20E-06 | 1.16E-05 | 4.80006588 |
| CD1D | -0.177941417 | 1.654689281 | -4.88177155 | 1.21E-06 | 1.17E-05 | 4.791137842 |
| ZC3H13 | -0.124935909 | 3.659418567 | -4.88065167 | 1.22E-06 | 1.18E-05 | 4.785842729 |
| NAP1L2 | -0.20563614 | 1.543386997 | -4.8805474 | 1.22E-06 | 1.18E-05 | 4.785349778 |
| FAM117A | -0.135722649 | 3.318673168 | -4.87980765 | 1.22E-06 | 1.18E-05 | 4.781852698 |
| CENPF | 0.222180428 | 3.564006553 | 4.879097956 | 1.23E-06 | 1.18E-05 | 4.778498217 |
| PSMD12 | 0.122868744 | 3.661337737 | 4.875166354 | 1.25E-06 | 1.20E-05 | 4.759923274 |
| MAGI2 | -0.133056233 | 2.159292864 | -4.87374126 | 1.26E-06 | 1.21E-05 | 4.753193912 |
| CCDC92 | -0.131245773 | 3.540611972 | -4.8712654 | 1.28E-06 | 1.23E-05 | 4.74150726 |
| NDUFV2 | 0.14742821 | 3.565819552 | 4.871133922 | 1.28E-06 | 1.23E-05 | 4.740886827 |
| GULP1 | -0.187684209 | 2.2012447 | -4.87097492 | 1.28E-06 | 1.23E-05 | 4.740136516 |
| KLC4 | -0.140034794 | 3.177271455 | -4.87086995 | 1.28E-06 | 1.23E-05 | 4.739641172 |
| MRPL15 | 0.155598846 | 4.678851631 | 4.866868119 | 1.31E-06 | 1.25E-05 | 4.720765064 |
| SEH1L | 0.126086847 | 3.293742755 | 4.866819522 | 1.31E-06 | 1.25E-05 | 4.72053593 |
| ATF7IP2 | -0.16454058 | 2.916861797 | -4.8641313 | 1.32E-06 | 1.26E-05 | 4.707864373 |
| RNF122 | -0.171831908 | 3.113648808 | -4.86336533 | 1.33E-06 | 1.27E-05 | 4.704255057 |
| DNAJC19 | 0.152787183 | 3.910379165 | 4.86252037 | 1.33E-06 | 1.27E-05 | 4.700274106 |
| SNCA | -0.202833771 | 1.93823161 | -4.86015828 | 1.35E-06 | 1.29E-05 | 4.689148908 |
| FMR1 | 0.163562945 | 4.022260762 | 4.859351533 | 1.35E-06 | 1.29E-05 | 4.685350396 |
| STYX | 0.109240566 | 2.915187286 | 4.859003026 | 1.36E-06 | 1.29E-05 | 4.683709662 |
| DDR2 | -0.206088878 | 2.811269193 | -4.85537416 | 1.38E-06 | 1.31E-05 | 4.666632047 |
| HOOK1 | 0.148563392 | 3.117956445 | 4.854377154 | 1.39E-06 | 1.32E-05 | 4.661942207 |
| PTH1R | -0.192261892 | 1.71679377 | -4.85404171 | 1.39E-06 | 1.32E-05 | 4.660364536 |
| CDKN2C | 0.290672567 | 3.067785844 | 4.8520505 | 1.40E-06 | 1.33E-05 | 4.651001421 |
| TRIM23 | -0.107302962 | 2.408244508 | -4.85170875 | 1.41E-06 | 1.33E-05 | 4.649394828 |
| S100P | 0.292939921 | 1.31011041 | 4.851692442 | 1.41E-06 | 1.33E-05 | 4.649318148 |
| CNNM3 | -0.130669401 | 3.46424552 | -4.85146037 | 1.41E-06 | 1.33E-05 | 4.648227232 |
| C7orf26 | -0.128878483 | 3.465292013 | -4.85106275 | 1.41E-06 | 1.33E-05 | 4.646358156 |
| FANCB | 0.125163638 | 1.965488146 | 4.850489587 | 1.42E-06 | 1.33E-05 | 4.643664244 |
| SELE | -0.197667256 | 1.223493808 | -4.85046637 | 1.42E-06 | 1.33E-05 | 4.643555107 |
| ATP8B4 | -0.128702006 | 1.856419886 | -4.84985192 | 1.42E-06 | 1.34E-05 | 4.640667492 |
| VKORC1L1 | 0.134980095 | 3.650676939 | 4.849836781 | 1.42E-06 | 1.34E-05 | 4.640596355 |
| TJP2 | 0.154614087 | 3.680615142 | 4.849376439 | 1.42E-06 | 1.34E-05 | 4.638433193 |
| AMMECR1L | -0.109860975 | 3.26490174 | -4.84885238 | 1.43E-06 | 1.34E-05 | 4.635970877 |
| SMARCA1 | -0.17820687 | 3.458263442 | -4.84853783 | 1.43E-06 | 1.34E-05 | 4.634493039 |
| SLC39A8 | 0.225506173 | 3.090255872 | 4.847155429 | 1.44E-06 | 1.35E-05 | 4.627999369 |
| TEK | -0.150212439 | 1.945657409 | -4.84642167 | 1.44E-06 | 1.35E-05 | 4.624553318 |
| RAB9A | 0.157775341 | 3.543333436 | 4.846223174 | 1.45E-06 | 1.35E-05 | 4.623621196 |
| KIT | -0.214209968 | 1.654166897 | -4.84541684 | 1.45E-06 | 1.36E-05 | 4.619835047 |
| MAPKBP1 | -0.122833537 | 2.745082451 | -4.84502399 | 1.45E-06 | 1.36E-05 | 4.617990621 |
| ANKRD29 | -0.19408379 | 1.49557881 | -4.84429762 | 1.46E-06 | 1.36E-05 | 4.614580724 |
| CTNS | -0.130733654 | 2.852411081 | -4.84294448 | 1.47E-06 | 1.37E-05 | 4.608229778 |
| CAV1 | -0.238156844 | 3.297684654 | -4.84139398 | 1.48E-06 | 1.38E-05 | 4.600954636 |
| CHI3L1 | 0.347174886 | 4.893341083 | 4.840789208 | 1.48E-06 | 1.38E-05 | 4.598117565 |
| UBE4B | -0.14235888 | 3.71830913 | -4.83907253 | 1.50E-06 | 1.39E-05 | 4.590066256 |
| TMCC2 | -0.190796831 | 2.553222225 | -4.8373572 | 1.51E-06 | 1.40E-05 | 4.582023948 |
| DDX19B | -0.101435473 | 2.724469774 | -4.83233539 | 1.55E-06 | 1.44E-05 | 4.558495031 |
| CDC7 | 0.202978704 | 2.86232896 | 4.830921608 | 1.56E-06 | 1.45E-05 | 4.551875208 |
| CFB | 0.316473954 | 4.044504648 | 4.830367912 | 1.56E-06 | 1.45E-05 | 4.549283107 |
| INSIG2 | 0.127837561 | 3.043916043 | 4.828410037 | 1.58E-06 | 1.46E-05 | 4.540119685 |
| PSMD6 | 0.102210704 | 3.450220418 | 4.827414063 | 1.59E-06 | 1.47E-05 | 4.535459606 |
| LDB3 | -0.146056041 | 1.564439971 | -4.82733301 | 1.59E-06 | 1.47E-05 | 4.535080395 |
| SUPT6H | -0.135348985 | 3.704694171 | -4.82405078 | 1.61E-06 | 1.49E-05 | 4.519729877 |
| FBXO5 | 0.176798921 | 2.780020873 | 4.823873719 | 1.61E-06 | 1.49E-05 | 4.518902068 |
| MAP3K3 | -0.122017118 | 3.232177952 | -4.82320999 | 1.62E-06 | 1.49E-05 | 4.515799239 |
| SPRED1 | -0.131197292 | 3.163566873 | -4.82185447 | 1.63E-06 | 1.50E-05 | 4.509463628 |
| HSP90AA1 | 0.152839159 | 6.109115312 | 4.820895176 | 1.64E-06 | 1.51E-05 | 4.504980974 |
| NSUN2 | 0.138934912 | 3.917265802 | 4.820065692 | 1.64E-06 | 1.51E-05 | 4.501105602 |
| GIMAP1 | -0.146188552 | 1.860018307 | -4.81833402 | 1.66E-06 | 1.53E-05 | 4.493017232 |
| DRAP1 | 0.126120453 | 4.628022559 | 4.817520993 | 1.66E-06 | 1.53E-05 | 4.489220682 |
| YIF1A | 0.139270991 | 4.26025061 | 4.816924241 | 1.67E-06 | 1.53E-05 | 4.486434442 |
| ZNF711 | -0.213913559 | 2.273668896 | -4.81222994 | 1.71E-06 | 1.57E-05 | 4.464528241 |
| LRSAM1 | -0.127495569 | 3.090641454 | -4.81192479 | 1.71E-06 | 1.57E-05 | 4.463104934 |
| DCLK1 | -0.150981854 | 1.802933408 | -4.81046579 | 1.72E-06 | 1.58E-05 | 4.456301008 |
| PECR | 0.153029247 | 2.79901854 | 4.809851665 | 1.73E-06 | 1.58E-05 | 4.453437697 |
| RELT | 0.130132218 | 2.650094949 | 4.808719111 | 1.74E-06 | 1.59E-05 | 4.448158133 |
| LIN54 | 0.109843858 | 2.518980633 | 4.806927098 | 1.75E-06 | 1.60E-05 | 4.439806836 |
| PLAGL1 | -0.22596453 | 2.620440941 | -4.80614022 | 1.76E-06 | 1.61E-05 | 4.436140707 |
| NIPSNAP3A | 0.151944072 | 3.540237485 | 4.805471543 | 1.77E-06 | 1.61E-05 | 4.433025724 |
| CFL1 | 0.148615906 | 5.724008487 | 4.80529246 | 1.77E-06 | 1.61E-05 | 4.432191549 |
| PRPH2 | -0.185938533 | 1.564207337 | -4.80497661 | 1.77E-06 | 1.61E-05 | 4.430720371 |
| WNT2B | -0.18084723 | 1.985287171 | -4.8037626 | 1.78E-06 | 1.62E-05 | 4.425066624 |
| ZNF19 | -0.107945705 | 1.945257848 | -4.80341723 | 1.78E-06 | 1.62E-05 | 4.42345845 |
| SMARCAD1 | -0.122996205 | 3.30135977 | -4.79763127 | 1.83E-06 | 1.67E-05 | 4.396533407 |
| SPOCD1 | 0.18341737 | 1.665624007 | 4.796709863 | 1.84E-06 | 1.67E-05 | 4.392248501 |
| KRT8 | 0.195279744 | 6.191325266 | 4.795076121 | 1.86E-06 | 1.69E-05 | 4.384652875 |
| MATN3 | -0.224930569 | 1.858767258 | -4.79362676 | 1.87E-06 | 1.70E-05 | 4.377916574 |
| ST3GAL1 | 0.24248349 | 3.751899789 | 4.792249529 | 1.88E-06 | 1.71E-05 | 4.371517283 |
| CCDC115 | -0.1139827 | 3.670941465 | -4.78995722 | 1.90E-06 | 1.72E-05 | 4.360870039 |
| TLE1 | 0.156242375 | 4.054998465 | 4.789149726 | 1.91E-06 | 1.73E-05 | 4.357120558 |
| DTL | 0.178789248 | 2.80364583 | 4.78810405 | 1.92E-06 | 1.74E-05 | 4.352266032 |
| ELF2 | -0.104994742 | 3.218960332 | -4.7861396 | 1.94E-06 | 1.75E-05 | 4.343148866 |
| UBAP1 | 0.118913078 | 4.166801591 | 4.786105334 | 1.94E-06 | 1.75E-05 | 4.342989869 |
| CMPK1 | 0.132711872 | 4.176886285 | 4.785925529 | 1.94E-06 | 1.75E-05 | 4.342155561 |
| NAPG | 0.127146947 | 3.228598079 | 4.785919071 | 1.94E-06 | 1.75E-05 | 4.342125595 |
| TBC1D14 | -0.153029827 | 3.388326632 | -4.78448806 | 1.96E-06 | 1.76E-05 | 4.335486696 |
| RNASEL | -0.124065654 | 2.267879801 | -4.78235081 | 1.98E-06 | 1.78E-05 | 4.325574922 |
| C7orf31 | -0.150438258 | 2.125270412 | -4.78143769 | 1.99E-06 | 1.78E-05 | 4.321341479 |
| ADHFE1 | -0.168142814 | 2.293115776 | -4.7797638 | 2.00E-06 | 1.79E-05 | 4.313582979 |
| SRRM2 | -0.165916074 | 5.238722266 | -4.77973624 | 2.00E-06 | 1.79E-05 | 4.313455235 |
| PLCG2 | -0.13438228 | 2.304249748 | -4.77971307 | 2.00E-06 | 1.79E-05 | 4.313347869 |
| PCSK2 | -0.320516698 | 1.355325519 | -4.77887226 | 2.01E-06 | 1.80E-05 | 4.309451707 |
| LDB2 | -0.151877617 | 2.497423836 | -4.77725554 | 2.03E-06 | 1.81E-05 | 4.30196199 |
| C6 | -0.217548272 | 1.197580475 | -4.77623037 | 2.04E-06 | 1.82E-05 | 4.297213962 |
| TMSB10 | 0.164454686 | 7.550900267 | 4.774255792 | 2.06E-06 | 1.84E-05 | 4.288071566 |
| PGM5 | -0.233075551 | 2.008467723 | -4.77371223 | 2.06E-06 | 1.84E-05 | 4.285555468 |
| GMDS | 0.15864946 | 3.219767361 | 4.771690466 | 2.08E-06 | 1.86E-05 | 4.276199382 |
| OSBPL1A | -0.149261958 | 3.150385094 | -4.77021509 | 2.10E-06 | 1.87E-05 | 4.26937417 |
| ATOX1 | 0.134287944 | 3.750424471 | 4.765944973 | 2.14E-06 | 1.91E-05 | 4.249631737 |
| TPM3 | 0.105004532 | 4.83067914 | 4.765707276 | 2.14E-06 | 1.91E-05 | 4.248533267 |
| CHIC2 | 0.125642811 | 3.374015524 | 4.765356111 | 2.15E-06 | 1.91E-05 | 4.246910522 |
| PDHX | 0.122085407 | 3.441691633 | 4.762474089 | 2.18E-06 | 1.93E-05 | 4.233596928 |
| EIF3L | -0.153807027 | 4.815275678 | -4.76115994 | 2.19E-06 | 1.94E-05 | 4.22752875 |
| PDK4 | -0.220477569 | 2.873247902 | -4.76061906 | 2.20E-06 | 1.95E-05 | 4.225031655 |
| TMEM106C | 0.168516268 | 4.176424348 | 4.757367896 | 2.23E-06 | 1.98E-05 | 4.21002765 |
| CAPN3 | -0.131744292 | 1.733847967 | -4.75596812 | 2.25E-06 | 1.99E-05 | 4.203570762 |
| PRPF38A | 0.106883127 | 3.742185044 | 4.755038065 | 2.26E-06 | 2.00E-05 | 4.199281612 |
| NR2F2 | -0.185701414 | 3.719869304 | -4.75418019 | 2.27E-06 | 2.00E-05 | 4.195326029 |
| TCERG1L | -0.170993476 | 0.993104772 | -4.75348463 | 2.27E-06 | 2.01E-05 | 4.192119405 |
| BCAS2 | 0.140330142 | 4.101239662 | 4.753404768 | 2.27E-06 | 2.01E-05 | 4.191751256 |
| TLR7 | -0.172841552 | 2.03917513 | -4.75075663 | 2.30E-06 | 2.03E-05 | 4.1795472 |
| SPAG5 | 0.180364324 | 3.063187091 | 4.750460711 | 2.31E-06 | 2.03E-05 | 4.178183856 |
| CPXM1 | -0.298821822 | 4.20331442 | -4.75036898 | 2.31E-06 | 2.03E-05 | 4.17776124 |
| HSD17B10 | 0.149898545 | 4.543284136 | 4.748552123 | 2.33E-06 | 2.05E-05 | 4.169392624 |
| KERA | -0.184893857 | 1.027465795 | -4.74771178 | 2.34E-06 | 2.05E-05 | 4.165522942 |
| UTRN | -0.148645074 | 3.547937332 | -4.74768116 | 2.34E-06 | 2.05E-05 | 4.165381959 |
| LGALS3 | 0.209661699 | 4.662316732 | 4.747056644 | 2.35E-06 | 2.06E-05 | 4.162506592 |
| NOP10 | 0.161666676 | 4.922274662 | 4.746741875 | 2.35E-06 | 2.06E-05 | 4.161057483 |
| FH | 0.136025505 | 4.120642019 | 4.74667785 | 2.35E-06 | 2.06E-05 | 4.160762742 |
| NOD1 | -0.107553568 | 2.637293886 | -4.74579796 | 2.36E-06 | 2.07E-05 | 4.156712514 |
| ZNF573 | -0.103072617 | 2.068594944 | -4.74161645 | 2.41E-06 | 2.11E-05 | 4.137474388 |
| EVC2 | -0.129837709 | 2.507037256 | -4.73957944 | 2.43E-06 | 2.13E-05 | 4.128108479 |
| DLD | 0.130429168 | 3.827418859 | 4.736684303 | 2.47E-06 | 2.15E-05 | 4.114803666 |
| ZBED5 | -0.106623699 | 3.325061102 | -4.73658266 | 2.47E-06 | 2.15E-05 | 4.114336689 |
| TIMM17B | 0.174065749 | 3.986322906 | 4.73602391 | 2.47E-06 | 2.16E-05 | 4.111769871 |
| PIF1 | 0.166927173 | 2.439987613 | 4.733830717 | 2.50E-06 | 2.18E-05 | 4.101697415 |
| ZNF623 | 0.14338018 | 3.529505733 | 4.732822792 | 2.51E-06 | 2.19E-05 | 4.09706992 |
| ZNF512 | -0.135917585 | 3.751700358 | -4.7288646 | 2.56E-06 | 2.23E-05 | 4.078906584 |
| BIVM | -0.149602 | 2.606351646 | -4.72695593 | 2.58E-06 | 2.25E-05 | 4.070153237 |
| COL4A4 | -0.204935816 | 1.638480896 | -4.72536709 | 2.60E-06 | 2.26E-05 | 4.062869279 |
| WDHD1 | 0.14117233 | 2.52798193 | 4.724244926 | 2.62E-06 | 2.27E-05 | 4.057726212 |
| CHRD | -0.165976014 | 1.911012456 | -4.72190321 | 2.65E-06 | 2.30E-05 | 4.046997466 |
| DNTTIP2 | 0.10885017 | 3.649084209 | 4.721889239 | 2.65E-06 | 2.30E-05 | 4.046933461 |
| ARHGAP24 | -0.123802786 | 2.002043697 | -4.72167046 | 2.65E-06 | 2.30E-05 | 4.045931376 |
| PCDHB11 | -0.242008994 | 2.217048162 | -4.72099843 | 2.66E-06 | 2.30E-05 | 4.042853516 |
| UQCRB | 0.159678005 | 4.492189463 | 4.719262327 | 2.68E-06 | 2.32E-05 | 4.034904158 |
| MCM6 | 0.163597014 | 3.730474656 | 4.715866039 | 2.73E-06 | 2.36E-05 | 4.019361197 |
| ODF2 | 0.136019941 | 3.647076517 | 4.715316238 | 2.73E-06 | 2.36E-05 | 4.016846065 |
| SURF4 | 0.12918285 | 4.898462583 | 4.715111428 | 2.74E-06 | 2.36E-05 | 4.015909211 |
| CNN1 | -0.279928588 | 2.506088862 | -4.71508954 | 2.74E-06 | 2.36E-05 | 4.015809079 |
| COQ10B | 0.120115691 | 3.195420637 | 4.713931535 | 2.75E-06 | 2.37E-05 | 4.01051282 |
| RASA3 | -0.156155978 | 2.843997248 | -4.71311302 | 2.76E-06 | 2.38E-05 | 4.006770014 |
| SETDB1 | -0.118364928 | 3.754809744 | -4.7124896 | 2.77E-06 | 2.38E-05 | 4.003919712 |
| ARMC10 | 0.12170282 | 3.908276414 | 4.712463791 | 2.77E-06 | 2.38E-05 | 4.003801714 |
| PJA2 | -0.129634034 | 3.960782991 | -4.7102947 | 2.80E-06 | 2.41E-05 | 3.993887447 |
| ROMO1 | 0.170563325 | 4.991395385 | 4.709965679 | 2.81E-06 | 2.41E-05 | 3.992383969 |
| MT1H | 0.460843592 | 2.730154287 | 4.707803305 | 2.83E-06 | 2.43E-05 | 3.982505418 |
| SPTBN1 | -0.143561878 | 3.865150987 | -4.70743909 | 2.84E-06 | 2.43E-05 | 3.980841976 |
| ARHGEF1 | -0.14615926 | 3.620861164 | -4.70684884 | 2.85E-06 | 2.44E-05 | 3.978146423 |
| NMD3 | 0.128051834 | 3.885088338 | 4.703215472 | 2.90E-06 | 2.48E-05 | 3.961560842 |
| ALDH3A2 | -0.143436242 | 3.665251256 | -4.70268502 | 2.90E-06 | 2.48E-05 | 3.959140457 |
| N4BP2L2 | -0.115420963 | 3.436185603 | -4.69735327 | 2.98E-06 | 2.55E-05 | 3.934826975 |
| FCGRT | -0.158424789 | 4.283965758 | -4.69680843 | 2.99E-06 | 2.55E-05 | 3.932343885 |
| TFPI2 | 0.361698182 | 2.863034585 | 4.693913558 | 3.03E-06 | 2.58E-05 | 3.919155437 |
| XK | 0.227358158 | 2.062428961 | 4.69349531 | 3.04E-06 | 2.59E-05 | 3.917250628 |
| MAPK7 | -0.117699132 | 3.027738703 | -4.69075165 | 3.08E-06 | 2.62E-05 | 3.904759343 |
| IQCE | -0.139182337 | 2.926801293 | -4.69003893 | 3.09E-06 | 2.63E-05 | 3.901515597 |
| BRMS1L | 0.127480659 | 2.542568914 | 4.689957138 | 3.09E-06 | 2.63E-05 | 3.901143383 |
| PSMB7 | 0.111455964 | 4.608674265 | 4.687824974 | 3.12E-06 | 2.65E-05 | 3.891442504 |
| ULK3 | -0.1592012 | 3.77189816 | -4.68735464 | 3.13E-06 | 2.65E-05 | 3.889303171 |
| GK | 0.137745681 | 2.163011242 | 4.68633732 | 3.14E-06 | 2.66E-05 | 3.884676509 |
| MAPK1 | 0.139042128 | 3.689463233 | 4.686224682 | 3.14E-06 | 2.66E-05 | 3.884164302 |
| CAPN2 | -0.16762229 | 4.410025069 | -4.68503825 | 3.16E-06 | 2.68E-05 | 3.878769896 |
| AURKAIP1 | 0.147333847 | 4.610470223 | 4.684127082 | 3.18E-06 | 2.69E-05 | 3.874627934 |
| SAP18 | 0.136574132 | 4.516071782 | 4.684054464 | 3.18E-06 | 2.69E-05 | 3.874297862 |
| AGPAT2 | 0.195504809 | 4.15339268 | 4.683698949 | 3.18E-06 | 2.69E-05 | 3.872682008 |
| ATG16L2 | -0.136027057 | 2.754880213 | -4.68293307 | 3.19E-06 | 2.70E-05 | 3.86920141 |
| ADAMTS10 | -0.168748496 | 2.416779638 | -4.68037904 | 3.23E-06 | 2.73E-05 | 3.857598307 |
| LRRC49 | -0.126448856 | 2.212567529 | -4.678825 | 3.26E-06 | 2.75E-05 | 3.850541232 |
| COPS6 | 0.115349107 | 4.608504048 | 4.678615118 | 3.26E-06 | 2.75E-05 | 3.849588283 |
| C22orf39 | 0.129045466 | 2.841788979 | 4.677531808 | 3.28E-06 | 2.76E-05 | 3.844670366 |
| PTP4A2 | 0.124058486 | 4.738878662 | 4.675891448 | 3.30E-06 | 2.78E-05 | 3.837225683 |
| DLG4 | -0.1533074 | 2.85960121 | -4.67371607 | 3.34E-06 | 2.80E-05 | 3.827356724 |
| TMEM123 | 0.167342968 | 4.990061109 | 4.673512178 | 3.34E-06 | 2.80E-05 | 3.826431949 |
| PHF3 | -0.112281445 | 3.556037016 | -4.67341956 | 3.34E-06 | 2.80E-05 | 3.826011883 |
| WIPI2 | -0.117878332 | 3.797361848 | -4.6726099 | 3.35E-06 | 2.81E-05 | 3.82234007 |
| PRPS2 | 0.156385287 | 3.757177312 | 4.671773685 | 3.37E-06 | 2.82E-05 | 3.818548476 |
| RNASE1 | 0.267373753 | 5.113450425 | 4.671725053 | 3.37E-06 | 2.82E-05 | 3.818327987 |
| CEBPB | 0.162452766 | 5.225412413 | 4.670516974 | 3.39E-06 | 2.83E-05 | 3.812851477 |
| TRIM6 | -0.185924245 | 1.937005771 | -4.66813995 | 3.43E-06 | 2.87E-05 | 3.80207983 |
| RAB7A | 0.119825287 | 5.164689944 | 4.66624659 | 3.46E-06 | 2.89E-05 | 3.793503691 |
| ZC4H2 | -0.190303997 | 2.228634403 | -4.66458914 | 3.49E-06 | 2.91E-05 | 3.785998888 |
| HSF1 | 0.154143015 | 4.601313844 | 4.663718571 | 3.50E-06 | 2.92E-05 | 3.782058025 |
| LPL | -0.214844835 | 2.163201618 | -4.66352302 | 3.50E-06 | 2.92E-05 | 3.781172917 |
| MDM4 | -0.131356971 | 3.307781739 | -4.66350254 | 3.50E-06 | 2.92E-05 | 3.781080232 |
| MRPL39 | 0.138493791 | 3.576611214 | 4.662728796 | 3.52E-06 | 2.93E-05 | 3.777578418 |
| LPCAT1 | 0.164969821 | 3.813004971 | 4.661095202 | 3.54E-06 | 2.95E-05 | 3.770186953 |
| IRAK1 | 0.17505524 | 4.532290998 | 4.657486721 | 3.61E-06 | 3.00E-05 | 3.753868593 |
| VSNL1 | -0.189167503 | 1.85193497 | -4.65716026 | 3.61E-06 | 3.00E-05 | 3.752392876 |
| TMEM88 | -0.226193342 | 2.230071313 | -4.65651677 | 3.62E-06 | 3.01E-05 | 3.749484335 |
| GPBP1L1 | 0.116166935 | 4.230525446 | 4.655185369 | 3.65E-06 | 3.02E-05 | 3.743467699 |
| DAPK1 | -0.156798099 | 3.101417794 | -4.65509415 | 3.65E-06 | 3.02E-05 | 3.743055515 |
| ZBTB8OS | 0.13075998 | 3.435492516 | 4.653200432 | 3.68E-06 | 3.05E-05 | 3.734500794 |
| IQSEC1 | -0.13630477 | 3.384094255 | -4.6531138 | 3.68E-06 | 3.05E-05 | 3.734109536 |
| DRG2 | -0.096612418 | 2.966996822 | -4.65199463 | 3.70E-06 | 3.06E-05 | 3.729055421 |
| FGFRL1 | -0.243535769 | 4.461291983 | -4.65011846 | 3.73E-06 | 3.08E-05 | 3.720585319 |
| GNA14 | -0.154289707 | 1.582764643 | -4.64962681 | 3.74E-06 | 3.09E-05 | 3.718366318 |
| HSPA14 | 0.125345744 | 3.359632103 | 4.649463037 | 3.75E-06 | 3.09E-05 | 3.717627166 |
| FOLR1 | 0.36774301 | 5.338275589 | 4.648111866 | 3.77E-06 | 3.11E-05 | 3.711530089 |
| MS4A7 | -0.202287941 | 2.963696011 | -4.64577698 | 3.81E-06 | 3.14E-05 | 3.70099806 |
| EZH1 | -0.114585759 | 3.09352657 | -4.64551498 | 3.82E-06 | 3.14E-05 | 3.69981659 |
| VDAC3 | 0.135789107 | 4.370374817 | 4.644551092 | 3.83E-06 | 3.15E-05 | 3.695470456 |
| GYG1 | 0.152655096 | 3.750567976 | 4.644336324 | 3.84E-06 | 3.15E-05 | 3.694502197 |
| BRCC3 | 0.157518462 | 3.479903369 | 4.643761521 | 3.85E-06 | 3.16E-05 | 3.691910971 |
| GPRC5C | 0.236505024 | 3.518856293 | 4.643587646 | 3.85E-06 | 3.16E-05 | 3.691127201 |
| KPNA5 | -0.116289886 | 2.503538557 | -4.64248156 | 3.87E-06 | 3.17E-05 | 3.686141989 |
| DENND2A | -0.159866084 | 1.994290712 | -4.64231303 | 3.87E-06 | 3.17E-05 | 3.685382498 |
| ACACB | -0.125374149 | 2.640974319 | -4.64037356 | 3.91E-06 | 3.20E-05 | 3.676644199 |
| TMBIM6 | 0.145531278 | 5.829276455 | 4.63847885 | 3.95E-06 | 3.23E-05 | 3.668110978 |
| MYNN | 0.123567348 | 3.143855311 | 4.635934768 | 3.99E-06 | 3.27E-05 | 3.656658412 |
| KIDINS220 | -0.13342377 | 3.517712964 | -4.63534693 | 4.01E-06 | 3.27E-05 | 3.654013051 |
| RAMP2 | -0.205815301 | 2.989966184 | -4.63509558 | 4.01E-06 | 3.27E-05 | 3.652881985 |
| MED8 | 0.111412652 | 3.778698879 | 4.633947651 | 4.03E-06 | 3.29E-05 | 3.647717302 |
| ECHS1 | 0.144667247 | 5.019185742 | 4.632591967 | 4.06E-06 | 3.31E-05 | 3.641619463 |
| AFF3 | -0.164385474 | 1.714286716 | -4.63195117 | 4.07E-06 | 3.32E-05 | 3.638737753 |
| RCBTB1 | -0.124313686 | 3.017926469 | -4.63157119 | 4.08E-06 | 3.32E-05 | 3.637029154 |
| HSPG2 | -0.21010605 | 4.275674228 | -4.63123787 | 4.08E-06 | 3.32E-05 | 3.635530492 |
| MAP3K13 | 0.13957118 | 3.473292232 | 4.63027519 | 4.10E-06 | 3.33E-05 | 3.631202613 |
| CYP39A1 | -0.163350034 | 2.163223556 | -4.6277063 | 4.15E-06 | 3.37E-05 | 3.619658024 |
| MRPL4 | 0.17000648 | 4.115036552 | 4.627426879 | 4.16E-06 | 3.37E-05 | 3.618402697 |
| ACSM5 | -0.161650651 | 1.374923218 | -4.62741454 | 4.16E-06 | 3.37E-05 | 3.618347261 |
| POLR3GL | -0.123371839 | 3.236629409 | -4.62738248 | 4.16E-06 | 3.37E-05 | 3.618203232 |
| RBMS3 | -0.158130503 | 2.180368927 | -4.62492465 | 4.21E-06 | 3.41E-05 | 3.607164252 |
| NME1 | 0.159653 | 4.108710558 | 4.623870396 | 4.23E-06 | 3.42E-05 | 3.60243097 |
| FES | -0.138127747 | 3.072276561 | -4.62228153 | 4.26E-06 | 3.44E-05 | 3.595299362 |
| CUBN | -0.126758414 | 1.837895229 | -4.62031349 | 4.30E-06 | 3.47E-05 | 3.586469159 |
| GRPEL2 | 0.119839389 | 3.054892119 | 4.619505408 | 4.32E-06 | 3.49E-05 | 3.582844465 |
| EDN1 | 0.269915769 | 2.660303977 | 4.617092628 | 4.37E-06 | 3.52E-05 | 3.572025498 |
| ENOX1 | -0.181969714 | 1.976702681 | -4.616197 | 4.38E-06 | 3.53E-05 | 3.568010845 |
| CPEB1 | -0.190900465 | 1.390598577 | -4.61572977 | 4.39E-06 | 3.54E-05 | 3.565916798 |
| DZIP1L | -0.119832406 | 2.273799304 | -4.61511518 | 4.41E-06 | 3.55E-05 | 3.563162604 |
| PTPRN2 | -0.183523445 | 1.731365925 | -4.61483492 | 4.41E-06 | 3.55E-05 | 3.56190678 |
| PNRC2 | -0.124144711 | 4.428154071 | -4.61373813 | 4.44E-06 | 3.56E-05 | 3.556992888 |
| SAC3D1 | 0.161523483 | 3.316178256 | 4.613725718 | 4.44E-06 | 3.56E-05 | 3.556937264 |
| COL24A1 | -0.150070827 | 1.779023516 | -4.61310393 | 4.45E-06 | 3.57E-05 | 3.554151973 |
| BST2 | 0.320032491 | 5.909663355 | 4.608777075 | 4.54E-06 | 3.64E-05 | 3.534779948 |
| FTL | 0.148170953 | 7.54835738 | 4.607597128 | 4.57E-06 | 3.66E-05 | 3.529500162 |
| FOXN1 | -0.206734525 | 1.396373982 | -4.6071065 | 4.58E-06 | 3.67E-05 | 3.527305163 |
| MAP6D1 | 0.160065712 | 2.048823493 | 4.605172071 | 4.62E-06 | 3.70E-05 | 3.51865309 |
| RGS5 | -0.168829956 | 3.310263109 | -4.60109817 | 4.71E-06 | 3.77E-05 | 3.500443223 |
| SEMA3A | -0.242715602 | 2.345948799 | -4.60059757 | 4.72E-06 | 3.77E-05 | 3.498206658 |
| TXNDC12 | 0.129136593 | 4.326886658 | 4.599831321 | 4.74E-06 | 3.78E-05 | 3.494783717 |
| SH3BP5 | -0.158345011 | 3.219057222 | -4.59920088 | 4.75E-06 | 3.79E-05 | 3.491967836 |
| HGF | -0.132168589 | 1.75144395 | -4.59760464 | 4.79E-06 | 3.82E-05 | 3.484839873 |
| GON4L | -0.113538281 | 3.378200456 | -4.59277231 | 4.90E-06 | 3.90E-05 | 3.463275753 |
| RSRC1 | 0.11847448 | 3.299632569 | 4.592635785 | 4.90E-06 | 3.90E-05 | 3.462666831 |
| TMEM52 | 0.224702313 | 1.799710301 | 4.591563843 | 4.92E-06 | 3.92E-05 | 3.457886412 |
| PMAIP1 | 0.212265698 | 2.702960673 | 4.588330604 | 5.00E-06 | 3.98E-05 | 3.443473988 |
| PUSL1 | 0.170324096 | 3.057334171 | 4.586988538 | 5.03E-06 | 4.00E-05 | 3.437494485 |
| INPP5D | -0.183888284 | 2.753846847 | -4.58626864 | 5.05E-06 | 4.01E-05 | 3.434287691 |
| INCENP | 0.153070978 | 3.379549571 | 4.585567398 | 5.06E-06 | 4.02E-05 | 3.431164506 |
| NDUFB4 | 0.133259026 | 4.741625796 | 4.585007827 | 5.08E-06 | 4.03E-05 | 3.428672607 |
| SPTLC1 | 0.142760801 | 3.773717449 | 4.583531856 | 5.11E-06 | 4.05E-05 | 3.422101167 |
| HTATIP2 | 0.181511753 | 3.21406451 | 4.583233822 | 5.12E-06 | 4.05E-05 | 3.420774478 |
| MFSD3 | 0.211891478 | 3.867329244 | 4.583101075 | 5.12E-06 | 4.05E-05 | 3.420183591 |
| C2 | 0.238857397 | 3.460370686 | 4.581851128 | 5.15E-06 | 4.07E-05 | 3.414620564 |
| S100A3 | 0.248626104 | 1.774319216 | 4.578624593 | 5.23E-06 | 4.13E-05 | 3.40026726 |
| MLLT1 | -0.143049495 | 3.808697666 | -4.57656205 | 5.28E-06 | 4.17E-05 | 3.391097082 |
| TTYH2 | -0.139981212 | 2.145505202 | -4.57555918 | 5.31E-06 | 4.19E-05 | 3.386639694 |
| ZDHHC9 | 0.139325712 | 3.736821313 | 4.575417095 | 5.31E-06 | 4.19E-05 | 3.386008269 |
| ABCG2 | -0.162177957 | 1.64647943 | -4.57534774 | 5.31E-06 | 4.19E-05 | 3.385700043 |
| SORT1 | 0.163767367 | 4.381752227 | 4.575102298 | 5.32E-06 | 4.19E-05 | 3.384609355 |
| PCDH18 | -0.173223739 | 2.32603522 | -4.57490522 | 5.32E-06 | 4.19E-05 | 3.383733604 |
| RBM19 | -0.115536965 | 3.25716155 | -4.57457631 | 5.33E-06 | 4.19E-05 | 3.382272151 |
| VWA1 | 0.189627389 | 4.391792544 | 4.574427049 | 5.34E-06 | 4.19E-05 | 3.381608945 |
| PPP1R12A | -0.109133528 | 3.487529085 | -4.57371551 | 5.35E-06 | 4.20E-05 | 3.378447744 |
| SLBP | 0.159716896 | 4.335235303 | 4.571731387 | 5.40E-06 | 4.24E-05 | 3.369635248 |
| TLR10 | -0.166137821 | 1.424901308 | -4.56756772 | 5.51E-06 | 4.32E-05 | 3.351154253 |
| CLDN7 | 0.199352559 | 4.780453713 | 4.56752231 | 5.51E-06 | 4.32E-05 | 3.350952775 |
| CHRDL1 | -0.271121141 | 2.315859052 | -4.56589481 | 5.55E-06 | 4.35E-05 | 3.343733348 |
| SON | -0.104478104 | 4.530063643 | -4.56216459 | 5.65E-06 | 4.42E-05 | 3.327195835 |
| HDGF | 0.115118609 | 5.849460238 | 4.561242867 | 5.68E-06 | 4.44E-05 | 3.323111487 |
| PRKG1 | -0.127138084 | 2.126461978 | -4.56062729 | 5.69E-06 | 4.45E-05 | 3.320384151 |
| LRRC8D | 0.124184508 | 3.73076022 | 4.558981837 | 5.74E-06 | 4.48E-05 | 3.313095732 |
| HIC1 | -0.188947312 | 2.388001166 | -4.55546456 | 5.83E-06 | 4.55E-05 | 3.29752461 |
| CYTH1 | -0.120247722 | 3.315778317 | -4.55419104 | 5.87E-06 | 4.57E-05 | 3.291889567 |
| CAPZA2 | 0.139284962 | 3.978563585 | 4.553525524 | 5.88E-06 | 4.58E-05 | 3.288945385 |
| SNX33 | -0.123663781 | 3.238973757 | -4.5532355 | 5.89E-06 | 4.59E-05 | 3.287662481 |
| TBC1D7 | 0.136956114 | 2.890620297 | 4.548296987 | 6.03E-06 | 4.69E-05 | 3.265829298 |
| LYNX1 | 0.302567697 | 2.71870355 | 4.547672411 | 6.05E-06 | 4.70E-05 | 3.263069664 |
| ADARB1 | -0.13010521 | 2.749070646 | -4.54739151 | 6.05E-06 | 4.70E-05 | 3.261828642 |
| GNG2 | -0.147138624 | 2.235978483 | -4.54677236 | 6.07E-06 | 4.71E-05 | 3.259093495 |
| GNL1 | -0.125200514 | 3.574961734 | -4.54523566 | 6.12E-06 | 4.74E-05 | 3.252306609 |
| GSTP1 | 0.156586661 | 6.11589563 | 4.541723154 | 6.22E-06 | 4.82E-05 | 3.236801702 |
| NSUN6 | -0.117309874 | 2.68308096 | -4.53958211 | 6.28E-06 | 4.86E-05 | 3.227356371 |
| PHF5A | 0.126415674 | 3.747464121 | 4.538310828 | 6.32E-06 | 4.89E-05 | 3.221750058 |
| WNT10B | -0.169790753 | 1.350859275 | -4.53750413 | 6.34E-06 | 4.90E-05 | 3.218193349 |
| LRRTM4 | -0.223776276 | 1.274516326 | -4.53690678 | 6.36E-06 | 4.91E-05 | 3.215560004 |
| GBP5 | 0.245762842 | 2.724480388 | 4.536740147 | 6.36E-06 | 4.91E-05 | 3.214825498 |
| GOPC | -0.121393022 | 3.436452129 | -4.53337673 | 6.46E-06 | 4.99E-05 | 3.200005178 |
| PDGFRA | -0.209508712 | 2.667842604 | -4.53151819 | 6.52E-06 | 5.03E-05 | 3.191820354 |
| EFS | -0.256437799 | 2.767868094 | -4.5311501 | 6.53E-06 | 5.03E-05 | 3.19019973 |
| PDK3 | 0.160529764 | 2.394314301 | 4.528978527 | 6.60E-06 | 5.08E-05 | 3.180641191 |
| SEMA3G | -0.14652261 | 2.054019143 | -4.52784895 | 6.63E-06 | 5.10E-05 | 3.17567091 |
| HDC | -0.17861232 | 1.31785171 | -4.52657889 | 6.67E-06 | 5.13E-05 | 3.17008391 |
| TMEM61 | 0.247893323 | 1.934673639 | 4.526435462 | 6.67E-06 | 5.13E-05 | 3.169453082 |
| HMBOX1 | -0.133346838 | 2.888711729 | -4.52603865 | 6.69E-06 | 5.14E-05 | 3.167707883 |
| ARNTL2 | 0.185876324 | 2.566015208 | 4.525713602 | 6.70E-06 | 5.14E-05 | 3.166278387 |
| TEKT3 | -0.170857098 | 1.180686724 | -4.52423098 | 6.74E-06 | 5.17E-05 | 3.159759459 |
| KCNJ8 | -0.189108463 | 2.30617243 | -4.5238954 | 6.75E-06 | 5.18E-05 | 3.158284199 |
| SIGLEC8 | -0.188495249 | 1.453383982 | -4.52241252 | 6.80E-06 | 5.21E-05 | 3.151766634 |
| CD200 | -0.276064972 | 3.538589519 | -4.52071916 | 6.85E-06 | 5.24E-05 | 3.144326493 |
| SNRPD1 | 0.148813375 | 3.736212414 | 4.520717204 | 6.85E-06 | 5.24E-05 | 3.144317909 |
| CHMP4B | 0.130391775 | 5.132466605 | 4.520451675 | 6.86E-06 | 5.25E-05 | 3.14315149 |
| NANS | 0.109062707 | 3.276677061 | 4.518920123 | 6.91E-06 | 5.28E-05 | 3.136424974 |
| PCMTD2 | -0.129293956 | 3.788072271 | -4.51808446 | 6.94E-06 | 5.30E-05 | 3.132755698 |
| MRPL35 | 0.095076351 | 3.539621267 | 4.514701033 | 7.05E-06 | 5.38E-05 | 3.117906225 |
| C8orf48 | -0.215601582 | 1.378675337 | -4.51431824 | 7.06E-06 | 5.38E-05 | 3.116226876 |
| NKTR | -0.119111107 | 3.309552071 | -4.51396367 | 7.07E-06 | 5.39E-05 | 3.114671426 |
| SORBS2 | -0.174755959 | 2.493710591 | -4.51235213 | 7.12E-06 | 5.42E-05 | 3.107603477 |
| SMU1 | 0.107169264 | 3.560142897 | 4.512196344 | 7.13E-06 | 5.42E-05 | 3.106920331 |
| TAF2 | 0.128971765 | 3.628376647 | 4.511981904 | 7.14E-06 | 5.43E-05 | 3.105980038 |
| UBE2D2 | 0.101663408 | 4.612703542 | 4.511719524 | 7.15E-06 | 5.43E-05 | 3.104829598 |
| NBR1 | -0.113191993 | 3.705838377 | -4.50971542 | 7.21E-06 | 5.48E-05 | 3.096044471 |
| TNNT2 | -0.270229168 | 2.162859635 | -4.50917303 | 7.23E-06 | 5.48E-05 | 3.093667498 |
| BIN2 | -0.166600587 | 2.480961137 | -4.50844196 | 7.25E-06 | 5.50E-05 | 3.090464103 |
| TNFAIP1 | -0.119758508 | 3.648689279 | -4.50569849 | 7.35E-06 | 5.57E-05 | 3.078447215 |
| F12 | 0.230530107 | 1.680362436 | 4.504571209 | 7.39E-06 | 5.59E-05 | 3.073511586 |
| STARD3 | -0.152522175 | 3.306778359 | -4.50442893 | 7.39E-06 | 5.59E-05 | 3.072888702 |
| ZNF366 | -0.115432129 | 1.442052171 | -4.504193 | 7.40E-06 | 5.59E-05 | 3.071855911 |
| PDCD4 | -0.146029964 | 3.844617366 | -4.50219037 | 7.47E-06 | 5.64E-05 | 3.063091338 |
| PIGQ | -0.125176227 | 3.527704207 | -4.50157411 | 7.49E-06 | 5.65E-05 | 3.060394982 |
| NDRG2 | -0.219958874 | 3.210857107 | -4.50149726 | 7.49E-06 | 5.65E-05 | 3.060058793 |
| RHBDF1 | -0.151897277 | 3.083915493 | -4.50136332 | 7.50E-06 | 5.65E-05 | 3.059472783 |
| RPE | 0.107920583 | 3.755166616 | 4.499151763 | 7.57E-06 | 5.71E-05 | 3.049799895 |
| MRPS25 | 0.13599129 | 3.68656132 | 4.496782627 | 7.66E-06 | 5.76E-05 | 3.039442849 |
| TEX264 | 0.144047795 | 3.636494856 | 4.496632418 | 7.66E-06 | 5.76E-05 | 3.038786362 |
| NEDD8 | 0.124277316 | 4.12227728 | 4.4963027 | 7.67E-06 | 5.77E-05 | 3.037345413 |
| TMEM41A | 0.119472483 | 3.532224586 | 4.495997948 | 7.68E-06 | 5.77E-05 | 3.036013661 |
| MAGI1 | -0.147747655 | 3.016220173 | -4.4957067 | 7.69E-06 | 5.78E-05 | 3.03474101 |
| CDC25B | 0.180014744 | 4.013531222 | 4.494782528 | 7.73E-06 | 5.80E-05 | 3.03070319 |
| TIMM8B | 0.162786311 | 4.33433126 | 4.494645121 | 7.73E-06 | 5.80E-05 | 3.030102912 |
| ASL | 0.154745385 | 3.355449292 | 4.492199555 | 7.82E-06 | 5.86E-05 | 3.019422135 |
| ABCD3 | 0.118150287 | 3.193925435 | 4.491971029 | 7.83E-06 | 5.86E-05 | 3.018424356 |
| ADAMTS6 | -0.139449529 | 1.975422643 | -4.49102247 | 7.86E-06 | 5.88E-05 | 3.014283343 |
| TMEM125 | 0.167964514 | 3.731927769 | 4.490847828 | 7.87E-06 | 5.88E-05 | 3.013520996 |
| EIF1AD | 0.101173352 | 3.414838166 | 4.490078457 | 7.90E-06 | 5.90E-05 | 3.010162973 |
| TMEM43 | -0.11794651 | 4.08560237 | -4.48955498 | 7.92E-06 | 5.91E-05 | 3.007878494 |
| CCL8 | 0.287974073 | 2.230236277 | 4.489037673 | 7.93E-06 | 5.92E-05 | 3.005621211 |
| ARPC3 | 0.130797697 | 5.124306789 | 4.481675737 | 8.21E-06 | 6.12E-05 | 2.973524205 |
| CYB5D2 | -0.142561433 | 3.075920394 | -4.48030956 | 8.26E-06 | 6.16E-05 | 2.967573425 |
| ALDH1L1 | -0.212450191 | 1.775011726 | -4.4793536 | 8.30E-06 | 6.18E-05 | 2.963410531 |
| UBA1 | 0.151533311 | 5.352781106 | 4.477651273 | 8.36E-06 | 6.22E-05 | 2.955999543 |
| DDX41 | 0.119956937 | 4.153920926 | 4.477375353 | 8.37E-06 | 6.23E-05 | 2.954798593 |
| AMT | -0.182481348 | 2.650915835 | -4.47716866 | 8.38E-06 | 6.23E-05 | 2.953899013 |
| APOBEC3B | 0.270246369 | 2.5766429 | 4.476883527 | 8.39E-06 | 6.23E-05 | 2.952658087 |
| PPP2R1B | 0.135319745 | 2.992304681 | 4.476292159 | 8.41E-06 | 6.25E-05 | 2.950084657 |
| FUT3 | 0.248662505 | 1.88008117 | 4.474648777 | 8.48E-06 | 6.29E-05 | 2.942934947 |
| SNX6 | 0.123406823 | 4.218178613 | 4.474226835 | 8.49E-06 | 6.30E-05 | 2.941099651 |
| MT1F | 0.308483239 | 3.712615285 | 4.474056217 | 8.50E-06 | 6.30E-05 | 2.94035757 |
| PRRG2 | 0.166128942 | 2.768725523 | 4.473848928 | 8.51E-06 | 6.30E-05 | 2.939456029 |
| CCL21 | -0.370288571 | 2.129365112 | -4.47383859 | 8.51E-06 | 6.30E-05 | 2.939411065 |
| SCN2A | -0.167930657 | 1.509757985 | -4.47251093 | 8.56E-06 | 6.33E-05 | 2.933637775 |
| HAAO | -0.147858054 | 2.007342535 | -4.46742712 | 8.76E-06 | 6.47E-05 | 2.911546283 |
| BCL2A1 | 0.236334093 | 2.199759488 | 4.466810947 | 8.79E-06 | 6.49E-05 | 2.908870383 |
| SCN7A | -0.175260441 | 1.429050877 | -4.46572785 | 8.83E-06 | 6.52E-05 | 2.90416758 |
| CUL7 | -0.167775952 | 3.953528274 | -4.46406964 | 8.90E-06 | 6.56E-05 | 2.896969767 |
| B3GALT2 | -0.177327042 | 1.081398911 | -4.46247233 | 8.96E-06 | 6.61E-05 | 2.890038742 |
| ARHGEF6 | -0.138535948 | 2.460482691 | -4.46083868 | 9.03E-06 | 6.65E-05 | 2.882952482 |
| CABLES1 | -0.182836614 | 3.366244114 | -4.46079093 | 9.03E-06 | 6.65E-05 | 2.882745399 |
| CBR1 | 0.155757686 | 3.73955722 | 4.460435742 | 9.05E-06 | 6.65E-05 | 2.881205047 |
| CYP4X1 | 0.30453093 | 3.037324341 | 4.459147714 | 9.10E-06 | 6.69E-05 | 2.875620242 |
| TMEM54 | 0.219974236 | 3.651448401 | 4.45910128 | 9.10E-06 | 6.69E-05 | 2.875418936 |
| PSEN2 | -0.12603059 | 2.877157567 | -4.45710789 | 9.19E-06 | 6.74E-05 | 2.866778884 |
| CTSO | -0.173590974 | 3.370457499 | -4.45670769 | 9.20E-06 | 6.75E-05 | 2.865044723 |
| TP53BP2 | -0.136138751 | 3.749469436 | -4.45601751 | 9.23E-06 | 6.77E-05 | 2.862054357 |
| AKAP9 | -0.101606956 | 3.296508408 | -4.45396668 | 9.32E-06 | 6.83E-05 | 2.853171294 |
| ITPR1 | -0.131359253 | 2.407796834 | -4.45232989 | 9.39E-06 | 6.87E-05 | 2.846084484 |
| VCP | 0.133003127 | 4.809647017 | 4.452231677 | 9.39E-06 | 6.87E-05 | 2.845659322 |
| COX16 | 0.123406505 | 3.580197095 | 4.452214875 | 9.39E-06 | 6.87E-05 | 2.845586592 |
| PSD3 | -0.129028088 | 2.359271257 | -4.4516475 | 9.42E-06 | 6.88E-05 | 2.843130663 |
| OSBPL5 | -0.126143026 | 2.803881149 | -4.45090367 | 9.45E-06 | 6.90E-05 | 2.8399114 |
| ZBTB12 | -0.173360218 | 3.066420842 | -4.45087862 | 9.45E-06 | 6.90E-05 | 2.839803014 |
| CINP | 0.124528567 | 3.042517614 | 4.44953229 | 9.51E-06 | 6.93E-05 | 2.83397748 |
| MRPS30 | 0.10666805 | 3.596814561 | 4.449027999 | 9.53E-06 | 6.95E-05 | 2.831795868 |
| CHMP2B | 0.126711871 | 3.76990338 | 4.448636736 | 9.55E-06 | 6.95E-05 | 2.830103392 |
| ZNF781 | -0.1384231 | 1.508090838 | -4.44810587 | 9.57E-06 | 6.96E-05 | 2.827807266 |
| HOXD1 | 0.436748151 | 2.558779602 | 4.448100074 | 9.57E-06 | 6.96E-05 | 2.8277822 |
| FAM135B | -0.206237735 | 1.479792399 | -4.44720819 | 9.61E-06 | 6.99E-05 | 2.823925206 |
| CRNKL1 | -0.11802872 | 3.576074613 | -4.44695617 | 9.62E-06 | 6.99E-05 | 2.822835465 |
| DGKD | -0.112145973 | 2.801310762 | -4.4464928 | 9.64E-06 | 7.00E-05 | 2.820831967 |
| DHX35 | -0.107754541 | 2.859654479 | -4.44370291 | 9.77E-06 | 7.08E-05 | 2.80877359 |
| FAM160A2 | -0.131574173 | 3.503018513 | -4.44249413 | 9.82E-06 | 7.12E-05 | 2.803551275 |
| PARD3B | -0.119798107 | 2.690106422 | -4.4417755 | 9.85E-06 | 7.13E-05 | 2.800447238 |
| UBAC1 | 0.108372926 | 3.918418246 | 4.441761303 | 9.85E-06 | 7.13E-05 | 2.800385912 |
| HMCN1 | -0.179386138 | 2.536517215 | -4.44116126 | 9.88E-06 | 7.15E-05 | 2.79779447 |
| PRX | -0.147161963 | 1.94702026 | -4.43887069 | 9.99E-06 | 7.22E-05 | 2.787905124 |
| MGP | -0.278181853 | 3.907382906 | -4.43807422 | 1.00E-05 | 7.24E-05 | 2.784467591 |
| GAL3ST3 | -0.334467838 | 1.983866384 | -4.43804508 | 1.00E-05 | 7.24E-05 | 2.784341819 |
| PPP4C | 0.121912892 | 4.711172722 | 4.437536783 | 1.00E-05 | 7.25E-05 | 2.782148358 |
| ISOC2 | 0.144058145 | 3.769154742 | 4.436973923 | 1.01E-05 | 7.26E-05 | 2.779719711 |
| ITM2B | -0.145899748 | 5.264906176 | -4.43678798 | 1.01E-05 | 7.26E-05 | 2.778917467 |
| ANP32E | 0.159575083 | 4.123890827 | 4.436723096 | 1.01E-05 | 7.26E-05 | 2.778637531 |
| TBL2 | 0.095341345 | 3.636244651 | 4.43638195 | 1.01E-05 | 7.26E-05 | 2.777165767 |
| PTGS1 | 0.245605559 | 4.739938071 | 4.436310789 | 1.01E-05 | 7.26E-05 | 2.776858783 |
| FAM120B | -0.112623568 | 3.322741683 | -4.43621794 | 1.01E-05 | 7.26E-05 | 2.776458245 |
| GAP43 | -0.16803673 | 1.114607062 | -4.43269096 | 1.03E-05 | 7.38E-05 | 2.761249161 |
| VDAC2 | 0.119369973 | 4.94006001 | 4.43141354 | 1.03E-05 | 7.42E-05 | 2.755743552 |
| ENTPD1 | -0.125562302 | 2.722692929 | -4.43084973 | 1.04E-05 | 7.43E-05 | 2.753314058 |
| DOCK11 | -0.169180688 | 2.507459534 | -4.42795744 | 1.05E-05 | 7.52E-05 | 2.74085559 |
| ZNF398 | 0.113264215 | 2.957061748 | 4.427300712 | 1.05E-05 | 7.54E-05 | 2.738027829 |
| ZCCHC14 | -0.126019522 | 3.10002704 | -4.42350174 | 1.07E-05 | 7.67E-05 | 2.721678083 |
| NEK8 | -0.12683801 | 2.35570972 | -4.41989891 | 1.09E-05 | 7.79E-05 | 2.706185025 |
| PHYHIP | -0.162361949 | 1.387631182 | -4.41870864 | 1.09E-05 | 7.82E-05 | 2.701069229 |
| NARF | 0.11560133 | 3.510952705 | 4.418631206 | 1.09E-05 | 7.82E-05 | 2.700736448 |
| SLTM | -0.099842249 | 3.856698773 | -4.41590456 | 1.11E-05 | 7.92E-05 | 2.68902248 |
| TMEM33 | 0.131215173 | 3.554434811 | 4.414998335 | 1.11E-05 | 7.94E-05 | 2.6851308 |
| C1orf198 | -0.140142199 | 4.110167166 | -4.41440712 | 1.12E-05 | 7.96E-05 | 2.682592281 |
| NOB1 | -0.123140307 | 4.031063095 | -4.41126134 | 1.13E-05 | 8.07E-05 | 2.669090791 |
| PRIM1 | 0.163103701 | 2.79124154 | 4.407733143 | 1.15E-05 | 8.19E-05 | 2.65395898 |
| TCF12 | -0.119739013 | 3.568023296 | -4.4057729 | 1.16E-05 | 8.26E-05 | 2.645556885 |
| MKLN1 | 0.087684394 | 3.126184495 | 4.404427594 | 1.17E-05 | 8.31E-05 | 2.639792662 |
| MRPS33 | 0.142064671 | 3.436786999 | 4.401455016 | 1.18E-05 | 8.41E-05 | 2.62706208 |
| PHF21A | -0.09677541 | 3.240815912 | -4.39547188 | 1.22E-05 | 8.64E-05 | 2.601463395 |
| PRKAA1 | 0.113495097 | 3.505942656 | 4.393745023 | 1.23E-05 | 8.70E-05 | 2.594081303 |
| SNCAIP | -0.152513196 | 1.881922537 | -4.3934063 | 1.23E-05 | 8.71E-05 | 2.592633619 |
| BAIAP2L1 | 0.13252277 | 3.870643175 | 4.392231883 | 1.23E-05 | 8.75E-05 | 2.587615138 |
| IL6ST | -0.139047564 | 3.624114152 | -4.39070395 | 1.24E-05 | 8.80E-05 | 2.58108792 |
| C1GALT1C1 | 0.139930527 | 3.505167464 | 4.389248463 | 1.25E-05 | 8.86E-05 | 2.574872257 |
| WDR19 | -0.121205416 | 2.950643714 | -4.38857409 | 1.25E-05 | 8.88E-05 | 2.571993019 |
| FYN | -0.187912383 | 3.363723996 | -4.38741582 | 1.26E-05 | 8.92E-05 | 2.56704875 |
| FGD3 | -0.120280178 | 2.417036278 | -4.38630231 | 1.27E-05 | 8.96E-05 | 2.562296759 |
| CACNA1C | -0.111227527 | 2.16123528 | -4.38576813 | 1.27E-05 | 8.97E-05 | 2.560017502 |
| CBR3 | 0.236431723 | 2.193454359 | 4.385753536 | 1.27E-05 | 8.97E-05 | 2.559955225 |
| RERGL | -0.167273219 | 0.918962581 | -4.38557522 | 1.27E-05 | 8.97E-05 | 2.559194444 |
| NLRP3 | -0.127194232 | 1.927749101 | -4.38218084 | 1.29E-05 | 9.10E-05 | 2.544718116 |
| ZNF23 | -0.100166307 | 1.843026221 | -4.38011859 | 1.30E-05 | 9.18E-05 | 2.53592829 |
| SLC25A4 | 0.130864303 | 3.260730161 | 4.380058144 | 1.30E-05 | 9.18E-05 | 2.535670724 |
| WSB2 | 0.12065549 | 3.894852662 | 4.379813991 | 1.31E-05 | 9.18E-05 | 2.534630361 |
| AACS | 0.113660473 | 3.048670573 | 4.377237239 | 1.32E-05 | 9.28E-05 | 2.523653976 |
| TBL1XR1 | 0.139713439 | 4.504852585 | 4.375729758 | 1.33E-05 | 9.34E-05 | 2.517235329 |
| PSMD8 | 0.156553822 | 4.951392367 | 4.375479912 | 1.33E-05 | 9.35E-05 | 2.516171725 |
| CASP6 | 0.15217479 | 3.442107447 | 4.37521287 | 1.33E-05 | 9.35E-05 | 2.515034984 |
| NDUFB3 | 0.146737617 | 4.255961893 | 4.374981763 | 1.33E-05 | 9.36E-05 | 2.514051263 |
| SLCO2B1 | -0.195874483 | 3.098821178 | -4.37401551 | 1.34E-05 | 9.39E-05 | 2.509938899 |
| FBXL6 | 0.17774907 | 3.428219546 | 4.373999201 | 1.34E-05 | 9.39E-05 | 2.509869488 |
| HEATR3 | 0.108339778 | 2.823688547 | 4.372731858 | 1.35E-05 | 9.43E-05 | 2.504477025 |
| PSTPIP2 | 0.17683396 | 2.488201483 | 4.372194012 | 1.35E-05 | 9.45E-05 | 2.502188979 |
| NHP2 | 0.135284726 | 4.867370958 | 4.371913185 | 1.35E-05 | 9.46E-05 | 2.500994424 |
| PLSCR2 | 0.143390133 | 1.509952835 | 4.370441709 | 1.36E-05 | 9.51E-05 | 2.494736407 |
| SLC25A33 | 0.150933408 | 3.044593172 | 4.370288718 | 1.36E-05 | 9.51E-05 | 2.494085871 |
| TMEM53 | 0.130679971 | 2.740002341 | 4.368555433 | 1.37E-05 | 9.58E-05 | 2.486717269 |
| ANK2 | -0.168432634 | 2.219516865 | -4.36484845 | 1.40E-05 | 9.74E-05 | 2.470967472 |
| HVCN1 | -0.135390607 | 2.231361974 | -4.36408268 | 1.40E-05 | 9.76E-05 | 2.467715579 |
| PTPN14 | -0.135508309 | 3.387211306 | -4.36338992 | 1.41E-05 | 9.79E-05 | 2.464774186 |
| TOR1AIP2 | -0.091829898 | 3.353008798 | -4.36282177 | 1.41E-05 | 9.81E-05 | 2.462362185 |
| ZKSCAN3 | -0.11634765 | 2.411090214 | -4.36223799 | 1.41E-05 | 9.83E-05 | 2.459884186 |
| HMGB2 | 0.177133451 | 4.472193331 | 4.361168105 | 1.42E-05 | 9.87E-05 | 2.455343605 |
| CHAC2 | 0.19328927 | 2.189145339 | 4.359066209 | 1.43E-05 | 9.95E-05 | 2.446426302 |
| ZEB2 | -0.137557707 | 2.657775543 | -4.3585233 | 1.44E-05 | 9.97E-05 | 2.444123669 |
| BMPR1A | -0.117152643 | 3.185132617 | -4.35810714 | 1.44E-05 | 9.99E-05 | 2.442358814 |
| PTCH2 | -0.149435975 | 1.976584519 | -4.35710593 | 1.45E-05 | 0.0001 | 2.438113536 |
| TNFSF10 | 0.297655392 | 4.464499962 | 4.355320073 | 1.46E-05 | 0.000101 | 2.430543586 |
| EPM2A | -0.107450664 | 2.021922548 | -4.35499816 | 1.46E-05 | 0.000101 | 2.42917938 |
| GIT2 | -0.084411366 | 2.814924104 | -4.35481003 | 1.46E-05 | 0.000101 | 2.428382162 |
| HP | 0.470543388 | 2.615696964 | 4.354320274 | 1.46E-05 | 0.000101 | 2.426306903 |
| CLIC1 | 0.127723386 | 5.585776105 | 4.352423935 | 1.48E-05 | 0.000102 | 2.418273681 |
| EME1 | 0.149715321 | 2.481057405 | 4.350482417 | 1.49E-05 | 0.000103 | 2.410052572 |
| FBXO6 | 0.185324232 | 3.087045454 | 4.347174283 | 1.51E-05 | 0.000104 | 2.396052852 |
| BCAP29 | 0.114922043 | 3.131757393 | 4.345284954 | 1.52E-05 | 0.000105 | 2.388061996 |
| MAP2K1 | 0.10601921 | 3.723368805 | 4.345106001 | 1.53E-05 | 0.000105 | 2.387305291 |
| NDUFA13 | 0.163067765 | 4.171435263 | 4.342325072 | 1.54E-05 | 0.000106 | 2.375550005 |
| EIF1AX | 0.138734069 | 4.155329606 | 4.341690075 | 1.55E-05 | 0.000107 | 2.372866822 |
| NOVA1 | -0.185307585 | 1.746288607 | -4.34052941 | 1.56E-05 | 0.000107 | 2.367963395 |
| FGFR2 | -0.174916331 | 3.7681587 | -4.33702907 | 1.58E-05 | 0.000109 | 2.353183297 |
| TFAP2C | 0.246493076 | 3.427113933 | 4.336874599 | 1.58E-05 | 0.000109 | 2.35253133 |
| PRMT2 | -0.106413247 | 3.809718958 | -4.33465346 | 1.60E-05 | 0.00011 | 2.343158908 |
| SNAPC1 | 0.124290995 | 2.801183348 | 4.33066907 | 1.63E-05 | 0.000112 | 2.32635783 |
| LIN7B | 0.161329961 | 1.911330124 | 4.330249191 | 1.63E-05 | 0.000112 | 2.324588183 |
| NOL7 | 0.112760583 | 4.310173816 | 4.330242518 | 1.63E-05 | 0.000112 | 2.324560059 |
| SPINT2 | 0.205477064 | 5.663512511 | 4.32979937 | 1.63E-05 | 0.000112 | 2.322692524 |
| DUSP23 | 0.160266204 | 4.618342009 | 4.328450501 | 1.64E-05 | 0.000113 | 2.317009191 |
| PTPRM | -0.181216395 | 3.139963929 | -4.32836617 | 1.64E-05 | 0.000113 | 2.31665392 |
| TSNAXIP1 | -0.141361132 | 1.869314847 | -4.32745374 | 1.65E-05 | 0.000113 | 2.312810546 |
| KIF16B | -0.101560963 | 2.647519669 | -4.32710388 | 1.65E-05 | 0.000113 | 2.311337031 |
| GPC1 | -0.193327565 | 3.808340881 | -4.32703248 | 1.65E-05 | 0.000113 | 2.311036346 |
| FGF17 | -0.268055012 | 1.470242083 | -4.32544582 | 1.67E-05 | 0.000114 | 2.304355382 |
| SASH1 | -0.13584065 | 3.045263292 | -4.32520789 | 1.67E-05 | 0.000114 | 2.303353738 |
| IGFBP4 | -0.229141911 | 4.990722505 | -4.32359194 | 1.68E-05 | 0.000115 | 2.296552261 |
| CREB3 | 0.127900707 | 4.112360103 | 4.322307818 | 1.69E-05 | 0.000115 | 2.29114918 |
| RAB1B | 0.10745521 | 5.047298485 | 4.321398236 | 1.70E-05 | 0.000115 | 2.287322954 |
| MS4A6A | -0.213513644 | 3.20646578 | -4.32123989 | 1.70E-05 | 0.000115 | 2.286656927 |
| BTBD2 | -0.125121531 | 4.140844622 | -4.32005743 | 1.71E-05 | 0.000116 | 2.281684189 |
| TNFRSF13C | -0.159959426 | 1.800219108 | -4.3200097 | 1.71E-05 | 0.000116 | 2.281483492 |
| C5 | -0.126593033 | 1.932593536 | -4.31973085 | 1.71E-05 | 0.000116 | 2.280310998 |
| ZNF546 | -0.107715771 | 1.881962095 | -4.3176058 | 1.72E-05 | 0.000117 | 2.271378156 |
| NPTX2 | 0.3339284 | 2.618507107 | 4.316960877 | 1.73E-05 | 0.000117 | 2.26866801 |
| MAP3K4 | -0.097000729 | 3.042982548 | -4.31543795 | 1.74E-05 | 0.000118 | 2.26226981 |
| MT1E | 0.354054713 | 4.424066014 | 4.314442757 | 1.75E-05 | 0.000118 | 2.2580899 |
| KLK10 | 0.358200446 | 4.371129017 | 4.314393321 | 1.75E-05 | 0.000118 | 2.257882288 |
| GIT1 | -0.110685786 | 3.874361464 | -4.31234664 | 1.77E-05 | 0.000119 | 2.249289085 |
| IGBP1 | -0.118385832 | 4.3483195 | -4.3111859 | 1.77E-05 | 0.00012 | 2.24441736 |
| ABTB1 | -0.134991327 | 3.269627039 | -4.31070465 | 1.78E-05 | 0.00012 | 2.242397861 |
| TAP1 | 0.236539361 | 4.180839437 | 4.310011279 | 1.78E-05 | 0.000121 | 2.239488627 |
| SUPT3H | -0.121861405 | 2.570201066 | -4.3084779 | 1.80E-05 | 0.000121 | 2.233056525 |
| TUBA1A | -0.220859063 | 4.907654008 | -4.30813938 | 1.80E-05 | 0.000121 | 2.231636815 |
| MRPL44 | 0.10821601 | 3.647629593 | 4.306238635 | 1.81E-05 | 0.000122 | 2.223667377 |
| UBE2J1 | 0.114585314 | 3.916260092 | 4.303218769 | 1.84E-05 | 0.000124 | 2.211012667 |
| CENPK | 0.162170563 | 2.708093809 | 4.302961977 | 1.84E-05 | 0.000124 | 2.209936979 |
| KCNK2 | -0.180525228 | 1.706648428 | -4.30102322 | 1.86E-05 | 0.000125 | 2.201817618 |
| COX5A | 0.156967117 | 4.954479497 | 4.300117748 | 1.86E-05 | 0.000125 | 2.198026801 |
| RBM47 | 0.143214026 | 3.973738344 | 4.299276736 | 1.87E-05 | 0.000126 | 2.19450653 |
| YIPF1 | 0.110452033 | 3.640327538 | 4.29921186 | 1.87E-05 | 0.000126 | 2.194235004 |
| IDH2 | 0.17285837 | 4.767649952 | 4.298831249 | 1.87E-05 | 0.000126 | 2.192642099 |
| CLOCK | 0.103754568 | 3.12798329 | 4.29874827 | 1.88E-05 | 0.000126 | 2.192294839 |
| ADAM22 | -0.137974704 | 1.875862551 | -4.29722025 | 1.89E-05 | 0.000127 | 2.185901392 |
| SUSD4 | 0.23644933 | 3.213087984 | 4.29549713 | 1.90E-05 | 0.000128 | 2.178694224 |
| CDC27 | 0.116235756 | 3.420899621 | 4.292168997 | 1.93E-05 | 0.000129 | 2.164781813 |
| FRZB | -0.286032897 | 2.629306647 | -4.29106458 | 1.94E-05 | 0.00013 | 2.160167366 |
| ETHE1 | 0.163064032 | 3.70152644 | 4.290952168 | 1.94E-05 | 0.00013 | 2.159697769 |
| NQO1 | 0.257141183 | 3.651210447 | 4.290712151 | 1.94E-05 | 0.00013 | 2.158695113 |
| ANKMY1 | -0.115981352 | 2.647254639 | -4.29058725 | 1.94E-05 | 0.00013 | 2.158173384 |
| CNR1 | -0.18826108 | 1.455817911 | -4.29049013 | 1.95E-05 | 0.00013 | 2.15776767 |
| DLL1 | -0.177358685 | 2.066126457 | -4.29044866 | 1.95E-05 | 0.00013 | 2.157594477 |
| MRGPRF | -0.187796907 | 2.315194651 | -4.29008196 | 1.95E-05 | 0.00013 | 2.1560628 |
| FOLR3 | 0.307081193 | 2.023742808 | 4.289939044 | 1.95E-05 | 0.00013 | 2.155465887 |
| COLQ | -0.123235867 | 2.123668547 | -4.2897657 | 1.95E-05 | 0.00013 | 2.154741911 |
| PKD2 | -0.129400614 | 3.116382333 | -4.28793327 | 1.97E-05 | 0.000131 | 2.147090511 |
| PCYT1B | -0.164613606 | 1.460191483 | -4.28787255 | 1.97E-05 | 0.000131 | 2.146836996 |
| AP2M1 | 0.139018014 | 5.451757546 | 4.286808553 | 1.98E-05 | 0.000131 | 2.142395733 |
| GRIK4 | -0.182806713 | 1.546830823 | -4.28651759 | 1.98E-05 | 0.000132 | 2.141181394 |
| FADS2 | 0.233368152 | 3.431919054 | 4.286109203 | 1.98E-05 | 0.000132 | 2.139477126 |
| CHMP7 | -0.110596575 | 3.242885861 | -4.28443047 | 2.00E-05 | 0.000133 | 2.13247312 |
| CILP | -0.281686789 | 2.839132597 | -4.28374305 | 2.00E-05 | 0.000133 | 2.129605838 |
| PPIH | 0.13014062 | 4.183625017 | 4.283317586 | 2.01E-05 | 0.000133 | 2.127831417 |
| CHEK2 | 0.133178807 | 3.013045473 | 4.281967558 | 2.02E-05 | 0.000134 | 2.122202172 |
| ITGA7 | -0.187217219 | 3.011360311 | -4.28084567 | 2.03E-05 | 0.000134 | 2.117525509 |
| COL18A1 | -0.186901121 | 4.995356337 | -4.2807991 | 2.03E-05 | 0.000134 | 2.117331418 |
| RAB2A | 0.110641512 | 4.448497399 | 4.280365161 | 2.03E-05 | 0.000135 | 2.115522843 |
| DEF6 | -0.139815507 | 3.085963557 | -4.28012695 | 2.04E-05 | 0.000135 | 2.114530105 |
| LCAT | -0.14176766 | 2.475581494 | -4.27912888 | 2.05E-05 | 0.000135 | 2.110371253 |
| PCIF1 | -0.107868288 | 4.033959043 | -4.27739474 | 2.06E-05 | 0.000136 | 2.103147528 |
| EIF5 | 0.114499558 | 4.302539844 | 4.277151761 | 2.06E-05 | 0.000136 | 2.102135591 |
| APEX2 | 0.114942652 | 3.561604227 | 4.276141779 | 2.07E-05 | 0.000137 | 2.097929938 |
| ITGBL1 | -0.217697073 | 2.043631835 | -4.27599445 | 2.07E-05 | 0.000137 | 2.097316515 |
| C21orf91 | 0.142149187 | 3.257627506 | 4.275788955 | 2.08E-05 | 0.000137 | 2.096460975 |
| UBQLNL | -0.14970082 | 1.512872482 | -4.2728155 | 2.10E-05 | 0.000138 | 2.084085814 |
| INMT | -0.156542771 | 1.748841891 | -4.27037052 | 2.13E-05 | 0.00014 | 2.073916334 |
| RPRM | -0.24048816 | 1.603470263 | -4.2689856 | 2.14E-05 | 0.000141 | 2.068158493 |
| RERG | -0.278349842 | 2.726743916 | -4.26833604 | 2.14E-05 | 0.000141 | 2.065458536 |
| TDRD3 | -0.097216394 | 2.65410205 | -4.2679298 | 2.15E-05 | 0.000141 | 2.063770158 |
| ATP6V1D | 0.099596649 | 3.490697951 | 4.26712842 | 2.16E-05 | 0.000142 | 2.060440019 |
| ABLIM3 | -0.121749339 | 1.867954042 | -4.26464275 | 2.18E-05 | 0.000143 | 2.050114654 |
| IFT20 | -0.113482329 | 3.232932358 | -4.26371988 | 2.19E-05 | 0.000144 | 2.046282542 |
| UBL4A | 0.124857986 | 3.896734744 | 4.263098412 | 2.20E-05 | 0.000144 | 2.043702436 |
| MAP4K1 | -0.169351254 | 2.741287477 | -4.26239373 | 2.20E-05 | 0.000144 | 2.040777273 |
| PEX12 | -0.127310907 | 2.326566835 | -4.2621869 | 2.20E-05 | 0.000144 | 2.039918832 |
| FMNL3 | -0.120322116 | 3.271921492 | -4.26173968 | 2.21E-05 | 0.000144 | 2.03806274 |
| GNPTG | -0.107944363 | 3.836496269 | -4.26144008 | 2.21E-05 | 0.000145 | 2.03681941 |
| SLC25A17 | 0.0921776 | 3.083808682 | 4.260639551 | 2.22E-05 | 0.000145 | 2.033497704 |
| SRF | -0.111170602 | 4.037042025 | -4.25916578 | 2.23E-05 | 0.000146 | 2.027384003 |
| CSK | -0.110366823 | 4.004839834 | -4.2572299 | 2.25E-05 | 0.000147 | 2.019356418 |
| TTC37 | -0.112062233 | 3.479420618 | -4.25679227 | 2.26E-05 | 0.000147 | 2.0175422 |
| ARRB2 | -0.113002008 | 3.662607402 | -4.25407384 | 2.28E-05 | 0.000149 | 2.006276672 |
| RPL23A | -0.136789334 | 6.342548454 | -4.25385212 | 2.29E-05 | 0.000149 | 2.005358132 |
| EIF3I | 0.132598984 | 5.150531171 | 4.25354729 | 2.29E-05 | 0.000149 | 2.004095368 |
| WNT6 | -0.329972527 | 2.398560434 | -4.25097524 | 2.32E-05 | 0.000151 | 1.993444138 |
| MED27 | 0.122456715 | 3.243297275 | 4.250194278 | 2.32E-05 | 0.000151 | 1.990211284 |
| CD47 | 0.173104574 | 5.074304509 | 4.249118314 | 2.33E-05 | 0.000152 | 1.985758198 |
| AFG3L2 | 0.128220196 | 3.747003075 | 4.248695007 | 2.34E-05 | 0.000152 | 1.984006559 |
| MTR | -0.120449515 | 3.334988348 | -4.24682898 | 2.36E-05 | 0.000153 | 1.97628696 |
| RAD23B | 0.119317506 | 4.557813337 | 4.246222809 | 2.36E-05 | 0.000153 | 1.973779998 |
| MAPK8IP3 | -0.140262382 | 3.396724304 | -4.24584155 | 2.37E-05 | 0.000154 | 1.972203386 |
| ANKS1A | -0.133238632 | 3.61777356 | -4.24415062 | 2.39E-05 | 0.000155 | 1.965212539 |
| IWS1 | -0.09590944 | 3.80124762 | -4.24255951 | 2.40E-05 | 0.000156 | 1.958636885 |
| SLC6A13 | -0.24632973 | 1.871243615 | -4.24029831 | 2.43E-05 | 0.000157 | 1.949295967 |
| SLC39A14 | 0.156487655 | 3.591561267 | 4.239915315 | 2.43E-05 | 0.000157 | 1.947714305 |
| ARHGAP12 | -0.105294518 | 3.324984228 | -4.23940375 | 2.44E-05 | 0.000157 | 1.94560189 |
| TNRC6A | -0.101559613 | 3.458356355 | -4.23614742 | 2.47E-05 | 0.00016 | 1.932161363 |
| GHDC | -0.117666993 | 3.125362398 | -4.23592063 | 2.47E-05 | 0.00016 | 1.93122564 |
| DTWD1 | -0.095702202 | 2.501754331 | -4.23388565 | 2.50E-05 | 0.000161 | 1.922831741 |
| NISCH | -0.127503703 | 3.967557295 | -4.23192704 | 2.52E-05 | 0.000162 | 1.914756527 |
| KLHL20 | -0.090776299 | 2.963693236 | -4.23186114 | 2.52E-05 | 0.000162 | 1.914484911 |
| ADAM17 | 0.136844284 | 3.651031927 | 4.23167819 | 2.52E-05 | 0.000162 | 1.91373081 |
| PPM1M | -0.117302931 | 3.078242822 | -4.22798334 | 2.56E-05 | 0.000165 | 1.898507893 |
| ZNRF2 | 0.121422842 | 3.176955992 | 4.227851776 | 2.56E-05 | 0.000165 | 1.897966088 |
| STIM2 | -0.092470899 | 2.979282371 | -4.22645241 | 2.58E-05 | 0.000166 | 1.892204196 |
| GJA4 | -0.21971423 | 2.700581564 | -4.22588945 | 2.58E-05 | 0.000166 | 1.889886718 |
| AGBL2 | -0.159419735 | 1.846704766 | -4.22548234 | 2.59E-05 | 0.000166 | 1.888210976 |
| PXMP2 | 0.166451378 | 3.180244275 | 4.225314248 | 2.59E-05 | 0.000166 | 1.887519147 |
| NRG4 | 0.215968066 | 1.728851354 | 4.224729344 | 2.60E-05 | 0.000167 | 1.88511197 |
| MBTD1 | -0.110907373 | 3.005848308 | -4.22443021 | 2.60E-05 | 0.000167 | 1.883880993 |
| LRFN5 | -0.153953287 | 1.386486207 | -4.22330156 | 2.61E-05 | 0.000167 | 1.879237254 |
| CLDN5 | -0.192076803 | 2.05817777 | -4.22327273 | 2.61E-05 | 0.000167 | 1.879118665 |
| ST3GAL2 | -0.115958164 | 2.93739408 | -4.22205082 | 2.63E-05 | 0.000168 | 1.874092599 |
| RCOR3 | -0.110294122 | 3.450439409 | -4.22172144 | 2.63E-05 | 0.000168 | 1.872738003 |
| ZNF622 | 0.109972865 | 3.788857768 | 4.220849742 | 2.64E-05 | 0.000169 | 1.869153599 |
| USP21 | -0.09731478 | 3.869088157 | -4.220523 | 2.65E-05 | 0.000169 | 1.867810239 |
| ZFYVE27 | -0.108691459 | 3.443194046 | -4.21966431 | 2.66E-05 | 0.00017 | 1.864280286 |
| SRC | -0.133496383 | 3.387268842 | -4.2194172 | 2.66E-05 | 0.00017 | 1.863264568 |
| ATP1B2 | -0.260184267 | 2.028042582 | -4.21771075 | 2.68E-05 | 0.000171 | 1.856252038 |
| LYPD3 | 0.242248397 | 2.163005293 | 4.217593716 | 2.68E-05 | 0.000171 | 1.855771191 |
| CTR9 | -0.098074722 | 3.479217982 | -4.21489265 | 2.71E-05 | 0.000173 | 1.844677283 |
| EIF3F | -0.122287504 | 4.140381824 | -4.21344513 | 2.73E-05 | 0.000174 | 1.838734769 |
| ZDHHC3 | 0.098254086 | 3.631976949 | 4.213382778 | 2.73E-05 | 0.000174 | 1.838478853 |
| TSPAN18 | -0.176147121 | 2.580366161 | -4.21090508 | 2.76E-05 | 0.000175 | 1.828311931 |
| POLE4 | 0.134502504 | 3.427011616 | 4.210664671 | 2.76E-05 | 0.000176 | 1.827325739 |
| C2orf42 | -0.086933783 | 2.932842386 | -4.20966954 | 2.77E-05 | 0.000176 | 1.823244198 |
| NNMT | 0.32869681 | 3.996498115 | 4.209079178 | 2.78E-05 | 0.000177 | 1.820823256 |
| LPAR4 | -0.141621331 | 1.292060201 | -4.20864177 | 2.79E-05 | 0.000177 | 1.819029749 |
| POLE3 | 0.140832851 | 4.259487958 | 4.208321029 | 2.79E-05 | 0.000177 | 1.817714744 |
| SDHD | 0.146474698 | 4.309414903 | 4.204586443 | 2.84E-05 | 0.00018 | 1.802410338 |
| DPH3 | 0.121944327 | 3.194342784 | 4.204171844 | 2.84E-05 | 0.00018 | 1.800712116 |
| SEC13 | 0.106171845 | 4.29329459 | 4.20398419 | 2.84E-05 | 0.00018 | 1.799943527 |
| SLC41A2 | 0.144377032 | 2.702904028 | 4.202893886 | 2.86E-05 | 0.000181 | 1.795478538 |
| ZDHHC1 | -0.127955349 | 2.9084303 | -4.20265498 | 2.86E-05 | 0.000181 | 1.794500314 |
| PLAT | -0.26854981 | 3.881503461 | -4.20245498 | 2.86E-05 | 0.000181 | 1.793681441 |
| ZNF638 | -0.093222827 | 3.806234359 | -4.19984521 | 2.89E-05 | 0.000183 | 1.782999593 |
| NTN3 | -0.175471184 | 1.42760619 | -4.1979261 | 2.92E-05 | 0.000184 | 1.775148745 |
| TMEM168 | -0.093793027 | 3.008989591 | -4.19687844 | 2.93E-05 | 0.000185 | 1.770864366 |
| DAB2 | -0.159373269 | 3.208085843 | -4.19578445 | 2.95E-05 | 0.000186 | 1.766391609 |
| C1orf43 | 0.102237527 | 5.122060437 | 4.194846663 | 2.96E-05 | 0.000186 | 1.762558361 |
| ABCF2 | 0.108416144 | 3.865581828 | 4.19415736 | 2.97E-05 | 0.000187 | 1.759741347 |
| AZIN1 | 0.139709585 | 4.571174357 | 4.194088093 | 2.97E-05 | 0.000187 | 1.759458293 |
| INO80D | -0.089810061 | 2.843719761 | -4.19399308 | 2.97E-05 | 0.000187 | 1.759070047 |
| DNASE2 | 0.140347203 | 4.250736024 | 4.193379171 | 2.98E-05 | 0.000187 | 1.756561616 |
| CHODL | 0.27366636 | 3.043128774 | 4.19337912 | 2.98E-05 | 0.000187 | 1.756561408 |
| EYA4 | -0.26887133 | 2.255991697 | -4.19294139 | 2.98E-05 | 0.000187 | 1.754773053 |
| CXXC5 | 0.155716422 | 4.904061505 | 4.192598679 | 2.99E-05 | 0.000188 | 1.753373044 |
| P2RY2 | 0.190744314 | 2.582781698 | 4.19116928 | 3.01E-05 | 0.000189 | 1.747534964 |
| LRRFIP2 | 0.086578311 | 3.102865373 | 4.190231071 | 3.02E-05 | 0.000189 | 1.743704091 |
| RHPN2 | 0.188659499 | 3.963926717 | 4.189199613 | 3.03E-05 | 0.00019 | 1.739493424 |
| MAN2C1 | -0.115724329 | 3.457702952 | -4.18898011 | 3.03E-05 | 0.00019 | 1.738597492 |
| C3orf18 | -0.127929069 | 2.514255535 | -4.18545152 | 3.08E-05 | 0.000193 | 1.724201219 |
| TMEM141 | 0.158140993 | 3.798141106 | 4.185307726 | 3.08E-05 | 0.000193 | 1.723614806 |
| TTLL1 | -0.135370695 | 2.685334314 | -4.18434103 | 3.10E-05 | 0.000194 | 1.719672969 |
| FANCG | 0.13271782 | 3.325734444 | 4.182218738 | 3.12E-05 | 0.000195 | 1.711022122 |
| KLK7 | 0.313448729 | 5.036515193 | 4.180752339 | 3.14E-05 | 0.000196 | 1.705047293 |
| DDX25 | -0.232992698 | 1.355915109 | -4.18046809 | 3.15E-05 | 0.000197 | 1.703889352 |
| SCARF2 | -0.188119621 | 3.058952771 | -4.18029005 | 3.15E-05 | 0.000197 | 1.703164106 |
| P2RY8 | -0.148110196 | 1.779955072 | -4.17899705 | 3.17E-05 | 0.000198 | 1.697898064 |
| PAK1IP1 | 0.132007917 | 3.698859158 | 4.178459811 | 3.18E-05 | 0.000198 | 1.695710469 |
| COPS5 | 0.101523744 | 3.790835788 | 4.17834812 | 3.18E-05 | 0.000198 | 1.695255712 |
| PXMP4 | 0.140318497 | 2.797425544 | 4.178277254 | 3.18E-05 | 0.000198 | 1.694967179 |
| MRPS7 | 0.132371002 | 3.980175079 | 4.178079898 | 3.18E-05 | 0.000198 | 1.69416367 |
| SPG7 | -0.103937326 | 3.149996321 | -4.17789799 | 3.18E-05 | 0.000198 | 1.693423085 |
| CEP120 | -0.096834483 | 2.705209601 | -4.17763926 | 3.19E-05 | 0.000198 | 1.692369807 |
| FASTKD3 | 0.116407319 | 2.817679573 | 4.176624784 | 3.20E-05 | 0.000199 | 1.688240489 |
| RFXANK | 0.147227538 | 4.446389529 | 4.172621965 | 3.26E-05 | 0.000202 | 1.671956935 |
| SLC12A4 | -0.116299337 | 3.04328071 | -4.1715863 | 3.27E-05 | 0.000203 | 1.667746292 |
| GRK5 | -0.113459939 | 2.096991965 | -4.17113705 | 3.28E-05 | 0.000203 | 1.6659201 |
| EIF4B | -0.120883561 | 5.140611552 | -4.169665 | 3.30E-05 | 0.000204 | 1.659937655 |
| IL1R1 | -0.200844823 | 4.031463494 | -4.16783702 | 3.32E-05 | 0.000206 | 1.652511505 |
| GART | 0.110041739 | 3.717510603 | 4.166917645 | 3.34E-05 | 0.000207 | 1.648777734 |
| LMBR1L | -0.10413906 | 3.116290839 | -4.16576661 | 3.35E-05 | 0.000208 | 1.644104276 |
| SLC39A5 | -0.166375771 | 1.369336454 | -4.16531746 | 3.36E-05 | 0.000208 | 1.642280988 |
| PLP1 | -0.168527288 | 1.018095529 | -4.16505978 | 3.36E-05 | 0.000208 | 1.641235021 |
| SEC31B | -0.124537543 | 2.257824964 | -4.16487609 | 3.37E-05 | 0.000208 | 1.640489436 |
| PIGX | 0.129129099 | 3.156469209 | 4.164111412 | 3.38E-05 | 0.000209 | 1.637386017 |
| MEIS2 | -0.19947815 | 2.732459408 | -4.16128062 | 3.42E-05 | 0.000211 | 1.625902133 |
| IMPA2 | 0.165411021 | 3.355084284 | 4.160708112 | 3.43E-05 | 0.000211 | 1.62358051 |
| NCOR1 | -0.096840076 | 3.695015579 | -4.1589862 | 3.45E-05 | 0.000213 | 1.61659972 |
| OS9 | -0.114362502 | 4.677647711 | -4.15849202 | 3.46E-05 | 0.000213 | 1.614596777 |
| PDE3A | -0.189595351 | 2.884460629 | -4.15842155 | 3.46E-05 | 0.000213 | 1.614311186 |
| PRDM5 | -0.11472937 | 1.879311509 | -4.15817673 | 3.47E-05 | 0.000213 | 1.61331901 |
| TIMM17A | 0.102235193 | 3.795008417 | 4.156959353 | 3.48E-05 | 0.000214 | 1.608386287 |
| RSAD1 | -0.116536674 | 3.321448774 | -4.15531122 | 3.51E-05 | 0.000216 | 1.601710371 |
| AZI2 | -0.085190768 | 2.932903335 | -4.15310634 | 3.54E-05 | 0.000218 | 1.592783301 |
| RB1 | -0.142303028 | 3.461783867 | -4.15228623 | 3.56E-05 | 0.000218 | 1.589464044 |
| IRF8 | -0.163924664 | 2.51698813 | -4.15208306 | 3.56E-05 | 0.000218 | 1.588641844 |
| LYN | 0.147101535 | 3.401852717 | 4.148678113 | 3.61E-05 | 0.000221 | 1.574868289 |
| FABP5 | 0.18694196 | 3.452511612 | 4.148420178 | 3.61E-05 | 0.000222 | 1.573825348 |
| DNMT3A | -0.142227554 | 3.528706448 | -4.14608809 | 3.65E-05 | 0.000224 | 1.564398553 |
| PRDX2 | 0.17686668 | 5.095619671 | 4.145709077 | 3.66E-05 | 0.000224 | 1.562866988 |
| TMCC3 | -0.228622319 | 2.731127409 | -4.1452425 | 3.66E-05 | 0.000224 | 1.560981764 |
| C5orf46 | 0.235823783 | 1.722608837 | 4.144924338 | 3.67E-05 | 0.000224 | 1.559696337 |
| G3BP1 | 0.101786717 | 4.162111642 | 4.143482275 | 3.69E-05 | 0.000226 | 1.553871365 |
| PPP1R10 | -0.099772546 | 3.913018487 | -4.14347795 | 3.69E-05 | 0.000226 | 1.553853882 |
| TLR4 | -0.149630996 | 2.343884486 | -4.14157794 | 3.72E-05 | 0.000227 | 1.546182108 |
| MRPS23 | 0.128639742 | 3.399243444 | 4.14135872 | 3.73E-05 | 0.000227 | 1.545297189 |
| IL34 | -0.199633634 | 2.19214417 | -4.14068846 | 3.74E-05 | 0.000228 | 1.542591809 |
| GHITM | 0.130523974 | 4.750095916 | 4.140413889 | 3.74E-05 | 0.000228 | 1.541483658 |
| UCP2 | 0.196265879 | 5.369850692 | 4.140046993 | 3.75E-05 | 0.000228 | 1.540003015 |
| PFKM | -0.140372874 | 3.684200795 | -4.13967668 | 3.75E-05 | 0.000228 | 1.538508733 |
| SLC4A2 | 0.137479665 | 4.284025068 | 4.138971231 | 3.76E-05 | 0.000229 | 1.535662419 |
| BTN3A1 | -0.145530476 | 3.654597681 | -4.13860851 | 3.77E-05 | 0.000229 | 1.534199103 |
| TRAF3IP3 | -0.133255215 | 2.03831674 | -4.1360313 | 3.81E-05 | 0.000232 | 1.523805639 |
| ZNF214 | -0.140967038 | 1.879820829 | -4.13473554 | 3.83E-05 | 0.000233 | 1.518582441 |
| CDC25A | 0.149897988 | 2.47999096 | 4.134018566 | 3.85E-05 | 0.000233 | 1.515692972 |
| ASB6 | 0.1112953 | 3.316511198 | 4.133166232 | 3.86E-05 | 0.000234 | 1.512258642 |
| POLR2G | 0.111488308 | 4.34691938 | 4.132480337 | 3.87E-05 | 0.000235 | 1.50949545 |
| STK36 | -0.146479153 | 3.603117003 | -4.13239966 | 3.87E-05 | 0.000235 | 1.509170466 |
| EIF3A | -0.117381432 | 4.597071513 | -4.12899226 | 3.93E-05 | 0.000238 | 1.495450263 |
| TDRD7 | 0.136842219 | 3.013612815 | 4.128476528 | 3.94E-05 | 0.000238 | 1.493374576 |
| REPS1 | -0.09925999 | 3.117711117 | -4.12843944 | 3.94E-05 | 0.000238 | 1.493225326 |
| ASH2L | -0.119638944 | 3.503563478 | -4.1254871 | 3.99E-05 | 0.000241 | 1.481347869 |
| GAS2L3 | 0.127699158 | 2.477701253 | 4.1239912 | 4.01E-05 | 0.000243 | 1.475332894 |
| WWP2 | -0.102281294 | 3.212258523 | -4.11990288 | 4.08E-05 | 0.000247 | 1.458904663 |
| NDUFB7 | 0.165781876 | 5.33393441 | 4.115777603 | 4.16E-05 | 0.000251 | 1.442343927 |
| FBP1 | 0.227937253 | 2.72829405 | 4.115619504 | 4.16E-05 | 0.000251 | 1.441709566 |
| FYTTD1 | 0.132919975 | 3.862656683 | 4.113930853 | 4.19E-05 | 0.000253 | 1.434935439 |
| MYOM2 | -0.170263607 | 1.189782482 | -4.11212396 | 4.22E-05 | 0.000255 | 1.427689961 |
| TRPT1 | 0.113725823 | 3.799660662 | 4.11060422 | 4.25E-05 | 0.000256 | 1.421598322 |
| KIF13B | -0.115359209 | 2.789742305 | -4.11054505 | 4.25E-05 | 0.000256 | 1.421361205 |
| RYR2 | -0.178717362 | 2.112696872 | -4.1102197 | 4.26E-05 | 0.000256 | 1.420057383 |
| ABCC10 | -0.113623594 | 3.184473469 | -4.11016489 | 4.26E-05 | 0.000256 | 1.419837743 |
| RPS6KA3 | 0.120851146 | 3.284220011 | 4.105921459 | 4.34E-05 | 0.000261 | 1.402841928 |
| EMILIN3 | -0.189134163 | 1.516517533 | -4.10468273 | 4.36E-05 | 0.000262 | 1.397883768 |
| PTEN | -0.12592935 | 3.726097306 | -4.10265118 | 4.40E-05 | 0.000264 | 1.389755375 |
| PSMB8 | 0.200911567 | 4.500793328 | 4.09842681 | 4.48E-05 | 0.000269 | 1.37286585 |
| ZMAT1 | -0.155213495 | 2.341998566 | -4.09839807 | 4.48E-05 | 0.000269 | 1.372751001 |
| TMED5 | 0.113539125 | 3.235904763 | 4.098197923 | 4.48E-05 | 0.000269 | 1.371951216 |
| ZNF205 | -0.114914315 | 3.269375163 | -4.0976929 | 4.49E-05 | 0.000269 | 1.369933307 |
| PTGES2 | 0.123305354 | 3.960344292 | 4.096776603 | 4.51E-05 | 0.00027 | 1.366272703 |
| PALLD | -0.179932746 | 3.71465261 | -4.09625185 | 4.52E-05 | 0.00027 | 1.36417666 |
| FDX1 | 0.119686475 | 3.14850794 | 4.096127257 | 4.52E-05 | 0.00027 | 1.36367905 |
| ACVR2A | -0.10777931 | 2.790217851 | -4.09523238 | 4.54E-05 | 0.000271 | 1.360105343 |
| ARPC1A | 0.123424304 | 4.813445389 | 4.094671575 | 4.55E-05 | 0.000272 | 1.357866124 |
| IFI27L1 | 0.134788028 | 3.007491484 | 4.092076925 | 4.60E-05 | 0.000275 | 1.347509957 |
| SMAP1 | 0.100189123 | 3.21304344 | 4.090850321 | 4.62E-05 | 0.000276 | 1.342616364 |
| ZNF678 | -0.114130466 | 2.40699015 | -4.09035745 | 4.63E-05 | 0.000276 | 1.340650413 |
| BAMBI | -0.263943635 | 1.914127023 | -4.08757793 | 4.69E-05 | 0.000279 | 1.329567956 |
| TIMELESS | 0.149350376 | 3.453438319 | 4.08712742 | 4.70E-05 | 0.00028 | 1.327772375 |
| SF3B1 | -0.106879436 | 4.836790833 | -4.08416556 | 4.76E-05 | 0.000283 | 1.315972171 |
| REEP1 | -0.1920969 | 1.724987961 | -4.08411554 | 4.76E-05 | 0.000283 | 1.31577295 |
| IL1RN | 0.187397628 | 2.700387487 | 4.083908883 | 4.76E-05 | 0.000283 | 1.31494994 |
| MERTK | -0.134276563 | 2.903365654 | -4.08380112 | 4.76E-05 | 0.000283 | 1.314520785 |
| LRRC4C | -0.16048992 | 1.894727823 | -4.08231172 | 4.79E-05 | 0.000285 | 1.308590585 |
| POLR2B | 0.114513451 | 4.013550627 | 4.081895625 | 4.80E-05 | 0.000285 | 1.306934233 |
| ARMC9 | -0.095642865 | 2.814982719 | -4.0818178 | 4.80E-05 | 0.000285 | 1.306624445 |
| ZBTB22 | -0.107301026 | 3.4259447 | -4.08025364 | 4.83E-05 | 0.000287 | 1.300399566 |
| EGR2 | -0.235215103 | 2.763755967 | -4.07999969 | 4.84E-05 | 0.000287 | 1.299389159 |
| CEP68 | -0.099114164 | 3.127642486 | -4.07813621 | 4.88E-05 | 0.000289 | 1.291976513 |
| POLD1 | 0.134573391 | 3.473805606 | 4.077253405 | 4.90E-05 | 0.00029 | 1.288466014 |
| CCT6A | 0.095526813 | 4.767500883 | 4.077222867 | 4.90E-05 | 0.00029 | 1.288344591 |
| IL22RA1 | -0.1969775 | 1.797743121 | -4.07720869 | 4.90E-05 | 0.00029 | 1.288288238 |
| MTFR1 | 0.116082014 | 3.023550435 | 4.076915601 | 4.90E-05 | 0.00029 | 1.287122916 |
| L2HGDH | 0.112456887 | 2.507149178 | 4.076095694 | 4.92E-05 | 0.000291 | 1.283863446 |
| ODF3L1 | -0.146200533 | 1.175038933 | -4.07542537 | 4.93E-05 | 0.000292 | 1.281199085 |
| PPAT | 0.119938047 | 3.064881002 | 4.074963799 | 4.94E-05 | 0.000292 | 1.279364735 |
| MRVI1 | -0.173970911 | 2.666672752 | -4.07421804 | 4.96E-05 | 0.000293 | 1.27640137 |
| CXCL3 | 0.254397756 | 1.849387272 | 4.072592531 | 4.99E-05 | 0.000294 | 1.269944065 |
| HSD17B11 | -0.158331247 | 3.708184217 | -4.07255696 | 4.99E-05 | 0.000294 | 1.269802804 |
| CAMK1D | -0.162875279 | 3.104958651 | -4.07201261 | 5.01E-05 | 0.000295 | 1.267640935 |
| GJB6 | 0.202702118 | 1.195907849 | 4.070114494 | 5.05E-05 | 0.000297 | 1.260104902 |
| GBP1 | 0.204796022 | 4.179206005 | 4.069345868 | 5.06E-05 | 0.000298 | 1.257054215 |
| KIF3C | -0.144119822 | 3.011067501 | -4.06915076 | 5.07E-05 | 0.000298 | 1.256279902 |
| CLDN3 | 0.203563694 | 5.96193738 | 4.068329697 | 5.08E-05 | 0.000299 | 1.253021874 |
| ANKRA2 | -0.113658053 | 3.037957598 | -4.06804863 | 5.09E-05 | 0.000299 | 1.251906705 |
| HECTD2 | -0.092268422 | 2.396671018 | -4.06804021 | 5.09E-05 | 0.000299 | 1.251873312 |
| C10orf82 | -0.209529689 | 1.579316795 | -4.06762945 | 5.10E-05 | 0.000299 | 1.25024374 |
| OAS1 | 0.238273491 | 3.89196808 | 4.067072835 | 5.11E-05 | 0.0003 | 1.248035781 |
| UBE2E1 | 0.115831458 | 4.59181346 | 4.065251066 | 5.15E-05 | 0.000302 | 1.240811298 |
| PARP12 | 0.153176612 | 3.860895202 | 4.063220474 | 5.20E-05 | 0.000305 | 1.232762394 |
| LEF1 | -0.206207415 | 3.165754022 | -4.0627562 | 5.21E-05 | 0.000305 | 1.230922637 |
| ETV3 | -0.097740101 | 3.206553962 | -4.06130631 | 5.24E-05 | 0.000307 | 1.225178537 |
| CSGALNACT1 | -0.128354799 | 2.015274702 | -4.05857433 | 5.30E-05 | 0.00031 | 1.214360558 |
| FAM124B | -0.137770809 | 1.239855467 | -4.05820014 | 5.31E-05 | 0.00031 | 1.212879405 |
| GRK6 | 0.104305027 | 3.733328647 | 4.055883691 | 5.36E-05 | 0.000313 | 1.203713174 |
| VAMP7 | 0.11958317 | 3.602696949 | 4.055640058 | 5.36E-05 | 0.000313 | 1.202749409 |
| RAB1A | 0.08609898 | 4.812626922 | 4.053376321 | 5.42E-05 | 0.000316 | 1.193797176 |
| PIK3CD | -0.132245189 | 2.887696151 | -4.05261232 | 5.43E-05 | 0.000317 | 1.190776947 |
| OBSCN | -0.134381194 | 3.210390904 | -4.05224332 | 5.44E-05 | 0.000317 | 1.189318391 |
| SSNA1 | 0.11799044 | 4.175445261 | 4.051319694 | 5.46E-05 | 0.000319 | 1.185668183 |
| PHLDB2 | -0.155017216 | 2.413355002 | -4.05087189 | 5.47E-05 | 0.000319 | 1.183898716 |
| RHOA | 0.100718018 | 5.642162182 | 4.050721785 | 5.48E-05 | 0.000319 | 1.183305651 |
| CACNB2 | -0.116803901 | 1.545801914 | -4.05003956 | 5.49E-05 | 0.00032 | 1.180610368 |
| INTS8 | 0.099819972 | 3.580544164 | 4.049977629 | 5.49E-05 | 0.00032 | 1.180365716 |
| CORO2B | -0.190491638 | 2.268965498 | -4.04871402 | 5.52E-05 | 0.000321 | 1.175374773 |
| ST14 | 0.17179182 | 4.790208061 | 4.048671551 | 5.52E-05 | 0.000321 | 1.175207066 |
| DGAT1 | 0.159794524 | 3.94749615 | 4.04639322 | 5.58E-05 | 0.000324 | 1.166212144 |
| DMBX1 | 0.244635729 | 1.519386191 | 4.04592668 | 5.59E-05 | 0.000324 | 1.164370838 |
| SLC22A18AS | 0.216000866 | 1.828956046 | 4.045193045 | 5.61E-05 | 0.000325 | 1.161475792 |
| EHF | 0.228103989 | 3.981066383 | 4.04482621 | 5.61E-05 | 0.000326 | 1.160028388 |
| PRPSAP2 | -0.089276124 | 3.041574364 | -4.04182634 | 5.69E-05 | 0.000329 | 1.148196728 |
| IDH3A | 0.114683954 | 2.946970652 | 4.041789216 | 5.69E-05 | 0.000329 | 1.148050375 |
| NR4A1 | -0.274626081 | 3.6063996 | -4.04161335 | 5.69E-05 | 0.00033 | 1.147357037 |
| ZNF276 | -0.106393567 | 2.812072834 | -4.0413087 | 5.70E-05 | 0.00033 | 1.146155986 |
| REEP4 | 0.121244824 | 3.641933322 | 4.041233855 | 5.70E-05 | 0.00033 | 1.145860955 |
| PPM1A | 0.090312452 | 3.360808688 | 4.04025934 | 5.72E-05 | 0.000331 | 1.142019791 |
| RHOT1 | -0.092312315 | 3.069255072 | -4.03969028 | 5.74E-05 | 0.000332 | 1.139777192 |
| POR | 0.134387291 | 4.220847913 | 4.038898963 | 5.76E-05 | 0.000332 | 1.136659212 |
| RINT1 | 0.099899113 | 3.103531591 | 4.038770342 | 5.76E-05 | 0.000332 | 1.13615247 |
| ELP4 | 0.11435483 | 3.053942682 | 4.03832322 | 5.77E-05 | 0.000333 | 1.13439102 |
| ARAP3 | -0.157677634 | 2.922423608 | -4.03810333 | 5.78E-05 | 0.000333 | 1.133524818 |
| MYO15A | -0.133609896 | 1.847578142 | -4.03717123 | 5.80E-05 | 0.000334 | 1.129853588 |
| VWCE | -0.236097896 | 2.558907647 | -4.03709334 | 5.80E-05 | 0.000334 | 1.129546823 |
| KISS1R | 0.185742785 | 1.288060325 | 4.036774614 | 5.81E-05 | 0.000334 | 1.128291695 |
| STRBP | 0.10541957 | 3.350177542 | 4.036762013 | 5.81E-05 | 0.000334 | 1.128242075 |
| CANX | 0.121424753 | 5.506720437 | 4.03411603 | 5.87E-05 | 0.000338 | 1.117825911 |
| TMOD1 | 0.249097101 | 3.742265842 | 4.033914281 | 5.88E-05 | 0.000338 | 1.117031979 |
| PNMT | -0.231331645 | 1.514680051 | -4.03350172 | 5.89E-05 | 0.000338 | 1.115408549 |
| PPCS | 0.118923556 | 4.183120578 | 4.03261917 | 5.91E-05 | 0.000339 | 1.111936311 |
| FBXL7 | -0.172759711 | 2.76358198 | -4.0291385 | 6.00E-05 | 0.000344 | 1.098249319 |
| ANKRD11 | -0.105219863 | 3.711122995 | -4.02908263 | 6.00E-05 | 0.000344 | 1.098029729 |
| BBS2 | -0.103546962 | 3.102287477 | -4.02849884 | 6.01E-05 | 0.000345 | 1.095735273 |
| CD160 | -0.12766402 | 1.081966385 | -4.02814241 | 6.02E-05 | 0.000345 | 1.094334544 |
| CYP4Z1 | 0.235665939 | 1.691309595 | 4.026099857 | 6.07E-05 | 0.000348 | 1.086309908 |
| CAD | -0.132900754 | 3.905922043 | -4.02580433 | 6.08E-05 | 0.000348 | 1.085149202 |
| C11orf45 | -0.128002133 | 2.04741891 | -4.02469893 | 6.11E-05 | 0.00035 | 1.080808341 |
| IL10RA | -0.170276483 | 2.852632477 | -4.02457875 | 6.11E-05 | 0.00035 | 1.080336439 |
| SMPD3 | -0.16414324 | 1.660444969 | -4.02441745 | 6.12E-05 | 0.00035 | 1.079703165 |
| NDUFV3 | 0.112180455 | 3.737169706 | 4.023660116 | 6.14E-05 | 0.000351 | 1.076730006 |
| GTPBP10 | 0.082435538 | 2.650017672 | 4.022487862 | 6.17E-05 | 0.000352 | 1.072129027 |
| UBE3B | -0.095962677 | 3.211176125 | -4.02220451 | 6.17E-05 | 0.000352 | 1.071017103 |
| PCM1 | -0.117633482 | 3.634648333 | -4.02212133 | 6.18E-05 | 0.000352 | 1.070690706 |
| MRPL48 | 0.123324196 | 3.540989344 | 4.022059956 | 6.18E-05 | 0.000352 | 1.070449864 |
| HDAC4 | -0.1041409 | 2.861962246 | -4.02199499 | 6.18E-05 | 0.000352 | 1.070194962 |
| BBS1 | -0.083885478 | 2.443352543 | -4.02065562 | 6.21E-05 | 0.000354 | 1.064940251 |
| SPIRE2 | -0.161431987 | 2.390225814 | -4.02047609 | 6.22E-05 | 0.000354 | 1.064236074 |
| HMGN2 | 0.118913215 | 5.393267356 | 4.016811866 | 6.31E-05 | 0.000359 | 1.049869708 |
| PROK2 | 0.271694888 | 1.324522466 | 4.015603489 | 6.35E-05 | 0.000361 | 1.045134802 |
| HTRA1 | -0.214112758 | 4.709392605 | -4.01475844 | 6.37E-05 | 0.000362 | 1.04182437 |
| S100B | -0.216443107 | 1.494074716 | -4.01256212 | 6.43E-05 | 0.000365 | 1.03322362 |
| ATN1 | -0.140221388 | 5.386649292 | -4.01215965 | 6.44E-05 | 0.000365 | 1.03164802 |
| ATAD2B | -0.088381377 | 2.565922815 | -4.01213248 | 6.44E-05 | 0.000365 | 1.031541669 |
| MED16 | -0.110221464 | 3.691251211 | -4.01195707 | 6.44E-05 | 0.000365 | 1.03085504 |
| CACNA2D4 | -0.099112104 | 1.954262639 | -4.01130975 | 6.46E-05 | 0.000366 | 1.028321369 |
| CDKN2B | 0.192496488 | 2.904229251 | 4.010826324 | 6.47E-05 | 0.000367 | 1.026429478 |
| PTPN21 | -0.107503489 | 2.753925128 | -4.01074206 | 6.48E-05 | 0.000367 | 1.02609972 |
| ZNF319 | -0.108199853 | 2.770516871 | -4.00954948 | 6.51E-05 | 0.000368 | 1.021433572 |
| GRHPR | 0.10155925 | 3.980737818 | 4.009072453 | 6.52E-05 | 0.000369 | 1.019567492 |
| MAPK9 | 0.100668465 | 3.131951844 | 4.008395698 | 6.54E-05 | 0.00037 | 1.01692048 |
| RNF146 | -0.104813592 | 3.458588621 | -4.00833049 | 6.54E-05 | 0.00037 | 1.016665442 |
| SIX4 | 0.131090155 | 3.018579129 | 4.006793795 | 6.58E-05 | 0.000372 | 1.010656635 |
| PTK6 | 0.2472929 | 2.023959233 | 4.006495448 | 6.59E-05 | 0.000372 | 1.009490292 |
| CDC23 | 0.097907484 | 3.432986214 | 4.004858006 | 6.64E-05 | 0.000375 | 1.003090454 |
| PRSS16 | 0.210693371 | 2.646170769 | 4.00223718 | 6.71E-05 | 0.000378 | 0.992852406 |
| MREG | 0.141396931 | 3.026730874 | 4.001012574 | 6.74E-05 | 0.00038 | 0.988070808 |
| SPATA7 | -0.09917091 | 2.388168731 | -3.99977361 | 6.78E-05 | 0.000382 | 0.983234586 |
| CROT | -0.115193119 | 2.465781286 | -3.99801185 | 6.83E-05 | 0.000385 | 0.976360168 |
| GTF3A | 0.115945363 | 4.490663928 | 3.997406754 | 6.85E-05 | 0.000385 | 0.97399976 |
| FEM1C | 0.101843299 | 3.156752553 | 3.996641214 | 6.87E-05 | 0.000386 | 0.971013965 |
| SENP5 | 0.105378446 | 3.412906365 | 3.996198931 | 6.88E-05 | 0.000387 | 0.969289207 |
| CLSPN | 0.136391499 | 2.416441328 | 3.995310921 | 6.91E-05 | 0.000388 | 0.965826816 |
| PDE8B | -0.155398664 | 1.62064854 | -3.99482103 | 6.92E-05 | 0.000389 | 0.963917011 |
| PLD3 | -0.163612334 | 4.826307803 | -3.99327789 | 6.96E-05 | 0.000391 | 0.957902757 |
| AUH | 0.110244726 | 3.265198701 | 3.991834733 | 7.01E-05 | 0.000393 | 0.952280183 |
| BTG3 | 0.180057378 | 4.321681908 | 3.990482995 | 7.05E-05 | 0.000395 | 0.94701558 |
| FUT2 | 0.232005069 | 2.370713586 | 3.990168782 | 7.05E-05 | 0.000395 | 0.945792062 |
| RNF14 | 0.092387898 | 3.343436609 | 3.990135561 | 7.06E-05 | 0.000395 | 0.945662707 |
| CTSB | 0.151148403 | 5.991813531 | 3.989951675 | 7.06E-05 | 0.000395 | 0.944946722 |
| INTS9 | -0.105016349 | 2.872837455 | -3.98919611 | 7.08E-05 | 0.000397 | 0.942005167 |
| GABRP | 0.287007688 | 2.348113404 | 3.98741038 | 7.14E-05 | 0.000399 | 0.935055118 |
| AP3B1 | -0.09100669 | 3.400754614 | -3.98575408 | 7.19E-05 | 0.000402 | 0.928611529 |
| TPST2 | 0.128261982 | 3.066287539 | 3.985683845 | 7.19E-05 | 0.000402 | 0.928338331 |
| CASD1 | -0.093533149 | 2.797651371 | -3.98560707 | 7.19E-05 | 0.000402 | 0.928039699 |
| NKD1 | -0.137899533 | 2.356727656 | -3.98333044 | 7.26E-05 | 0.000405 | 0.919187381 |
| PIGO | 0.108553316 | 3.351388787 | 3.982754897 | 7.28E-05 | 0.000406 | 0.91695022 |
| HOXD8 | 0.258509755 | 3.000280468 | 3.982169575 | 7.29E-05 | 0.000407 | 0.914675391 |
| FANCD2 | 0.110257888 | 2.837806928 | 3.981266485 | 7.32E-05 | 0.000408 | 0.911166207 |
| DERA | 0.14523032 | 3.682779779 | 3.980988122 | 7.33E-05 | 0.000408 | 0.910084711 |
| MRPL41 | 0.151176083 | 3.983750305 | 3.979838939 | 7.36E-05 | 0.00041 | 0.905620688 |
| RAD52 | -0.11915914 | 2.79975392 | -3.97715968 | 7.45E-05 | 0.000414 | 0.895217901 |
| MAP1B | -0.181769699 | 3.243583268 | -3.97669855 | 7.46E-05 | 0.000415 | 0.893428178 |
| SQSTM1 | 0.140546726 | 4.703213135 | 3.97555223 | 7.50E-05 | 0.000417 | 0.888979915 |
| RUFY3 | -0.101879122 | 2.94766025 | -3.9739558 | 7.55E-05 | 0.000419 | 0.882787103 |
| MORN4 | -0.130983097 | 2.690058096 | -3.97389698 | 7.55E-05 | 0.000419 | 0.882558988 |
| DACT3 | -0.146164579 | 2.014302569 | -3.97373556 | 7.55E-05 | 0.000419 | 0.881932956 |
| MB | 0.201164799 | 1.495423468 | 3.97301311 | 7.57E-05 | 0.00042 | 0.879131397 |
| AR | -0.227289764 | 2.972538049 | -3.9706925 | 7.65E-05 | 0.000424 | 0.870135771 |
| ADAMTS19 | -0.188586297 | 1.095426597 | -3.97064213 | 7.65E-05 | 0.000424 | 0.869940573 |
| BMP4 | -0.209089469 | 2.491134732 | -3.96950877 | 7.69E-05 | 0.000426 | 0.86554909 |
| LIPG | 0.170405228 | 1.939051785 | 3.969450911 | 7.69E-05 | 0.000426 | 0.865324942 |
| STYK1 | 0.168587419 | 1.860880412 | 3.968825819 | 7.71E-05 | 0.000427 | 0.862903436 |
| MS4A1 | -0.195649927 | 1.407744902 | -3.96570583 | 7.81E-05 | 0.000432 | 0.850822609 |
| DCUN1D5 | 0.120366994 | 3.755044296 | 3.965662476 | 7.81E-05 | 0.000432 | 0.850654823 |
| CCDC127 | 0.099155428 | 2.940715895 | 3.964226792 | 7.86E-05 | 0.000434 | 0.845098914 |
| CSF2RA | -0.153366668 | 2.35907203 | -3.96386792 | 7.87E-05 | 0.000435 | 0.843710443 |
| DPM1 | 0.109429638 | 4.474393853 | 3.963568486 | 7.88E-05 | 0.000435 | 0.842552011 |
| IFRD1 | 0.110483949 | 3.305844628 | 3.963325436 | 7.88E-05 | 0.000435 | 0.841611784 |
| INO80 | -0.094928646 | 3.234827581 | -3.96315669 | 7.89E-05 | 0.000435 | 0.840959016 |
| RAP2C | 0.106835668 | 3.242322151 | 3.963049478 | 7.89E-05 | 0.000435 | 0.840544323 |
| ZNF213 | -0.10638704 | 2.9998062 | -3.96286199 | 7.90E-05 | 0.000435 | 0.839819124 |
| BIK | 0.239173904 | 3.145993479 | 3.962810197 | 7.90E-05 | 0.000435 | 0.839618793 |
| FBXL14 | -0.141481372 | 3.352247323 | -3.96093589 | 7.96E-05 | 0.000438 | 0.832370912 |
| KCNA5 | -0.161148128 | 1.301022373 | -3.95988309 | 8.00E-05 | 0.00044 | 0.828301236 |
| BBS12 | -0.123287685 | 2.228463598 | -3.95919957 | 8.02E-05 | 0.000441 | 0.825659622 |
| MRPS28 | 0.127589281 | 3.32515518 | 3.959155708 | 8.02E-05 | 0.000441 | 0.825490118 |
| LRCH1 | -0.102574715 | 2.768380281 | -3.9590826 | 8.02E-05 | 0.000441 | 0.825207621 |
| DPY19L2 | -0.128438561 | 1.893750276 | -3.95879473 | 8.03E-05 | 0.000441 | 0.824095231 |
| TGFBR3 | -0.1615582 | 2.93043085 | -3.95839145 | 8.05E-05 | 0.000442 | 0.822536999 |
| MAPK4 | -0.15357634 | 1.191010534 | -3.9577424 | 8.07E-05 | 0.000443 | 0.820029496 |
| CD1E | -0.191891409 | 1.326760176 | -3.95708543 | 8.09E-05 | 0.000444 | 0.817491818 |
| DYNC2LI1 | -0.106913339 | 2.982808636 | -3.95686554 | 8.10E-05 | 0.000444 | 0.8166425 |
| CCPG1 | -0.109797603 | 2.552113481 | -3.95497586 | 8.16E-05 | 0.000447 | 0.809345864 |
| ARF5 | 0.125505758 | 4.914566568 | 3.954383471 | 8.18E-05 | 0.000448 | 0.807059151 |
| EIF4A3 | 0.100453524 | 4.299921614 | 3.95145814 | 8.28E-05 | 0.000453 | 0.795771831 |
| PSMB5 | 0.119458824 | 4.717677817 | 3.950520455 | 8.31E-05 | 0.000455 | 0.792155512 |
| MAPK13 | 0.149005815 | 3.282665704 | 3.950167561 | 8.32E-05 | 0.000455 | 0.790794738 |
| LGSN | -0.275711036 | 1.439934111 | -3.94715517 | 8.43E-05 | 0.000461 | 0.779183641 |
| ADAM15 | 0.133916852 | 4.144298059 | 3.946723501 | 8.44E-05 | 0.000461 | 0.777520521 |
| CHD9 | -0.085144458 | 3.054680582 | -3.94665779 | 8.45E-05 | 0.000461 | 0.777267346 |
| RPS6KC1 | -0.093875707 | 3.16963608 | -3.94597499 | 8.47E-05 | 0.000462 | 0.774637082 |
| TBC1D9 | -0.116259497 | 3.124494223 | -3.94593167 | 8.47E-05 | 0.000462 | 0.7744702 |
| RBBP7 | 0.123216882 | 4.366675385 | 3.944672053 | 8.51E-05 | 0.000464 | 0.7696191 |
| SPINK5 | -0.206277756 | 2.218933004 | -3.94396574 | 8.54E-05 | 0.000465 | 0.766899577 |
| BLCAP | -0.106642021 | 3.953673783 | -3.943392 | 8.56E-05 | 0.000466 | 0.764690814 |
| CYYR1 | -0.264311781 | 2.683976961 | -3.94173241 | 8.62E-05 | 0.000469 | 0.758303637 |
| KRT18 | 0.16527671 | 6.038259519 | 3.941184395 | 8.64E-05 | 0.00047 | 0.756195078 |
| VLDLR | 0.163943147 | 2.743649161 | 3.940668466 | 8.66E-05 | 0.000471 | 0.754210244 |
| CPSF2 | 0.092709919 | 3.240575361 | 3.938981518 | 8.72E-05 | 0.000474 | 0.747722131 |
| CKAP5 | 0.121033387 | 4.099275706 | 3.938951799 | 8.72E-05 | 0.000474 | 0.747607855 |
| SCOC | 0.136205781 | 3.653956841 | 3.938727784 | 8.72E-05 | 0.000474 | 0.746746488 |
| TKT | 0.137592745 | 4.406262451 | 3.938237195 | 8.74E-05 | 0.000474 | 0.744860275 |
| BATF2 | 0.190176203 | 2.485632276 | 3.938110055 | 8.75E-05 | 0.000474 | 0.744371484 |
| TFAP4 | -0.098585749 | 3.030009305 | -3.93780173 | 8.76E-05 | 0.000475 | 0.743186183 |
| OMD | -0.235909672 | 1.70179509 | -3.93602596 | 8.82E-05 | 0.000478 | 0.736361352 |
| DHDH | 0.1762743 | 1.563543152 | 3.935404095 | 8.84E-05 | 0.000479 | 0.733972055 |
| GALC | -0.112842122 | 2.958728296 | -3.9327704 | 8.94E-05 | 0.000484 | 0.723857022 |
| CHN2 | -0.120296996 | 2.173964862 | -3.93202133 | 8.97E-05 | 0.000485 | 0.720981312 |
| NDUFV1 | 0.110022893 | 4.669560111 | 3.931088056 | 9.00E-05 | 0.000487 | 0.7173992 |
| IL1A | 0.166421206 | 1.210226418 | 3.929187433 | 9.07E-05 | 0.000491 | 0.710106734 |
| TULP3 | -0.123119214 | 3.791914554 | -3.92865575 | 9.09E-05 | 0.000491 | 0.708067339 |
| SPAG9 | -0.101270659 | 3.222990448 | -3.92858538 | 9.10E-05 | 0.000491 | 0.707797438 |
| ZNF384 | -0.105660383 | 3.818129427 | -3.92852791 | 9.10E-05 | 0.000491 | 0.707577031 |
| FUNDC2 | 0.116255702 | 3.698482535 | 3.927356914 | 9.14E-05 | 0.000493 | 0.703086486 |
| CCNDBP1 | -0.110294502 | 3.318368825 | -3.92682942 | 9.16E-05 | 0.000494 | 0.701064077 |
| CRTAP | -0.10578467 | 4.372927369 | -3.92542477 | 9.21E-05 | 0.000497 | 0.695679935 |
| SLC11A1 | 0.127307931 | 2.616472426 | 3.925082641 | 9.23E-05 | 0.000497 | 0.694368815 |
| GIGYF2 | -0.085537215 | 3.50426264 | -3.92483426 | 9.24E-05 | 0.000497 | 0.693417037 |
| ADCY7 | -0.11842854 | 2.669445114 | -3.92471139 | 9.24E-05 | 0.000497 | 0.692946223 |
| TELO2 | -0.117670417 | 3.452109496 | -3.92421741 | 9.26E-05 | 0.000498 | 0.691053512 |
| BANF1 | 0.120779679 | 5.169567614 | 3.924164288 | 9.26E-05 | 0.000498 | 0.690850001 |
| PTX3 | 0.294429513 | 2.689113473 | 3.923444877 | 9.29E-05 | 0.000499 | 0.688094027 |
| TCEAL7 | -0.190891237 | 1.662241122 | -3.92319859 | 9.30E-05 | 0.0005 | 0.687150656 |
| ISL1 | -0.152511645 | 1.296191599 | -3.92161718 | 9.36E-05 | 0.000502 | 0.681094533 |
| FBXL13 | -0.106453377 | 1.851016997 | -3.92160303 | 9.36E-05 | 0.000502 | 0.68104037 |
| CXCL11 | 0.337918637 | 2.662020098 | 3.921338169 | 9.37E-05 | 0.000503 | 0.680026307 |
| LPAR5 | -0.137851254 | 2.297923721 | -3.92091648 | 9.39E-05 | 0.000503 | 0.678411925 |
| ITGB3 | 0.191001812 | 1.993442167 | 3.920816852 | 9.39E-05 | 0.000503 | 0.678030554 |
| ZFHX4 | -0.139969793 | 2.241321624 | -3.91737357 | 9.52E-05 | 0.00051 | 0.664855155 |
| ZMPSTE24 | 0.126306784 | 4.123432215 | 3.912869958 | 9.70E-05 | 0.000519 | 0.647639431 |
| TM6SF1 | -0.126266966 | 1.981439788 | -3.91230625 | 9.72E-05 | 0.00052 | 0.645485912 |
| ETV7 | 0.203066408 | 2.313952154 | 3.911730929 | 9.74E-05 | 0.000521 | 0.643288377 |
| LGALS1 | 0.192496568 | 5.618522389 | 3.910325362 | 9.80E-05 | 0.000524 | 0.637920848 |
| SCGB2A1 | 0.393945079 | 4.211986294 | 3.906925979 | 9.94E-05 | 0.000531 | 0.62494716 |
| UBL5 | 0.151685909 | 4.859819633 | 3.906346095 | 9.96E-05 | 0.000532 | 0.622735138 |
| RSAD2 | 0.222817915 | 3.382751798 | 3.906262871 | 9.96E-05 | 0.000532 | 0.622417699 |
| RBX1 | 0.107675803 | 3.868768167 | 3.906027967 | 9.97E-05 | 0.000532 | 0.621521742 |
| PARVG | -0.133875128 | 2.752065358 | -3.90060228 | 0.000102 | 0.000544 | 0.600842025 |
| GPNMB | 0.237228181 | 4.06942612 | 3.898676181 | 0.000103 | 0.000548 | 0.593507525 |
| ALKBH8 | -0.095906557 | 2.299221441 | -3.8985984 | 0.000103 | 0.000548 | 0.593211395 |
| PTPRH | 0.188705956 | 1.600063502 | 3.895742809 | 0.000104 | 0.000554 | 0.582344128 |
| AP1M2 | 0.163302858 | 4.423348284 | 3.895020266 | 0.000104 | 0.000555 | 0.57959563 |
| MSN | 0.144993196 | 4.88993096 | 3.892494453 | 0.000105 | 0.000561 | 0.569991533 |
| MRTO4 | 0.102646367 | 3.77219408 | 3.892097382 | 0.000106 | 0.000561 | 0.56848227 |
| ARHGAP28 | -0.112202196 | 2.091147147 | -3.89082073 | 0.000106 | 0.000564 | 0.563630748 |
| NGF | -0.17237669 | 1.505811957 | -3.89003725 | 0.000106 | 0.000566 | 0.560654136 |
| COL2A1 | -0.255367926 | 1.566854435 | -3.88777512 | 0.000107 | 0.00057 | 0.552063084 |
| PFDN2 | 0.103276745 | 4.658396829 | 3.887679012 | 0.000107 | 0.00057 | 0.551698178 |
| HDAC5 | -0.128071892 | 3.457137649 | -3.88748002 | 0.000108 | 0.000571 | 0.550942694 |
| SNRPB | 0.138725277 | 5.493853374 | 3.887216588 | 0.000108 | 0.000571 | 0.549942633 |
| MRRF | 0.105365834 | 2.952120686 | 3.886785658 | 0.000108 | 0.000572 | 0.54830684 |
| GNG4 | -0.208122507 | 1.650400357 | -3.8859327 | 0.000108 | 0.000573 | 0.545069569 |
| ZNF286A | -0.107490354 | 2.242191965 | -3.88512349 | 0.000109 | 0.000575 | 0.541998949 |
| ZFP37 | -0.10538661 | 1.799461543 | -3.88499724 | 0.000109 | 0.000575 | 0.54151994 |
| CILP2 | -0.195465994 | 2.839666868 | -3.88380404 | 0.000109 | 0.000578 | 0.536993526 |
| CLK1 | -0.119106454 | 3.946491807 | -3.88275472 | 0.00011 | 0.00058 | 0.533014059 |
| ZNF423 | -0.199755535 | 2.610174435 | -3.88181609 | 0.00011 | 0.000582 | 0.529455243 |
| CCL19 | -0.24175792 | 1.698302582 | -3.88144498 | 0.00011 | 0.000582 | 0.528048437 |
| MYOCD | -0.154186611 | 1.244270741 | -3.88131291 | 0.00011 | 0.000582 | 0.527547806 |
| PRDM4 | -0.094195961 | 3.423813653 | -3.88078644 | 0.000111 | 0.000583 | 0.525552313 |
| NR4A3 | -0.195550979 | 2.167588736 | -3.87994569 | 0.000111 | 0.000585 | 0.522366125 |
| ARNT | -0.097606852 | 3.594211506 | -3.87952351 | 0.000111 | 0.000586 | 0.520766472 |
| NCOA5 | -0.0904149 | 3.954264087 | -3.87680559 | 0.000112 | 0.000592 | 0.510472138 |
| TRAPPC3 | 0.103261844 | 4.129127682 | 3.876093246 | 0.000113 | 0.000593 | 0.507775224 |
| BNIP3L | 0.127003932 | 4.137724489 | 3.87571895 | 0.000113 | 0.000594 | 0.506358351 |
| LMO4 | 0.127079301 | 4.214932497 | 3.875399548 | 0.000113 | 0.000594 | 0.505149382 |
| NEGR1 | -0.1184638 | 1.529898799 | -3.87538005 | 0.000113 | 0.000594 | 0.505075583 |
| RHBDL3 | -0.16759009 | 1.373090985 | -3.87449976 | 0.000113 | 0.000596 | 0.501744116 |
| GLE1 | 0.099473898 | 3.49682648 | 3.873863933 | 0.000114 | 0.000597 | 0.499338261 |
| POU2F2 | -0.09923574 | 2.261467283 | -3.87333037 | 0.000114 | 0.000598 | 0.497319669 |
| TRAF1 | -0.102035913 | 2.503106575 | -3.87290516 | 0.000114 | 0.000599 | 0.495711165 |
| ADAM28 | -0.173330621 | 2.697395173 | -3.8722997 | 0.000114 | 0.0006 | 0.493421146 |
| LLGL1 | -0.107904388 | 3.211766807 | -3.87226979 | 0.000114 | 0.0006 | 0.493308012 |
| BCL9 | -0.126756875 | 3.894318663 | -3.87110108 | 0.000115 | 0.000603 | 0.488888627 |
| TRAPPC9 | 0.119156024 | 3.830942073 | 3.870434664 | 0.000115 | 0.000604 | 0.486369209 |
| OXSR1 | 0.102956355 | 3.69900262 | 3.869674022 | 0.000116 | 0.000605 | 0.48349407 |
| RIOK3 | 0.118792713 | 3.724724699 | 3.868794063 | 0.000116 | 0.000607 | 0.480168612 |
| HNRNPH3 | -0.086089246 | 4.398819806 | -3.86858289 | 0.000116 | 0.000608 | 0.479370685 |
| MTX2 | 0.109269593 | 4.15512649 | 3.866936568 | 0.000117 | 0.000611 | 0.47315136 |
| COL8A2 | -0.218327187 | 3.084811367 | -3.86665921 | 0.000117 | 0.000612 | 0.472103824 |
| MAGEH1 | -0.161887319 | 3.302735014 | -3.86591991 | 0.000117 | 0.000613 | 0.469312009 |
| GPR161 | -0.131540285 | 2.54034052 | -3.86484265 | 0.000118 | 0.000616 | 0.465244864 |
| RNF175 | -0.147096401 | 1.229305696 | -3.86452898 | 0.000118 | 0.000616 | 0.464060822 |
| AKNA | -0.117348871 | 3.042006755 | -3.8627026 | 0.000119 | 0.00062 | 0.457168512 |
| SLC2A4 | -0.161736287 | 1.518688203 | -3.86091403 | 0.00012 | 0.000625 | 0.450421914 |
| SH2D3C | -0.101357021 | 2.295086136 | -3.86075676 | 0.00012 | 0.000625 | 0.449828832 |
| SLC29A3 | -0.129690586 | 3.310824601 | -3.86011202 | 0.00012 | 0.000626 | 0.44739769 |
| MAP4K4 | -0.125436966 | 3.675522693 | -3.85962052 | 0.00012 | 0.000627 | 0.445544627 |
| DET1 | -0.120015459 | 2.615276499 | -3.85848741 | 0.000121 | 0.00063 | 0.44127342 |
| PELI2 | 0.170026649 | 3.076334243 | 3.858007299 | 0.000121 | 0.00063 | 0.439464046 |
| PLA2R1 | -0.107988706 | 2.292719348 | -3.85658842 | 0.000122 | 0.000634 | 0.434118048 |
| GALE | 0.145511817 | 3.383559594 | 3.856483414 | 0.000122 | 0.000634 | 0.433722472 |
| ZDHHC12 | 0.125692232 | 3.810918311 | 3.855119827 | 0.000123 | 0.000637 | 0.428586726 |
| RFC5 | 0.116827704 | 3.375037413 | 3.853657977 | 0.000123 | 0.00064 | 0.423082848 |
| GDAP2 | 0.077780819 | 2.870829517 | 3.852206883 | 0.000124 | 0.000644 | 0.417621472 |
| DSTYK | -0.094172857 | 3.017738743 | -3.85195885 | 0.000124 | 0.000644 | 0.416688186 |
| SPIB | -0.152312385 | 1.371184104 | -3.84935941 | 0.000125 | 0.000651 | 0.406910445 |
| FKBP2 | 0.128112154 | 4.64539376 | 3.848142014 | 0.000126 | 0.000654 | 0.402333474 |
| SEPHS2 | 0.117423923 | 4.140022133 | 3.847010242 | 0.000127 | 0.000656 | 0.398079666 |
| TRA2A | -0.103681901 | 4.029466463 | -3.84633692 | 0.000127 | 0.000658 | 0.395549549 |
| P4HB | 0.134243808 | 5.530690325 | 3.843525366 | 0.000128 | 0.000665 | 0.384989262 |
| TSR2 | 0.109412034 | 3.949997013 | 3.843502942 | 0.000128 | 0.000665 | 0.384905067 |
| IGFBP7 | -0.164964855 | 5.543232123 | -3.8433913 | 0.000129 | 0.000665 | 0.384485906 |
| ALG13 | 0.100819182 | 3.145183109 | 3.842615395 | 0.000129 | 0.000666 | 0.381572997 |
| PDIA2 | -0.22379865 | 1.770674813 | -3.84163654 | 0.000129 | 0.000669 | 0.377898986 |
| RGS18 | -0.156127641 | 1.552136935 | -3.84112716 | 0.00013 | 0.00067 | 0.375987459 |
| MARCO | 0.233382129 | 2.110898325 | 3.841056937 | 0.00013 | 0.00067 | 0.375723966 |
| IRF1 | 0.169705028 | 3.857295337 | 3.840903902 | 0.00013 | 0.00067 | 0.375149736 |
| SCG5 | -0.257216262 | 2.915788372 | -3.83796639 | 0.000131 | 0.000677 | 0.364131685 |
| IQGAP3 | 0.141078587 | 3.115436164 | 3.837860996 | 0.000131 | 0.000677 | 0.363736538 |
| CRTAC1 | -0.332230025 | 2.976638509 | -3.83732185 | 0.000132 | 0.000679 | 0.361715247 |
| NLGN3 | -0.134999908 | 1.843579445 | -3.83668167 | 0.000132 | 0.00068 | 0.359315525 |
| PTK2B | -0.115409253 | 2.958679483 | -3.83487938 | 0.000133 | 0.000685 | 0.352561748 |
| CAMK1G | -0.173113214 | 1.521452304 | -3.83430499 | 0.000133 | 0.000686 | 0.350409965 |
| ZNF830 | -0.09965047 | 3.02860688 | -3.83382943 | 0.000134 | 0.000687 | 0.348628658 |
| VPS41 | -0.093519337 | 3.479185132 | -3.83305803 | 0.000134 | 0.000689 | 0.345739699 |
| PAEP | 0.366664741 | 2.268482317 | 3.832786307 | 0.000134 | 0.000689 | 0.344722186 |
| SH2D3A | 0.141763102 | 2.981269147 | 3.830320018 | 0.000135 | 0.000696 | 0.33549008 |
| RTKN2 | 0.112271896 | 2.214116196 | 3.82912602 | 0.000136 | 0.000699 | 0.331022645 |
| PRKAG2 | 0.119124991 | 3.122323873 | 3.82855085 | 0.000136 | 0.0007 | 0.328871083 |
| RBP7 | -0.204198974 | 2.113574705 | -3.82816085 | 0.000137 | 0.000701 | 0.327412368 |
| ASAP1 | 0.130211757 | 3.763538917 | 3.825045446 | 0.000138 | 0.000709 | 0.315765076 |
| SRPK1 | 0.11159241 | 3.841038309 | 3.824688073 | 0.000139 | 0.00071 | 0.314429588 |
| GPR135 | -0.097408639 | 2.019878103 | -3.82424041 | 0.000139 | 0.000711 | 0.312756871 |
| SCN4B | -0.144593236 | 1.564342431 | -3.8241292 | 0.000139 | 0.000711 | 0.312341355 |
| CCNF | 0.122996687 | 2.854794865 | 3.823593317 | 0.000139 | 0.000712 | 0.310339276 |
| CAPRIN2 | -0.10679247 | 2.96629678 | -3.82321025 | 0.000139 | 0.000713 | 0.308908302 |
| ATRX | -0.082379642 | 3.389408756 | -3.82270501 | 0.00014 | 0.000714 | 0.307021138 |
| LY6H | -0.20929627 | 1.367946051 | -3.82249873 | 0.00014 | 0.000714 | 0.306250742 |
| SIRT1 | -0.091620241 | 3.222019243 | -3.8219574 | 0.00014 | 0.000715 | 0.30422915 |
| FST | -0.285507295 | 2.681914485 | -3.82164485 | 0.00014 | 0.000716 | 0.303062085 |
| B3GNT4 | 0.137965128 | 1.559094216 | 3.821534689 | 0.00014 | 0.000716 | 0.302650763 |
| UBE2A | 0.098556653 | 3.747844796 | 3.821441524 | 0.00014 | 0.000716 | 0.302302908 |
| SAE1 | 0.119556374 | 4.019199641 | 3.821202954 | 0.00014 | 0.000716 | 0.301412184 |
| GOLT1B | 0.126891741 | 3.766286181 | 3.820987125 | 0.000141 | 0.000716 | 0.300606409 |
| SMC5 | 0.1055683 | 3.397986989 | 3.820671231 | 0.000141 | 0.000717 | 0.299427138 |
| MMGT1 | 0.122904424 | 3.592353726 | 3.819381713 | 0.000142 | 0.00072 | 0.294614184 |
| CYP4B1 | 0.379162954 | 3.566709492 | 3.819217684 | 0.000142 | 0.000721 | 0.294002081 |
| RECK | -0.10789565 | 2.289402602 | -3.81894785 | 0.000142 | 0.000721 | 0.292995191 |
| CNTN1 | -0.19687525 | 2.186230145 | -3.81878933 | 0.000142 | 0.000721 | 0.292403742 |
| SLC25A45 | -0.08825211 | 2.016527302 | -3.81628453 | 0.000143 | 0.000728 | 0.283060826 |
| CISD2 | 0.109214552 | 3.404947202 | 3.816187683 | 0.000143 | 0.000728 | 0.282699706 |
| POPDC2 | -0.100663784 | 1.904840796 | -3.81455828 | 0.000144 | 0.000732 | 0.276625395 |
| TJAP1 | -0.098712608 | 3.490065413 | -3.8140087 | 0.000145 | 0.000734 | 0.274577159 |
| CHRNA5 | 0.176378176 | 1.734839483 | 3.813068568 | 0.000145 | 0.000736 | 0.271074031 |
| SOBP | -0.150254126 | 2.064647022 | -3.81277673 | 0.000145 | 0.000737 | 0.269986743 |
| TBX1 | -0.182018259 | 1.688104478 | -3.81255755 | 0.000145 | 0.000737 | 0.269170225 |
| AMHR2 | -0.151387657 | 1.186859943 | -3.81241908 | 0.000146 | 0.000737 | 0.268654392 |
| HSPA13 | 0.131969443 | 3.253754879 | 3.812146172 | 0.000146 | 0.000737 | 0.267637818 |
| RIN2 | -0.111196381 | 3.815616057 | -3.81214281 | 0.000146 | 0.000737 | 0.267625298 |
| DLGAP4 | -0.100417796 | 4.13237796 | -3.81196778 | 0.000146 | 0.000737 | 0.266973352 |
| APLNR | -0.155079077 | 2.254914384 | -3.81095441 | 0.000146 | 0.00074 | 0.263199308 |
| LATS1 | -0.08649212 | 2.922606742 | -3.81071938 | 0.000147 | 0.00074 | 0.262324167 |
| NUDT11 | -0.175339535 | 1.551610942 | -3.81011553 | 0.000147 | 0.000742 | 0.260075869 |
| FGL2 | -0.194708271 | 2.977434229 | -3.80941089 | 0.000147 | 0.000743 | 0.257452772 |
| LYL1 | -0.124889093 | 2.183639751 | -3.80867541 | 0.000148 | 0.000745 | 0.254715372 |
| SLC16A4 | -0.156174458 | 2.094371376 | -3.80703324 | 0.000149 | 0.00075 | 0.248605199 |
| KIF22 | 0.111245683 | 3.870158574 | 3.806536892 | 0.000149 | 0.000751 | 0.246758901 |
| DCN | -0.280261261 | 4.102536096 | -3.80621073 | 0.000149 | 0.000752 | 0.24554578 |
| PCMT1 | 0.104899905 | 3.947621671 | 3.80508376 | 0.00015 | 0.000755 | 0.241354917 |
| PDIA6 | 0.13142712 | 5.05418185 | 3.804989091 | 0.00015 | 0.000755 | 0.241002926 |
| MRPL16 | 0.094057192 | 3.850311819 | 3.79878511 | 0.000154 | 0.000773 | 0.217954373 |
| CEACAM1 | 0.197545183 | 2.32358127 | 3.798765317 | 0.000154 | 0.000773 | 0.217880896 |
| SCARA5 | -0.163548693 | 1.146319997 | -3.79769002 | 0.000154 | 0.000775 | 0.213889793 |
| CCDC3 | -0.255372474 | 3.443103768 | -3.79768567 | 0.000154 | 0.000775 | 0.213873629 |
| KCNIP1 | -0.221228649 | 1.550345364 | -3.79679949 | 0.000155 | 0.000778 | 0.210585265 |
| ALKBH5 | 0.100737802 | 4.158354485 | 3.796029061 | 0.000155 | 0.00078 | 0.20772705 |
| VENTX | -0.113306476 | 1.703318493 | -3.79572956 | 0.000156 | 0.00078 | 0.206616079 |
| SLIT2 | -0.158758706 | 2.763487648 | -3.79490399 | 0.000156 | 0.000783 | 0.20355413 |
| HGFAC | -0.183206053 | 1.804436934 | -3.79464041 | 0.000156 | 0.000783 | 0.202576685 |
| PODXL | -0.199721267 | 4.557653522 | -3.79047276 | 0.000159 | 0.000796 | 0.187130321 |
| ZNF334 | -0.162105419 | 2.603755657 | -3.78784144 | 0.00016 | 0.000804 | 0.17738651 |
| TMCO6 | -0.104652571 | 3.213635191 | -3.78771486 | 0.000161 | 0.000804 | 0.176917943 |
| CLK4 | -0.092313044 | 2.742512425 | -3.78767699 | 0.000161 | 0.000804 | 0.176777746 |
| DNAJA2 | 0.101904234 | 3.798265235 | 3.787165549 | 0.000161 | 0.000805 | 0.17488473 |
| GPR35 | -0.124131062 | 2.082438373 | -3.7869378 | 0.000161 | 0.000805 | 0.174041832 |
| CPA3 | -0.189891271 | 1.358833729 | -3.78693236 | 0.000161 | 0.000805 | 0.174021686 |
| SLC26A11 | -0.11584752 | 3.058060407 | -3.78570105 | 0.000162 | 0.000808 | 0.169465451 |
| ATP6V1C2 | 0.212039328 | 2.290415226 | 3.785615273 | 0.000162 | 0.000808 | 0.169148108 |
| XRCC3 | 0.115306374 | 2.796268234 | 3.783553832 | 0.000163 | 0.000815 | 0.161523515 |
| NUDT13 | -0.11780027 | 1.828066764 | -3.78310214 | 0.000164 | 0.000816 | 0.159853397 |
| NIPA2 | 0.110506396 | 3.658299931 | 3.781428806 | 0.000165 | 0.000821 | 0.153667976 |
| MST1R | 0.187476268 | 2.597146636 | 3.780076925 | 0.000165 | 0.000825 | 0.148672742 |
| COL11A2 | -0.183347434 | 2.120444779 | -3.7784563 | 0.000167 | 0.00083 | 0.142686772 |
| TTC31 | -0.084148873 | 3.374808663 | -3.77825594 | 0.000167 | 0.00083 | 0.141946913 |
| NFAT5 | -0.100733007 | 2.975887691 | -3.77763254 | 0.000167 | 0.000832 | 0.139645077 |
| PRAM1 | -0.116975966 | 1.906132059 | -3.77742044 | 0.000167 | 0.000832 | 0.138862039 |
| TOX2 | -0.171965509 | 2.136761676 | -3.77665866 | 0.000168 | 0.000834 | 0.136049919 |
| DR1 | 0.09108031 | 3.477974625 | 3.774733447 | 0.000169 | 0.00084 | 0.128945467 |
| TMEM155 | -0.152156278 | 1.03996017 | -3.77456575 | 0.000169 | 0.00084 | 0.128326781 |
| TOR1B | 0.102717179 | 3.255323832 | 3.773464568 | 0.00017 | 0.000844 | 0.124264956 |
| C1orf21 | -0.155615941 | 2.656280403 | -3.77282227 | 0.00017 | 0.000846 | 0.121896285 |
| MCM3AP | -0.105953996 | 3.518570969 | -3.77237804 | 0.000171 | 0.000847 | 0.120258307 |
| FSTL1 | -0.193012963 | 4.068520305 | -3.77088266 | 0.000172 | 0.000851 | 0.114745812 |
| ACOT4 | 0.172085857 | 2.026260947 | 3.769495494 | 0.000173 | 0.000856 | 0.109634134 |
| ID4 | -0.236948733 | 4.584445109 | -3.76910688 | 0.000173 | 0.000856 | 0.108202441 |
| C6orf15 | 0.281068379 | 1.583206878 | 3.768867251 | 0.000173 | 0.000857 | 0.107319674 |
| NMB | 0.186229566 | 2.928784305 | 3.768830964 | 0.000173 | 0.000857 | 0.107186006 |
| SERPINB1 | 0.158038901 | 4.021370557 | 3.768517216 | 0.000173 | 0.000857 | 0.106030304 |
| PBX2 | -0.12679603 | 4.344094406 | -3.76834331 | 0.000173 | 0.000858 | 0.105389768 |
| CIB1 | 0.134413583 | 4.526587096 | 3.768184411 | 0.000173 | 0.000858 | 0.104804506 |
| XRCC2 | 0.115824464 | 2.694546006 | 3.765654069 | 0.000175 | 0.000866 | 0.095488125 |
| MAP2K5 | -0.094775972 | 2.883564213 | -3.76424415 | 0.000176 | 0.00087 | 0.090299631 |
| RANBP2 | -0.082296485 | 3.624998402 | -3.76268393 | 0.000177 | 0.000875 | 0.084560253 |
| CD37 | -0.159475 | 2.863585877 | -3.760995 | 0.000178 | 0.000881 | 0.078349988 |
| TRIP6 | -0.118149423 | 4.514966787 | -3.75936895 | 0.00018 | 0.000886 | 0.072373518 |
| CARD8 | -0.081436115 | 2.791719761 | -3.75824773 | 0.00018 | 0.00089 | 0.068253977 |
| KRT23 | 0.308335683 | 3.534386887 | 3.758085449 | 0.000181 | 0.00089 | 0.067657841 |
| SLCO2A1 | -0.157855259 | 2.5555763 | -3.75719212 | 0.000181 | 0.000893 | 0.064376606 |
| TM4SF1 | 0.196392167 | 5.131583149 | 3.756649608 | 0.000182 | 0.000894 | 0.062384318 |
| CREG1 | -0.108618325 | 4.953210125 | -3.75457982 | 0.000183 | 0.000901 | 0.054785906 |
| MAN2B1 | -0.130287293 | 4.005502271 | -3.75368767 | 0.000184 | 0.000904 | 0.051511965 |
| DCTN3 | 0.105163717 | 3.943674951 | 3.752671204 | 0.000184 | 0.000907 | 0.047782773 |
| WDR17 | -0.13310988 | 1.377222919 | -3.75238165 | 0.000185 | 0.000908 | 0.046720653 |
| PRICKLE3 | 0.130991129 | 3.279698489 | 3.75146623 | 0.000185 | 0.00091 | 0.04336325 |
| PSKH1 | -0.103965316 | 3.234100235 | -3.74985896 | 0.000186 | 0.000916 | 0.037470357 |
| MAMDC2 | -0.163858125 | 1.846464714 | -3.74920268 | 0.000187 | 0.000918 | 0.035064891 |
| KAT2A | -0.117854907 | 3.987508316 | -3.74905754 | 0.000187 | 0.000918 | 0.034532943 |
| CAP2 | -0.139539614 | 3.017998802 | -3.74868041 | 0.000187 | 0.000919 | 0.033150883 |
| FGFBP1 | 0.23054194 | 1.630472324 | 3.748124642 | 0.000188 | 0.00092 | 0.031114419 |
| SLC47A2 | -0.174411327 | 1.386166745 | -3.74768602 | 0.000188 | 0.000921 | 0.029507428 |
| SEC31A | -0.084613332 | 4.569421427 | -3.74765591 | 0.000188 | 0.000921 | 0.0293971 |
| HMGA2 | -0.249286898 | 2.605431684 | -3.74728893 | 0.000188 | 0.000922 | 0.028052703 |
| MTPN | 0.121703407 | 4.331272629 | 3.744123145 | 0.000191 | 0.000933 | 0.016460632 |
| NBEA | -0.110142046 | 2.114399967 | -3.74396225 | 0.000191 | 0.000934 | 0.015871742 |
| TTC1 | 0.093595885 | 4.254740185 | 3.743779964 | 0.000191 | 0.000934 | 0.015204586 |
| NUB1 | 0.106784842 | 3.89097771 | 3.742343087 | 0.000192 | 0.000938 | 0.009946822 |
| HTR7 | -0.114956779 | 1.35988742 | -3.74230668 | 0.000192 | 0.000938 | 0.00981361 |
| REST | 0.113219068 | 3.878267981 | 3.741693422 | 0.000193 | 0.00094 | 0.007570237 |
| RAB2B | -0.107881111 | 3.358962498 | -3.73998875 | 0.000194 | 0.000946 | 0.001336186 |
| UBA6 | 0.089331687 | 3.041458247 | 3.739692279 | 0.000194 | 0.000947 | 0.000252244 |
| NUDT21 | 0.107785365 | 3.913234109 | 3.739592635 | 0.000194 | 0.000947 | -0.00011204 |
| SNRNP25 | 0.122623557 | 3.241580226 | 3.739026273 | 0.000195 | 0.000949 | -0.00218243 |
| TMSB4X | 0.142984851 | 6.882316069 | 3.738717591 | 0.000195 | 0.000949 | -0.00331072 |
| MYH11 | -0.195341007 | 2.276454286 | -3.73836639 | 0.000195 | 0.00095 | -0.00459432 |
| HIGD2A | 0.135401807 | 5.226750355 | 3.736516338 | 0.000197 | 0.000957 | -0.01135405 |
| LOXL1 | -0.198835747 | 3.90410324 | -3.7364149 | 0.000197 | 0.000957 | -0.01172461 |
| AIFM1 | 0.137085763 | 4.240483119 | 3.736283489 | 0.000197 | 0.000957 | -0.0122046 |
| ST6GALNAC3 | -0.13595746 | 1.631514525 | -3.73521482 | 0.000198 | 0.00096 | -0.0161076 |
| PROS1 | -0.177123895 | 2.95558568 | -3.73516482 | 0.000198 | 0.00096 | -0.01629017 |
| ATP9B | -0.078355735 | 2.725325379 | -3.73497039 | 0.000198 | 0.00096 | -0.01700015 |
| GSPT1 | 0.093880866 | 4.085142079 | 3.734832307 | 0.000198 | 0.00096 | -0.01750435 |
| RNF114 | 0.086807995 | 4.253714152 | 3.734608608 | 0.000198 | 0.000961 | -0.01832112 |
| HECA | -0.096246044 | 3.131739659 | -3.73450876 | 0.000198 | 0.000961 | -0.01868569 |
| MMRN2 | -0.166893115 | 2.814330452 | -3.73427673 | 0.000198 | 0.000961 | -0.01953278 |
| POLE2 | 0.13656477 | 2.521799985 | 3.73409622 | 0.000198 | 0.000962 | -0.02019178 |
| SGSM2 | -0.117033257 | 3.301116205 | -3.73347334 | 0.000199 | 0.000963 | -0.02246552 |
| CTTN | 0.102867751 | 4.482606028 | 3.732502631 | 0.0002 | 0.000967 | -0.02600817 |
| ITGB3BP | 0.10264444 | 3.017280475 | 3.732194151 | 0.0002 | 0.000967 | -0.02713381 |
| UBL3 | -0.109650738 | 3.524130213 | -3.73141329 | 0.0002 | 0.00097 | -0.02998272 |
| PRDM16 | -0.151495477 | 1.459403057 | -3.73094683 | 0.000201 | 0.000971 | -0.03168431 |
| CIAPIN1 | 0.091795512 | 3.561167605 | 3.730633469 | 0.000201 | 0.000972 | -0.03282729 |
| GPRC5A | 0.250765915 | 3.226475951 | 3.729484022 | 0.000202 | 0.000976 | -0.03701907 |
| DLAT | 0.11324267 | 3.61154759 | 3.728763002 | 0.000203 | 0.000978 | -0.03964783 |
| DHFR | 0.124288216 | 3.16025473 | 3.726851381 | 0.000204 | 0.000985 | -0.046615 |
| TUBB6 | -0.156664767 | 3.762344762 | -3.72618838 | 0.000205 | 0.000987 | -0.04903059 |
| RAD51AP1 | 0.162447828 | 3.265113436 | 3.725353972 | 0.000205 | 0.00099 | -0.05207008 |
| NXF1 | -0.083032236 | 3.990049704 | -3.72479361 | 0.000206 | 0.000992 | -0.05411093 |
| ZNF587 | 0.104312356 | 3.097490618 | 3.724468094 | 0.000206 | 0.000993 | -0.05529634 |
| GPR180 | 0.098638633 | 2.585130927 | 3.724153504 | 0.000206 | 0.000994 | -0.05644186 |
| HAT1 | 0.097343383 | 3.591651171 | 3.722968434 | 0.000207 | 0.000998 | -0.06075622 |
| YPEL3 | -0.115550771 | 4.36448527 | -3.72216157 | 0.000208 | 0.001001 | -0.06369293 |
| HACE1 | -0.101532565 | 2.565481943 | -3.72120317 | 0.000209 | 0.001004 | -0.06718034 |
| NRAS | 0.120772204 | 4.070345126 | 3.717878632 | 0.000211 | 0.001017 | -0.07927092 |
| PSMC6 | 0.084000646 | 3.734846688 | 3.716470502 | 0.000213 | 0.001022 | -0.08438879 |
| MBD5 | -0.08252424 | 2.752144184 | -3.71541727 | 0.000213 | 0.001025 | -0.08821554 |
| SPHK2 | -0.111868105 | 3.003416444 | -3.71502812 | 0.000214 | 0.001027 | -0.0896292 |
| RNF216 | -0.09109998 | 3.463693919 | -3.71489861 | 0.000214 | 0.001027 | -0.09009962 |
| NDN | -0.222594249 | 2.880683104 | -3.71233692 | 0.000216 | 0.001037 | -0.09940145 |
| FBXO31 | -0.084279994 | 2.868072696 | -3.71198851 | 0.000216 | 0.001037 | -0.10066608 |
| ZNF10 | -0.104622446 | 2.470915787 | -3.7118619 | 0.000216 | 0.001038 | -0.10112559 |
| UFSP2 | -0.09291586 | 3.089240668 | -3.70910375 | 0.000219 | 0.001048 | -0.11113256 |
| CLIC4 | 0.135774662 | 4.526105152 | 3.708830151 | 0.000219 | 0.001049 | -0.11212482 |
| ACSS1 | -0.139783102 | 2.95396058 | -3.70857767 | 0.000219 | 0.00105 | -0.11304044 |
| GIMAP6 | -0.144542463 | 2.458241759 | -3.70837812 | 0.000219 | 0.00105 | -0.11376405 |
| ATXN2 | -0.085163411 | 3.506472341 | -3.70788883 | 0.00022 | 0.001051 | -0.11553817 |
| ARMC4 | -0.17119271 | 1.580438055 | -3.70729874 | 0.00022 | 0.001053 | -0.11767745 |
| ARHGEF15 | -0.094804865 | 2.069616319 | -3.70728976 | 0.00022 | 0.001053 | -0.11771002 |
| NAPA | 0.100977238 | 3.916676832 | 3.7049295 | 0.000222 | 0.001062 | -0.12626353 |
| GNAI3 | 0.082531283 | 3.383617351 | 3.703785079 | 0.000223 | 0.001067 | -0.13040897 |
| SPINK1 | 0.201821868 | 1.008867578 | 3.703541449 | 0.000224 | 0.001067 | -0.13129131 |
| NFIX | -0.16505377 | 4.490186866 | -3.70328219 | 0.000224 | 0.001068 | -0.1322302 |
| ZNF232 | -0.118254788 | 2.714711682 | -3.7008564 | 0.000226 | 0.001077 | -0.14101184 |
| GGCT | 0.119073748 | 4.187420112 | 3.700522662 | 0.000226 | 0.001078 | -0.14221959 |
| ATP10B | 0.17665999 | 1.939063665 | 3.700425514 | 0.000226 | 0.001078 | -0.14257113 |
| PTMA | 0.092083976 | 6.681407324 | 3.699996306 | 0.000227 | 0.001079 | -0.14412415 |
| CD79B | -0.176283353 | 1.565793869 | -3.69992692 | 0.000227 | 0.001079 | -0.14437518 |
| PARP16 | -0.106481273 | 2.797442688 | -3.69991376 | 0.000227 | 0.001079 | -0.14442281 |
| GNB2 | 0.104892044 | 5.041321453 | 3.698960109 | 0.000228 | 0.001083 | -0.14787276 |
| OXNAD1 | 0.081680662 | 2.837036932 | 3.69882883 | 0.000228 | 0.001083 | -0.14834761 |
| ADAMTS3 | -0.151642149 | 1.759937036 | -3.69773504 | 0.000229 | 0.001087 | -0.15230332 |
| ZNF140 | -0.099843163 | 2.97574931 | -3.69740594 | 0.000229 | 0.001088 | -0.15349328 |
| ITGB5 | -0.147574931 | 4.456716094 | -3.69714106 | 0.000229 | 0.001088 | -0.15445098 |
| SDF2 | -0.093793053 | 3.569262056 | -3.69654934 | 0.00023 | 0.00109 | -0.15659013 |
| RBP5 | -0.169157517 | 2.04695353 | -3.69550215 | 0.000231 | 0.001094 | -0.16037505 |
| PTK7 | -0.128466672 | 4.185177458 | -3.69523633 | 0.000231 | 0.001095 | -0.16133567 |
| ERRFI1 | 0.181248054 | 3.768104213 | 3.694752524 | 0.000231 | 0.001097 | -0.16308383 |
| SLC22A3 | -0.152799651 | 1.426936943 | -3.69465767 | 0.000231 | 0.001097 | -0.16342656 |
| NPM2 | -0.166277793 | 1.602924916 | -3.69344262 | 0.000232 | 0.001101 | -0.1678159 |
| NIT1 | -0.079130814 | 3.391798799 | -3.6932928 | 0.000233 | 0.001101 | -0.16835702 |
| PRMT7 | -0.085787949 | 2.959239107 | -3.69321247 | 0.000233 | 0.001101 | -0.16864717 |
| GTF2IRD2 | -0.095211505 | 2.295067862 | -3.69318537 | 0.000233 | 0.001101 | -0.16874502 |
| IRX5 | 0.279720075 | 1.803052867 | 3.692333517 | 0.000233 | 0.001104 | -0.17182129 |
| AMPD2 | -0.107694265 | 3.583833806 | -3.69114284 | 0.000235 | 0.001109 | -0.17611995 |
| FUNDC1 | 0.120362341 | 3.336017316 | 3.690465605 | 0.000235 | 0.001111 | -0.17856437 |
| JAM2 | -0.147933499 | 2.110704228 | -3.6901952 | 0.000235 | 0.001112 | -0.17954025 |
| EFR3A | 0.129389541 | 4.101498155 | 3.689691285 | 0.000236 | 0.001114 | -0.18135866 |
| PLAC8 | -0.156095182 | 1.789295578 | -3.6892169 | 0.000236 | 0.001115 | -0.18307027 |
| CPAMD8 | -0.240012921 | 2.747131418 | -3.6890281 | 0.000237 | 0.001115 | -0.18375142 |
| FXYD5 | -0.177412248 | 4.047410208 | -3.68893241 | 0.000237 | 0.001115 | -0.18409664 |
| DNAJC21 | 0.086506879 | 3.355628317 | 3.688242207 | 0.000237 | 0.001118 | -0.1865864 |
| TTC9C | 0.081011481 | 3.400712386 | 3.687858796 | 0.000238 | 0.001119 | -0.18796928 |
| RNF130 | -0.086117933 | 3.50423828 | -3.68739255 | 0.000238 | 0.001121 | -0.18965075 |
| PLCG1 | -0.125375394 | 3.576612677 | -3.68663652 | 0.000239 | 0.001124 | -0.19237681 |
| ERC1 | -0.121554489 | 3.482429709 | -3.68515235 | 0.00024 | 0.00113 | -0.1977268 |
| FOXO1 | -0.131332238 | 3.278549806 | -3.68504547 | 0.00024 | 0.00113 | -0.19811201 |
| IGF2R | -0.118344354 | 3.576238708 | -3.68408514 | 0.000241 | 0.001133 | -0.20157249 |
| GPATCH3 | -0.094084077 | 2.927127944 | -3.68384499 | 0.000241 | 0.001134 | -0.20243772 |
| TIMP3 | -0.228020676 | 3.466620267 | -3.68377943 | 0.000241 | 0.001134 | -0.20267391 |
| GRTP1 | 0.163411017 | 2.640087363 | 3.682986186 | 0.000242 | 0.001137 | -0.20553144 |
| VPS28 | 0.126315611 | 4.762203316 | 3.68184339 | 0.000243 | 0.001141 | -0.20964709 |
| FLII | -0.1097594 | 4.191912728 | -3.68140544 | 0.000244 | 0.001143 | -0.21122399 |
| ADNP | -0.096427781 | 4.387541776 | -3.6811714 | 0.000244 | 0.001143 | -0.2120666 |
| CSDC2 | -0.179423553 | 2.106496564 | -3.67977738 | 0.000245 | 0.001149 | -0.21708445 |
| SLC27A1 | -0.134589964 | 3.425414832 | -3.67974463 | 0.000245 | 0.001149 | -0.21720233 |
| SEC14L2 | 0.12583046 | 2.364440337 | 3.679352271 | 0.000246 | 0.00115 | -0.21861428 |
| MCM7 | 0.113802565 | 4.529941452 | 3.678892554 | 0.000246 | 0.001151 | -0.22026846 |
| UBR5 | 0.109824887 | 4.042500712 | 3.678642925 | 0.000246 | 0.001152 | -0.22116661 |
| PARP11 | -0.119116185 | 2.488320527 | -3.67704771 | 0.000248 | 0.001159 | -0.22690464 |
| YPEL4 | -0.10663341 | 1.665028258 | -3.6768821 | 0.000248 | 0.001159 | -0.22750023 |
| C1QTNF2 | -0.135335954 | 1.303225462 | -3.6767379 | 0.000248 | 0.001159 | -0.22801876 |
| PBX1 | -0.115159837 | 4.126085838 | -3.67551549 | 0.000249 | 0.001164 | -0.23241384 |
| MAP3K12 | -0.128343219 | 3.120203745 | -3.67277884 | 0.000252 | 0.001176 | -0.24224803 |
| CUL1 | 0.091080282 | 4.0054038 | 3.672719815 | 0.000252 | 0.001176 | -0.24246004 |
| DYNLT3 | 0.117916888 | 3.345593841 | 3.67251189 | 0.000252 | 0.001176 | -0.24320692 |
| PHF20L1 | 0.0952424 | 3.360761053 | 3.671862507 | 0.000253 | 0.001179 | -0.24553928 |
| ZC3HAV1 | 0.095545153 | 3.638980314 | 3.671530976 | 0.000253 | 0.00118 | -0.24672986 |
| GPR3 | 0.138625428 | 1.605837879 | 3.669890561 | 0.000255 | 0.001187 | -0.25261934 |
| SNAP29 | 0.102935035 | 3.315723463 | 3.669264375 | 0.000255 | 0.001189 | -0.25486682 |
| FCRLA | -0.1338834 | 1.071035978 | -3.66923283 | 0.000255 | 0.001189 | -0.25498002 |
| PSENEN | 0.141562405 | 4.281524833 | 3.669051431 | 0.000256 | 0.001189 | -0.25563103 |
| GOLGB1 | -0.086289188 | 4.080573829 | -3.66671965 | 0.000258 | 0.001199 | -0.26399638 |
| CCL22 | -0.142318263 | 1.484568867 | -3.66596619 | 0.000259 | 0.001202 | -0.26669834 |
| RILPL2 | -0.106481192 | 2.703862501 | -3.66518506 | 0.000259 | 0.001205 | -0.26949897 |
| ENG | -0.11622359 | 3.932132868 | -3.66509136 | 0.000259 | 0.001205 | -0.26983488 |
| TCTN3 | -0.085238526 | 3.863513735 | -3.66460648 | 0.00026 | 0.001207 | -0.271573 |
| ATP6V1G1 | 0.105334854 | 4.902567062 | 3.664316776 | 0.00026 | 0.001208 | -0.27261138 |
| ASB13 | -0.116061053 | 3.280739429 | -3.66393832 | 0.000261 | 0.001209 | -0.27396776 |
| RBAK | -0.097644307 | 2.74021684 | -3.6633378 | 0.000261 | 0.001211 | -0.27611971 |
| FUT11 | 0.095197361 | 2.925799333 | 3.662960579 | 0.000262 | 0.001213 | -0.27747131 |
| NCOR2 | -0.124652073 | 4.431175989 | -3.66286796 | 0.000262 | 0.001213 | -0.27780314 |
| TSPAN17 | 0.097017815 | 3.603319326 | 3.662449126 | 0.000262 | 0.001214 | -0.27930364 |
| FAM3A | 0.108009274 | 3.634518684 | 3.661532353 | 0.000263 | 0.001218 | -0.28258745 |
| SREBF2 | 0.121944367 | 3.860947411 | 3.661291266 | 0.000263 | 0.001218 | -0.28345087 |
| API5 | 0.088727961 | 4.08937492 | 3.660909537 | 0.000264 | 0.00122 | -0.28481787 |
| CD28 | -0.129718893 | 1.708479477 | -3.66071479 | 0.000264 | 0.00122 | -0.28551522 |
| TRIM24 | 0.096466557 | 3.204760888 | 3.660477189 | 0.000264 | 0.001221 | -0.28636598 |
| TRNT1 | 0.08688627 | 3.06340165 | 3.660401248 | 0.000264 | 0.001221 | -0.28663788 |
| SOX18 | 0.205960078 | 3.392582746 | 3.659155413 | 0.000265 | 0.001226 | -0.29109774 |
| MRPL50 | 0.105029942 | 3.066099379 | 3.6587635 | 0.000266 | 0.001227 | -0.2925004 |
| PPARGC1B | 0.126785413 | 2.835872015 | 3.658670493 | 0.000266 | 0.001227 | -0.29283326 |
| SPCS1 | 0.097933064 | 4.230665903 | 3.658619225 | 0.000266 | 0.001227 | -0.29301673 |
| ECEL1 | -0.259651789 | 1.86298947 | -3.65827236 | 0.000266 | 0.001228 | -0.29425799 |
| CNFN | 0.222438119 | 2.685437256 | 3.658017184 | 0.000267 | 0.001229 | -0.29517109 |
| AEBP1 | -0.267535456 | 4.854823387 | -3.65704485 | 0.000268 | 0.001233 | -0.29864976 |
| FADS3 | 0.107725096 | 3.369791041 | 3.656372402 | 0.000268 | 0.001236 | -0.30105505 |
| POU2F3 | 0.209614349 | 2.379862197 | 3.655655813 | 0.000269 | 0.001238 | -0.30361773 |
| RPP14 | 0.096862243 | 2.980101905 | 3.654285799 | 0.00027 | 0.001245 | -0.30851585 |
| PARP2 | 0.110627572 | 3.396919661 | 3.653830739 | 0.000271 | 0.001246 | -0.3101424 |
| TALDO1 | 0.109198862 | 4.675261711 | 3.653365241 | 0.000271 | 0.001248 | -0.31180605 |
| NEK4 | 0.090675857 | 2.922790403 | 3.653279881 | 0.000272 | 0.001248 | -0.31211109 |
| GLS | -0.111505144 | 3.271467548 | -3.65321085 | 0.000272 | 0.001248 | -0.31235778 |
| SAP30 | 0.127899791 | 2.926859701 | 3.652224522 | 0.000273 | 0.001252 | -0.31588201 |
| PROCR | -0.162080389 | 2.660126277 | -3.65199621 | 0.000273 | 0.001252 | -0.31669766 |
| TAGAP | -0.131561336 | 2.133571233 | -3.6518922 | 0.000273 | 0.001252 | -0.31706923 |
| PSMB9 | 0.221298244 | 3.84911866 | 3.651705689 | 0.000273 | 0.001253 | -0.31773547 |
| DNPEP | 0.094962523 | 3.929958292 | 3.651280011 | 0.000274 | 0.001254 | -0.31925596 |
| DPP10 | -0.186791101 | 1.430512927 | -3.65102238 | 0.000274 | 0.001255 | -0.3201761 |
| TATDN1 | 0.122841064 | 3.771690831 | 3.650030764 | 0.000275 | 0.001259 | -0.32371716 |
| C1QTNF3 | -0.189134263 | 2.742389211 | -3.6496799 | 0.000275 | 0.00126 | -0.32496988 |
| OLA1 | 0.111912894 | 4.171839269 | 3.648146235 | 0.000277 | 0.001267 | -0.33044421 |
| TTC19 | -0.077378145 | 3.528786045 | -3.64766294 | 0.000277 | 0.001269 | -0.33216883 |
| RFX1 | -0.107164815 | 3.340595937 | -3.6472018 | 0.000278 | 0.001271 | -0.33381422 |
| LRPPRC | 0.091557754 | 4.255277337 | 3.645680127 | 0.00028 | 0.001278 | -0.33924216 |
| DYNC1LI1 | 0.088250982 | 3.346581279 | 3.645332527 | 0.00028 | 0.001279 | -0.34048177 |
| PSMG2 | 0.094470163 | 3.900354138 | 3.644000958 | 0.000281 | 0.001285 | -0.34522935 |
| PPP2R3C | 0.092706376 | 2.916826387 | 3.641413999 | 0.000284 | 0.001297 | -0.35444806 |
| DDC | 0.186187611 | 1.141543848 | 3.640456498 | 0.000285 | 0.001302 | -0.35785852 |
| DUSP19 | -0.105848047 | 1.947885053 | -3.63930721 | 0.000287 | 0.001307 | -0.36195094 |
| ZFAND5 | 0.092716777 | 4.38530765 | 3.638396524 | 0.000288 | 0.001311 | -0.36519286 |
| FLT3 | -0.132025575 | 1.311149702 | -3.6370531 | 0.000289 | 0.001317 | -0.3699738 |
| CA14 | -0.172529884 | 1.457020726 | -3.63497426 | 0.000291 | 0.001327 | -0.37736856 |
| USP44 | -0.163388154 | 1.52004902 | -3.63453351 | 0.000292 | 0.001329 | -0.37893583 |
| RPL19 | -0.107923483 | 6.594990702 | -3.63375001 | 0.000293 | 0.001332 | -0.38172147 |
| STAT5B | -0.088592834 | 3.212532298 | -3.63244872 | 0.000294 | 0.001338 | -0.38634671 |
| LSR | 0.130847529 | 5.185328709 | 3.632238839 | 0.000294 | 0.001339 | -0.38709256 |
| ZNF117 | -0.129088548 | 3.101815853 | -3.63146389 | 0.000295 | 0.001342 | -0.38984609 |
| ZNF256 | 0.110643095 | 2.695715976 | 3.631443106 | 0.000295 | 0.001342 | -0.38991993 |
| RALA | 0.096014463 | 4.110156288 | 3.630917924 | 0.000296 | 0.001344 | -0.39178566 |
| MRPS27 | -0.100043811 | 3.336711349 | -3.62977466 | 0.000297 | 0.001349 | -0.39584623 |
| CETN2 | 0.133382919 | 4.490584884 | 3.629479037 | 0.000297 | 0.00135 | -0.39689601 |
| SAFB2 | -0.087035206 | 3.653349833 | -3.62831494 | 0.000299 | 0.001356 | -0.40102895 |
| VPS13C | -0.084151897 | 3.100048228 | -3.62806411 | 0.000299 | 0.001356 | -0.40191932 |
| TEAD3 | -0.117339091 | 4.036020955 | -3.6273492 | 0.0003 | 0.00136 | -0.40445669 |
| IRX2 | -0.148487286 | 1.089221477 | -3.627202 | 0.0003 | 0.00136 | -0.4049791 |
| SUB1 | 0.10347412 | 4.5140885 | 3.626381103 | 0.000301 | 0.001364 | -0.40789192 |
| RPL31 | -0.126634957 | 5.849996615 | -3.62395525 | 0.000304 | 0.001375 | -0.41649593 |
| TDO2 | 0.209519238 | 2.170448572 | 3.623863402 | 0.000304 | 0.001375 | -0.41682159 |
| EIF4E | 0.090759887 | 2.900405754 | 3.623848676 | 0.000304 | 0.001375 | -0.4168738 |
| TTC17 | -0.084325469 | 3.681115795 | -3.62299079 | 0.000305 | 0.001379 | -0.4199151 |
| PIBF1 | -0.095356092 | 3.028463836 | -3.62081564 | 0.000308 | 0.00139 | -0.42762316 |
| AGER | -0.136718961 | 2.705553782 | -3.62053715 | 0.000308 | 0.001391 | -0.42860973 |
| VASN | -0.145933278 | 4.298939942 | -3.62042635 | 0.000308 | 0.001391 | -0.4290022 |
| AHI1 | -0.089765227 | 2.69513006 | -3.62033547 | 0.000308 | 0.001391 | -0.42932411 |
| TMEM87B | 0.097130897 | 3.530706155 | 3.620163939 | 0.000308 | 0.001391 | -0.4299317 |
| SLC1A5 | 0.13552391 | 4.336250765 | 3.620078325 | 0.000308 | 0.001391 | -0.43023494 |
| MRPL33 | 0.112193247 | 4.588510066 | 3.617103397 | 0.000312 | 0.001406 | -0.44076774 |
| PTGER4 | -0.122507014 | 2.353905174 | -3.61589903 | 0.000313 | 0.001412 | -0.44502944 |
| FBN1 | -0.212194707 | 3.142076511 | -3.61463163 | 0.000315 | 0.001418 | -0.44951267 |
| BRAF | 0.081983802 | 3.132030792 | 3.614622373 | 0.000315 | 0.001418 | -0.4495454 |
| FAM110A | 0.130136148 | 2.952573609 | 3.614366928 | 0.000315 | 0.001419 | -0.45044881 |
| ALG8 | 0.123305143 | 4.011741072 | 3.614305668 | 0.000315 | 0.001419 | -0.45066545 |
| ATP1B3 | 0.125016486 | 4.268445174 | 3.613565779 | 0.000316 | 0.001422 | -0.45328176 |
| PI4KB | -0.100226407 | 4.194077485 | -3.61350386 | 0.000316 | 0.001422 | -0.45350068 |
| NCALD | 0.163071884 | 3.023710169 | 3.613068455 | 0.000317 | 0.001424 | -0.45504004 |
| IMPAD1 | 0.097625069 | 3.924816145 | 3.612766815 | 0.000317 | 0.001425 | -0.45610637 |
| HAX1 | 0.089920445 | 4.491227687 | 3.61171702 | 0.000318 | 0.00143 | -0.45981683 |
| ARL4C | -0.178264457 | 4.555134313 | -3.61099514 | 0.000319 | 0.001433 | -0.46236769 |
| PRRX2 | 0.228018123 | 3.253999699 | 3.610518311 | 0.00032 | 0.001435 | -0.46405233 |
| RNF135 | -0.088098484 | 3.301944263 | -3.6102394 | 0.00032 | 0.001436 | -0.46503763 |
| PDZD7 | -0.138752723 | 1.725758741 | -3.6086628 | 0.000322 | 0.001444 | -0.47060585 |
| UBXN1 | -0.09800093 | 4.285343643 | -3.60834659 | 0.000322 | 0.001445 | -0.47172237 |
| FBXW4 | -0.098375311 | 3.642810343 | -3.60747299 | 0.000324 | 0.00145 | -0.47480646 |
| SIRT3 | -0.087897274 | 2.9384416 | -3.60682488 | 0.000324 | 0.001453 | -0.47709404 |
| ZNF672 | 0.101356344 | 3.668193179 | 3.606559017 | 0.000325 | 0.001453 | -0.47803231 |
| KLHL32 | -0.138907325 | 1.482225995 | -3.60516679 | 0.000326 | 0.001461 | -0.48294459 |
| TCF7 | -0.129239509 | 2.734812409 | -3.60496702 | 0.000327 | 0.001461 | -0.48364931 |
| STAB1 | -0.156353888 | 3.480334303 | -3.60493365 | 0.000327 | 0.001461 | -0.48376703 |
| AKAP3 | -0.178281707 | 1.959995123 | -3.6033829 | 0.000329 | 0.001468 | -0.48923614 |
| COMMD8 | 0.13040944 | 3.255116049 | 3.603381842 | 0.000329 | 0.001468 | -0.48923986 |
| TFAP2A | 0.248183877 | 2.817484612 | 3.603166754 | 0.000329 | 0.001469 | -0.48999824 |
| DPP6 | -0.193430029 | 1.639133358 | -3.60303385 | 0.000329 | 0.001469 | -0.49046683 |
| ERLIN1 | -0.101570314 | 3.344449008 | -3.60291546 | 0.000329 | 0.001469 | -0.49088421 |
| MFHAS1 | 0.145049521 | 3.46102783 | 3.60270209 | 0.000329 | 0.00147 | -0.49163644 |
| PNPLA4 | 0.148253231 | 3.089416888 | 3.602185012 | 0.00033 | 0.001472 | -0.4934592 |
| CYB561D2 | 0.119389746 | 2.98708816 | 3.602087185 | 0.00033 | 0.001472 | -0.49380402 |
| ZNF345 | -0.099733609 | 2.470090264 | -3.60110028 | 0.000331 | 0.001477 | -0.49728216 |
| ZNF692 | -0.128689781 | 3.670099333 | -3.60076846 | 0.000332 | 0.001478 | -0.49845139 |
| DIDO1 | -0.092396048 | 3.750307644 | -3.59958908 | 0.000333 | 0.001484 | -0.50260628 |
| ANKRD23 | -0.10699204 | 1.700874341 | -3.59833898 | 0.000335 | 0.00149 | -0.50700887 |
| GNG11 | -0.154945096 | 2.630080364 | -3.59664143 | 0.000337 | 0.0015 | -0.51298488 |
| RNF208 | 0.157317884 | 3.43522408 | 3.595881267 | 0.000338 | 0.001503 | -0.51566006 |
| NXT2 | 0.144401328 | 3.15096909 | 3.595737471 | 0.000338 | 0.001503 | -0.51616604 |
| ESPL1 | 0.137060613 | 3.104082547 | 3.595726841 | 0.000338 | 0.001503 | -0.51620345 |
| MYO1F | -0.124477546 | 2.719342456 | -3.59505731 | 0.000339 | 0.001506 | -0.51855911 |
| KRT15 | 0.213924886 | 2.250947675 | 3.593811382 | 0.000341 | 0.001513 | -0.52294161 |
| PNRC1 | -0.106237971 | 4.980113286 | -3.59001915 | 0.000346 | 0.001534 | -0.53627151 |
| MRPS11 | 0.087990369 | 3.164775031 | 3.588236572 | 0.000348 | 0.001544 | -0.54253263 |
| RBM39 | -0.089567886 | 4.628024253 | -3.58745215 | 0.000349 | 0.001547 | -0.54528685 |
| KIAA0355 | -0.104366836 | 3.277245959 | -3.58743979 | 0.000349 | 0.001547 | -0.54533027 |
| GLYATL2 | 0.209152266 | 1.250874061 | 3.587286861 | 0.000349 | 0.001547 | -0.54586715 |
| DNAJC18 | -0.101415138 | 2.370938041 | -3.58621039 | 0.000351 | 0.001553 | -0.5496457 |
| PLCB1 | -0.130563518 | 2.710249223 | -3.58523423 | 0.000352 | 0.001558 | -0.55307118 |
| SERPINF1 | -0.2448417 | 4.117156733 | -3.58478039 | 0.000353 | 0.00156 | -0.55466346 |
| VKORC1 | 0.107859838 | 3.760798741 | 3.584334173 | 0.000353 | 0.001562 | -0.5562288 |
| DHRS1 | 0.102277811 | 3.286561054 | 3.584028609 | 0.000354 | 0.001563 | -0.55730062 |
| MAP3K6 | 0.132930142 | 3.073790252 | 3.583591429 | 0.000354 | 0.001565 | -0.55883394 |
| MMRN1 | -0.151791651 | 1.167883259 | -3.58336302 | 0.000355 | 0.001566 | -0.55963499 |
| IMPDH2 | -0.123658358 | 5.065877735 | -3.58131992 | 0.000357 | 0.001578 | -0.56679789 |
| MST1 | -0.150083911 | 2.840453407 | -3.58122987 | 0.000357 | 0.001578 | -0.56711353 |
| FER | -0.075101942 | 2.503955602 | -3.58054745 | 0.000358 | 0.001581 | -0.56950509 |
| SYNGAP1 | -0.127329733 | 3.283592153 | -3.57878263 | 0.000361 | 0.001591 | -0.57568786 |
| CTSH | -0.133508414 | 3.967413623 | -3.57839801 | 0.000361 | 0.001592 | -0.57703494 |
| SLC34A2 | 0.255746457 | 5.494132973 | 3.578329518 | 0.000361 | 0.001592 | -0.5772748 |
| TREML2 | -0.153219348 | 1.294136152 | -3.57756844 | 0.000362 | 0.001596 | -0.5799399 |
| GIMAP7 | -0.17040326 | 2.398149601 | -3.57723155 | 0.000363 | 0.001598 | -0.58111939 |
| CORO7 | -0.089268861 | 2.299367016 | -3.57705467 | 0.000363 | 0.001598 | -0.58173865 |
| HYAL3 | 0.157119666 | 2.463862139 | 3.576848402 | 0.000363 | 0.001599 | -0.58246074 |
| ARHGEF10 | -0.120956792 | 2.762451231 | -3.57652368 | 0.000364 | 0.0016 | -0.58359744 |
| PCBD1 | 0.125140242 | 4.81829552 | 3.576376173 | 0.000364 | 0.0016 | -0.58411375 |
| FOLR2 | -0.173269094 | 2.757396202 | -3.57585815 | 0.000365 | 0.001603 | -0.58592682 |
| NXPH3 | -0.126203702 | 1.677159705 | -3.57551613 | 0.000365 | 0.001604 | -0.58712374 |
| USP18 | 0.191995544 | 3.44215205 | 3.575096611 | 0.000366 | 0.001606 | -0.58859171 |
| STRA6 | -0.193066931 | 2.754150342 | -3.57383569 | 0.000368 | 0.001613 | -0.5930029 |
| KCNQ1 | -0.15200824 | 2.645581695 | -3.57219813 | 0.00037 | 0.001622 | -0.59872945 |
| DCK | 0.125002496 | 3.387917236 | 3.572181076 | 0.00037 | 0.001622 | -0.59878909 |
| ICAM2 | -0.139235171 | 2.166504917 | -3.57147405 | 0.000371 | 0.001626 | -0.60126076 |
| ZDHHC7 | -0.083324842 | 3.647282953 | -3.5702597 | 0.000373 | 0.001632 | -0.60550485 |
| SOCS5 | -0.088044516 | 3.343016405 | -3.5702021 | 0.000373 | 0.001632 | -0.60570611 |
| SF3A3 | 0.095812643 | 4.186685666 | 3.569525217 | 0.000374 | 0.001636 | -0.60807115 |
| TCTN1 | -0.115054953 | 3.093146222 | -3.56865229 | 0.000375 | 0.001641 | -0.61112049 |
| ALG2 | 0.089356813 | 3.611976931 | 3.568422501 | 0.000375 | 0.001641 | -0.6119231 |
| ABCB8 | 0.095338783 | 3.122418964 | 3.566870007 | 0.000377 | 0.00165 | -0.61734421 |
| SERINC2 | 0.178415235 | 3.970839233 | 3.566351352 | 0.000378 | 0.001653 | -0.61915478 |
| ARAP1 | -0.103143287 | 4.003477621 | -3.56481821 | 0.00038 | 0.001662 | -0.62450529 |
| PAN2 | -0.120456464 | 3.44636111 | -3.56443726 | 0.000381 | 0.001663 | -0.62583443 |
| BRMS1 | 0.096631845 | 3.772047236 | 3.564407827 | 0.000381 | 0.001663 | -0.62593711 |
| MRPL19 | 0.082166378 | 3.386882741 | 3.564376858 | 0.000381 | 0.001663 | -0.62604516 |
| PRKACB | -0.111798647 | 2.717160272 | -3.56387714 | 0.000382 | 0.001665 | -0.62778841 |
| PLEKHB2 | 0.09656586 | 3.983829094 | 3.563197925 | 0.000383 | 0.001669 | -0.6301575 |
| UBC | 0.083720762 | 5.940679106 | 3.563021671 | 0.000383 | 0.001669 | -0.63077219 |
| C1orf112 | 0.096356665 | 2.686553227 | 3.562950852 | 0.000383 | 0.001669 | -0.63101917 |
| CD44 | 0.170080942 | 3.460394083 | 3.562258861 | 0.000384 | 0.001673 | -0.63343218 |
| CHAF1A | 0.103459314 | 3.065239245 | 3.561612212 | 0.000385 | 0.001676 | -0.63568667 |
| CD200R1 | -0.115079491 | 1.620829607 | -3.56085225 | 0.000386 | 0.001679 | -0.6383357 |
| PPP2R2B | -0.205411985 | 1.982171684 | -3.5608059 | 0.000386 | 0.001679 | -0.63849723 |
| PDE1A | -0.167876648 | 2.098177547 | -3.56079272 | 0.000386 | 0.001679 | -0.63854318 |
| FAM172A | -0.093276475 | 3.118302778 | -3.56048395 | 0.000387 | 0.001681 | -0.6396193 |
| CCDC88A | -0.103246548 | 2.693232335 | -3.56008601 | 0.000387 | 0.001682 | -0.64100607 |
| ATG4D | 0.1347697 | 3.390229291 | 3.559217112 | 0.000388 | 0.001687 | -0.64403351 |
| TTBK2 | -0.069013066 | 2.229716227 | -3.55869378 | 0.000389 | 0.00169 | -0.64585658 |
| ACSM3 | -0.160507491 | 2.580825475 | -3.55773811 | 0.000391 | 0.001695 | -0.64918504 |
| TSFM | 0.094237263 | 3.363905927 | 3.556573092 | 0.000392 | 0.001701 | -0.65324146 |
| METRN | 0.181152279 | 3.436286306 | 3.556562996 | 0.000392 | 0.001701 | -0.65327661 |
| ZAP70 | -0.125382983 | 1.94126121 | -3.55650512 | 0.000392 | 0.001701 | -0.65347809 |
| DEK | 0.138681707 | 4.881558844 | 3.555308189 | 0.000394 | 0.001708 | -0.6576442 |
| TMEM187 | 0.141870537 | 2.932244949 | 3.554602288 | 0.000395 | 0.001712 | -0.66010056 |
| POLR2H | 0.095375212 | 4.464674257 | 3.554576249 | 0.000395 | 0.001712 | -0.66019115 |
| MRPS35 | 0.119003692 | 4.377870697 | 3.551365812 | 0.0004 | 0.001732 | -0.67135658 |
| YKT6 | 0.095413681 | 4.105536296 | 3.549032105 | 0.000404 | 0.001746 | -0.67946669 |
| CEP164 | -0.10026304 | 3.315934865 | -3.5481485 | 0.000405 | 0.001751 | -0.68253602 |
| PTDSS1 | 0.105302874 | 4.15707003 | 3.547875172 | 0.000405 | 0.001752 | -0.68348533 |
| RER1 | 0.087418341 | 4.245107126 | 3.54776628 | 0.000405 | 0.001753 | -0.68386351 |
| SEMA3C | -0.173579731 | 3.510164355 | -3.54740243 | 0.000406 | 0.001754 | -0.68512706 |
| BTN2A1 | -0.090635461 | 3.386138828 | -3.54726101 | 0.000406 | 0.001754 | -0.68561813 |
| WDR1 | 0.100561518 | 4.663016921 | 3.546932143 | 0.000407 | 0.001756 | -0.68676003 |
| PCCB | 0.104558259 | 3.463747415 | 3.545801659 | 0.000408 | 0.001763 | -0.69068455 |
| DUSP10 | 0.166503318 | 3.030382492 | 3.543780416 | 0.000412 | 0.001775 | -0.69769833 |
| OVOL2 | 0.124480654 | 3.34835042 | 3.54333604 | 0.000412 | 0.001778 | -0.69923981 |
| ZNF624 | -0.078219389 | 1.945898075 | -3.54303101 | 0.000413 | 0.001779 | -0.70029782 |
| BMPR1B | -0.149499521 | 2.947373845 | -3.54280589 | 0.000413 | 0.00178 | -0.70107858 |
| EFEMP2 | -0.151017638 | 4.044305186 | -3.54222528 | 0.000414 | 0.001783 | -0.70309206 |
| RPL6 | -0.112928001 | 6.081937357 | -3.54157076 | 0.000415 | 0.001787 | -0.70536145 |
| MNAT1 | 0.088088718 | 3.187305699 | 3.541013185 | 0.000416 | 0.00179 | -0.70729439 |
| TMEM11 | 0.080954742 | 3.257898901 | 3.540506914 | 0.000417 | 0.001792 | -0.70904922 |
| FOXK2 | 0.089888796 | 3.634458805 | 3.540450313 | 0.000417 | 0.001792 | -0.70924539 |
| RC3H2 | 0.090979482 | 3.312172556 | 3.540219892 | 0.000417 | 0.001793 | -0.71004398 |
| RIT1 | 0.091601 | 3.294141418 | 3.538775698 | 0.000419 | 0.001802 | -0.71504807 |
| ALDH2 | -0.153183187 | 3.489976235 | -3.53756577 | 0.000421 | 0.001809 | -0.71923892 |
| EFTUD2 | 0.086111717 | 3.751510942 | 3.537162884 | 0.000422 | 0.001811 | -0.72063408 |
| DDX58 | 0.172426815 | 3.813320304 | 3.537086969 | 0.000422 | 0.001811 | -0.72089695 |
| SGSH | -0.109225457 | 3.351159579 | -3.53707378 | 0.000422 | 0.001811 | -0.72094262 |
| ZDHHC5 | 0.079252455 | 4.249108712 | 3.53680414 | 0.000422 | 0.001812 | -0.72187625 |
| HSPD1 | 0.11216059 | 5.068687446 | 3.536214004 | 0.000423 | 0.001815 | -0.72391937 |
| STX5 | 0.066429353 | 4.134680819 | 3.53614848 | 0.000423 | 0.001815 | -0.7241462 |
| SLC29A2 | 0.124934521 | 3.634489904 | 3.535988138 | 0.000424 | 0.001815 | -0.72470126 |
| PIGS | -0.109083619 | 3.362966094 | -3.53524595 | 0.000425 | 0.00182 | -0.72727017 |
| PIGR | 0.317211284 | 1.971766986 | 3.534855516 | 0.000426 | 0.001821 | -0.72862135 |
| VEZT | -0.092578336 | 3.366660597 | -3.53481935 | 0.000426 | 0.001821 | -0.7287465 |
| CLEC14A | -0.119374584 | 2.52268692 | -3.53395853 | 0.000427 | 0.001826 | -0.73172502 |
| TWF1 | 0.108260973 | 3.999243082 | 3.532597378 | 0.000429 | 0.001835 | -0.73643328 |
| CBLL1 | 0.089739477 | 3.319745517 | 3.531760498 | 0.00043 | 0.00184 | -0.7393272 |
| RASGRP2 | -0.10894322 | 1.895494812 | -3.531072 | 0.000432 | 0.001844 | -0.74170751 |
| RAB5A | 0.08768616 | 3.861022 | 3.530840065 | 0.000432 | 0.001845 | -0.74250926 |
| BEND5 | -0.156620355 | 2.194777807 | -3.53075017 | 0.000432 | 0.001845 | -0.74282 |
| KATNAL1 | -0.089005817 | 2.271217356 | -3.52993522 | 0.000433 | 0.00185 | -0.74563663 |
| ZNF101 | 0.087921001 | 2.646358915 | 3.529399003 | 0.000434 | 0.001853 | -0.74748958 |
| SETD3 | 0.084008255 | 3.612870066 | 3.528643594 | 0.000436 | 0.001857 | -0.75009949 |
| GSN | -0.15329323 | 4.638796983 | -3.52760013 | 0.000437 | 0.001864 | -0.75370371 |
| KCNH2 | 0.186935313 | 2.709490259 | 3.527220853 | 0.000438 | 0.001865 | -0.75501352 |
| PPP1R16B | -0.169737166 | 2.672546605 | -3.52714856 | 0.000438 | 0.001865 | -0.75526316 |
| CBLN4 | -0.147251563 | 1.238998125 | -3.52680042 | 0.000439 | 0.001867 | -0.75646529 |
| PSCA | 0.244879899 | 1.681165226 | 3.526296152 | 0.000439 | 0.001869 | -0.75820632 |
| ZNF174 | -0.082365461 | 2.694699357 | -3.52626573 | 0.000439 | 0.001869 | -0.75831134 |
| PSMB10 | 0.158290609 | 3.521437302 | 3.525065725 | 0.000441 | 0.001877 | -0.76245347 |
| LY6E | 0.201576249 | 6.191871429 | 3.5245814 | 0.000442 | 0.00188 | -0.76412485 |
| PTPRJ | -0.104461114 | 2.96627485 | -3.5237634 | 0.000444 | 0.001885 | -0.7669472 |
| SESN2 | 0.122058968 | 3.036804503 | 3.523429833 | 0.000444 | 0.001886 | -0.76809794 |
| MKRN1 | 0.079724999 | 4.30551823 | 3.52342734 | 0.000444 | 0.001886 | -0.76810654 |
| CALR | 0.113788748 | 6.369780259 | 3.522847897 | 0.000445 | 0.001889 | -0.77010523 |
| TBCEL | -0.082555669 | 2.798572396 | -3.52267752 | 0.000445 | 0.001889 | -0.77069287 |
| TNFRSF8 | -0.14478836 | 1.771950659 | -3.52226089 | 0.000446 | 0.001892 | -0.77212968 |
| PEPD | 0.126000724 | 4.034113192 | 3.521688152 | 0.000447 | 0.001895 | -0.77410462 |
| CCDC149 | -0.086349434 | 2.630088229 | -3.52110888 | 0.000448 | 0.001898 | -0.77610176 |
| HOXD4 | 0.255817496 | 2.3899614 | 3.51955037 | 0.000451 | 0.001909 | -0.78147339 |
| ZNF84 | -0.103591815 | 3.146802382 | -3.51927636 | 0.000451 | 0.00191 | -0.78241756 |
| KCTD3 | -0.094176442 | 3.684756971 | -3.51884559 | 0.000452 | 0.001912 | -0.78390176 |
| PLP2 | 0.166811256 | 4.958663738 | 3.51797804 | 0.000453 | 0.001918 | -0.7868903 |
| SMAD9 | -0.114525904 | 1.928661424 | -3.51456803 | 0.000459 | 0.001941 | -0.79863016 |
| OXSM | 0.097528811 | 3.015345707 | 3.513278441 | 0.000461 | 0.00195 | -0.80306704 |
| GOLGA5 | 0.088004401 | 3.9659506 | 3.51234249 | 0.000463 | 0.001956 | -0.8062862 |
| MRC2 | -0.145029374 | 3.988683303 | -3.51179995 | 0.000464 | 0.001959 | -0.80815187 |
| ZNF268 | -0.08361749 | 2.580903572 | -3.51027786 | 0.000466 | 0.00197 | -0.81338444 |
| ZNF593 | 0.110757881 | 2.928748741 | 3.507592175 | 0.000471 | 0.001988 | -0.82261178 |
| SLC7A10 | -0.178478395 | 1.320326994 | -3.50756433 | 0.000471 | 0.001988 | -0.8227074 |
| TMEM158 | 0.24919079 | 2.80572953 | 3.507540879 | 0.000471 | 0.001988 | -0.82278796 |
| CACYBP | 0.10054061 | 4.12269956 | 3.506876517 | 0.000472 | 0.001991 | -0.82506944 |
| HCLS1 | -0.159422169 | 3.227368139 | -3.50684046 | 0.000472 | 0.001991 | -0.82519325 |
| LRRN3 | -0.126580291 | 1.26732241 | -3.50603149 | 0.000474 | 0.001996 | -0.82797075 |
| NAT9 | -0.092332535 | 3.301870033 | -3.50522326 | 0.000475 | 0.002002 | -0.83074506 |
| CPNE3 | 0.12201667 | 4.097199852 | 3.505127489 | 0.000475 | 0.002002 | -0.83107376 |
| DSE | -0.107982138 | 2.658965905 | -3.50444874 | 0.000477 | 0.002006 | -0.83340309 |
| FGF7 | -0.155362033 | 1.923545223 | -3.50388297 | 0.000478 | 0.002009 | -0.83534435 |
| MLF1 | 0.163874302 | 3.777522031 | 3.503468527 | 0.000478 | 0.002012 | -0.83676618 |
| ZNF589 | -0.104909611 | 2.818699815 | -3.5029236 | 0.000479 | 0.002014 | -0.83863544 |
| ELP2 | -0.095769178 | 3.454329505 | -3.50291186 | 0.000479 | 0.002014 | -0.83867571 |
| ZNF124 | -0.090921914 | 2.552134844 | -3.50276128 | 0.00048 | 0.002014 | -0.83919215 |
| DOK6 | -0.101979968 | 1.483545912 | -3.50269195 | 0.00048 | 0.002014 | -0.83942996 |
| GOLIM4 | -0.129763745 | 3.573887455 | -3.50149285 | 0.000482 | 0.002023 | -0.84354192 |
| LRCH2 | -0.13415959 | 1.965797392 | -3.5007205 | 0.000483 | 0.002028 | -0.84618974 |
| ATXN1 | -0.094196037 | 3.013580828 | -3.50038497 | 0.000484 | 0.002029 | -0.84733984 |
| MPHOSPH9 | 0.086069576 | 2.781254439 | 3.499046338 | 0.000486 | 0.002039 | -0.85192724 |
| KCNIP4 | -0.19652934 | 1.569861248 | -3.49770962 | 0.000489 | 0.002048 | -0.85650636 |
| MYOZ3 | -0.115880185 | 1.44556226 | -3.49688242 | 0.00049 | 0.002054 | -0.85933921 |
| MMP9 | 0.267594343 | 3.284062673 | 3.494929074 | 0.000494 | 0.002067 | -0.86602607 |
| BCL7A | -0.11608678 | 2.971326208 | -3.4948656 | 0.000494 | 0.002067 | -0.86624328 |
| CTNNBL1 | -0.093849499 | 3.590760708 | -3.494149 | 0.000495 | 0.002072 | -0.86869546 |
| ZBED1 | -0.111998929 | 3.584617923 | -3.49390486 | 0.000496 | 0.002073 | -0.86953078 |
| SERBP1 | 0.087453171 | 4.946624381 | 3.493247609 | 0.000497 | 0.002078 | -0.87177928 |
| OAS3 | 0.195836705 | 3.92922126 | 3.492846593 | 0.000498 | 0.00208 | -0.87315098 |
| ARFGAP3 | 0.107940763 | 3.49188014 | 3.49266255 | 0.000498 | 0.00208 | -0.87378045 |
| PTGR1 | 0.128321065 | 3.740075864 | 3.492580115 | 0.000498 | 0.00208 | -0.87406239 |
| MAP7 | 0.109206705 | 3.332291711 | 3.492578295 | 0.000498 | 0.00208 | -0.87406862 |
| WIPF1 | -0.121556924 | 3.151371385 | -3.49240085 | 0.000498 | 0.00208 | -0.8746755 |
| MANBAL | -0.083093642 | 4.22572685 | -3.49150651 | 0.0005 | 0.002086 | -0.87773367 |
| TRMT2B | 0.097427799 | 3.001831535 | 3.490851801 | 0.000501 | 0.002091 | -0.87997198 |
| SAR1A | 0.079092948 | 3.967283922 | 3.489646636 | 0.000503 | 0.002099 | -0.88409108 |
| SERPINA1 | 0.226972334 | 3.569334236 | 3.48948694 | 0.000504 | 0.002099 | -0.88463679 |
| MCM2 | 0.12638403 | 4.237965294 | 3.488340063 | 0.000506 | 0.002108 | -0.88855521 |
| CACNA1H | -0.135875134 | 2.136239105 | -3.48746496 | 0.000508 | 0.002114 | -0.89154422 |
| GFOD1 | 0.158567895 | 2.807534055 | 3.486685952 | 0.000509 | 0.002119 | -0.89420442 |
| PTGES3 | 0.081956216 | 5.381841593 | 3.486150942 | 0.00051 | 0.002122 | -0.89603106 |
| SH3PXD2A | -0.12609423 | 3.503408799 | -3.48524735 | 0.000512 | 0.002129 | -0.89911547 |
| TCTA | 0.106922597 | 3.829339412 | 3.483993709 | 0.000514 | 0.002138 | -0.90339352 |
| GATAD2B | -0.074693052 | 3.785534167 | -3.48373412 | 0.000515 | 0.002139 | -0.90427916 |
| PSMA4 | 0.095095677 | 4.159777023 | 3.483628429 | 0.000515 | 0.002139 | -0.90463975 |
| CLCN7 | -0.108870371 | 3.641552215 | -3.48352659 | 0.000515 | 0.002139 | -0.90498718 |
| MRPS16 | 0.092393334 | 4.413715695 | 3.483474861 | 0.000515 | 0.002139 | -0.90516364 |
| DNAJB4 | -0.108067357 | 3.015099776 | -3.48265594 | 0.000517 | 0.002144 | -0.90795698 |
| HSPBAP1 | -0.085038064 | 2.810233923 | -3.48196233 | 0.000518 | 0.002149 | -0.91032237 |
| COL15A1 | -0.159099779 | 2.94080603 | -3.48168066 | 0.000519 | 0.00215 | -0.91128283 |
| GBP3 | 0.185189636 | 3.484073211 | 3.481169086 | 0.000519 | 0.002154 | -0.913027 |
| COG3 | -0.085611128 | 2.98820319 | -3.48049882 | 0.000521 | 0.002158 | -0.91531186 |
| CD320 | 0.148706191 | 3.824202678 | 3.480016927 | 0.000522 | 0.002161 | -0.91695429 |
| RIMKLA | 0.166981081 | 1.765144533 | 3.479264506 | 0.000523 | 0.002166 | -0.91951833 |
| TBKBP1 | -0.112805439 | 2.798624895 | -3.47716254 | 0.000527 | 0.002182 | -0.92667836 |
| TGFBRAP1 | -0.083508229 | 3.183500922 | -3.47698933 | 0.000528 | 0.002183 | -0.92726821 |
| SLC30A7 | 0.080291159 | 3.181420015 | 3.476608195 | 0.000528 | 0.002185 | -0.92856596 |
| MAGOHB | 0.103910612 | 3.103643717 | 3.476467076 | 0.000529 | 0.002186 | -0.92904644 |
| HAS3 | -0.118041056 | 1.79746035 | -3.47633018 | 0.000529 | 0.002186 | -0.92951252 |
| NOTCH2 | -0.108947057 | 4.208202539 | -3.47443793 | 0.000533 | 0.0022 | -0.93595306 |
| LRMP | -0.115612114 | 1.98307097 | -3.47225298 | 0.000537 | 0.002217 | -0.94338561 |
| CCNA1 | 0.295015013 | 3.338448783 | 3.471403061 | 0.000539 | 0.002223 | -0.94627553 |
| USP33 | 0.083675388 | 3.650754757 | 3.469116322 | 0.000543 | 0.002241 | -0.95404757 |
| SFXN3 | -0.114109136 | 3.347632573 | -3.46909538 | 0.000543 | 0.002241 | -0.95411872 |
| WDR53 | 0.103357802 | 2.963787208 | 3.468498651 | 0.000544 | 0.002245 | -0.95614602 |
| KIF3A | -0.091283105 | 2.683081415 | -3.46825895 | 0.000545 | 0.002246 | -0.95696026 |
| UQCRFS1 | 0.136710823 | 4.606577871 | 3.467792863 | 0.000546 | 0.002248 | -0.95854338 |
| LAMP2 | 0.098479191 | 4.420313882 | 3.467790665 | 0.000546 | 0.002248 | -0.95855085 |
| TSC2 | -0.114500823 | 4.045860553 | -3.46724017 | 0.000547 | 0.002252 | -0.96042039 |
| HSPB6 | -0.197806588 | 3.266151311 | -3.46601978 | 0.000549 | 0.002261 | -0.96456395 |
| CXCL14 | -0.332915732 | 2.986114283 | -3.46534313 | 0.000551 | 0.002266 | -0.96686073 |
| TCAP | -0.124829198 | 1.840624063 | -3.46484445 | 0.000552 | 0.002269 | -0.96855316 |
| LZTS2 | -0.103843863 | 4.072052369 | -3.46320563 | 0.000555 | 0.002282 | -0.97411328 |
| RPS6KA5 | -0.074949136 | 2.029797156 | -3.46203816 | 0.000557 | 0.002291 | -0.97807271 |
| PLA2G4F | -0.144877088 | 1.435656084 | -3.46142935 | 0.000559 | 0.002295 | -0.98013691 |
| CHCHD1 | 0.104996348 | 3.963398841 | 3.460069816 | 0.000561 | 0.002306 | -0.98474524 |
| ZW10 | 0.090768563 | 3.294680292 | 3.459635347 | 0.000562 | 0.002309 | -0.98621756 |
| TRMU | 0.083231296 | 2.897287263 | 3.458252254 | 0.000565 | 0.00232 | -0.99090335 |
| PSMG1 | 0.114086675 | 3.675278639 | 3.458087417 | 0.000566 | 0.00232 | -0.99146168 |
| CDH3 | -0.20811641 | 3.663888995 | -3.45668832 | 0.000568 | 0.002331 | -0.99619962 |
| ZNF511 | 0.102336073 | 3.278283471 | 3.454919734 | 0.000572 | 0.002346 | -1.0021861 |
| CCDC18 | 0.08761938 | 2.3041231 | 3.453786007 | 0.000575 | 0.002355 | -1.00602209 |
| ERGIC2 | 0.102814375 | 3.691573322 | 3.453442255 | 0.000575 | 0.002357 | -1.00718493 |
| SS18L2 | 0.107351474 | 3.520899824 | 3.453249993 | 0.000576 | 0.002357 | -1.00783527 |
| TEX101 | -0.11222746 | 1.879407076 | -3.45319043 | 0.000576 | 0.002357 | -1.00803674 |
| TAX1BP1 | 0.102708909 | 3.980741308 | 3.452745271 | 0.000577 | 0.00236 | -1.00954235 |
| RASGRP4 | -0.111445868 | 1.722837071 | -3.45237327 | 0.000578 | 0.002362 | -1.01080038 |
| ABHD3 | 0.143821277 | 2.661321919 | 3.452236368 | 0.000578 | 0.002363 | -1.01126332 |
| CMKLR1 | -0.123777726 | 2.352105328 | -3.45191984 | 0.000578 | 0.002365 | -1.01233362 |
| CALML4 | -0.109862661 | 2.279594869 | -3.45165547 | 0.000579 | 0.002366 | -1.01322747 |
| RFWD3 | 0.0976603 | 3.132390248 | 3.451077006 | 0.00058 | 0.00237 | -1.01518304 |
| TMED2 | 0.098158139 | 5.16615458 | 3.449893214 | 0.000583 | 0.00238 | -1.01918403 |
| WDR3 | 0.09653103 | 3.223558562 | 3.449647948 | 0.000583 | 0.002381 | -1.02001282 |
| PLEKHO1 | -0.116573772 | 3.519914805 | -3.44931163 | 0.000584 | 0.002383 | -1.02114918 |
| PRKCB | -0.119666349 | 2.05931098 | -3.44873071 | 0.000585 | 0.002387 | -1.02311178 |
| NTF3 | -0.240691856 | 2.090336301 | -3.44765362 | 0.000588 | 0.002396 | -1.02674977 |
| PDSS1 | 0.098984774 | 2.755468487 | 3.447151841 | 0.000589 | 0.002399 | -1.0284442 |
| PLA2G7 | -0.174569267 | 2.262206865 | -3.44642527 | 0.00059 | 0.002405 | -1.03089731 |
| MED10 | 0.105509157 | 3.721685976 | 3.44535792 | 0.000593 | 0.002413 | -1.03450004 |
| RBMS1 | -0.08816183 | 3.814754603 | -3.44520471 | 0.000593 | 0.002414 | -1.0350171 |
| PRR16 | 0.148743415 | 1.681463851 | 3.444973204 | 0.000593 | 0.002415 | -1.03579835 |
| CENPO | 0.10375428 | 2.829396296 | 3.444561302 | 0.000594 | 0.002418 | -1.03718825 |
| TGFB2 | -0.192635946 | 2.604500081 | -3.44377477 | 0.000596 | 0.002424 | -1.03984182 |
| ZBTB37 | -0.067512087 | 2.451432569 | -3.44341031 | 0.000597 | 0.002426 | -1.04107122 |
| KCNN3 | -0.126811967 | 2.180982132 | -3.4428332 | 0.000598 | 0.00243 | -1.04301766 |
| GZMB | 0.223270051 | 1.969338754 | 3.441105695 | 0.000602 | 0.002445 | -1.04884222 |
| VAMP8 | 0.113868336 | 5.164517124 | 3.441012846 | 0.000602 | 0.002445 | -1.04915519 |
| NPR2 | -0.123842807 | 2.909826742 | -3.44081919 | 0.000602 | 0.002445 | -1.04980793 |
| APOL6 | 0.161812114 | 3.275938288 | 3.439387119 | 0.000606 | 0.002457 | -1.05463382 |
| NOTCH4 | -0.082455879 | 2.526907154 | -3.43875878 | 0.000607 | 0.002462 | -1.05675062 |
| C2CD2 | -0.095777243 | 3.049254538 | -3.43854313 | 0.000607 | 0.002462 | -1.05747702 |
| NID2 | -0.164641665 | 2.848804725 | -3.43852338 | 0.000608 | 0.002462 | -1.05754355 |
| SETX | -0.076074771 | 3.430867493 | -3.4382523 | 0.000608 | 0.002464 | -1.0584566 |
| STAT3 | 0.099000641 | 4.487655539 | 3.437452651 | 0.00061 | 0.00247 | -1.06114961 |
| CYTH3 | -0.108575656 | 3.197202921 | -3.43598234 | 0.000613 | 0.002483 | -1.06609959 |
| WFIKKN2 | -0.136287634 | 1.136733776 | -3.43535235 | 0.000615 | 0.002487 | -1.06821989 |
| ABCC5 | -0.103358672 | 3.346899737 | -3.43466245 | 0.000616 | 0.002493 | -1.07054139 |
| EMX2 | -0.243583928 | 4.589504631 | -3.43414367 | 0.000617 | 0.002497 | -1.0722868 |
| DDX1 | -0.085263161 | 4.217565067 | -3.43215072 | 0.000622 | 0.002514 | -1.07898949 |
| HMGCR | 0.102598794 | 3.270237551 | 3.428886346 | 0.000629 | 0.002543 | -1.08996002 |
| GJB1 | 0.251034713 | 3.0267833 | 3.428814799 | 0.000629 | 0.002543 | -1.09020035 |
| PHKG2 | 0.085022292 | 3.202700616 | 3.427840334 | 0.000632 | 0.002551 | -1.09347318 |
| KLK14 | 0.196457213 | 2.359434141 | 3.42747217 | 0.000633 | 0.002553 | -1.09470945 |
| TRDN | -0.160511596 | 1.17233999 | -3.42619788 | 0.000635 | 0.002564 | -1.09898745 |
| ABCB9 | 0.094653241 | 2.386679601 | 3.425678711 | 0.000637 | 0.002568 | -1.10072993 |
| SNX21 | -0.095590416 | 2.923564601 | -3.42504275 | 0.000638 | 0.002573 | -1.10286406 |
| ATF7IP | -0.085769721 | 3.729738982 | -3.4244622 | 0.00064 | 0.002578 | -1.1048119 |
| EED | 0.090894493 | 3.094456719 | 3.424124392 | 0.00064 | 0.00258 | -1.10594514 |
| SGCB | 0.117696168 | 3.256495856 | 3.423588392 | 0.000642 | 0.002584 | -1.10774305 |
| ZFYVE16 | -0.076670894 | 2.84754403 | -3.42120456 | 0.000647 | 0.002606 | -1.1157358 |
| ZNF496 | -0.101201014 | 3.485825584 | -3.4210678 | 0.000647 | 0.002606 | -1.11619417 |
| CISD1 | 0.099169688 | 3.503640124 | 3.420504975 | 0.000649 | 0.00261 | -1.11808041 |
| CAPN9 | -0.202992466 | 1.999675084 | -3.41954405 | 0.000651 | 0.002619 | -1.12130013 |
| ACAP1 | -0.112745303 | 2.281310307 | -3.41934926 | 0.000652 | 0.002619 | -1.12195269 |
| RABAC1 | 0.132863616 | 4.508840325 | 3.419316841 | 0.000652 | 0.002619 | -1.12206129 |
| ERH | 0.107657715 | 5.091845707 | 3.419160963 | 0.000652 | 0.002619 | -1.12258346 |
| SLC9A9 | -0.100637879 | 2.173771503 | -3.41873452 | 0.000653 | 0.002623 | -1.12401186 |
| KALRN | -0.098684734 | 2.640885479 | -3.41851082 | 0.000653 | 0.002624 | -1.12476112 |
| CSNK2B | 0.109499408 | 4.43045613 | 3.416884303 | 0.000657 | 0.002638 | -1.13020734 |
| MAL | 0.376619885 | 3.93723019 | 3.41637409 | 0.000659 | 0.002642 | -1.13191521 |
| RASSF4 | -0.118549486 | 2.967855729 | -3.41405242 | 0.000664 | 0.002664 | -1.13968357 |
| CIC | -0.127520358 | 4.287507735 | -3.41341418 | 0.000666 | 0.002669 | -1.14181822 |
| CLEC4A | -0.155537441 | 1.983216125 | -3.41274119 | 0.000667 | 0.002674 | -1.14406869 |
| ARL4A | -0.121066362 | 2.646230231 | -3.41254672 | 0.000668 | 0.002675 | -1.14471893 |
| FAM118B | 0.097222722 | 2.909264035 | 3.412338994 | 0.000668 | 0.002676 | -1.14541342 |
| GAL3ST4 | -0.128073886 | 2.873967249 | -3.41197441 | 0.000669 | 0.002679 | -1.14663226 |
| XKR8 | -0.087616213 | 2.797928976 | -3.41171503 | 0.00067 | 0.002681 | -1.1474993 |
| HYDIN | -0.087850442 | 2.299329612 | -3.41091161 | 0.000672 | 0.002687 | -1.15018456 |
| FGFBP3 | -0.108841937 | 1.825734939 | -3.41026163 | 0.000673 | 0.002693 | -1.15235652 |
| RNF139 | 0.116363318 | 3.905201613 | 3.409431853 | 0.000675 | 0.0027 | -1.15512872 |
| NDUFB2 | 0.101556524 | 4.163905105 | 3.408896954 | 0.000677 | 0.002704 | -1.1569154 |
| FLOT2 | -0.108071774 | 4.236462324 | -3.40837013 | 0.000678 | 0.002708 | -1.15867484 |
| EXOC7 | -0.091602131 | 3.927840306 | -3.40795269 | 0.000679 | 0.002711 | -1.16006878 |
| TRAM1L1 | -0.144217777 | 2.469873796 | -3.40790985 | 0.000679 | 0.002711 | -1.16021183 |
| CFP | -0.112331519 | 1.547313426 | -3.40768302 | 0.00068 | 0.002712 | -1.16096918 |
| CDK10 | -0.108858753 | 3.366056211 | -3.40736963 | 0.00068 | 0.002714 | -1.16201551 |
| ACE2 | 0.16539514 | 1.248015473 | 3.407150756 | 0.000681 | 0.002715 | -1.16274618 |
| HGD | 0.202431797 | 1.614264823 | 3.407088472 | 0.000681 | 0.002715 | -1.1629541 |
| PRKAB2 | -0.097796122 | 2.968695579 | -3.40658734 | 0.000682 | 0.002719 | -1.16462688 |
| APOE | -0.196717819 | 5.268105765 | -3.40613222 | 0.000684 | 0.002722 | -1.16614587 |
| COL21A1 | -0.143398575 | 1.40542955 | -3.40605978 | 0.000684 | 0.002722 | -1.1663876 |
| MEF2A | -0.091876886 | 3.459451301 | -3.40513177 | 0.000686 | 0.00273 | -1.16948421 |
| METTL7B | 0.204336689 | 3.259177143 | 3.404091428 | 0.000689 | 0.00274 | -1.17295464 |
| SUCLG2 | 0.09689003 | 4.010298647 | 3.40397606 | 0.000689 | 0.00274 | -1.17333942 |
| SLC44A4 | 0.219967952 | 3.803774649 | 3.402917902 | 0.000692 | 0.002749 | -1.17686811 |
| BCLAF1 | -0.075688406 | 3.912900303 | -3.4026087 | 0.000692 | 0.002752 | -1.17789903 |
| MAP3K14 | -0.092333562 | 2.709174257 | -3.40197803 | 0.000694 | 0.002757 | -1.18000143 |
| SLC13A3 | -0.124649943 | 1.981622663 | -3.3996949 | 0.0007 | 0.002779 | -1.18760938 |
| RAB5C | 0.098561619 | 4.197211446 | 3.397930332 | 0.000704 | 0.002795 | -1.19348593 |
| LLPH | 0.094290212 | 2.768817286 | 3.397700791 | 0.000705 | 0.002797 | -1.19425015 |
| STEAP1 | 0.16553569 | 3.091039317 | 3.397339986 | 0.000706 | 0.002799 | -1.19545129 |
| ZNF740 | -0.086095289 | 3.135045409 | -3.39709395 | 0.000706 | 0.002801 | -1.1962703 |
| PCSK5 | -0.128032703 | 2.351619037 | -3.39499088 | 0.000712 | 0.002821 | -1.20326856 |
| BLK | -0.132363184 | 1.314815823 | -3.39477983 | 0.000712 | 0.002822 | -1.20397063 |
| DNAJC3 | 0.109518167 | 3.380597683 | 3.394482155 | 0.000713 | 0.002824 | -1.20496079 |
| RILPL1 | -0.079478854 | 2.778203029 | -3.39298832 | 0.000717 | 0.002839 | -1.20992844 |
| PLA2G6 | -0.117451738 | 3.025202488 | -3.39288023 | 0.000717 | 0.002839 | -1.21028782 |
| KPNA1 | 0.07482012 | 3.462884031 | 3.392300892 | 0.000719 | 0.002844 | -1.21221371 |
| DPP3 | 0.097556205 | 3.886507399 | 3.391462394 | 0.000721 | 0.002851 | -1.2150006 |
| BTAF1 | -0.102282595 | 3.263209249 | -3.39128633 | 0.000721 | 0.002852 | -1.21558568 |
| PLA2G15 | -0.088001987 | 3.091064531 | -3.38745715 | 0.000731 | 0.002891 | -1.22830334 |
| DUSP1 | -0.228570218 | 4.81135346 | -3.38725584 | 0.000732 | 0.002892 | -1.22897157 |
| REV1 | -0.070711197 | 3.133887064 | -3.38718642 | 0.000732 | 0.002892 | -1.22920199 |
| ETV1 | -0.134476833 | 2.356180834 | -3.38702562 | 0.000732 | 0.002892 | -1.22973568 |
| SLC25A16 | 0.084977493 | 2.815067896 | 3.386920675 | 0.000733 | 0.002892 | -1.230084 |
| TNNT3 | -0.190998076 | 1.59627127 | -3.38651793 | 0.000734 | 0.002895 | -1.23142059 |
| ZNRF1 | -0.089645453 | 3.231157688 | -3.38493694 | 0.000738 | 0.002911 | -1.23666594 |
| ARHGEF11 | -0.084684653 | 3.635348637 | -3.38479329 | 0.000738 | 0.002911 | -1.2371424 |
| C2orf69 | 0.096160524 | 3.03393745 | 3.384492303 | 0.000739 | 0.002914 | -1.23814071 |
| ST6GALNAC1 | 0.243724767 | 3.019024577 | 3.381620386 | 0.000747 | 0.002943 | -1.24766167 |
| PRR3 | -0.099257856 | 3.222089196 | -3.38113865 | 0.000748 | 0.002947 | -1.24925796 |
| STYXL1 | 0.103272982 | 3.969330079 | 3.380299187 | 0.00075 | 0.002955 | -1.25203905 |
| EBI3 | -0.169893035 | 1.915139019 | -3.3780679 | 0.000756 | 0.002976 | -1.25942793 |
| ACTR3 | 0.097749292 | 4.42158088 | 3.378063933 | 0.000756 | 0.002976 | -1.25944105 |
| BGN | -0.202146494 | 5.271783357 | -3.37772527 | 0.000757 | 0.002979 | -1.2605621 |
| ZNF652 | -0.088438879 | 3.314735739 | -3.37700791 | 0.000759 | 0.002986 | -1.2629364 |
| ALDH3A1 | -0.178779326 | 1.735592167 | -3.37673665 | 0.00076 | 0.002987 | -1.26383407 |
| PDAP1 | 0.088064179 | 4.460177376 | 3.376395786 | 0.000761 | 0.00299 | -1.26496197 |
| MVD | 0.096862359 | 2.854071656 | 3.37623593 | 0.000761 | 0.00299 | -1.26549089 |
| PAFAH1B3 | 0.13935172 | 4.068392268 | 3.376233723 | 0.000761 | 0.00299 | -1.26549819 |
| MARVELD3 | 0.100966004 | 2.643392885 | 3.375518653 | 0.000763 | 0.002996 | -1.26786387 |
| ZNF34 | 0.109272094 | 2.769955039 | 3.374935702 | 0.000765 | 0.003001 | -1.26979208 |
| UPF3A | -0.103414704 | 3.484026429 | -3.37492469 | 0.000765 | 0.003001 | -1.26982851 |
| TOR1AIP1 | -0.091189073 | 3.453391869 | -3.37457155 | 0.000766 | 0.003003 | -1.27099641 |
| PAPOLA | 0.079329828 | 4.22940497 | 3.373597725 | 0.000769 | 0.003013 | -1.27421646 |
| ARMC1 | 0.098589862 | 3.822822488 | 3.372110074 | 0.000773 | 0.003028 | -1.27913376 |
| TMEM80 | -0.098084311 | 3.124707635 | -3.37200976 | 0.000773 | 0.003028 | -1.27946526 |
| TES | 0.108945544 | 4.071578895 | 3.369228197 | 0.000781 | 0.003057 | -1.28865352 |
| PABPC5 | -0.117821811 | 1.11721456 | -3.36910878 | 0.000781 | 0.003057 | -1.28904781 |
| CLK2 | -0.090873885 | 4.205513113 | -3.36867624 | 0.000782 | 0.003061 | -1.29047591 |
| G6PD | 0.100298597 | 3.734856971 | 3.366917383 | 0.000787 | 0.003079 | -1.29628112 |
| APC | -0.071710895 | 2.625941379 | -3.36674892 | 0.000788 | 0.00308 | -1.29683697 |
| GTF2F1 | -0.082159795 | 4.024821052 | -3.3664791 | 0.000789 | 0.003082 | -1.29772723 |
| KCTD1 | 0.144182314 | 3.705652433 | 3.365235733 | 0.000792 | 0.003095 | -1.30182873 |
| KANK3 | -0.103152557 | 1.720872214 | -3.36240284 | 0.0008 | 0.003125 | -1.31116808 |
| DLG3 | 0.0878355 | 3.324274742 | 3.361082656 | 0.000804 | 0.003139 | -1.31551775 |
| TMUB1 | 0.099507727 | 4.182896592 | 3.360625701 | 0.000805 | 0.003143 | -1.31702291 |
| KIF20B | 0.101828639 | 2.942552886 | 3.359741163 | 0.000808 | 0.003152 | -1.31993593 |
| CD27 | -0.172730967 | 2.125216438 | -3.3587965 | 0.000811 | 0.003161 | -1.32304611 |
| WWC2 | -0.088021712 | 2.604101495 | -3.35759179 | 0.000814 | 0.003174 | -1.32701123 |
| WDR91 | 0.115084907 | 3.512349594 | 3.357332735 | 0.000815 | 0.003176 | -1.3278637 |
| SELPLG | -0.152227875 | 2.910187464 | -3.35713597 | 0.000815 | 0.003176 | -1.32851113 |
| NKAIN4 | -0.247668999 | 1.754054541 | -3.35710108 | 0.000816 | 0.003176 | -1.32862594 |
| ASPN | -0.244271773 | 2.160799427 | -3.35690293 | 0.000816 | 0.003177 | -1.32927788 |
| DTX1 | -0.121812169 | 2.100663159 | -3.35659475 | 0.000817 | 0.00318 | -1.3302918 |
| SMARCD1 | -0.091058364 | 3.95306056 | -3.35567627 | 0.00082 | 0.003189 | -1.33331301 |
| PRDX5 | 0.102351897 | 5.780609462 | 3.354457231 | 0.000823 | 0.003202 | -1.33732165 |
| TIMM9 | 0.093570418 | 3.73214362 | 3.353737595 | 0.000825 | 0.003207 | -1.3396874 |
| BAZ1A | 0.102610035 | 3.517822567 | 3.353711357 | 0.000825 | 0.003207 | -1.33977364 |
| CLEC1A | -0.108551437 | 1.6558084 | -3.35365247 | 0.000826 | 0.003207 | -1.3399672 |
| APOOL | 0.097634283 | 2.704511086 | 3.353571052 | 0.000826 | 0.003207 | -1.34023482 |
| PLEKHA5 | -0.100515225 | 2.936482334 | -3.35320246 | 0.000827 | 0.003211 | -1.34144628 |
| DPYSL5 | -0.192305571 | 1.261143916 | -3.35283414 | 0.000828 | 0.003214 | -1.34265672 |
| SCN2B | -0.134464589 | 1.723683417 | -3.35234332 | 0.00083 | 0.003218 | -1.34426955 |
| JAGN1 | 0.090353643 | 4.059401931 | 3.3517966 | 0.000831 | 0.003223 | -1.34606579 |
| ZNF544 | 0.087868419 | 3.275572571 | 3.351497671 | 0.000832 | 0.003226 | -1.34704779 |
| FBXW7 | -0.065128572 | 2.619964413 | -3.35122786 | 0.000833 | 0.003228 | -1.34793407 |
| FOXA2 | 0.23293802 | 1.910355899 | 3.351064869 | 0.000833 | 0.003228 | -1.34846942 |
| RFX2 | -0.101142142 | 2.406608954 | -3.35042771 | 0.000835 | 0.003235 | -1.35056199 |
| RXRB | -0.090122368 | 3.778067727 | -3.3500142 | 0.000836 | 0.003238 | -1.35191983 |
| SKP2 | 0.111492197 | 3.417134309 | 3.349979225 | 0.000837 | 0.003238 | -1.35203467 |
| PSME3 | 0.080221134 | 4.126059631 | 3.349760106 | 0.000837 | 0.003239 | -1.35275411 |
| LRRC40 | 0.08324171 | 3.05836139 | 3.349321808 | 0.000839 | 0.003243 | -1.35419307 |
| SPEN | -0.093489684 | 3.955415881 | -3.34887755 | 0.00084 | 0.003247 | -1.3556514 |
| ID2 | -0.144430878 | 3.904624032 | -3.34861751 | 0.000841 | 0.003249 | -1.35650491 |
| ROBO4 | -0.090900856 | 2.212642612 | -3.34830487 | 0.000842 | 0.003251 | -1.35753101 |
| SALL2 | -0.149507044 | 3.109052996 | -3.34688852 | 0.000846 | 0.003267 | -1.36217832 |
| SNIP1 | 0.085666082 | 2.954632742 | 3.346737432 | 0.000846 | 0.003267 | -1.36267395 |
| DOLK | 0.100353741 | 3.149492981 | 3.345972638 | 0.000849 | 0.003275 | -1.36518247 |
| RUFY1 | -0.075319961 | 3.595077027 | -3.34571383 | 0.000849 | 0.003276 | -1.36603122 |
| YIPF5 | 0.085705573 | 3.723797513 | 3.345693148 | 0.000849 | 0.003276 | -1.36609906 |
| IKZF4 | -0.083603384 | 2.571386457 | -3.34436446 | 0.000853 | 0.00329 | -1.37045546 |
| SERPING1 | -0.158983598 | 5.566396007 | -3.34389977 | 0.000855 | 0.003295 | -1.37197864 |
| PNPO | 0.108455192 | 3.546457614 | 3.341289342 | 0.000863 | 0.003324 | -1.38053141 |
| EFNB3 | -0.168527362 | 2.66151079 | -3.34014938 | 0.000866 | 0.003337 | -1.38426432 |
| TIE1 | -0.105737618 | 2.363344323 | -3.33879948 | 0.000871 | 0.003352 | -1.38868306 |
| PARP9 | 0.133371114 | 3.957589629 | 3.338474609 | 0.000872 | 0.003354 | -1.38974622 |
| GLRX3 | 0.091341749 | 3.607997279 | 3.337800836 | 0.000874 | 0.003361 | -1.39195088 |
| BICD1 | -0.136310055 | 3.618864411 | -3.33769954 | 0.000874 | 0.003361 | -1.39228228 |
| ZNF7 | 0.092391902 | 3.092832389 | 3.337355632 | 0.000875 | 0.003364 | -1.3934074 |
| TM9SF2 | 0.094728219 | 4.904393967 | 3.335745957 | 0.00088 | 0.003382 | -1.39867197 |
| SPPL2A | 0.091768623 | 3.286248212 | 3.334999207 | 0.000882 | 0.00339 | -1.40111344 |
| MS4A14 | -0.092099749 | 1.798027057 | -3.33475598 | 0.000883 | 0.003392 | -1.40190853 |
| DMTF1 | -0.091931162 | 3.535841626 | -3.33421724 | 0.000885 | 0.003397 | -1.40366947 |
| TPMT | 0.113014556 | 3.337947898 | 3.334053234 | 0.000885 | 0.003398 | -1.40420549 |
| DIP2C | -0.107181589 | 2.888446328 | -3.33219121 | 0.000891 | 0.00342 | -1.41028925 |
| TBRG1 | -0.072345143 | 2.995972171 | -3.33178464 | 0.000893 | 0.003423 | -1.41161719 |
| FIGNL1 | 0.095781996 | 2.862655894 | 3.331735097 | 0.000893 | 0.003423 | -1.41177901 |
| ARHGAP21 | -0.093092664 | 3.418441823 | -3.33155347 | 0.000893 | 0.003423 | -1.41237217 |
| WNT5B | -0.167344856 | 2.005399416 | -3.33148084 | 0.000894 | 0.003423 | -1.41260937 |
| IFIH1 | 0.166957891 | 3.289814955 | 3.331309068 | 0.000894 | 0.003424 | -1.41317032 |
| ZNF441 | -0.092763586 | 2.25438111 | -3.33119773 | 0.000894 | 0.003425 | -1.4135339 |
| CNOT10 | 0.073721038 | 3.466285042 | 3.330703733 | 0.000896 | 0.003429 | -1.41514689 |
| FNDC5 | -0.12947167 | 1.647608735 | -3.33065736 | 0.000896 | 0.003429 | -1.41529831 |
| PLEKHF1 | 0.16496169 | 3.321827115 | 3.328841357 | 0.000902 | 0.00345 | -1.42122584 |
| VAT1 | -0.115164912 | 4.409236485 | -3.32866653 | 0.000903 | 0.003451 | -1.42179631 |
| PUM2 | -0.077586765 | 4.165915351 | -3.32829393 | 0.000904 | 0.003453 | -1.42301206 |
| GNRH2 | -0.124010502 | 1.350793664 | -3.32828515 | 0.000904 | 0.003453 | -1.42304071 |
| PDCD5 | 0.119871899 | 4.3544194 | 3.328044367 | 0.000905 | 0.003455 | -1.42382626 |
| LOXL2 | 0.148093079 | 3.162186863 | 3.326613451 | 0.000909 | 0.003471 | -1.42849352 |
| LIN7A | -0.100697008 | 1.464770703 | -3.32614248 | 0.000911 | 0.003476 | -1.43002928 |
| PES1 | 0.091786674 | 3.730291013 | 3.325363957 | 0.000913 | 0.003484 | -1.43256743 |
| SLC39A10 | -0.12167784 | 3.799746273 | -3.32529047 | 0.000913 | 0.003484 | -1.43280697 |
| GPHA2 | -0.128424664 | 1.316801573 | -3.32467939 | 0.000915 | 0.00349 | -1.43479877 |
| HUS1 | 0.07453008 | 3.026290496 | 3.324583327 | 0.000916 | 0.00349 | -1.43511187 |
| ZNF133 | -0.085083769 | 3.093706276 | -3.32386457 | 0.000918 | 0.003498 | -1.43745413 |
| RAB11FIP3 | -0.100239154 | 3.317965852 | -3.3229915 | 0.000921 | 0.003508 | -1.44029858 |
| BMF | -0.139940097 | 3.231221069 | -3.32233346 | 0.000923 | 0.003515 | -1.44244197 |
| DOLPP1 | 0.096047286 | 3.133043385 | 3.321705428 | 0.000925 | 0.003521 | -1.44448725 |
| SEC63 | -0.08596848 | 3.696636737 | -3.32061876 | 0.000929 | 0.003534 | -1.44802523 |
| PTGDR | -0.089269709 | 1.3870649 | -3.32024801 | 0.00093 | 0.003537 | -1.44923208 |
| PLXNB3 | 0.137159926 | 1.795604571 | 3.319740016 | 0.000932 | 0.003542 | -1.45088543 |
| MAD2L2 | 0.133624805 | 3.546692075 | 3.319000969 | 0.000934 | 0.00355 | -1.45329036 |
| GPR162 | -0.168281424 | 2.257259696 | -3.31798139 | 0.000937 | 0.003561 | -1.4566073 |
| CAMLG | -0.098336694 | 3.949302331 | -3.3179564 | 0.000937 | 0.003561 | -1.45668858 |
| DNASE1 | -0.086773013 | 2.551980799 | -3.31640245 | 0.000943 | 0.003579 | -1.46174201 |
| BACH1 | -0.077887816 | 3.109486444 | -3.31444176 | 0.000949 | 0.003603 | -1.46811484 |
| KLHL4 | -0.116269246 | 1.488447339 | -3.31418235 | 0.00095 | 0.003605 | -1.46895773 |
| ANKRD13A | -0.09996112 | 3.460470084 | -3.31388643 | 0.000951 | 0.003608 | -1.46991918 |
| SORCS2 | -0.156864837 | 2.113206652 | -3.31262984 | 0.000955 | 0.003623 | -1.47400084 |
| GYPC | -0.140053015 | 3.169612983 | -3.31227649 | 0.000957 | 0.003626 | -1.47514835 |
| LGI2 | -0.132381442 | 1.836340966 | -3.31189964 | 0.000958 | 0.003629 | -1.47637202 |
| RASL12 | -0.12253686 | 2.256527891 | -3.31150443 | 0.000959 | 0.003632 | -1.47765516 |
| PPP3CB | -0.073316646 | 3.658758888 | -3.31149148 | 0.000959 | 0.003632 | -1.47769719 |
| MYH3 | -0.088340247 | 1.810527187 | -3.31139851 | 0.00096 | 0.003632 | -1.47799903 |
| DDA1 | 0.100168601 | 3.839996873 | 3.309536183 | 0.000966 | 0.003655 | -1.48404329 |
| MLH3 | -0.074917778 | 2.911551562 | -3.30859994 | 0.000969 | 0.003666 | -1.48708064 |
| NUMA1 | -0.103992675 | 4.478362111 | -3.30792354 | 0.000971 | 0.003673 | -1.48927452 |
| WDR37 | -0.08587745 | 3.020108905 | -3.30766372 | 0.000972 | 0.003675 | -1.49011709 |
| NUDCD2 | 0.084992836 | 3.022048341 | 3.307260118 | 0.000974 | 0.003679 | -1.49142584 |
| CHMP1B | 0.102349472 | 4.152261874 | 3.306265541 | 0.000977 | 0.003691 | -1.49465025 |
| TNFAIP6 | 0.185263742 | 2.241357901 | 3.305149498 | 0.000981 | 0.003704 | -1.49826731 |
| FIBP | 0.092055374 | 4.347424408 | 3.304943033 | 0.000982 | 0.003706 | -1.49893633 |
| PDE1B | -0.094009437 | 2.07196239 | -3.30467525 | 0.000983 | 0.003708 | -1.49980397 |
| CD83 | -0.115111149 | 3.145352316 | -3.30381178 | 0.000986 | 0.003718 | -1.50260124 |
| MAGED2 | -0.105483181 | 5.002945499 | -3.30364378 | 0.000986 | 0.003719 | -1.50314542 |
| BCAS3 | -0.08600848 | 2.923397538 | -3.30286327 | 0.000989 | 0.003728 | -1.50567317 |
| GABARAPL1 | -0.1259403 | 3.367386954 | -3.30244026 | 0.00099 | 0.003732 | -1.5070429 |
| FBXO32 | 0.183580144 | 3.771798969 | 3.302246006 | 0.000991 | 0.003734 | -1.50767185 |
| ARHGAP4 | -0.140650824 | 3.556891574 | -3.30213672 | 0.000991 | 0.003734 | -1.50802567 |
| IGDCC3 | -0.167153106 | 1.318301214 | -3.30070431 | 0.000996 | 0.003751 | -1.51266218 |
| PROCA1 | -0.106202517 | 2.128127489 | -3.30033772 | 0.000998 | 0.003755 | -1.51384848 |
| PDIA3 | 0.104057557 | 5.43855592 | 3.300243574 | 0.000998 | 0.003755 | -1.5141531 |
| GOLGA7 | 0.098378973 | 4.271694497 | 3.299887072 | 0.000999 | 0.003758 | -1.51530658 |
| RFT1 | 0.078598939 | 3.141771035 | 3.299391166 | 0.001001 | 0.003764 | -1.51691091 |
| BPGM | 0.106219891 | 3.303997893 | 3.299023693 | 0.001002 | 0.003767 | -1.51809959 |
| IFNAR2 | 0.090940865 | 3.273205064 | 3.298907684 | 0.001003 | 0.003768 | -1.51847482 |
| ZNF333 | -0.090997957 | 2.587059048 | -3.29824382 | 0.001005 | 0.003775 | -1.52062184 |
| C1QBP | 0.110916913 | 4.567192704 | 3.29802147 | 0.001006 | 0.003777 | -1.52134085 |
| AQP1 | -0.136070935 | 3.317798703 | -3.29624833 | 0.001012 | 0.003799 | -1.52707297 |
| CFDP1 | -0.090544986 | 3.479722544 | -3.29583878 | 0.001014 | 0.003802 | -1.52839651 |
| TNFAIP8L2 | -0.151388623 | 2.23871057 | -3.29576463 | 0.001014 | 0.003802 | -1.52863611 |
| C9orf78 | 0.085885085 | 3.630954477 | 3.295729996 | 0.001014 | 0.003802 | -1.52874803 |
| RASGRF2 | -0.105449507 | 2.202439204 | -3.29317392 | 0.001023 | 0.003835 | -1.53700458 |
| TUBE1 | -0.085876071 | 2.446323475 | -3.29302365 | 0.001024 | 0.003836 | -1.53748978 |
| BAG1 | 0.115183045 | 3.680092852 | 3.292360841 | 0.001026 | 0.003842 | -1.53962964 |
| FGF1 | -0.129951862 | 1.915105133 | -3.29230963 | 0.001026 | 0.003842 | -1.53979494 |
| NCK1 | 0.103238693 | 3.68391379 | 3.292292613 | 0.001026 | 0.003842 | -1.53984988 |
| BHMT2 | -0.170169411 | 1.655285008 | -3.29058893 | 0.001033 | 0.003864 | -1.54534814 |
| NARS2 | 0.10716012 | 3.550918694 | 3.29011565 | 0.001034 | 0.003869 | -1.54687504 |
| TCEA1 | 0.083025587 | 4.028473902 | 3.289974502 | 0.001035 | 0.00387 | -1.54733037 |
| BCL2L12 | 0.111518651 | 3.34930031 | 3.288372999 | 0.001041 | 0.00389 | -1.55249537 |
| CH25H | -0.17493273 | 1.645248049 | -3.28766744 | 0.001043 | 0.003898 | -1.55477008 |
| CNOT4 | 0.063444517 | 3.21763052 | 3.287015258 | 0.001046 | 0.003906 | -1.55687229 |
| EPHB4 | -0.132982234 | 3.654704249 | -3.28652696 | 0.001047 | 0.003911 | -1.55844596 |
| ATXN7L1 | -0.071195238 | 2.555088189 | -3.28633276 | 0.001048 | 0.003913 | -1.55907178 |
| ADAM23 | -0.142751253 | 1.481530992 | -3.28623956 | 0.001049 | 0.003913 | -1.5593721 |
| RPL3 | -0.097086334 | 6.627531019 | -3.28343615 | 0.001059 | 0.00395 | -1.56840168 |
| TGM5 | 0.116515518 | 1.178695945 | 3.281749418 | 0.001065 | 0.003972 | -1.57383088 |
| FOXA1 | 0.233029311 | 1.274687415 | 3.281442653 | 0.001066 | 0.003975 | -1.574818 |
| ENAH | -0.111045237 | 3.857655425 | -3.28128802 | 0.001067 | 0.003976 | -1.57531554 |
| DONSON | 0.11001419 | 2.870559343 | 3.280933648 | 0.001068 | 0.00398 | -1.57645568 |
| GRIN2A | -0.198645984 | 1.879244507 | -3.2807597 | 0.001069 | 0.003981 | -1.57701528 |
| TNIP3 | 0.135519898 | 1.373893424 | 3.279995957 | 0.001072 | 0.00399 | -1.57947198 |
| ANGEL2 | -0.076077449 | 3.145440443 | -3.27958824 | 0.001073 | 0.003994 | -1.58078321 |
| AGFG1 | 0.076034388 | 3.676778434 | 3.278652865 | 0.001077 | 0.004006 | -1.58379087 |
| MAP3K5 | -0.10087233 | 2.787182689 | -3.27785406 | 0.00108 | 0.004015 | -1.58635872 |
| PXN | -0.093436432 | 4.106744869 | -3.27784244 | 0.00108 | 0.004015 | -1.58639607 |
| FMO5 | -0.094865606 | 1.647573144 | -3.27697541 | 0.001083 | 0.004026 | -1.5891825 |
| CPXM2 | -0.23793664 | 2.49360376 | -3.27489923 | 0.001091 | 0.004054 | -1.595852 |
| CMTM8 | 0.11966558 | 3.584503258 | 3.272863188 | 0.001099 | 0.004081 | -1.60238851 |
| ZDHHC4 | -0.094106223 | 3.563899535 | -3.27220647 | 0.001101 | 0.00409 | -1.604496 |
| PKP3 | 0.145761563 | 3.970695985 | 3.271561786 | 0.001104 | 0.004097 | -1.60656445 |
| RPL26L1 | 0.103842932 | 3.773563084 | 3.271161958 | 0.001105 | 0.004102 | -1.60784708 |
| CCDC148 | -0.102009713 | 1.805843997 | -3.27078275 | 0.001107 | 0.004106 | -1.60906342 |
| EP400 | -0.088010324 | 3.216503372 | -3.27064911 | 0.001107 | 0.004106 | -1.60949206 |
| ASGR2 | -0.115554556 | 1.317890118 | -3.27031877 | 0.001109 | 0.00411 | -1.61055151 |
| APEH | 0.097620284 | 4.130906416 | 3.270195932 | 0.001109 | 0.00411 | -1.61094545 |
| PKHD1L1 | 0.259176796 | 2.311029797 | 3.266987179 | 0.001122 | 0.004155 | -1.62123053 |
| GSPT2 | -0.151010559 | 2.475792609 | -3.26673118 | 0.001123 | 0.004157 | -1.62205065 |
| C1RL | -0.117092462 | 3.772596529 | -3.26662428 | 0.001123 | 0.004158 | -1.62239311 |
| ASPHD1 | 0.196160565 | 2.853638692 | 3.265850793 | 0.001126 | 0.004167 | -1.62487064 |
| SLC33A1 | 0.081565019 | 3.340093604 | 3.265796163 | 0.001126 | 0.004167 | -1.6250456 |
| UFC1 | -0.098406462 | 4.644108306 | -3.26523988 | 0.001129 | 0.004174 | -1.62682702 |
| KIRREL3 | -0.159994558 | 1.356265111 | -3.26463245 | 0.001131 | 0.004181 | -1.62877188 |
| DUS4L | 0.079444242 | 2.535683135 | 3.264468286 | 0.001132 | 0.004182 | -1.62929745 |
| LAT | -0.089697751 | 1.74117647 | -3.26373317 | 0.001135 | 0.004191 | -1.63165057 |
| CD244 | -0.116391857 | 1.274344104 | -3.26365095 | 0.001135 | 0.004191 | -1.63191372 |
| UGP2 | 0.069805195 | 3.886132717 | 3.263025653 | 0.001137 | 0.004199 | -1.63391484 |
| ZNF641 | -0.081674576 | 3.011159669 | -3.26295428 | 0.001138 | 0.004199 | -1.63414323 |
| POLK | -0.075994572 | 2.576561363 | -3.26246922 | 0.00114 | 0.004204 | -1.63569526 |
| SRD5A1 | 0.106320415 | 2.86570993 | 3.26102983 | 0.001145 | 0.004223 | -1.6402995 |
| ETF1 | 0.077943806 | 4.253780564 | 3.261026583 | 0.001145 | 0.004223 | -1.64030989 |
| UGCG | 0.109970457 | 3.630356746 | 3.260933115 | 0.001146 | 0.004223 | -1.6406088 |
| EMP2 | 0.136238223 | 4.012497903 | 3.260671583 | 0.001147 | 0.004225 | -1.64144513 |
| GPKOW | 0.105761908 | 3.539519914 | 3.258839817 | 0.001154 | 0.004248 | -1.64730097 |
| TMEM147 | 0.115392104 | 4.652695447 | 3.258819847 | 0.001154 | 0.004248 | -1.6473648 |
| ZDHHC21 | 0.08827824 | 2.824347054 | 3.258818693 | 0.001154 | 0.004248 | -1.64736848 |
| HSD17B12 | 0.094212083 | 3.584934166 | 3.258521674 | 0.001155 | 0.004251 | -1.64831769 |
| TNNI1 | -0.176506355 | 1.731332603 | -3.25793698 | 0.001158 | 0.004259 | -1.65018601 |
| MMP10 | 0.268591079 | 1.517683406 | 3.256906221 | 0.001162 | 0.004272 | -1.65347885 |
| SLC12A2 | 0.10127466 | 2.904064603 | 3.256855733 | 0.001162 | 0.004272 | -1.65364011 |
| CHD2 | -0.077406515 | 3.715683475 | -3.25644101 | 0.001164 | 0.004276 | -1.65496467 |
| CREB5 | -0.103376849 | 2.296405414 | -3.25641558 | 0.001164 | 0.004276 | -1.65504588 |
| USP20 | -0.089999844 | 3.17885545 | -3.25623221 | 0.001165 | 0.004277 | -1.65563148 |
| TRAF3IP2 | -0.100295588 | 2.930988598 | -3.25614388 | 0.001165 | 0.004277 | -1.65591357 |
| FSCN1 | -0.163871629 | 4.302712508 | -3.25539747 | 0.001168 | 0.004287 | -1.65829684 |
| OBP2A | 0.322421009 | 2.342323689 | 3.254432421 | 0.001172 | 0.0043 | -1.66137745 |
| BASP1 | -0.190713606 | 3.526704944 | -3.2539828 | 0.001174 | 0.004305 | -1.66281241 |
| TWISTNB | 0.08715939 | 3.035029175 | 3.252961919 | 0.001178 | 0.004319 | -1.66606983 |
| OSGIN2 | 0.095624548 | 2.997079336 | 3.252710715 | 0.001179 | 0.004321 | -1.66687121 |
| ZNF236 | -0.071745966 | 2.665984641 | -3.25262912 | 0.001179 | 0.004321 | -1.66713152 |
| GAS2 | -0.144954851 | 1.250210227 | -3.25204209 | 0.001182 | 0.004328 | -1.66900394 |
| DMXL2 | -0.084199759 | 2.901121345 | -3.25168543 | 0.001183 | 0.004332 | -1.67014144 |
| TTLL12 | 0.10572452 | 3.770546548 | 3.251287781 | 0.001185 | 0.004337 | -1.67140948 |
| BAG5 | 0.08327777 | 3.739581642 | 3.249983588 | 0.00119 | 0.004354 | -1.67556733 |
| VAMP3 | 0.087680933 | 4.367075759 | 3.249974122 | 0.00119 | 0.004354 | -1.6755975 |
| MLYCD | -0.066745009 | 2.325670325 | -3.2493654 | 0.001193 | 0.004362 | -1.67753758 |
| TP63 | -0.131017945 | 1.414308027 | -3.24895062 | 0.001195 | 0.004366 | -1.67885935 |
| FOXC1 | 0.223053925 | 2.806063408 | 3.248863845 | 0.001195 | 0.004366 | -1.67913584 |
| PER2 | -0.096616198 | 2.8880225 | -3.24604359 | 0.001207 | 0.004408 | -1.68811835 |
| MRPL32 | 0.075858328 | 3.777036911 | 3.244629755 | 0.001213 | 0.004428 | -1.69261855 |
| APBB1IP | -0.138505304 | 2.586770861 | -3.24372598 | 0.001216 | 0.00444 | -1.69549423 |
| THG1L | -0.096082091 | 2.846824853 | -3.24367792 | 0.001217 | 0.00444 | -1.69564713 |
| STARD7 | 0.080465486 | 4.831192745 | 3.243126661 | 0.001219 | 0.004447 | -1.69740075 |
| IKZF1 | -0.101192938 | 2.235687006 | -3.2428084 | 0.00122 | 0.00445 | -1.69841303 |
| ZZEF1 | -0.071267444 | 3.02971375 | -3.24247123 | 0.001222 | 0.004454 | -1.69948538 |
| TIPARP | 0.110400962 | 3.274757712 | 3.242073829 | 0.001223 | 0.004459 | -1.70074911 |
| DCTN5 | 0.076544824 | 3.307000324 | 3.24179717 | 0.001225 | 0.004462 | -1.7016288 |
| CCDC43 | 0.085942304 | 3.267749828 | 3.241270806 | 0.001227 | 0.004468 | -1.70330228 |
| SIRPA | -0.130607743 | 3.436536904 | -3.24109271 | 0.001228 | 0.00447 | -1.70386844 |
| MATN1 | -0.114833364 | 1.245551997 | -3.24048088 | 0.00123 | 0.004478 | -1.70581321 |
| EPB41L3 | -0.11809 | 2.399207092 | -3.23864498 | 0.001238 | 0.004504 | -1.71164661 |
| CARHSP1 | 0.101234275 | 4.027158972 | 3.238605576 | 0.001238 | 0.004504 | -1.71177179 |
| TGM1 | 0.216935248 | 2.978832193 | 3.237329157 | 0.001244 | 0.004523 | -1.71582555 |
| SLC2A3 | 0.172793525 | 3.213901842 | 3.236793976 | 0.001246 | 0.004529 | -1.71752475 |
| VARS2 | -0.096009508 | 3.190079152 | -3.23625801 | 0.001248 | 0.004536 | -1.71922619 |
| BCCIP | 0.085013819 | 3.798063978 | 3.235949512 | 0.00125 | 0.00454 | -1.72020538 |
| AGT | -0.189183725 | 1.589748856 | -3.23450361 | 0.001256 | 0.004561 | -1.72479359 |
| RGS2 | -0.195624643 | 3.16578033 | -3.23429817 | 0.001257 | 0.004563 | -1.72544533 |
| TLCD1 | 0.14911452 | 2.925418687 | 3.234212926 | 0.001257 | 0.004563 | -1.72571575 |
| ERCC5 | -0.078540328 | 2.813505919 | -3.23322575 | 0.001262 | 0.004577 | -1.72884686 |
| TMEM208 | 0.092781221 | 3.645029519 | 3.232664001 | 0.001264 | 0.004584 | -1.73062821 |
| GPT | 0.184618524 | 2.588077197 | 3.232560526 | 0.001265 | 0.004584 | -1.7309563 |
| GLT1D1 | -0.138784658 | 1.34715276 | -3.23197208 | 0.001267 | 0.004592 | -1.7328219 |
| CBFA2T3 | -0.120757797 | 1.703678377 | -3.23143623 | 0.001269 | 0.004599 | -1.73452047 |
| GNL2 | 0.096198788 | 4.015401645 | 3.230438952 | 0.001274 | 0.004614 | -1.73768098 |
| PRSS8 | 0.148177271 | 4.59641711 | 3.230151889 | 0.001275 | 0.004617 | -1.73859054 |
| TMEM50A | 0.084362721 | 4.490050182 | 3.22913047 | 0.00128 | 0.004632 | -1.74182627 |
| NPHP1 | -0.089475191 | 2.428339197 | -3.2286258 | 0.001282 | 0.004636 | -1.74342464 |
| PBX3 | -0.113990158 | 2.850655415 | -3.22857696 | 0.001282 | 0.004636 | -1.7435793 |
| C12orf57 | -0.129038562 | 4.621087029 | -3.22855769 | 0.001282 | 0.004636 | -1.74364034 |
| TRPS1 | 0.15820839 | 3.494409655 | 3.228170317 | 0.001284 | 0.004641 | -1.74486701 |
| SLC16A6 | 0.111299632 | 1.823340133 | 3.227670588 | 0.001286 | 0.004646 | -1.74644926 |
| TMCO1 | 0.093067364 | 4.130086815 | 3.227621899 | 0.001286 | 0.004646 | -1.74660341 |
| AKR1C1 | -0.146030541 | 1.943636433 | -3.22759297 | 0.001286 | 0.004646 | -1.74669501 |
| IFI6 | 0.253307608 | 6.191475819 | 3.226330437 | 0.001292 | 0.004664 | -1.75069129 |
| F7 | -0.113635561 | 1.218637879 | -3.22614697 | 0.001293 | 0.004666 | -1.7512719 |
| UGT8 | 0.147448472 | 2.615100075 | 3.226022728 | 0.001293 | 0.004666 | -1.75166505 |
| HSDL1 | -0.086741981 | 2.939793658 | -3.22547527 | 0.001296 | 0.004674 | -1.75339729 |
| LRRC14 | 0.110447969 | 3.401812116 | 3.223782435 | 0.001304 | 0.0047 | -1.75875182 |
| TP53I13 | -0.095820588 | 3.254427918 | -3.22320283 | 0.001306 | 0.004708 | -1.76058452 |
| NPY | -0.250149968 | 1.480512578 | -3.22050101 | 0.001318 | 0.00475 | -1.7691233 |
| MDM1 | -0.087630009 | 2.703999193 | -3.22044096 | 0.001319 | 0.00475 | -1.769313 |
| ESD | -0.096845181 | 3.89451333 | -3.21936861 | 0.001324 | 0.004764 | -1.77270003 |
| DPM3 | 0.133625286 | 4.399043356 | 3.219354958 | 0.001324 | 0.004764 | -1.77274315 |
| LIG1 | 0.102144675 | 3.462675786 | 3.21894597 | 0.001326 | 0.00477 | -1.77403465 |
| ASRGL1 | 0.21415514 | 3.956645713 | 3.218306082 | 0.001328 | 0.004777 | -1.77605496 |
| OSGEPL1 | -0.088727809 | 2.81015611 | -3.21827452 | 0.001329 | 0.004777 | -1.7761546 |
| FEZ1 | -0.097403422 | 2.026357178 | -3.21697359 | 0.001335 | 0.004797 | -1.78026073 |
| CCDC6 | 0.128694186 | 4.662065675 | 3.216822296 | 0.001335 | 0.004798 | -1.78073817 |
| DACT1 | -0.138956263 | 2.220052308 | -3.21651702 | 0.001337 | 0.004802 | -1.78170145 |
| FZD7 | -0.150848714 | 3.464438729 | -3.21337996 | 0.001351 | 0.004853 | -1.79159492 |
| PPIB | 0.10911358 | 5.458458366 | 3.212888029 | 0.001354 | 0.004859 | -1.7931455 |
| MC4R | -0.153315922 | 1.25360938 | -3.21126304 | 0.001361 | 0.004885 | -1.7982658 |
| EDN3 | -0.193600544 | 1.107259877 | -3.2106369 | 0.001364 | 0.004894 | -1.80023811 |
| SPTAN1 | -0.099524529 | 4.485378066 | -3.21010796 | 0.001367 | 0.004901 | -1.80190389 |
| ZNF540 | -0.093834423 | 1.608086907 | -3.20993845 | 0.001367 | 0.004901 | -1.80243768 |
| CNTNAP1 | -0.089056455 | 2.353640347 | -3.20992221 | 0.001367 | 0.004901 | -1.80248884 |
| ITIH4 | -0.095978451 | 1.508375977 | -3.20919103 | 0.001371 | 0.004912 | -1.80479101 |
| MCM5 | 0.105695803 | 3.701937456 | 3.209048584 | 0.001372 | 0.004913 | -1.80523944 |
| MDH1 | 0.073841159 | 4.456201309 | 3.20807687 | 0.001376 | 0.004928 | -1.80829801 |
| VPS35 | 0.087955061 | 3.960096706 | 3.20771067 | 0.001378 | 0.004931 | -1.80945043 |
| DMXL1 | -0.075049162 | 2.851489744 | -3.20766072 | 0.001378 | 0.004931 | -1.80960762 |
| CLCN6 | -0.090243474 | 2.984297531 | -3.20760686 | 0.001378 | 0.004931 | -1.80977709 |
| RP1 | -0.120511176 | 1.647152425 | -3.20722889 | 0.00138 | 0.004936 | -1.81096638 |
| NGFR | -0.168739857 | 1.887285792 | -3.20514097 | 0.00139 | 0.00497 | -1.81753353 |
| NBN | 0.094971539 | 3.637408564 | 3.204279264 | 0.001394 | 0.004983 | -1.82024266 |
| PNPLA3 | 0.120732131 | 1.872285346 | 3.203849012 | 0.001396 | 0.004989 | -1.82159506 |
| ITGB8 | 0.161620858 | 3.872233507 | 3.203750642 | 0.001397 | 0.004989 | -1.82190423 |
| ZFAND2A | 0.104955877 | 2.960659108 | 3.203167162 | 0.0014 | 0.004997 | -1.82373794 |
| RBM41 | 0.076433373 | 2.663390577 | 3.202721884 | 0.001402 | 0.005003 | -1.82513709 |
| CRCP | 0.07255472 | 3.585377805 | 3.202456422 | 0.001403 | 0.005006 | -1.82597113 |
| S1PR1 | -0.11898418 | 2.413813459 | -3.20229164 | 0.001404 | 0.005007 | -1.82648883 |
| LIG3 | -0.079863803 | 2.852491152 | -3.20148242 | 0.001408 | 0.00502 | -1.82903073 |
| ICA1L | -0.095442698 | 2.143064355 | -3.20086745 | 0.001411 | 0.005029 | -1.83096205 |
| DSN1 | 0.101154033 | 3.319514187 | 3.199914906 | 0.001415 | 0.005043 | -1.83395278 |
| TMEM140 | 0.123578069 | 3.144916778 | 3.199788057 | 0.001416 | 0.005044 | -1.83435099 |
| SBNO2 | 0.108368477 | 3.939484072 | 3.198520987 | 0.001422 | 0.005064 | -1.83832775 |
| DPEP1 | -0.168302754 | 1.545305057 | -3.19774882 | 0.001426 | 0.005076 | -1.84075049 |
| TMEM9 | 0.090229571 | 4.437766057 | 3.197270955 | 0.001428 | 0.005083 | -1.84224953 |
| LYST | -0.064727108 | 2.492067209 | -3.19662269 | 0.001431 | 0.005093 | -1.84428278 |
| TYW1 | -0.072882236 | 3.295651949 | -3.19570183 | 0.001436 | 0.005106 | -1.84717029 |
| SLC19A3 | 0.147911879 | 2.034752941 | 3.195653884 | 0.001436 | 0.005106 | -1.8473206 |
| C4orf19 | 0.176461118 | 2.146492515 | 3.195346438 | 0.001438 | 0.005109 | -1.84828445 |
| CALML5 | 0.275868166 | 1.597894166 | 3.195283944 | 0.001438 | 0.005109 | -1.84848036 |
| FKBP5 | 0.161520171 | 3.202118763 | 3.195123405 | 0.001439 | 0.005111 | -1.84898362 |
| PDPK1 | -0.073465651 | 2.839318826 | -3.19469229 | 0.001441 | 0.005117 | -1.85033493 |
| PIK3R5 | -0.092914581 | 2.217977641 | -3.19444342 | 0.001442 | 0.005119 | -1.85111492 |
| PEF1 | 0.085201532 | 4.324371564 | 3.194286311 | 0.001443 | 0.00512 | -1.85160729 |
| GLIPR1 | -0.121961382 | 2.691836773 | -3.19304945 | 0.001449 | 0.005141 | -1.85548272 |
| ZNF532 | -0.095107393 | 3.620051936 | -3.19157332 | 0.001456 | 0.005165 | -1.8601059 |
| DNAJC27 | -0.068436834 | 2.225642903 | -3.1911805 | 0.001458 | 0.005168 | -1.86133584 |
| CHGB | -0.1697492 | 1.175594763 | -3.19103605 | 0.001459 | 0.005168 | -1.86178812 |
| NAP1L5 | -0.129725218 | 1.970253493 | -3.19100861 | 0.001459 | 0.005168 | -1.86187401 |
| EPB41L2 | -0.13182273 | 3.582294704 | -3.19100644 | 0.001459 | 0.005168 | -1.86188081 |
| STK4 | -0.070677777 | 3.249283826 | -3.19030254 | 0.001463 | 0.005179 | -1.86408428 |
| ALMS1 | -0.08277323 | 3.108522145 | -3.18998038 | 0.001464 | 0.005183 | -1.86509259 |
| IL15RA | 0.1467019 | 2.795312775 | 3.188249318 | 0.001473 | 0.005212 | -1.8705089 |
| EHMT2 | -0.098540815 | 4.340578274 | -3.18785375 | 0.001475 | 0.005218 | -1.87174619 |
| NICN1 | -0.091800263 | 3.109394241 | -3.18718252 | 0.001479 | 0.005228 | -1.87384537 |
| EPHX2 | -0.126824673 | 2.840254732 | -3.18703818 | 0.001479 | 0.005229 | -1.87429669 |
| PRPS1 | 0.093301583 | 3.357051545 | 3.186819896 | 0.00148 | 0.005229 | -1.87497923 |
| CTNNA1 | 0.071837958 | 4.826221902 | 3.186787912 | 0.001481 | 0.005229 | -1.87507923 |
| TSPAN13 | 0.151885684 | 3.585096043 | 3.18677001 | 0.001481 | 0.005229 | -1.87513521 |
| PSMA2 | 0.101194103 | 3.530514037 | 3.186333826 | 0.001483 | 0.005235 | -1.87649891 |
| NCOA1 | -0.077337214 | 3.568963509 | -3.18591787 | 0.001485 | 0.005241 | -1.87779918 |
| UXT | 0.104057916 | 4.722284232 | 3.184355848 | 0.001493 | 0.005267 | -1.88268061 |
| SMTN | -0.10478473 | 3.195167792 | -3.18369501 | 0.001496 | 0.005277 | -1.88474505 |
| ENPP6 | -0.119460735 | 0.922152309 | -3.18349654 | 0.001497 | 0.005278 | -1.885365 |
| CACNA2D3 | 0.155998575 | 2.133299789 | 3.183482341 | 0.001497 | 0.005278 | -1.88540935 |
| ZNF701 | 0.078784197 | 2.541777672 | 3.18316028 | 0.001499 | 0.005282 | -1.88641525 |
| ALCAM | 0.174861893 | 3.69547734 | 3.183033659 | 0.0015 | 0.005283 | -1.8868107 |
| RPRD2 | -0.094390473 | 3.99113487 | -3.18270566 | 0.001501 | 0.005287 | -1.88783501 |
| FARSA | 0.109615243 | 4.308923911 | 3.181995233 | 0.001505 | 0.005298 | -1.89005322 |
| ILK | -0.086832115 | 3.35648952 | -3.18193303 | 0.001505 | 0.005298 | -1.89024742 |
| ELL3 | 0.131472932 | 1.347568907 | 3.181809159 | 0.001506 | 0.005298 | -1.89063413 |
| GGT7 | -0.112074238 | 3.483891677 | -3.18129554 | 0.001509 | 0.005303 | -1.89223747 |
| PHRF1 | -0.090246981 | 3.63755553 | -3.18126711 | 0.001509 | 0.005303 | -1.89232619 |
| PCDHB4 | -0.12053175 | 1.72958271 | -3.1812581 | 0.001509 | 0.005303 | -1.89235432 |
| ANXA1 | 0.171299305 | 4.761426503 | 3.180607203 | 0.001512 | 0.005313 | -1.89438578 |
| COL6A2 | -0.195703628 | 4.990839353 | -3.17985155 | 0.001516 | 0.005325 | -1.89674367 |
| ST8SIA5 | 0.133628606 | 1.54196416 | 3.178880814 | 0.001521 | 0.005341 | -1.89977189 |
| ABCD4 | -0.077450385 | 3.276506109 | -3.178735 | 0.001522 | 0.005342 | -1.90022668 |
| ARID4B | -0.07854361 | 3.375388603 | -3.17645357 | 0.001534 | 0.005382 | -1.90733973 |
| RBM5 | -0.09074957 | 3.760796451 | -3.17633799 | 0.001534 | 0.005383 | -1.90769996 |
| CAMK2D | -0.099649414 | 2.759417953 | -3.17546939 | 0.001539 | 0.005395 | -1.91040666 |
| NDUFS7 | 0.086144971 | 3.294632578 | 3.175411496 | 0.001539 | 0.005395 | -1.91058705 |
| STAT4 | -0.108812504 | 1.874869357 | -3.17541056 | 0.001539 | 0.005395 | -1.91058997 |
| CYP2C8 | -0.144714083 | 1.408964289 | -3.17500543 | 0.001541 | 0.0054 | -1.91185216 |
| VANGL1 | 0.101460205 | 3.569559843 | 3.174809728 | 0.001542 | 0.005402 | -1.91246181 |
| CHRNE | -0.112296226 | 1.786907158 | -3.17418782 | 0.001546 | 0.005412 | -1.91439895 |
| RUSC2 | -0.097047307 | 3.139902274 | -3.17189833 | 0.001558 | 0.005453 | -1.92152709 |
| ANKRD53 | -0.125348622 | 1.750431372 | -3.16951387 | 0.001571 | 0.005496 | -1.92894557 |
| HMGCL | 0.086789789 | 3.382927568 | 3.168632986 | 0.001575 | 0.00551 | -1.93168475 |
| MTX1 | 0.074693532 | 3.530989022 | 3.168381561 | 0.001577 | 0.005513 | -1.93246645 |
| ANXA7 | 0.079716504 | 4.437452111 | 3.167667882 | 0.00158 | 0.005525 | -1.93468498 |
| CREB3L2 | -0.095170784 | 3.69777067 | -3.16667125 | 0.001586 | 0.005542 | -1.93778228 |
| OXGR1 | 0.193533184 | 1.97731223 | 3.165762514 | 0.001591 | 0.005557 | -1.9406056 |
| CUX2 | -0.162312186 | 1.282447114 | -3.16502969 | 0.001595 | 0.00557 | -1.94288178 |
| ZNF485 | -0.086916344 | 2.336805289 | -3.16488381 | 0.001595 | 0.005571 | -1.94333484 |
| FOSB | -0.287628115 | 3.490114996 | -3.16461353 | 0.001597 | 0.005574 | -1.94417417 |
| NUP62 | 0.078676315 | 4.001250553 | 3.164513621 | 0.001597 | 0.005574 | -1.94448442 |
| TNIP1 | 0.087061435 | 4.285737991 | 3.163757285 | 0.001602 | 0.005587 | -1.94683273 |
| PHPT1 | 0.107170975 | 4.480408476 | 3.163608442 | 0.001602 | 0.005588 | -1.9472948 |
| TCF21 | -0.169551857 | 1.739703434 | -3.1608527 | 0.001618 | 0.005639 | -1.9558459 |
| AKT1 | 0.103582193 | 4.211841281 | 3.160707522 | 0.001618 | 0.00564 | -1.95629619 |
| DNAH1 | -0.081092298 | 2.488710859 | -3.16051041 | 0.001619 | 0.005642 | -1.95690751 |
| GBP4 | 0.182034377 | 3.156923084 | 3.160203319 | 0.001621 | 0.005646 | -1.95785988 |
| CDH22 | -0.193970622 | 1.715537338 | -3.15767655 | 0.001635 | 0.005693 | -1.96569247 |
| MYOM3 | -0.135543826 | 1.680763232 | -3.15668923 | 0.001641 | 0.00571 | -1.96875134 |
| PAK3 | -0.112162727 | 1.663202366 | -3.15627425 | 0.001643 | 0.005716 | -1.97003674 |
| SEMA5A | -0.120763341 | 2.547585861 | -3.15481382 | 0.001651 | 0.005743 | -1.97455907 |
| CLASP2 | -0.074153994 | 2.923030053 | -3.15425776 | 0.001654 | 0.005752 | -1.97628041 |
| MEIS3 | -0.11984737 | 2.328994096 | -3.15417156 | 0.001655 | 0.005752 | -1.97654721 |
| LRRC8E | 0.095524981 | 1.870659419 | 3.153634883 | 0.001658 | 0.00576 | -1.97820823 |
| DDRGK1 | 0.102726645 | 4.431008481 | 3.153016251 | 0.001661 | 0.005771 | -1.98012255 |
| NFKBIA | 0.115279203 | 4.794582135 | 3.152540399 | 0.001664 | 0.005778 | -1.98159479 |
| HYLS1 | 0.112720276 | 2.714760042 | 3.152098385 | 0.001666 | 0.005785 | -1.98296215 |
| MYOD1 | 0.163742366 | 1.306541661 | 3.151373754 | 0.00167 | 0.005797 | -1.98520337 |
| BSG | 0.107695783 | 5.455624989 | 3.150926385 | 0.001673 | 0.005804 | -1.98658679 |
| TSC22D1 | -0.109206912 | 4.620833025 | -3.15036104 | 0.001676 | 0.005814 | -1.98833475 |
| FBXO21 | -0.145806455 | 4.242743281 | -3.14921114 | 0.001683 | 0.005835 | -1.99188912 |
| TMEM59L | -0.14966027 | 1.463057661 | -3.14857727 | 0.001686 | 0.005845 | -1.99384788 |
| COQ2 | 0.086770376 | 2.828599855 | 3.146012887 | 0.001701 | 0.005895 | -2.00176833 |
| DYNLL1 | 0.079802273 | 5.230869219 | 3.145377388 | 0.001705 | 0.005905 | -2.00373017 |
| ZNF75A | -0.074092859 | 2.91387901 | -3.14484931 | 0.001708 | 0.005914 | -2.00536009 |
| ARPC4 | 0.080379769 | 4.28991847 | 3.143948062 | 0.001713 | 0.005929 | -2.00814121 |
| KCNK1 | 0.129500904 | 2.944108369 | 3.143943384 | 0.001713 | 0.005929 | -2.00815564 |
| C1QTNF1 | -0.149267888 | 2.980247185 | -3.14340952 | 0.001716 | 0.005937 | -2.00980268 |
| FCER1G | 0.1650473 | 4.377817008 | 3.141447347 | 0.001728 | 0.005975 | -2.01585392 |
| SPRY4 | 0.14697129 | 3.38419407 | 3.140740805 | 0.001732 | 0.005988 | -2.01803195 |
| CLN3 | 0.094114386 | 3.049775592 | 3.140224004 | 0.001735 | 0.005996 | -2.01962477 |
| ANKFN1 | -0.133369655 | 1.337539908 | -3.13913629 | 0.001741 | 0.006016 | -2.02297632 |
| FHL5 | -0.118068836 | 1.160247997 | -3.13840097 | 0.001745 | 0.006029 | -2.02524142 |
| MDGA1 | -0.093062201 | 2.052666406 | -3.13833808 | 0.001746 | 0.006029 | -2.02543513 |
| ZNF613 | 0.078045691 | 2.208604175 | 3.137431628 | 0.001751 | 0.006045 | -2.02822661 |
| VPS11 | -0.077945233 | 3.4709891 | -3.13665145 | 0.001756 | 0.00606 | -2.03062859 |
| CDK5RAP3 | -0.105020206 | 4.201820155 | -3.135855 | 0.001761 | 0.006073 | -2.03308006 |
| ZNF585B | -0.08854306 | 2.729653165 | -3.13579488 | 0.001761 | 0.006073 | -2.03326508 |
| CSNK1A1 | 0.090162614 | 3.896377235 | 3.135609283 | 0.001762 | 0.006075 | -2.03383625 |
| MTSS1 | -0.119256962 | 2.809455429 | -3.13526458 | 0.001764 | 0.00608 | -2.03489699 |
| PARK7 | 0.087751625 | 4.979019398 | 3.134040821 | 0.001771 | 0.006104 | -2.03866181 |
| CHMP6 | -0.093662198 | 3.417254999 | -3.13304723 | 0.001777 | 0.006122 | -2.0417175 |
| BRCA2 | 0.084678673 | 2.27664838 | 3.132776091 | 0.001779 | 0.006125 | -2.04255118 |
| GBA2 | 0.096170393 | 3.859964239 | 3.132725977 | 0.001779 | 0.006125 | -2.04270527 |
| PRR11 | 0.114396541 | 3.087599223 | 3.131974969 | 0.001784 | 0.006139 | -2.04501404 |
| CYTH4 | -0.111564873 | 2.661801279 | -3.13042739 | 0.001793 | 0.006168 | -2.04976996 |
| CTDSP2 | -0.087163479 | 4.966790864 | -3.13039698 | 0.001793 | 0.006168 | -2.04986338 |
| PTCD2 | -0.076427116 | 2.215905818 | -3.12999662 | 0.001796 | 0.006174 | -2.05109335 |
| SLC26A1 | -0.129622978 | 1.643488162 | -3.12920023 | 0.001801 | 0.006184 | -2.05353954 |
| GOT2 | 0.083654905 | 4.207811124 | 3.129169049 | 0.001801 | 0.006184 | -2.0536353 |
| NAB2 | -0.092608467 | 4.020043814 | -3.12915915 | 0.001801 | 0.006184 | -2.05366569 |
| PITX1 | 0.256642103 | 2.432469458 | 3.129140787 | 0.001801 | 0.006184 | -2.05372209 |
| SGCA | -0.108691967 | 1.062675241 | -3.12866783 | 0.001804 | 0.006192 | -2.05517449 |
| PGAM5 | 0.087484207 | 3.465147378 | 3.12845592 | 0.001805 | 0.006195 | -2.05582519 |
| FZD9 | 0.117431754 | 1.247370047 | 3.128004573 | 0.001808 | 0.006202 | -2.05721095 |
| IDUA | -0.124227072 | 3.43324809 | -3.125824 | 0.001821 | 0.006246 | -2.06390311 |
| ANK3 | -0.096336328 | 2.734348737 | -3.12340666 | 0.001836 | 0.006295 | -2.0713166 |
| SPOCK2 | -0.222190055 | 4.395761446 | -3.12334061 | 0.001836 | 0.006295 | -2.07151906 |
| ZMYND19 | 0.09177914 | 3.43095968 | 3.123069927 | 0.001838 | 0.006298 | -2.07234883 |
| WDR7 | -0.061178568 | 2.445491 | -3.12268671 | 0.00184 | 0.006304 | -2.07352344 |
| TAS2R14 | -0.088243693 | 1.877476339 | -3.12262865 | 0.001841 | 0.006304 | -2.07370136 |
| NFATC3 | -0.080778665 | 2.885593771 | -3.12253021 | 0.001841 | 0.006304 | -2.07400309 |
| CLIP2 | -0.125973545 | 3.056635497 | -3.12033837 | 0.001855 | 0.006349 | -2.08071825 |
| PANK3 | 0.078633364 | 3.36791689 | 3.119866448 | 0.001858 | 0.006355 | -2.0821635 |
| USP47 | -0.072073984 | 3.224995546 | -3.11984733 | 0.001858 | 0.006355 | -2.08222204 |
| RNF111 | -0.074559163 | 3.256299619 | -3.11815148 | 0.001869 | 0.00639 | -2.08741366 |
| TRPC1 | -0.095621414 | 2.233070019 | -3.1172055 | 0.001875 | 0.006408 | -2.09030846 |
| CALD1 | -0.144291261 | 4.366014117 | -3.11670361 | 0.001878 | 0.006417 | -2.09184395 |
| SLC2A12 | -0.140161924 | 2.275549319 | -3.11547293 | 0.001886 | 0.006441 | -2.09560806 |
| EIF3H | 0.102175337 | 5.063345923 | 3.115175572 | 0.001888 | 0.006446 | -2.09651734 |
| ABHD12 | 0.08878048 | 4.170561204 | 3.115073813 | 0.001888 | 0.006446 | -2.09682848 |
| HERC5 | 0.166053024 | 2.966564879 | 3.114693623 | 0.001891 | 0.006452 | -2.09799088 |
| CHD1 | -0.078385545 | 3.124325655 | -3.11453787 | 0.001892 | 0.006454 | -2.09846705 |
| IFIT3 | 0.195569175 | 4.218556628 | 3.113882723 | 0.001896 | 0.006466 | -2.10046966 |
| ZFPL1 | 0.075342062 | 3.205513923 | 3.112975676 | 0.001902 | 0.006482 | -2.1032416 |
| XYLT1 | -0.093252917 | 2.102368693 | -3.112934 | 0.001902 | 0.006482 | -2.10336894 |
| MYOZ1 | -0.18194673 | 1.927038412 | -3.11284705 | 0.001902 | 0.006482 | -2.10363461 |
| RABEP1 | -0.074457813 | 3.253573674 | -3.11261658 | 0.001904 | 0.006485 | -2.10433878 |
| LY86 | -0.15531772 | 2.614558349 | -3.11236145 | 0.001905 | 0.006489 | -2.10511822 |
| ATP6AP1 | 0.095468369 | 4.638602084 | 3.111841194 | 0.001909 | 0.006498 | -2.10670747 |
| CORO1C | 0.090209904 | 3.793666497 | 3.111577223 | 0.00191 | 0.006502 | -2.10751373 |
| PPP2R5B | 0.095768154 | 2.908459623 | 3.110569288 | 0.001917 | 0.006522 | -2.1105917 |
| IDH1 | 0.103394922 | 4.202244425 | 3.110347243 | 0.001918 | 0.006525 | -2.11126963 |
| ALG5 | 0.093072572 | 3.362169421 | 3.109545715 | 0.001924 | 0.006541 | -2.11371642 |
| RPL10A | -0.1017078 | 6.709562352 | -3.10908042 | 0.001927 | 0.006549 | -2.11513653 |
| ANKRD26 | -0.072538738 | 2.612277701 | -3.1089944 | 0.001927 | 0.006549 | -2.11539904 |
| EOMES | -0.119617127 | 1.633078549 | -3.10833934 | 0.001931 | 0.006561 | -2.11739789 |
| ANKS6 | -0.081094457 | 3.034656949 | -3.1080281 | 0.001933 | 0.006566 | -2.11834748 |
| GRAMD1C | -0.127380981 | 2.572282846 | -3.10790348 | 0.001934 | 0.006567 | -2.11872765 |
| ZNF254 | -0.080255066 | 2.880844548 | -3.10737405 | 0.001938 | 0.006576 | -2.12034265 |
| THYN1 | -0.098933438 | 3.791227087 | -3.10715463 | 0.001939 | 0.006579 | -2.1210119 |
| TTC25 | -0.138989442 | 1.924389827 | -3.10626734 | 0.001945 | 0.006597 | -2.12371769 |
| PDE2A | -0.084789767 | 1.862052439 | -3.10616743 | 0.001945 | 0.006597 | -2.12402233 |
| ASXL1 | -0.081595736 | 3.978366002 | -3.10608006 | 0.001946 | 0.006597 | -2.12428873 |
| EIF5A | 0.107169004 | 5.296240953 | 3.105057039 | 0.001953 | 0.006617 | -2.12740733 |
| PCMTD1 | -0.090784463 | 3.673145888 | -3.10442215 | 0.001957 | 0.006629 | -2.12934225 |
| S100A4 | 0.212200391 | 4.98351982 | 3.103276348 | 0.001964 | 0.006653 | -2.13283324 |
| HERPUD2 | -0.0681477 | 3.52016542 | -3.10278201 | 0.001968 | 0.006662 | -2.13433898 |
| DYNLRB2 | -0.156131942 | 1.263290575 | -3.10178045 | 0.001974 | 0.006682 | -2.13738899 |
| OAS2 | 0.187550675 | 3.773830833 | 3.100875108 | 0.00198 | 0.0067 | -2.14014517 |
| FBXO34 | 0.085717996 | 3.819336762 | 3.100452264 | 0.001983 | 0.006708 | -2.14143218 |
| CDC26 | 0.089245177 | 3.149322053 | 3.09996871 | 0.001986 | 0.006717 | -2.14290376 |
| NELL1 | -0.169688976 | 1.209517001 | -3.09937343 | 0.00199 | 0.006728 | -2.14471504 |
| PHB | 0.090706768 | 4.417497045 | 3.098923677 | 0.001993 | 0.006736 | -2.14608331 |
| STX17 | 0.076926462 | 2.9374851 | 3.098658878 | 0.001995 | 0.00674 | -2.1468888 |
| CD63 | 0.091012488 | 5.835170247 | 3.095809372 | 0.002014 | 0.006802 | -2.15555243 |
| TMEM106A | -0.07767594 | 2.544063896 | -3.09531319 | 0.002017 | 0.00681 | -2.15706023 |
| SCYL1 | 0.069268968 | 4.12777079 | 3.095282002 | 0.002018 | 0.00681 | -2.15715499 |
| CLDN9 | -0.202520876 | 2.783883607 | -3.09462567 | 0.002022 | 0.006823 | -2.15914907 |
| NKAP | 0.078610221 | 2.772892971 | 3.093029242 | 0.002033 | 0.006857 | -2.16399759 |
| CALHM2 | -0.125615184 | 3.58931458 | -3.09246013 | 0.002037 | 0.006868 | -2.16572544 |
| MYO3B | -0.145875836 | 2.00449556 | -3.09214986 | 0.002039 | 0.006873 | -2.16666733 |
| RPL37A | -0.112917507 | 6.130893802 | -3.09197549 | 0.00204 | 0.006875 | -2.16719661 |
| LIN7C | 0.081891244 | 3.408758752 | 3.091550453 | 0.002043 | 0.006883 | -2.16848665 |
| DDX49 | 0.089652252 | 3.911677315 | 3.088355212 | 0.002065 | 0.006955 | -2.17817902 |
| WDR59 | -0.076954746 | 3.019814806 | -3.0879843 | 0.002067 | 0.006961 | -2.17930351 |
| DDAH2 | -0.119507798 | 4.728036389 | -3.08757128 | 0.00207 | 0.006969 | -2.18055549 |
| STK24 | 0.084318726 | 3.711175531 | 3.086550331 | 0.002077 | 0.00699 | -2.18364956 |
| MAOB | -0.189235597 | 2.535552672 | -3.08635309 | 0.002079 | 0.006993 | -2.18424719 |
| WWTR1 | 0.092080218 | 3.921038231 | 3.085965443 | 0.002081 | 0.007 | -2.18542166 |
| ISG20 | 0.131130138 | 2.781616399 | 3.085517153 | 0.002084 | 0.007008 | -2.18677967 |
| PPP2R5E | 0.072653838 | 3.355073584 | 3.085096753 | 0.002087 | 0.007014 | -2.18805303 |
| ACADS | 0.102292056 | 2.849722985 | 3.085095984 | 0.002087 | 0.007014 | -2.18805535 |
| METTL6 | 0.066899396 | 2.694212123 | 3.084899672 | 0.002089 | 0.007015 | -2.1886499 |
| SETD6 | -0.081556629 | 2.943449909 | -3.08485893 | 0.002089 | 0.007015 | -2.1887733 |
| NASP | 0.094736566 | 4.194030763 | 3.084568351 | 0.002091 | 0.007019 | -2.18965326 |
| FSTL3 | -0.130129321 | 2.99294319 | -3.08431804 | 0.002093 | 0.007023 | -2.19041122 |
| CDH5 | -0.098537489 | 2.584489588 | -3.08324937 | 0.0021 | 0.007045 | -2.19364654 |
| ARL15 | -0.084442424 | 2.433057356 | -3.08318827 | 0.002101 | 0.007045 | -2.19383148 |
| BEND6 | -0.104974695 | 1.682920817 | -3.0821386 | 0.002108 | 0.007067 | -2.19700815 |
| CYB5R4 | 0.079270701 | 2.384153592 | 3.081989671 | 0.002109 | 0.007067 | -2.19745878 |
| VWC2 | -0.129454196 | 0.954098056 | -3.08198001 | 0.002109 | 0.007067 | -2.19748803 |
| MSX2 | 0.158384774 | 1.414879283 | 3.081635787 | 0.002112 | 0.007073 | -2.19852947 |
| ITGAL | -0.126954063 | 2.433262746 | -3.08064489 | 0.002119 | 0.007095 | -2.2015268 |
| DOCK5 | -0.092282177 | 2.566511001 | -3.08046101 | 0.00212 | 0.007097 | -2.20208294 |
| AADAC | 0.223949001 | 2.146323087 | 3.080374622 | 0.00212 | 0.007097 | -2.20234418 |
| POLR3D | -0.082355978 | 3.167949508 | -3.07988141 | 0.002124 | 0.007106 | -2.20383559 |
| TBC1D13 | -0.083019456 | 3.354315436 | -3.07709146 | 0.002144 | 0.00717 | -2.21226773 |
| FMNL2 | -0.091056432 | 3.619114099 | -3.07647491 | 0.002148 | 0.007183 | -2.21413014 |
| PMPCA | 0.076503274 | 3.523835436 | 3.076387567 | 0.002149 | 0.007183 | -2.21439394 |
| CHRNA1 | 0.16735174 | 1.738051023 | 3.076141513 | 0.002151 | 0.007184 | -2.21513707 |
| ARHGAP9 | -0.114876149 | 2.408044015 | -3.07613613 | 0.002151 | 0.007184 | -2.21515332 |
| MTHFD1 | 0.082825743 | 3.638097305 | 3.075973238 | 0.002152 | 0.007186 | -2.21564526 |
| CENPB | -0.091442138 | 4.342479129 | -3.07466011 | 0.002161 | 0.007215 | -2.21960994 |
| ROS1 | 0.176914354 | 1.5546409 | 3.073880736 | 0.002167 | 0.007232 | -2.22196229 |
| IER5 | 0.105179166 | 3.313789924 | 3.073711425 | 0.002168 | 0.007234 | -2.22247324 |
| SF1 | -0.060434668 | 4.728759145 | -3.0735577 | 0.002169 | 0.007234 | -2.22293711 |
| C19orf53 | 0.11535617 | 5.354650293 | 3.073537922 | 0.002169 | 0.007234 | -2.22299681 |
| DAB1 | -0.10851261 | 1.56073752 | -3.07287639 | 0.002174 | 0.007247 | -2.2249928 |
| WASL | 0.074246387 | 3.959938437 | 3.072700944 | 0.002175 | 0.007249 | -2.2255221 |
| SOD3 | -0.182323025 | 3.200759684 | -3.07224003 | 0.002179 | 0.007258 | -2.22691245 |
| HSPH1 | 0.094392909 | 3.960466439 | 3.071914907 | 0.002181 | 0.007264 | -2.22789307 |
| PIK3R2 | -0.110409455 | 1.687693825 | -3.0717897 | 0.002182 | 0.007265 | -2.2282707 |
| BMP1 | -0.102569964 | 3.380016313 | -3.0712325 | 0.002186 | 0.007276 | -2.22995097 |
| USPL1 | -0.06927048 | 2.7872954 | -3.0706713 | 0.00219 | 0.007287 | -2.23164304 |
| C16orf54 | -0.132064104 | 1.582645655 | -3.06936229 | 0.002199 | 0.007317 | -2.2355886 |
| ABCC4 | 0.138987698 | 3.26745451 | 3.069268649 | 0.0022 | 0.007317 | -2.23587077 |
| COL4A3 | -0.148239778 | 1.442432897 | -3.06911336 | 0.002201 | 0.007319 | -2.23633873 |
| ZNF165 | 0.108069896 | 2.525110702 | 3.068951845 | 0.002202 | 0.00732 | -2.23682539 |
| ITGA4 | -0.102676801 | 2.266109212 | -3.06853617 | 0.002206 | 0.007328 | -2.23807779 |
| CSRP1 | -0.104075955 | 4.791905916 | -3.06758579 | 0.002212 | 0.007349 | -2.24094059 |
| EPB41 | -0.090312548 | 3.235765455 | -3.0661962 | 0.002223 | 0.007381 | -2.24512484 |
| TIGD7 | -0.076225518 | 2.288083055 | -3.06594145 | 0.002225 | 0.007385 | -2.24589173 |
| PPP1R13B | 0.098456494 | 3.56063054 | 3.065258747 | 0.00223 | 0.007399 | -2.24794659 |
| PA2G4 | 0.0751136 | 4.624970585 | 3.064667985 | 0.002234 | 0.007412 | -2.24972436 |
| EDF1 | 0.085430513 | 5.372800831 | 3.063013898 | 0.002246 | 0.00745 | -2.25470019 |
| RNF19B | 0.111358933 | 3.908920575 | 3.061642675 | 0.002256 | 0.007482 | -2.2588231 |
| RBBP9 | -0.090102704 | 3.105611081 | -3.0614205 | 0.002258 | 0.007485 | -2.25949096 |
| HRC | -0.117095316 | 1.286078691 | -3.06042447 | 0.002266 | 0.007507 | -2.2624844 |
| PSMC2 | 0.068100889 | 4.135400815 | 3.059540012 | 0.002272 | 0.007527 | -2.26514174 |
| IL5RA | -0.121557782 | 1.2398437 | -3.05747934 | 0.002288 | 0.007576 | -2.27133006 |
| ARNTL | -0.080215716 | 2.530913186 | -3.05711264 | 0.002291 | 0.007583 | -2.27243083 |
| UBE2Q1 | -0.058654444 | 4.209683753 | -3.0567947 | 0.002293 | 0.007586 | -2.27338515 |
| CNTFR | -0.225808452 | 2.161391717 | -3.05672573 | 0.002294 | 0.007586 | -2.27359217 |
| EIF2AK3 | -0.065433426 | 3.115068634 | -3.05669244 | 0.002294 | 0.007586 | -2.27369209 |
| ABCC9 | -0.108974024 | 2.194435435 | -3.05665968 | 0.002294 | 0.007586 | -2.2737904 |
| ZFAND1 | 0.088759668 | 3.723987978 | 3.055851528 | 0.0023 | 0.007603 | -2.27621557 |
| CHSY1 | -0.100975549 | 3.546871122 | -3.05578569 | 0.002301 | 0.007603 | -2.27641312 |
| HRAS | 0.097392798 | 3.510539798 | 3.055451966 | 0.002303 | 0.007609 | -2.27741438 |
| FLYWCH1 | -0.082803971 | 2.998952827 | -3.05495091 | 0.002307 | 0.00762 | -2.27891746 |
| ZNF362 | -0.093164822 | 3.892890332 | -3.05483868 | 0.002308 | 0.00762 | -2.27925413 |
| LAS1L | 0.071189607 | 3.481349887 | 3.054389035 | 0.002311 | 0.007629 | -2.28060274 |
| RANGAP1 | 0.082616044 | 4.063102077 | 3.053935993 | 0.002315 | 0.007638 | -2.28196135 |
| NDUFA7 | 0.124489268 | 2.997834311 | 3.053710212 | 0.002316 | 0.007642 | -2.28263836 |
| FHL3 | -0.097741918 | 3.568864181 | -3.05203264 | 0.002329 | 0.007682 | -2.28766709 |
| TSPAN9 | -0.0955548 | 3.511697239 | -3.05175442 | 0.002331 | 0.007687 | -2.28850081 |
| SLC25A32 | 0.083817316 | 3.438995206 | 3.051230247 | 0.002336 | 0.007697 | -2.29007139 |
| GARNL3 | -0.074756285 | 2.210449174 | -3.05115588 | 0.002336 | 0.007697 | -2.2902942 |
| DARS2 | 0.094568166 | 3.609001058 | 3.050875249 | 0.002338 | 0.007701 | -2.29113492 |
| CRIPT | 0.08718891 | 3.634327924 | 3.050820488 | 0.002339 | 0.007701 | -2.29129896 |
| BRSK2 | -0.131845586 | 1.547969463 | -3.05072826 | 0.002339 | 0.007701 | -2.29157524 |
| TMEM117 | -0.097158026 | 2.425228471 | -3.05014133 | 0.002344 | 0.007714 | -2.29333327 |
| MLLT10 | -0.084685807 | 3.344917933 | -3.04954851 | 0.002349 | 0.007727 | -2.29510856 |
| TP53BP1 | -0.074654639 | 3.279335387 | -3.04854822 | 0.002356 | 0.00775 | -2.29810336 |
| NCBP2 | 0.091830069 | 4.191957979 | 3.048393086 | 0.002358 | 0.007752 | -2.29856775 |
| WDR76 | 0.104493993 | 2.515824518 | 3.047705306 | 0.002363 | 0.007765 | -2.30062625 |
| STMN3 | -0.153697976 | 3.021606237 | -3.04767136 | 0.002363 | 0.007765 | -2.30072782 |
| TSHZ2 | -0.119072508 | 2.82036135 | -3.04756688 | 0.002364 | 0.007766 | -2.30104051 |
| EFCAB2 | -0.108065059 | 2.618678432 | -3.04737603 | 0.002365 | 0.007768 | -2.30161159 |
| HOXB6 | 0.231965601 | 2.796997552 | 3.046881854 | 0.002369 | 0.007776 | -2.30309021 |
| ZNF529 | -0.088783268 | 2.927128134 | -3.04688029 | 0.002369 | 0.007776 | -2.3030949 |
| ZNF703 | -0.16548356 | 3.771010407 | -3.04646514 | 0.002373 | 0.007785 | -2.30433686 |
| GRID1 | -0.115133274 | 1.702844246 | -3.04609976 | 0.002375 | 0.007792 | -2.30542981 |
| HOMER1 | 0.099149861 | 2.541075366 | 3.045017802 | 0.002384 | 0.007817 | -2.30866548 |
| PKHD1 | -0.149315828 | 1.755576742 | -3.04444631 | 0.002388 | 0.007829 | -2.31037412 |
| ZNF225 | -0.066451054 | 2.028588085 | -3.04439589 | 0.002389 | 0.007829 | -2.31052484 |
| ANXA5 | -0.101295525 | 5.097658582 | -3.04401592 | 0.002392 | 0.007836 | -2.31166067 |
| ZFC3H1 | -0.077337413 | 3.208928726 | -3.04374449 | 0.002394 | 0.007841 | -2.31247197 |
| DPH2 | 0.090974962 | 3.695043123 | 3.043073601 | 0.002399 | 0.007856 | -2.3144769 |
| RPL41 | -0.092043009 | 6.755878528 | -3.04232317 | 0.002405 | 0.007873 | -2.31671905 |
| SP4 | -0.079089398 | 2.56894413 | -3.04168982 | 0.00241 | 0.007886 | -2.31861095 |
| ILF2 | 0.084664406 | 5.348342979 | 3.041631915 | 0.002411 | 0.007886 | -2.31878389 |
| NT5C2 | -0.09247691 | 3.641945962 | -3.040599 | 0.002419 | 0.007909 | -2.32186846 |
| SUMO1 | 0.075056925 | 4.713946339 | 3.040577939 | 0.002419 | 0.007909 | -2.32193135 |
| TRUB2 | 0.078629195 | 3.357789202 | 3.039602079 | 0.002427 | 0.007932 | -2.32484457 |
| ACSS2 | 0.095783854 | 3.674514627 | 3.039402905 | 0.002428 | 0.007935 | -2.32543905 |
| CD5 | -0.118590036 | 1.874299579 | -3.03845051 | 0.002436 | 0.007958 | -2.32828116 |
| RPL35 | 0.105763538 | 6.544211553 | 3.038021205 | 0.002439 | 0.007967 | -2.32956198 |
| WNT10A | -0.222191788 | 3.264634749 | -3.03713059 | 0.002447 | 0.007988 | -2.33221856 |
| HIF3A | -0.182406266 | 2.664723258 | -3.0365406 | 0.002451 | 0.008001 | -2.33397801 |
| DBT | 0.077295719 | 2.917899219 | 3.036404673 | 0.002452 | 0.008002 | -2.33438331 |
| RBL1 | 0.078150095 | 2.497678682 | 3.03572931 | 0.002458 | 0.008017 | -2.33639684 |
| PIGG | -0.074903732 | 3.150987788 | -3.03521137 | 0.002462 | 0.008029 | -2.33794073 |
| DDX50 | -0.063465841 | 3.71088699 | -3.03475925 | 0.002466 | 0.008036 | -2.3392882 |
| RAB18 | 0.077317564 | 3.839151372 | 3.034753797 | 0.002466 | 0.008036 | -2.33930446 |
| TLR8 | -0.124026341 | 1.606537713 | -3.03452457 | 0.002468 | 0.00804 | -2.33998758 |
| FAM120A | 0.081850688 | 4.318290278 | 3.033341941 | 0.002477 | 0.008069 | -2.34351102 |
| IFI44 | 0.178485836 | 4.262853311 | 3.032268855 | 0.002486 | 0.008095 | -2.34670695 |
| ATP8B1 | 0.114386705 | 3.284822895 | 3.031707455 | 0.002491 | 0.008107 | -2.3483785 |
| HLA-B | 0.176167927 | 6.337208747 | 3.031085634 | 0.002496 | 0.00812 | -2.3502296 |
| EEF1A1 | -0.095866864 | 7.298621228 | -3.03104195 | 0.002496 | 0.00812 | -2.35035963 |
| TRAF7 | -0.086124307 | 4.221446953 | -3.02980848 | 0.002506 | 0.008148 | -2.35403039 |
| NUP62CL | 0.130557245 | 1.987402768 | 3.029805731 | 0.002506 | 0.008148 | -2.35403857 |
| SLC2A4RG | 0.101866213 | 4.501428676 | 3.029416771 | 0.002509 | 0.008156 | -2.35519579 |
| ARRDC1 | 0.097011993 | 3.876784687 | 3.029313171 | 0.00251 | 0.008157 | -2.355504 |
| TTYH1 | -0.227049187 | 2.012743074 | -3.02841954 | 0.002518 | 0.008178 | -2.35816207 |
| LCK | -0.130537625 | 2.106883148 | -3.02774602 | 0.002523 | 0.008194 | -2.36016491 |
| YWHAH | 0.08819159 | 4.550797591 | 3.027598277 | 0.002524 | 0.008196 | -2.3606042 |
| CYP26B1 | -0.13274666 | 1.755352528 | -3.0263321 | 0.002535 | 0.008227 | -2.36436806 |
| B3GNT8 | -0.13537366 | 1.962571371 | -3.02550958 | 0.002542 | 0.008247 | -2.36681227 |
| SLC35A2 | 0.100629589 | 3.666981463 | 3.02524656 | 0.002544 | 0.008252 | -2.36759373 |
| SPSB4 | -0.138316117 | 1.612076115 | -3.02512379 | 0.002545 | 0.008253 | -2.36795848 |
| SUOX | -0.073095626 | 3.200897505 | -3.02210821 | 0.00257 | 0.008332 | -2.3769129 |
| DLK1 | -0.23065014 | 1.353919192 | -3.02158746 | 0.002575 | 0.008344 | -2.37845832 |
| FLOT1 | 0.101190994 | 5.124577811 | 3.020674704 | 0.002582 | 0.008367 | -2.38116646 |
| TRDMT1 | -0.08025439 | 2.240851912 | -3.01959258 | 0.002592 | 0.008394 | -2.38437608 |
| CLPP | 0.091210789 | 3.826779199 | 3.01931679 | 0.002594 | 0.008399 | -2.38519389 |
| CYGB | -0.113616996 | 2.30896454 | -3.01908859 | 0.002596 | 0.008403 | -2.38587054 |
| ZNF16 | 0.087358784 | 3.099270864 | 3.01892077 | 0.002597 | 0.008405 | -2.38636811 |
| KLHDC7B | 0.152497228 | 2.020812751 | 3.017695186 | 0.002608 | 0.008436 | -2.39000106 |
| HARBI1 | 0.077447467 | 2.08353787 | 3.017234154 | 0.002612 | 0.008446 | -2.39136731 |
| CBX7 | -0.092474871 | 2.697661158 | -3.01715018 | 0.002612 | 0.008446 | -2.39161614 |
| NOTUM | -0.212766848 | 2.045809708 | -3.01663378 | 0.002617 | 0.008458 | -2.39314618 |
| KBTBD7 | -0.085775786 | 2.478959377 | -3.01601795 | 0.002622 | 0.008473 | -2.39497049 |
| SPAG7 | -0.081131596 | 3.732673245 | -3.0138604 | 0.002641 | 0.00853 | -2.40135902 |
| AKAP12 | -0.167580777 | 3.301847599 | -3.01353678 | 0.002643 | 0.008537 | -2.40231687 |
| TFCP2 | -0.081152715 | 3.753338982 | -3.01318776 | 0.002646 | 0.008544 | -2.40334981 |
| SULT2B1 | 0.177484307 | 1.796186576 | 3.012993166 | 0.002648 | 0.008547 | -2.40392565 |
| ZNF239 | -0.097189046 | 3.028975367 | -3.01278716 | 0.00265 | 0.00855 | -2.40453522 |
| SCFD1 | 0.073130172 | 3.4757489 | 3.012623046 | 0.002651 | 0.008552 | -2.40502082 |
| CAPZA1 | 0.073537799 | 4.457920536 | 3.011823665 | 0.002658 | 0.008572 | -2.4073857 |
| GIPC1 | 0.104065246 | 4.758372954 | 3.011397425 | 0.002662 | 0.008581 | -2.40864644 |
| PTPRF | 0.11285102 | 5.205974347 | 3.010898965 | 0.002666 | 0.008593 | -2.41012056 |
| TUSC3 | -0.140919099 | 3.542065294 | -3.00998596 | 0.002674 | 0.008616 | -2.41282001 |
| KLRB1 | -0.135698431 | 1.502459737 | -3.00969752 | 0.002677 | 0.008622 | -2.41367268 |
| LY9 | -0.099019489 | 1.579370081 | -3.00899841 | 0.002683 | 0.008637 | -2.41573896 |
| FOXF1 | -0.106534172 | 1.625651847 | -3.00898163 | 0.002683 | 0.008637 | -2.41578858 |
| ARL8B | 0.076529636 | 4.130768458 | 3.008512549 | 0.002687 | 0.008648 | -2.41717472 |
| PNKP | 0.088136789 | 3.656642424 | 3.00827151 | 0.002689 | 0.008652 | -2.41788691 |
| ESRRG | -0.128844623 | 2.027698376 | -3.00783912 | 0.002693 | 0.008662 | -2.41916436 |
| ZNF408 | 0.073773498 | 3.026402414 | 3.007416283 | 0.002697 | 0.008671 | -2.42041339 |
| NPDC1 | -0.159873358 | 3.853415721 | -3.00638065 | 0.002706 | 0.008698 | -2.42347186 |
| TSPAN14 | 0.076904788 | 3.970276053 | 3.006192559 | 0.002707 | 0.008701 | -2.42402725 |
| CCDC65 | -0.154830381 | 1.819983802 | -3.00528146 | 0.002716 | 0.008724 | -2.42671692 |
| CREB3L4 | -0.0978028 | 3.584251367 | -3.00493392 | 0.002719 | 0.008731 | -2.42774269 |
| CPT1C | -0.147906923 | 2.292261359 | -3.00384858 | 0.002728 | 0.00876 | -2.43094537 |
| STAT1 | 0.137002431 | 4.865801319 | 3.003429459 | 0.002732 | 0.008769 | -2.43218181 |
| CAMK1 | -0.084291939 | 2.452037112 | -3.00327162 | 0.002733 | 0.008771 | -2.4326474 |
| SLC45A3 | -0.104843929 | 2.390297287 | -3.00277557 | 0.002738 | 0.00878 | -2.43411052 |
| TACR2 | -0.128820155 | 1.568662544 | -3.0027718 | 0.002738 | 0.00878 | -2.43412163 |
| CALCOCO2 | -0.077828892 | 3.415079647 | -3.00240422 | 0.002741 | 0.008788 | -2.43520564 |
| TMEM26 | -0.087927707 | 1.542542095 | -3.00162823 | 0.002748 | 0.008808 | -2.43749371 |
| LSM1 | 0.095904768 | 3.620580925 | 3.001284173 | 0.002751 | 0.008815 | -2.43850797 |
| C16orf46 | -0.087849354 | 1.935431458 | -3.00056262 | 0.002758 | 0.008831 | -2.44063475 |
| ZHX3 | -0.079646482 | 2.597776653 | -3.00054133 | 0.002758 | 0.008831 | -2.44069747 |
| VIPR2 | -0.121068746 | 1.463907937 | -2.99995023 | 0.002763 | 0.008846 | -2.44243936 |
| PRKAR1B | -0.107718666 | 3.250878153 | -2.99903703 | 0.002771 | 0.00887 | -2.44512973 |
| TPD52L1 | 0.143602915 | 3.676538558 | 2.998604299 | 0.002775 | 0.00888 | -2.44640432 |
| ATP13A3 | 0.091251679 | 3.920301402 | 2.998313776 | 0.002778 | 0.008885 | -2.44725994 |
| SBF2 | -0.057481054 | 2.844806841 | -2.99722637 | 0.002788 | 0.008914 | -2.45046175 |
| TNFRSF25 | -0.113308313 | 3.106242184 | -2.99661409 | 0.002793 | 0.00893 | -2.45226406 |
| ELOVL6 | 0.125203571 | 2.908641604 | 2.995488866 | 0.002803 | 0.008958 | -2.45557535 |
| TSPAN15 | 0.104538194 | 3.879498956 | 2.995468495 | 0.002804 | 0.008958 | -2.45563528 |
| LPIN1 | -0.088351419 | 3.083201635 | -2.99510964 | 0.002807 | 0.008966 | -2.45669105 |
| RAPGEF5 | -0.09257329 | 2.870404972 | -2.99477533 | 0.00281 | 0.008973 | -2.45767449 |
| SNRPC | 0.092967565 | 4.92096451 | 2.994568059 | 0.002812 | 0.008976 | -2.45828417 |
| ZNF217 | 0.102459967 | 4.439424703 | 2.9937032 | 0.00282 | 0.008999 | -2.46082766 |
| FOXP4 | -0.134913344 | 4.37259437 | -2.99348232 | 0.002822 | 0.009003 | -2.46147713 |
| POP1 | 0.069843024 | 2.479542675 | 2.992593574 | 0.00283 | 0.009026 | -2.46408993 |
| PPOX | -0.088128945 | 3.077018001 | -2.99231704 | 0.002833 | 0.009032 | -2.46490275 |
| SNRPF | 0.10061774 | 4.028364561 | 2.991666119 | 0.002839 | 0.009048 | -2.46681571 |
| LIMS1 | 0.092624521 | 3.554491433 | 2.99105786 | 0.002844 | 0.009063 | -2.46860293 |
| UBR7 | 0.086737828 | 3.452430455 | 2.990940143 | 0.002845 | 0.009064 | -2.46894877 |
| NKD2 | -0.176798093 | 3.000826347 | -2.9895994 | 0.002858 | 0.009101 | -2.4728868 |
| RP2 | 0.088776154 | 2.734725777 | 2.988993473 | 0.002863 | 0.009112 | -2.47466595 |
| AGAP2 | -0.084878251 | 1.859298393 | -2.98898693 | 0.002863 | 0.009112 | -2.47468516 |
| HADH | 0.099924952 | 3.931931546 | 2.98886659 | 0.002864 | 0.009112 | -2.47503847 |
| SLCO5A1 | -0.096245917 | 1.465349434 | -2.98885935 | 0.002865 | 0.009112 | -2.47505972 |
| SLC44A1 | 0.08128475 | 3.616130793 | 2.988799925 | 0.002865 | 0.009112 | -2.47523418 |
| PFDN1 | 0.068376723 | 4.194494583 | 2.987872004 | 0.002874 | 0.009137 | -2.47795794 |
| CLTB | 0.095105379 | 4.204263515 | 2.987534097 | 0.002877 | 0.009142 | -2.4789496 |
| SLC8A1 | -0.085337986 | 2.307589633 | -2.98751759 | 0.002877 | 0.009142 | -2.47899803 |
| RPLP0 | -0.097064132 | 6.844366271 | -2.98551991 | 0.002896 | 0.009199 | -2.48485838 |
| ZBTB39 | -0.078802997 | 2.795771506 | -2.98504861 | 0.0029 | 0.00921 | -2.48624041 |
| NDUFA9 | 0.096433953 | 3.303642018 | 2.984777408 | 0.002903 | 0.009215 | -2.48703558 |
| MRPL20 | 0.082695632 | 4.104124239 | 2.984527394 | 0.002905 | 0.00922 | -2.48776857 |
| SIRT5 | 0.088586388 | 3.184728127 | 2.9836158 | 0.002914 | 0.009245 | -2.49044064 |
| LOX | 0.192672913 | 3.098001297 | 2.981770872 | 0.002931 | 0.009298 | -2.49584605 |
| CHST1 | 0.187378588 | 2.977272747 | 2.980212354 | 0.002946 | 0.009342 | -2.50040976 |
| ZC3H10 | -0.061840805 | 2.401045457 | -2.97942104 | 0.002953 | 0.009363 | -2.502726 |
| PROX1 | -0.098207741 | 1.466424689 | -2.97859066 | 0.002961 | 0.009386 | -2.50515599 |
| NEU4 | -0.157262887 | 1.966647129 | -2.97803679 | 0.002967 | 0.0094 | -2.50677639 |
| COL6A3 | -0.197239663 | 4.581931467 | -2.97769848 | 0.00297 | 0.009408 | -2.50776603 |
| DDX19A | -0.067242242 | 3.020861505 | -2.97724589 | 0.002974 | 0.009419 | -2.50908977 |
| PAX6 | 0.202301315 | 1.524564234 | 2.977104228 | 0.002976 | 0.00942 | -2.50950408 |
| TWF2 | 0.098298858 | 3.638340891 | 2.976712801 | 0.002979 | 0.009426 | -2.51064873 |
| SLC35B1 | 0.078110765 | 3.544783221 | 2.976657749 | 0.00298 | 0.009426 | -2.5108097 |
| CXCL6 | 0.18864658 | 1.418210703 | 2.97664276 | 0.00298 | 0.009426 | -2.51085353 |
| RPS12 | -0.110970905 | 6.908267058 | -2.97645243 | 0.002982 | 0.009429 | -2.51141003 |
| VRK2 | 0.065278522 | 3.168601124 | 2.976240428 | 0.002984 | 0.009433 | -2.51202989 |
| FAM160B2 | -0.085390511 | 3.208648912 | -2.97607233 | 0.002986 | 0.009433 | -2.51252134 |
| TUFM | 0.083900181 | 4.986526726 | 2.976066225 | 0.002986 | 0.009433 | -2.51253918 |
| CEBPD | 0.141610102 | 4.675323496 | 2.975595637 | 0.00299 | 0.009445 | -2.51391483 |
| TASP1 | -0.070500281 | 3.001648567 | -2.97537494 | 0.002992 | 0.009448 | -2.51455989 |
| ELAC2 | -0.071783586 | 3.561191154 | -2.97531669 | 0.002993 | 0.009448 | -2.51473016 |
| ARHGAP18 | -0.093209693 | 3.078396705 | -2.97510029 | 0.002995 | 0.009451 | -2.51536261 |
| ETFA | 0.089423194 | 3.823414131 | 2.975029558 | 0.002996 | 0.009451 | -2.51556933 |
| RAB9B | -0.109870121 | 1.578824659 | -2.97405951 | 0.003005 | 0.009478 | -2.51840382 |
| EIF4G1 | 0.096060241 | 5.136535821 | 2.973650279 | 0.003009 | 0.009488 | -2.51959932 |
| VILL | -0.113151454 | 2.612426431 | -2.97295515 | 0.003016 | 0.009505 | -2.52162966 |
| P2RY10 | -0.120406213 | 1.430702704 | -2.97291653 | 0.003016 | 0.009505 | -2.52174244 |
| GDF15 | 0.192896205 | 2.505646791 | 2.972799356 | 0.003017 | 0.009506 | -2.52208464 |
| RIC8B | -0.07448494 | 2.849660635 | -2.97245932 | 0.003021 | 0.009514 | -2.52307761 |
| CREB1 | -0.064469918 | 3.352675333 | -2.97156635 | 0.003029 | 0.009539 | -2.52568468 |
| BMP8B | 0.13163853 | 2.051392465 | 2.970341714 | 0.003041 | 0.009571 | -2.52925883 |
| ATP6V0E2 | 0.116841334 | 3.740452541 | 2.970341502 | 0.003041 | 0.009571 | -2.52925945 |
| CXorf56 | 0.072753285 | 3.138647936 | 2.969102663 | 0.003054 | 0.009606 | -2.53287358 |
| CCNT2 | -0.070517104 | 3.166930418 | -2.96847456 | 0.00306 | 0.009623 | -2.53470541 |
| P2RY14 | -0.11241828 | 1.177832326 | -2.96807897 | 0.003064 | 0.009633 | -2.53585894 |
| SEC61A1 | 0.073324763 | 5.032181376 | 2.966673347 | 0.003078 | 0.009674 | -2.53995643 |
| LIMA1 | -0.101812769 | 3.396971298 | -2.96497534 | 0.003095 | 0.009724 | -2.54490372 |
| IL18R1 | -0.09433601 | 1.861242062 | -2.9647958 | 0.003096 | 0.009727 | -2.54542668 |
| ELMOD3 | -0.059808457 | 2.852793976 | -2.96402992 | 0.003104 | 0.009748 | -2.54765709 |
| LYAR | 0.09295467 | 3.451993163 | 2.962735425 | 0.003117 | 0.009786 | -2.55142564 |
| PITPNB | 0.079009134 | 3.915310042 | 2.962193463 | 0.003122 | 0.009801 | -2.55300293 |
| SCN1B | -0.088961577 | 2.112835489 | -2.96146095 | 0.00313 | 0.009821 | -2.55513433 |
| DYRK4 | -0.091603554 | 2.989528154 | -2.96054733 | 0.003139 | 0.009847 | -2.557792 |
| DGCR6L | 0.105504009 | 3.781371133 | 2.96042794 | 0.00314 | 0.009848 | -2.55813923 |
| SAT2 | -0.096369183 | 3.588806286 | -2.96018953 | 0.003143 | 0.009853 | -2.55883258 |
| OXER1 | -0.110590387 | 1.653212258 | -2.95964176 | 0.003148 | 0.009867 | -2.56042543 |
| STX7 | -0.075673015 | 3.137289917 | -2.95883582 | 0.003156 | 0.00989 | -2.56276847 |
| TNFSF15 | -0.102154649 | 1.982852092 | -2.95677537 | 0.003177 | 0.009953 | -2.56875582 |
| ADIPOQ | -0.168777437 | 1.464326321 | -2.95662188 | 0.003179 | 0.009955 | -2.56920167 |
| ELFN2 | -0.111094217 | 1.524791738 | -2.95610724 | 0.003184 | 0.009969 | -2.57069641 |
| RAB25 | 0.261329954 | 3.935600491 | 2.955133206 | 0.003194 | 0.009997 | -2.57352473 |
| TMEM134 | 0.078630675 | 3.499918043 | 2.954205797 | 0.003204 | 0.010024 | -2.57621682 |
| FGFR1 | -0.146688119 | 3.944736389 | -2.95381393 | 0.003208 | 0.010034 | -2.57735408 |
| CHRNA10 | -0.100014665 | 1.541841053 | -2.95257693 | 0.003221 | 0.010071 | -2.58094309 |
| WDR20 | 0.062970004 | 2.905615676 | 2.952238648 | 0.003224 | 0.010079 | -2.58192432 |
| SYT12 | 0.126591769 | 2.165136909 | 2.95211699 | 0.003225 | 0.010081 | -2.58227718 |
| GPS2 | -0.091246873 | 2.598688361 | -2.95145479 | 0.003232 | 0.010096 | -2.58419758 |
| DCLRE1B | 0.078432292 | 2.810313 | 2.951451228 | 0.003232 | 0.010096 | -2.5842079 |
| MCM3 | 0.102603701 | 4.485985565 | 2.950622239 | 0.003241 | 0.01012 | -2.58661138 |
| FAM53B | -0.092606596 | 3.21517656 | -2.94979602 | 0.003249 | 0.010144 | -2.58900618 |
| COQ9 | 0.079294451 | 3.781692359 | 2.949359686 | 0.003254 | 0.010156 | -2.59027062 |
| SLC38A10 | -0.080690595 | 3.793463692 | -2.94825317 | 0.003266 | 0.010189 | -2.59347634 |
| APAF1 | -0.072981387 | 2.637602332 | -2.94795527 | 0.003269 | 0.010196 | -2.59433921 |
| GPC6 | -0.130239457 | 2.449144441 | -2.94743078 | 0.003274 | 0.01021 | -2.59585816 |
| CCDC120 | 0.104899806 | 3.298280862 | 2.946629551 | 0.003283 | 0.010234 | -2.59817806 |
| CENPH | 0.115633037 | 2.954578351 | 2.945747384 | 0.003292 | 0.01026 | -2.60073159 |
| SIGLEC11 | -0.142088011 | 1.332871101 | -2.94436442 | 0.003307 | 0.010302 | -2.60473323 |
| RNASEH2B | -0.066373426 | 2.83851785 | -2.94417165 | 0.003309 | 0.010306 | -2.60529086 |
| SUPV3L1 | 0.068917856 | 3.571114893 | 2.943839615 | 0.003312 | 0.010314 | -2.60625127 |
| TIMP2 | -0.134492176 | 4.820401765 | -2.9433435 | 0.003317 | 0.010327 | -2.60768609 |
| C4orf46 | 0.105289029 | 2.785031476 | 2.942925521 | 0.003322 | 0.010338 | -2.60889474 |
| BBOX1 | 0.22709112 | 2.63570484 | 2.94274845 | 0.003324 | 0.010341 | -2.60940671 |
| STK25 | -0.077416477 | 4.154873909 | -2.94107579 | 0.003342 | 0.010393 | -2.6142415 |
| OSGIN1 | 0.111597159 | 1.750011268 | 2.941003594 | 0.003342 | 0.010393 | -2.61445012 |
| BFSP1 | -0.110310362 | 1.59234448 | -2.94014834 | 0.003351 | 0.010419 | -2.61692112 |
| TMEM163 | -0.1284617 | 1.714655856 | -2.93995575 | 0.003354 | 0.010422 | -2.61747744 |
| NUDT1 | 0.095765741 | 3.022396458 | 2.939333496 | 0.00336 | 0.01044 | -2.61927468 |
| ZCCHC3 | -0.113677425 | 3.081248273 | -2.93750629 | 0.00338 | 0.010499 | -2.62455001 |
| CASQ2 | -0.116760044 | 1.309484075 | -2.93617715 | 0.003394 | 0.010538 | -2.62838534 |
| RRP9 | 0.083958701 | 3.748858052 | 2.936171621 | 0.003394 | 0.010538 | -2.6284013 |
| ALDH1A1 | -0.174510412 | 2.916390505 | -2.93589493 | 0.003397 | 0.010543 | -2.62919949 |
| FLCN | -0.066511212 | 2.807390387 | -2.93583188 | 0.003398 | 0.010543 | -2.62938138 |
| POFUT1 | -0.079156945 | 3.910470495 | -2.93554461 | 0.003401 | 0.01055 | -2.63020998 |
| ROR2 | -0.139101733 | 2.791969141 | -2.93436869 | 0.003414 | 0.010587 | -2.63360107 |
| NEXN | -0.122722737 | 2.563836677 | -2.93342691 | 0.003424 | 0.010616 | -2.63631595 |
| MRPL27 | 0.092170086 | 3.724855186 | 2.933104587 | 0.003428 | 0.010623 | -2.63724493 |
| PARD3 | -0.083140728 | 3.906460692 | -2.93215377 | 0.003438 | 0.010652 | -2.63998472 |
| THUMPD3 | 0.066124681 | 3.362882938 | 2.932081805 | 0.003439 | 0.010652 | -2.64019204 |
| C14orf119 | 0.076144124 | 3.911968662 | 2.931063778 | 0.00345 | 0.010682 | -2.64312445 |
| ZNF337 | -0.072920083 | 2.776942335 | -2.93104472 | 0.00345 | 0.010682 | -2.64317933 |
| GBF1 | -0.075085515 | 3.908278416 | -2.93062186 | 0.003455 | 0.010693 | -2.64439709 |
| FANCA | 0.091876565 | 2.773276421 | 2.930194172 | 0.00346 | 0.010705 | -2.64562855 |
| PIK3CG | -0.088452169 | 1.957584734 | -2.92989643 | 0.003463 | 0.01071 | -2.64648576 |
| GADD45A | 0.120195229 | 3.972997283 | 2.92981385 | 0.003464 | 0.01071 | -2.64672348 |
| C14orf93 | -0.074244426 | 2.699090382 | -2.9297616 | 0.003465 | 0.01071 | -2.64687389 |
| NOS1 | -0.13909845 | 1.488554606 | -2.92871898 | 0.003476 | 0.010743 | -2.64987479 |
| ZNF552 | 0.079093525 | 2.765894878 | 2.928356601 | 0.00348 | 0.010753 | -2.65091756 |
| NDUFB1 | 0.0959297 | 4.031187116 | 2.927965723 | 0.003485 | 0.010763 | -2.65204219 |
| MCTP1 | -0.085436188 | 1.945657217 | -2.92666893 | 0.003499 | 0.010805 | -2.65577224 |
| RRP12 | -0.081040115 | 3.168580255 | -2.92651693 | 0.003501 | 0.010807 | -2.65620935 |
| ANAPC4 | -0.064440333 | 3.057793527 | -2.92607756 | 0.003506 | 0.010819 | -2.65747271 |
| ZBTB32 | -0.105259255 | 1.312650973 | -2.92581126 | 0.003509 | 0.010825 | -2.65823834 |
| KCNC4 | -0.089111014 | 2.232451447 | -2.9256235 | 0.003511 | 0.010829 | -2.6587781 |
| PPP1R16A | 0.113639278 | 4.172800049 | 2.924829415 | 0.00352 | 0.01085 | -2.66106058 |
| SLC12A9 | -0.082510726 | 3.281826693 | -2.92481304 | 0.00352 | 0.01085 | -2.66110765 |
| ZNF43 | -0.089020119 | 3.181576947 | -2.92459965 | 0.003522 | 0.01085 | -2.6617209 |
| HLA-DMB | -0.154323143 | 3.934660759 | -2.92454331 | 0.003523 | 0.01085 | -2.66188281 |
| RAMP3 | -0.108600931 | 2.18025011 | -2.92450539 | 0.003523 | 0.01085 | -2.66199176 |
| SLC38A3 | -0.144397723 | 1.645057274 | -2.92447711 | 0.003524 | 0.01085 | -2.66207303 |
| FCHSD1 | -0.083832704 | 2.974360089 | -2.92391028 | 0.00353 | 0.010867 | -2.66370173 |
| CCDC8 | -0.128012788 | 3.906215616 | -2.92382132 | 0.003531 | 0.010867 | -2.6639573 |
| BBS5 | -0.085900316 | 2.431428907 | -2.92286239 | 0.003542 | 0.010897 | -2.66671187 |
| GAS1 | -0.177975072 | 4.025296831 | -2.92248662 | 0.003546 | 0.010907 | -2.66779104 |
| RANBP9 | 0.08086585 | 3.992647469 | 2.921490601 | 0.003557 | 0.010939 | -2.67065082 |
| HPS3 | 0.080311713 | 3.400300361 | 2.919298259 | 0.003582 | 0.011012 | -2.67694213 |
| ITLN1 | -0.177361364 | 1.150583202 | -2.91920997 | 0.003583 | 0.011012 | -2.67719538 |
| CCDC88B | -0.087178366 | 2.652787752 | -2.9190602 | 0.003585 | 0.011014 | -2.677625 |
| PLAU | 0.202377171 | 3.977417905 | 2.91862464 | 0.00359 | 0.011027 | -2.67887427 |
| PHF7 | 0.080994868 | 2.051670197 | 2.91812871 | 0.003595 | 0.01104 | -2.68029646 |
| C6orf118 | -0.173358357 | 1.405302767 | -2.91806761 | 0.003596 | 0.01104 | -2.68047168 |
| USP34 | -0.076894943 | 3.516361292 | -2.91789557 | 0.003598 | 0.011043 | -2.68096495 |
| KNDC1 | -0.132552072 | 1.563669824 | -2.91748319 | 0.003603 | 0.011054 | -2.68214729 |
| MCC | -0.104348715 | 2.611925307 | -2.91708771 | 0.003607 | 0.011065 | -2.68328099 |
| CRLF3 | -0.073722258 | 2.9444472 | -2.91631584 | 0.003616 | 0.011089 | -2.68549326 |
| DIS3L2 | -0.065306763 | 2.998998854 | -2.9161352 | 0.003618 | 0.011093 | -2.68601091 |
| MACROD1 | 0.118603427 | 3.261011006 | 2.914580118 | 0.003636 | 0.011145 | -2.69046591 |
| PDE3B | -0.078644526 | 1.791144916 | -2.91375581 | 0.003646 | 0.011171 | -2.69282643 |
| NR1I2 | -0.11020647 | 1.472119532 | -2.91361955 | 0.003647 | 0.011172 | -2.69321658 |
| LAP3 | 0.11449431 | 4.436203304 | 2.913351085 | 0.00365 | 0.011179 | -2.6939852 |
| HIVEP3 | -0.075988427 | 2.388917494 | -2.91311122 | 0.003653 | 0.011184 | -2.69467188 |
| FGD6 | -0.077272318 | 2.453028855 | -2.91263229 | 0.003659 | 0.011198 | -2.69604276 |
| CCNO | 0.202119813 | 2.56509557 | 2.912263113 | 0.003663 | 0.011208 | -2.69709935 |
| DSCAML1 | -0.144145256 | 1.888376122 | -2.91146779 | 0.003672 | 0.011233 | -2.69937511 |
| CRISPLD2 | -0.173076747 | 3.16100886 | -2.91127581 | 0.003675 | 0.011237 | -2.69992436 |
| SPI1 | -0.134792619 | 3.45931596 | -2.91102301 | 0.003678 | 0.011243 | -2.70064755 |
| ZDHHC15 | -0.112171669 | 1.533219502 | -2.91089794 | 0.003679 | 0.011244 | -2.70100532 |
| ALDOC | 0.156060703 | 2.66755599 | 2.910533902 | 0.003683 | 0.011252 | -2.70204658 |
| MYO10 | 0.118082893 | 3.619199985 | 2.910523849 | 0.003683 | 0.011252 | -2.70207533 |
| BMP8A | -0.114770628 | 1.51108018 | -2.90989558 | 0.003691 | 0.011269 | -2.70387208 |
| NUDT15 | 0.106342533 | 3.647502313 | 2.909863641 | 0.003691 | 0.011269 | -2.70396341 |
| CD300A | 0.112132581 | 2.60582966 | 2.909032555 | 0.003701 | 0.011296 | -2.70633956 |
| DMC1 | 0.119733602 | 1.444452872 | 2.908137025 | 0.003711 | 0.011325 | -2.70889921 |
| GPD2 | 0.099899601 | 3.684344875 | 2.907758033 | 0.003716 | 0.011335 | -2.70998224 |
| FAT2 | 0.160604995 | 2.673517931 | 2.906520273 | 0.00373 | 0.011377 | -2.71351834 |
| SNRNP40 | 0.073222674 | 3.773671408 | 2.90602895 | 0.003736 | 0.011391 | -2.71492157 |
| EGR1 | -0.200056769 | 4.877696598 | -2.90563705 | 0.003741 | 0.011402 | -2.71604069 |
| ELMO1 | -0.100817875 | 2.76976454 | -2.90540572 | 0.003744 | 0.011407 | -2.71670119 |
| RHOT2 | -0.087413979 | 3.894895177 | -2.90339483 | 0.003768 | 0.011477 | -2.72244071 |
| ARHGEF4 | -0.107379567 | 2.383125461 | -2.90305794 | 0.003772 | 0.011486 | -2.72340187 |
| LAT2 | -0.102461735 | 2.517794006 | -2.90181465 | 0.003787 | 0.011528 | -2.72694811 |
| GPR20 | -0.146273911 | 1.613878138 | -2.901532 | 0.00379 | 0.011536 | -2.72775411 |
| SLC38A9 | -0.068465595 | 2.608996721 | -2.90118408 | 0.003794 | 0.011545 | -2.72874613 |
| TMEM38A | 0.118107611 | 2.731711226 | 2.901101336 | 0.003795 | 0.011545 | -2.72898204 |
| SLC30A5 | -0.066281051 | 3.451825023 | -2.89846444 | 0.003827 | 0.011639 | -2.73649653 |
| RHOJ | -0.095183355 | 2.015378695 | -2.8976492 | 0.003837 | 0.011665 | -2.73881838 |
| SNAI1 | 0.127127351 | 2.235428045 | 2.897204426 | 0.003842 | 0.011679 | -2.74008487 |
| BANK1 | -0.133992842 | 2.055395089 | -2.8968329 | 0.003847 | 0.011689 | -2.74114263 |
| THY1 | -0.162976519 | 4.319575209 | -2.89513133 | 0.003867 | 0.011749 | -2.74598542 |
| XPOT | 0.088979751 | 4.089502657 | 2.89477203 | 0.003872 | 0.011759 | -2.74700765 |
| CLSTN3 | -0.117188209 | 3.896828237 | -2.89460362 | 0.003874 | 0.011762 | -2.74748674 |
| NUP37 | 0.080484464 | 3.117519255 | 2.894288672 | 0.003878 | 0.011771 | -2.74838264 |
| MCTP2 | -0.07752793 | 2.36159326 | -2.89373678 | 0.003885 | 0.011788 | -2.7499523 |
| PFDN5 | -0.08260414 | 5.233082487 | -2.89347438 | 0.003888 | 0.011794 | -2.75069851 |
| FZD2 | -0.161164658 | 2.973616097 | -2.89289686 | 0.003895 | 0.011808 | -2.75234061 |
| ANTXR1 | -0.156945334 | 4.003916111 | -2.89289522 | 0.003895 | 0.011808 | -2.75234527 |
| BZW1 | 0.080759721 | 4.020309637 | 2.892858446 | 0.003895 | 0.011808 | -2.75244983 |
| RNF166 | -0.071945889 | 2.893575825 | -2.8926675 | 0.003898 | 0.011812 | -2.75299267 |
| CD96 | -0.101153666 | 1.932574472 | -2.89251282 | 0.0039 | 0.011814 | -2.75343239 |
| TSHZ3 | -0.109271598 | 2.380972664 | -2.89238994 | 0.003901 | 0.011815 | -2.7537817 |
| RMND1 | -0.087421393 | 3.153970759 | -2.89178036 | 0.003909 | 0.011832 | -2.7555143 |
| CD84 | -0.099242434 | 2.462329901 | -2.89177944 | 0.003909 | 0.011832 | -2.75551693 |
| LIPT1 | -0.077453276 | 2.706682619 | -2.89157541 | 0.003911 | 0.011836 | -2.75609677 |
| PLEKHO2 | -0.085486673 | 3.541868916 | -2.89124593 | 0.003915 | 0.011845 | -2.75703303 |
| AADAT | -0.109599115 | 2.223662815 | -2.89081534 | 0.003921 | 0.011858 | -2.75825647 |
| DLG1 | 0.086110248 | 3.623616303 | 2.89056581 | 0.003924 | 0.011864 | -2.75896536 |
| LAMB2 | -0.107281063 | 4.611628765 | -2.88949984 | 0.003937 | 0.011901 | -2.76199305 |
| TBC1D1 | -0.080010094 | 3.412363091 | -2.88899015 | 0.003943 | 0.011917 | -2.76344033 |
| AMBRA1 | -0.069949943 | 3.205564821 | -2.88835503 | 0.003951 | 0.011937 | -2.76524343 |
| XRCC6 | 0.066876332 | 4.824714449 | 2.887514152 | 0.003962 | 0.011966 | -2.76763009 |
| SPN | -0.093551435 | 2.109675224 | -2.8865707 | 0.003973 | 0.011998 | -2.77030706 |
| ZMYND15 | -0.106495011 | 2.368088491 | -2.88594163 | 0.003981 | 0.012019 | -2.77209152 |
| LEMD3 | -0.069648953 | 3.346887093 | -2.8854491 | 0.003987 | 0.012034 | -2.77348841 |
| RCOR1 | 0.081030057 | 3.488032512 | 2.885304901 | 0.003989 | 0.012036 | -2.77389732 |
| HEBP1 | -0.082248593 | 3.622035888 | -2.88502456 | 0.003993 | 0.012044 | -2.77469225 |
| ZFP36 | -0.174369842 | 5.130154947 | -2.88426258 | 0.004002 | 0.012069 | -2.77685254 |
| ACOX2 | -0.129590301 | 2.398002499 | -2.88327533 | 0.004015 | 0.012103 | -2.77965066 |
| GORASP2 | 0.066902187 | 4.525282368 | 2.88319498 | 0.004016 | 0.012103 | -2.77987836 |
| MAP2K4 | -0.069168657 | 3.005673213 | -2.88283551 | 0.00402 | 0.012114 | -2.78089691 |
| RAB26 | 0.130274976 | 1.887358685 | 2.882448659 | 0.004025 | 0.012125 | -2.78199294 |
| RNF112 | -0.104046999 | 1.452129339 | -2.87967247 | 0.004061 | 0.012228 | -2.78985408 |
| PLA2G4C | -0.085942506 | 1.949059306 | -2.87911538 | 0.004068 | 0.012246 | -2.79143066 |
| ARHGEF16 | 0.105753484 | 2.772273803 | 2.878900531 | 0.004071 | 0.012251 | -2.79203861 |
| CAMK2N2 | 0.16205262 | 1.950341736 | 2.878136293 | 0.00408 | 0.012278 | -2.79420077 |
| MTMR9 | -0.06299614 | 2.425754825 | -2.87789213 | 0.004083 | 0.012284 | -2.79489144 |
| APTX | 0.067587068 | 3.173477835 | 2.877509903 | 0.004088 | 0.012295 | -2.79597252 |
| KRTCAP2 | 0.073097016 | 3.736102104 | 2.876661487 | 0.004099 | 0.012325 | -2.79837166 |
| ADORA2B | 0.121278629 | 1.41988363 | 2.87609523 | 0.004107 | 0.012343 | -2.79997253 |
| SFXN2 | -0.089959932 | 2.892260495 | -2.87540023 | 0.004116 | 0.012367 | -2.80193694 |
| TLR1 | -0.099803958 | 2.354416368 | -2.87523495 | 0.004118 | 0.01237 | -2.80240404 |
| CCT7 | 0.05943392 | 4.972329173 | 2.874853115 | 0.004123 | 0.012381 | -2.80348303 |
| LRRC17 | -0.146864242 | 2.204640768 | -2.87421429 | 0.004131 | 0.012403 | -2.80528791 |
| C1QTNF4 | -0.161381963 | 1.548111226 | -2.87337045 | 0.004142 | 0.012432 | -2.80767142 |
| MMP24 | -0.127448226 | 2.311760424 | -2.87251753 | 0.004153 | 0.012462 | -2.81007988 |
| TP53I3 | -0.111439709 | 3.705491776 | -2.8719858 | 0.00416 | 0.01248 | -2.81158101 |
| ESR2 | -0.086709336 | 1.403816846 | -2.86905703 | 0.004198 | 0.012592 | -2.81984437 |
| KIF24 | 0.09741357 | 2.455169102 | 2.867323859 | 0.004221 | 0.012657 | -2.82473048 |
| GPT2 | 0.116504102 | 3.548779936 | 2.867237566 | 0.004222 | 0.012657 | -2.82497369 |
| CCDC69 | -0.10683747 | 2.389370726 | -2.86651261 | 0.004232 | 0.012682 | -2.82701655 |
| S100A10 | 0.151076231 | 5.094688326 | 2.866347328 | 0.004234 | 0.012685 | -2.82748222 |
| NME7 | -0.082215728 | 2.973254565 | -2.86624049 | 0.004236 | 0.012686 | -2.82778323 |
| USP5 | -0.081263062 | 4.183103433 | -2.86599598 | 0.004239 | 0.012691 | -2.82847206 |
| ZNF12 | -0.07448667 | 3.403030421 | -2.86589977 | 0.00424 | 0.012691 | -2.82874307 |
| IFT81 | -0.081486858 | 2.795401817 | -2.86585488 | 0.004241 | 0.012691 | -2.82886954 |
| NUBP2 | -0.08099349 | 3.610005226 | -2.86562342 | 0.004244 | 0.012697 | -2.82952152 |
| PHGDH | 0.133315362 | 4.167142543 | 2.863631395 | 0.00427 | 0.012773 | -2.83513052 |
| COMMD2 | 0.087789479 | 3.558627191 | 2.863490922 | 0.004272 | 0.012775 | -2.83552591 |
| MGST1 | 0.177957026 | 3.213015925 | 2.863290496 | 0.004275 | 0.01278 | -2.83609002 |
| PLEKHG5 | -0.093591314 | 3.224478786 | -2.86211882 | 0.004291 | 0.012823 | -2.83938697 |
| SMPD1 | -0.092652202 | 3.247828839 | -2.86182216 | 0.004295 | 0.012832 | -2.84022151 |
| FKBP11 | -0.087931092 | 3.326719416 | -2.86092694 | 0.004307 | 0.012864 | -2.8427394 |
| CD55 | 0.137061979 | 4.385121727 | 2.860832134 | 0.004308 | 0.012865 | -2.843006 |
| OBP2B | 0.214043259 | 1.672954029 | 2.860655213 | 0.00431 | 0.012868 | -2.8435035 |
| COLEC11 | -0.241554879 | 2.003657631 | -2.86020296 | 0.004316 | 0.012883 | -2.8447751 |
| LMO2 | -0.106457338 | 2.595220201 | -2.85912365 | 0.004331 | 0.012923 | -2.84780894 |
| CEACAM21 | -0.10589581 | 1.51853611 | -2.8589346 | 0.004334 | 0.012927 | -2.84834025 |
| ANKFY1 | -0.077613448 | 3.214752753 | -2.85852374 | 0.004339 | 0.01294 | -2.84949477 |
| MED19 | 0.076104772 | 3.493024099 | 2.858060351 | 0.004345 | 0.012956 | -2.85079671 |
| UBA2 | 0.095647236 | 4.34355245 | 2.857641316 | 0.004351 | 0.012969 | -2.85197385 |
| DNAJC9 | 0.078576071 | 3.24284436 | 2.856942864 | 0.004361 | 0.012994 | -2.85393555 |
| ZNF586 | 0.086732259 | 2.709396549 | 2.854503174 | 0.004394 | 0.01309 | -2.86078404 |
| UFM1 | 0.078293404 | 3.888206537 | 2.854317893 | 0.004397 | 0.013094 | -2.86130391 |
| KLHL12 | -0.074560515 | 3.655199918 | -2.85377893 | 0.004404 | 0.013112 | -2.86281598 |
| PRSS23 | 0.141440921 | 3.658918014 | 2.85353359 | 0.004407 | 0.013119 | -2.86350418 |
| PRR7 | 0.123725806 | 3.228509173 | 2.851124507 | 0.004441 | 0.013214 | -2.87025884 |
| TAS1R1 | -0.106553238 | 1.194165919 | -2.8509301 | 0.004443 | 0.013219 | -2.87080367 |
| BNIP1 | 0.080097557 | 2.655403144 | 2.849920698 | 0.004457 | 0.013255 | -2.87363201 |
| FYCO1 | -0.078423154 | 3.234451258 | -2.84981869 | 0.004459 | 0.013255 | -2.87391777 |
| CCL2 | 0.175220538 | 3.818670322 | 2.849789308 | 0.004459 | 0.013255 | -2.87400009 |
| PRAF2 | 0.115320499 | 4.042332357 | 2.84954107 | 0.004463 | 0.01326 | -2.87469547 |
| CDC37L1 | 0.081629815 | 2.903206246 | 2.849506329 | 0.004463 | 0.01326 | -2.87479278 |
| DVL2 | -0.077367597 | 3.401394813 | -2.84910802 | 0.004469 | 0.013273 | -2.8759084 |
| COX7A2L | 0.07815262 | 4.390247066 | 2.848982908 | 0.00447 | 0.013274 | -2.8762588 |
| MYH10 | -0.116087258 | 4.188945081 | -2.84794193 | 0.004485 | 0.013314 | -2.87917362 |
| LYG2 | 0.131102803 | 1.317342962 | 2.847097914 | 0.004497 | 0.013346 | -2.88153618 |
| PLXNA1 | -0.096482271 | 3.779598956 | -2.84667092 | 0.004503 | 0.01336 | -2.88273115 |
| VASP | 0.085364258 | 4.257147128 | 2.846136423 | 0.00451 | 0.013378 | -2.88422673 |
| MPP7 | 0.105722202 | 3.389626597 | 2.845981116 | 0.004512 | 0.013378 | -2.88466124 |
| MOXD1 | -0.145417991 | 2.232764583 | -2.84597431 | 0.004513 | 0.013378 | -2.88468029 |
| NCKAP1L | -0.108613465 | 2.592375142 | -2.84434531 | 0.004536 | 0.013443 | -2.88923641 |
| CDC42EP3 | -0.092951354 | 3.152558144 | -2.84318611 | 0.004552 | 0.013488 | -2.892477 |
| PPRC1 | -0.082106656 | 3.838321941 | -2.84300743 | 0.004554 | 0.013491 | -2.89297639 |
| GOSR2 | 0.058727923 | 2.922829209 | 2.840959547 | 0.004584 | 0.013574 | -2.89869781 |
| DNMT3B | 0.104069124 | 2.734117608 | 2.840803899 | 0.004586 | 0.013577 | -2.89913249 |
| POP4 | 0.113675249 | 3.533663407 | 2.840289814 | 0.004593 | 0.013592 | -2.90056804 |
| COX7A1 | -0.144300814 | 2.081862121 | -2.84008875 | 0.004596 | 0.013592 | -2.90112943 |
| APBA2 | -0.102842497 | 2.677905548 | -2.84007027 | 0.004596 | 0.013592 | -2.90118102 |
| GMFB | 0.078715882 | 3.585942459 | 2.840013529 | 0.004597 | 0.013592 | -2.90133944 |
| RAB20 | 0.099249424 | 3.436496953 | 2.839962598 | 0.004598 | 0.013592 | -2.90148163 |
| CAB39L | -0.095828269 | 2.455639287 | -2.83993956 | 0.004598 | 0.013592 | -2.90154595 |
| IER3 | 0.157392029 | 4.313119285 | 2.83922202 | 0.004608 | 0.013618 | -2.90354895 |
| LONRF3 | 0.079300531 | 1.892648435 | 2.839146404 | 0.00461 | 0.013618 | -2.90376001 |
| NALCN | -0.093927209 | 1.708804265 | -2.83864004 | 0.004617 | 0.013636 | -2.90517317 |
| BTD | -0.085811294 | 2.64427488 | -2.83768564 | 0.00463 | 0.013673 | -2.90783607 |
| TNK2 | -0.090434464 | 3.408253166 | -2.83708198 | 0.004639 | 0.013695 | -2.90951988 |
| XPNPEP1 | 0.066338703 | 3.628696754 | 2.836920474 | 0.004642 | 0.013698 | -2.90997034 |
| PHACTR3 | -0.124414173 | 1.308451071 | -2.83655448 | 0.004647 | 0.013707 | -2.910991 |
| RNF7 | 0.06859021 | 4.239433553 | 2.836539156 | 0.004647 | 0.013707 | -2.91103373 |
| SDK1 | -0.13372377 | 2.491668802 | -2.83635842 | 0.00465 | 0.013711 | -2.91153769 |
| TBRG4 | 0.07907026 | 3.65548196 | 2.835920047 | 0.004656 | 0.013723 | -2.91275996 |
| ABCB7 | 0.064778004 | 3.258847274 | 2.835892236 | 0.004656 | 0.013723 | -2.91283749 |
| RPL9 | -0.109052239 | 6.077948669 | -2.83494707 | 0.00467 | 0.01376 | -2.91547211 |
| SLC12A8 | -0.108246558 | 3.16698472 | -2.83456654 | 0.004676 | 0.013773 | -2.91653257 |
| SOCS2 | -0.103269858 | 1.784275866 | -2.83416242 | 0.004681 | 0.013786 | -2.91765864 |
| UBASH3B | 0.091004615 | 2.212739746 | 2.833173216 | 0.004696 | 0.013825 | -2.92041432 |
| PDGFC | -0.104626961 | 3.589519754 | -2.83243489 | 0.004707 | 0.013853 | -2.9224705 |
| CD59 | 0.097542555 | 4.777147125 | 2.832171672 | 0.00471 | 0.013861 | -2.92320342 |
| RASEF | 0.116764343 | 2.750824538 | 2.831605345 | 0.004719 | 0.013881 | -2.9247801 |
| RPUSD4 | -0.072315429 | 3.129521839 | -2.83154005 | 0.00472 | 0.013881 | -2.92496187 |
| TULP1 | -0.100407053 | 1.322823014 | -2.83145014 | 0.004721 | 0.013881 | -2.92521214 |
| IFITM3 | 0.115223583 | 6.78459383 | 2.830704645 | 0.004732 | 0.013909 | -2.92728705 |
| SLCO1A2 | -0.16882146 | 1.678328317 | -2.83041877 | 0.004736 | 0.013918 | -2.92808256 |
| ENTPD4 | -0.07402842 | 3.145050642 | -2.83012917 | 0.00474 | 0.013927 | -2.92888836 |
| PUM1 | -0.062078696 | 4.513773373 | -2.82987016 | 0.004744 | 0.013934 | -2.929609 |
| GJB2 | 0.221194474 | 3.119045211 | 2.829619038 | 0.004748 | 0.013941 | -2.93030761 |
| PCDHB6 | -0.140872497 | 1.851897988 | -2.82925638 | 0.004753 | 0.013953 | -2.93131639 |
| TXNDC9 | 0.080053564 | 3.45796423 | 2.827734336 | 0.004776 | 0.014016 | -2.93554884 |
| MKS1 | -0.07949444 | 2.975316085 | -2.82672701 | 0.004791 | 0.014056 | -2.93834874 |
| RNH1 | -0.065683048 | 3.954504686 | -2.82650837 | 0.004794 | 0.014061 | -2.93895631 |
| SPAG16 | -0.080699926 | 2.587087157 | -2.8259337 | 0.004802 | 0.014083 | -2.94055306 |
| PELP1 | -0.079520778 | 3.930613928 | -2.82520252 | 0.004813 | 0.014111 | -2.94258424 |
| GRSF1 | 0.073623302 | 3.978743665 | 2.8250819 | 0.004815 | 0.014113 | -2.94291926 |
| TMPO | 0.096019878 | 3.893636475 | 2.824731597 | 0.00482 | 0.014124 | -2.94389215 |
| LASP1 | -0.084369226 | 4.585677827 | -2.82455228 | 0.004823 | 0.014128 | -2.94439011 |
| SSH1 | -0.076060858 | 3.225057524 | -2.82333293 | 0.004841 | 0.014178 | -2.94777546 |
| CASS4 | -0.077455067 | 1.713731036 | -2.82311437 | 0.004845 | 0.014184 | -2.94838209 |
| TUBGCP6 | -0.081957834 | 3.090784303 | -2.82288183 | 0.004848 | 0.01419 | -2.94902751 |
| CYHR1 | 0.09411813 | 3.82921692 | 2.822705675 | 0.004851 | 0.014194 | -2.94951638 |
| RPA3 | 0.10343469 | 3.305894837 | 2.82194627 | 0.004862 | 0.014224 | -2.95162356 |
| CASP3 | 0.084788776 | 3.426934426 | 2.821331334 | 0.004871 | 0.014247 | -2.95332947 |
| KCNJ10 | -0.103991882 | 1.337816015 | -2.82102389 | 0.004876 | 0.014256 | -2.9541822 |
| TLE4 | -0.115710819 | 2.524318192 | -2.82096213 | 0.004877 | 0.014256 | -2.95435352 |
| FAM83E | 0.156673223 | 2.250148107 | 2.819833469 | 0.004894 | 0.0143 | -2.95748319 |
| ERMAP | -0.08818636 | 2.845469472 | -2.81979817 | 0.004895 | 0.0143 | -2.95758106 |
| EPHB3 | 0.14868624 | 3.58922741 | 2.819228194 | 0.004903 | 0.014321 | -2.95916105 |
| CENPQ | 0.108703447 | 2.650402056 | 2.819072586 | 0.004906 | 0.014324 | -2.95959236 |
| PDGFRL | -0.144756885 | 3.041327672 | -2.81829494 | 0.004917 | 0.014355 | -2.96174741 |
| PPM1J | 0.113346825 | 2.290671368 | 2.817892177 | 0.004924 | 0.014369 | -2.96286334 |
| TCEAL4 | -0.075898971 | 4.388053464 | -2.81754415 | 0.004929 | 0.014381 | -2.96382748 |
| EVI2B | -0.158875931 | 2.897031512 | -2.81709535 | 0.004936 | 0.014397 | -2.96507064 |
| SCNN1A | 0.126652193 | 5.107751972 | 2.816913171 | 0.004938 | 0.014399 | -2.9655752 |
| PNPLA8 | 0.069970918 | 3.178532281 | 2.816876953 | 0.004939 | 0.014399 | -2.96567551 |
| SORBS1 | -0.091969747 | 2.760723919 | -2.81665892 | 0.004942 | 0.014405 | -2.96627932 |
| IL1RAP | 0.123321126 | 3.34946049 | 2.816433974 | 0.004946 | 0.014411 | -2.96690225 |
| DCPS | 0.08361227 | 2.83403628 | 2.816256277 | 0.004948 | 0.014415 | -2.96739429 |
| SIPA1L2 | -0.099688444 | 2.966947653 | -2.81575916 | 0.004956 | 0.014434 | -2.96877064 |
| ZNF639 | 0.075051685 | 3.420658973 | 2.815601662 | 0.004958 | 0.014437 | -2.96920665 |
| NCOA7 | 0.106810779 | 3.280768921 | 2.815403366 | 0.004962 | 0.014442 | -2.96975557 |
| HS6ST2 | -0.135652286 | 1.348814637 | -2.81430329 | 0.004978 | 0.014487 | -2.97280009 |
| HOXC6 | -0.130994516 | 2.207034772 | -2.81416217 | 0.004981 | 0.01449 | -2.97319056 |
| PLA2G3 | 0.110991666 | 1.23648739 | 2.811386752 | 0.005023 | 0.014611 | -2.98086606 |
| UBE2K | 0.071428153 | 4.074418738 | 2.810121235 | 0.005043 | 0.014664 | -2.98436341 |
| OAZ2 | -0.066958479 | 3.944994237 | -2.80959603 | 0.005051 | 0.014684 | -2.9858144 |
| TDP1 | 0.071964488 | 2.971028919 | 2.809411026 | 0.005054 | 0.014688 | -2.98632545 |
| PSMD11 | 0.061374779 | 3.74949008 | 2.809299387 | 0.005056 | 0.014689 | -2.98663382 |
| C17orf80 | -0.069392536 | 3.1004583 | -2.80877841 | 0.005064 | 0.014708 | -2.98807272 |
| PYCR1 | 0.116666493 | 3.837549344 | 2.808705209 | 0.005065 | 0.014708 | -2.98827487 |
| MRPS18A | 0.08793273 | 4.025777727 | 2.808639373 | 0.005066 | 0.014708 | -2.98845668 |
| MFSD11 | -0.065683396 | 2.989920703 | -2.80826347 | 0.005072 | 0.014721 | -2.98949468 |
| DYNC1LI2 | -0.091986923 | 3.572542617 | -2.80816359 | 0.005074 | 0.014722 | -2.98977045 |
| DYNLL2 | 0.07644675 | 3.703057892 | 2.807924774 | 0.005077 | 0.014729 | -2.99042981 |
| SLC43A1 | -0.099261318 | 2.233335832 | -2.80742773 | 0.005085 | 0.014747 | -2.99180195 |
| SPNS1 | -0.071537708 | 2.482026133 | -2.80711842 | 0.00509 | 0.014754 | -2.99265571 |
| GBP2 | 0.137254498 | 3.583225881 | 2.807104239 | 0.00509 | 0.014754 | -2.99269485 |
| PTPRU | -0.133936317 | 3.862219975 | -2.80672324 | 0.005096 | 0.014768 | -2.99374635 |
| NR3C2 | -0.097086907 | 2.215530124 | -2.80637792 | 0.005102 | 0.01478 | -2.99469928 |
| ZNF577 | -0.081091704 | 2.559783505 | -2.80619496 | 0.005104 | 0.014784 | -2.99520409 |
| DLST | 0.08026401 | 4.043270556 | 2.805805362 | 0.005111 | 0.014798 | -2.99627899 |
| FRAS1 | -0.104659661 | 2.587420148 | -2.80422294 | 0.005136 | 0.014866 | -3.00064332 |
| APOM | -0.124460205 | 1.984536627 | -2.80384644 | 0.005142 | 0.014877 | -3.00168138 |
| ALOX15B | 0.132207671 | 1.890878038 | 2.803811456 | 0.005142 | 0.014877 | -3.00177782 |
| THADA | -0.056352382 | 3.162812481 | -2.80284791 | 0.005157 | 0.014918 | -3.00443371 |
| SLC2A8 | 0.085618253 | 2.837489079 | 2.802598376 | 0.005161 | 0.014925 | -3.00512138 |
| CCR2 | -0.122501436 | 1.707448973 | -2.80189438 | 0.005172 | 0.014954 | -3.00706112 |
| FZD6 | 0.107370166 | 3.927583974 | 2.801576466 | 0.005178 | 0.014964 | -3.00793693 |
| MTHFSD | -0.064501415 | 2.564288979 | -2.80117396 | 0.005184 | 0.014979 | -3.00904562 |
| STK17B | 0.103065335 | 3.360157497 | 2.800940734 | 0.005188 | 0.014986 | -3.00968798 |
| THUMPD1 | -0.074299019 | 3.645497652 | -2.79944098 | 0.005212 | 0.015051 | -3.01381731 |
| GDA | 0.163185278 | 1.593221481 | 2.798744767 | 0.005223 | 0.01508 | -3.01573349 |
| RNASET2 | 0.097206074 | 4.053391439 | 2.798323729 | 0.00523 | 0.015095 | -3.01689208 |
| ZNF555 | -0.055370022 | 1.993561814 | -2.79814785 | 0.005232 | 0.015099 | -3.01737601 |
| NAALADL1 | -0.118681535 | 2.22050267 | -2.79804764 | 0.005234 | 0.0151 | -3.01765171 |
| PYGO2 | -0.070108736 | 4.050614275 | -2.79708918 | 0.005249 | 0.015141 | -3.02028825 |
| ANKRD22 | 0.142222235 | 1.943439213 | 2.796869081 | 0.005253 | 0.015147 | -3.02089357 |
| ITK | -0.095989657 | 1.813521995 | -2.79668705 | 0.005256 | 0.015151 | -3.02139416 |
| NUP210 | 0.107807945 | 3.720116686 | 2.795932662 | 0.005268 | 0.015183 | -3.02346841 |
| MACROD2 | -0.11803861 | 2.55965021 | -2.79539654 | 0.005277 | 0.015204 | -3.0249422 |
| IFI35 | 0.133138054 | 3.702583004 | 2.795278146 | 0.005279 | 0.015205 | -3.02526761 |
| TRPC4AP | -0.068936981 | 4.432954958 | -2.79517249 | 0.00528 | 0.015206 | -3.02555801 |
| MED14 | 0.069865495 | 3.31226063 | 2.794636971 | 0.005289 | 0.015227 | -3.02702974 |
| SOX5 | -0.106563688 | 2.507151549 | -2.79373114 | 0.005304 | 0.015266 | -3.02951852 |
| GDAP1 | -0.111889517 | 2.072526906 | -2.79239725 | 0.005326 | 0.015324 | -3.03318197 |
| ZNHIT1 | 0.085547311 | 4.275174778 | 2.791799436 | 0.005335 | 0.015348 | -3.03482328 |
| SYT7 | 0.119386887 | 2.396646268 | 2.788821421 | 0.005384 | 0.015485 | -3.04299427 |
| TACR1 | 0.153633926 | 1.704595496 | 2.788053853 | 0.005397 | 0.015518 | -3.04509891 |
| MINPP1 | -0.0821401 | 3.070456847 | -2.78739929 | 0.005408 | 0.015545 | -3.04689324 |
| ACAT1 | 0.088312474 | 3.122402472 | 2.786856453 | 0.005417 | 0.015567 | -3.04838099 |
| DCTD | -0.075554179 | 3.971355016 | -2.78672207 | 0.005419 | 0.015569 | -3.04874926 |
| PHF1 | -0.080397705 | 3.839792957 | -2.78623644 | 0.005427 | 0.015588 | -3.05007991 |
| AKAP11 | -0.071505075 | 3.161455971 | -2.78597147 | 0.005432 | 0.015593 | -3.05080584 |
| IBSP | 0.159826965 | 1.63423078 | 2.785957516 | 0.005432 | 0.015593 | -3.05084407 |
| EIF2B5 | 0.07165825 | 3.611726775 | 2.785154079 | 0.005445 | 0.015628 | -3.05304481 |
| GTPBP4 | 0.078293718 | 3.761938431 | 2.784842361 | 0.00545 | 0.015639 | -3.05389849 |
| ZNF501 | -0.082362545 | 2.185966469 | -2.78460495 | 0.005454 | 0.015646 | -3.05454861 |
| SPDEF | 0.221869764 | 2.822379086 | 2.782279819 | 0.005493 | 0.015753 | -3.06091276 |
| HMBS | 0.079208841 | 3.233649789 | 2.781491007 | 0.005507 | 0.015787 | -3.06307064 |
| CYP1A1 | 0.099227151 | 1.08127026 | 2.78051115 | 0.005523 | 0.015831 | -3.06575031 |
| DIRAS3 | -0.150994549 | 1.950746476 | -2.77999107 | 0.005532 | 0.015852 | -3.06717222 |
| FOSL2 | 0.098084022 | 4.733065011 | 2.779312436 | 0.005543 | 0.015881 | -3.06902723 |
| PDE7B | -0.079709555 | 1.813732266 | -2.7791357 | 0.005546 | 0.015882 | -3.06951026 |
| GUK1 | 0.086010161 | 4.816060355 | 2.77910486 | 0.005547 | 0.015882 | -3.06959454 |
| CRMP1 | -0.143620577 | 2.484433139 | -2.77843416 | 0.005558 | 0.01591 | -3.07142728 |
| MTM1 | 0.099401532 | 2.993682163 | 2.778371502 | 0.005559 | 0.01591 | -3.07159848 |
| SOSTDC1 | -0.222697792 | 1.931219403 | -2.77776022 | 0.00557 | 0.015935 | -3.07326846 |
| SNAPIN | 0.072788705 | 4.120886438 | 2.776896381 | 0.005584 | 0.01597 | -3.07562776 |
| MMP16 | -0.077553463 | 1.655700819 | -2.77687353 | 0.005585 | 0.01597 | -3.07569014 |
| POLR2I | 0.097104177 | 4.380713558 | 2.776614449 | 0.005589 | 0.015979 | -3.07639761 |
| TUSC2 | 0.080385182 | 3.625859049 | 2.775955194 | 0.0056 | 0.016007 | -3.0781975 |
| LRP2 | 0.182607999 | 2.037143303 | 2.775590218 | 0.005607 | 0.016021 | -3.07919377 |
| XPC | -0.070706149 | 3.38645812 | -2.77498951 | 0.005617 | 0.016046 | -3.08083323 |
| PIN4 | 0.07841287 | 3.113710696 | 2.774400917 | 0.005627 | 0.016071 | -3.08243929 |
| HCP5 | 0.150714529 | 3.435308017 | 2.773756149 | 0.005638 | 0.016098 | -3.08419824 |
| TCF25 | -0.062572777 | 3.966129204 | -2.77363368 | 0.00564 | 0.0161 | -3.08453229 |
| PDXK | 0.087266498 | 4.097514284 | 2.773367181 | 0.005645 | 0.016108 | -3.08525917 |
| SETMAR | -0.066583109 | 2.828613845 | -2.77329919 | 0.005646 | 0.016108 | -3.08544459 |
| PEBP1 | 0.077328076 | 5.421182163 | 2.772388375 | 0.005662 | 0.016149 | -3.08792825 |
| C9orf16 | 0.101893234 | 4.776363021 | 2.771614126 | 0.005675 | 0.016183 | -3.09003888 |
| RAB3A | 0.09420958 | 2.584281099 | 2.771376119 | 0.005679 | 0.01619 | -3.09068758 |
| IQGAP2 | -0.098090013 | 2.529168041 | -2.77056611 | 0.005693 | 0.016226 | -3.09289487 |
| NTS | 0.26160221 | 1.751254527 | 2.769613624 | 0.00571 | 0.016266 | -3.09548962 |
| ZNF175 | -0.070234164 | 2.295817452 | -2.76958493 | 0.00571 | 0.016266 | -3.09556777 |
| RFX7 | -0.065463109 | 2.860391745 | -2.76866811 | 0.005726 | 0.016308 | -3.09806449 |
| MMP2 | -0.181247613 | 4.501502375 | -2.76797643 | 0.005738 | 0.016338 | -3.09994756 |
| TEAD1 | -0.093784603 | 3.648123439 | -2.76773638 | 0.005743 | 0.016346 | -3.10060099 |
| FDXR | -0.103973936 | 2.869645533 | -2.76716534 | 0.005753 | 0.01637 | -3.10215513 |
| ANKRD12 | -0.074293063 | 3.017815171 | -2.76672614 | 0.00576 | 0.016388 | -3.10335026 |
| STT3A | 0.079209627 | 4.2163603 | 2.766378615 | 0.005766 | 0.016401 | -3.10429579 |
| MED1 | -0.077492104 | 3.254318521 | -2.76522947 | 0.005787 | 0.016454 | -3.1074215 |
| RHOC | 0.084663942 | 4.405839156 | 2.764530637 | 0.005799 | 0.016485 | -3.10932171 |
| NDUFA5 | 0.072265941 | 3.635313873 | 2.763995779 | 0.005808 | 0.016502 | -3.11077573 |
| ZNF655 | -0.086433106 | 3.508153687 | -2.76396728 | 0.005809 | 0.016502 | -3.11085319 |
| CUL2 | 0.074583034 | 3.479938261 | 2.763924785 | 0.00581 | 0.016502 | -3.11096871 |
| ADAP2 | -0.099103474 | 2.956187314 | -2.76378081 | 0.005812 | 0.016505 | -3.11136006 |
| MLLT11 | -0.124462989 | 2.456037513 | -2.76230494 | 0.005838 | 0.016575 | -3.1153705 |
| WSB1 | -0.093040535 | 3.765326998 | -2.76140085 | 0.005854 | 0.016617 | -3.11782618 |
| CHST4 | 0.176261947 | 1.816161359 | 2.761193966 | 0.005858 | 0.01662 | -3.11838801 |
| F13A1 | -0.15959295 | 3.122904594 | -2.76116003 | 0.005859 | 0.01662 | -3.11848016 |
| FASTK | 0.080166266 | 4.279770389 | 2.760971871 | 0.005862 | 0.016626 | -3.1189911 |
| PNPT1 | 0.076185914 | 3.545396317 | 2.760645215 | 0.005868 | 0.016638 | -3.11987803 |
| ISLR | -0.187744163 | 4.156696767 | -2.75920083 | 0.005894 | 0.016707 | -3.12379857 |
| KCMF1 | 0.05226216 | 3.633722051 | 2.75837196 | 0.005909 | 0.016745 | -3.12604747 |
| MYCT1 | -0.08704998 | 1.712285331 | -2.75702037 | 0.005933 | 0.016809 | -3.1297132 |
| MMP7 | 0.282765099 | 4.450788609 | 2.756746527 | 0.005938 | 0.016819 | -3.13045569 |
| BARX2 | -0.201568807 | 2.318533677 | -2.7562 | 0.005948 | 0.016843 | -3.13193731 |
| SLFN5 | -0.099309177 | 3.499570354 | -2.75595916 | 0.005952 | 0.016851 | -3.13259014 |
| ESPN | 0.163489947 | 3.785661634 | 2.755198584 | 0.005966 | 0.016885 | -3.13465138 |
| DOCK6 | -0.091158523 | 2.940751347 | -2.75454759 | 0.005978 | 0.016914 | -3.13641519 |
| PPM1D | -0.074939456 | 2.759874413 | -2.75416586 | 0.005984 | 0.016929 | -3.13744929 |
| TWSG1 | -0.106366591 | 3.833867828 | -2.75386237 | 0.00599 | 0.016941 | -3.13827132 |
| RPP25 | 0.133554959 | 2.951927882 | 2.753259611 | 0.006001 | 0.016964 | -3.13990367 |
| ATP2C2 | 0.136513407 | 1.75037602 | 2.753228003 | 0.006002 | 0.016964 | -3.13998927 |
| DNASE1L1 | 0.080431457 | 3.189613922 | 2.753159624 | 0.006003 | 0.016964 | -3.14017442 |
| RASL10A | -0.094868031 | 1.550302876 | -2.75304997 | 0.006005 | 0.016965 | -3.14047133 |
| DUOX1 | -0.121108755 | 2.094234767 | -2.75285981 | 0.006008 | 0.016971 | -3.14098621 |
| NUP85 | 0.070905425 | 3.697190141 | 2.752519981 | 0.006014 | 0.016984 | -3.14190623 |
| STRADA | -0.062339047 | 2.703132336 | -2.75238258 | 0.006017 | 0.016987 | -3.14227818 |
| MBD2 | 0.069377538 | 3.666596308 | 2.752190066 | 0.00602 | 0.016992 | -3.1427993 |
| BEND7 | -0.107921992 | 2.950402792 | -2.75121714 | 0.006038 | 0.017038 | -3.14543237 |
| ANPEP | 0.183721756 | 3.068971668 | 2.750843805 | 0.006045 | 0.017053 | -3.14644252 |
| GSDMD | 0.104332166 | 3.917899983 | 2.750307246 | 0.006055 | 0.017076 | -3.14789405 |
| ZMIZ1 | -0.097335657 | 4.176194921 | -2.74914711 | 0.006076 | 0.017131 | -3.15103158 |
| ASB2 | -0.10574477 | 1.552255673 | -2.74906408 | 0.006078 | 0.017131 | -3.15125606 |
| EDC4 | -0.082089974 | 3.237459527 | -2.74900724 | 0.006079 | 0.017131 | -3.15140974 |
| ZDHHC14 | -0.077333935 | 2.322855839 | -2.74863203 | 0.006086 | 0.017146 | -3.15242415 |
| EIF2B1 | -0.063032519 | 3.973591286 | -2.74816286 | 0.006094 | 0.017166 | -3.15369238 |
| CASZ1 | 0.085509954 | 2.792235003 | 2.746934812 | 0.006117 | 0.017225 | -3.15701097 |
| IKZF3 | -0.09183801 | 1.908638523 | -2.74631802 | 0.006128 | 0.017253 | -3.15867719 |
| NPC2 | 0.099129881 | 5.349421712 | 2.746084362 | 0.006133 | 0.017261 | -3.15930831 |
| KCND3 | -0.115800182 | 1.819032062 | -2.74559841 | 0.006142 | 0.017282 | -3.1606207 |
| LRTOMT | 0.08386104 | 2.634517851 | 2.745371311 | 0.006146 | 0.017289 | -3.16123395 |
| NSMAF | -0.062849999 | 3.106844791 | -2.74479495 | 0.006157 | 0.017312 | -3.16279009 |
| FRS3 | -0.075901031 | 2.561059097 | -2.74476646 | 0.006157 | 0.017312 | -3.16286701 |
| SLC2A10 | -0.099228522 | 3.120201257 | -2.74419093 | 0.006168 | 0.017338 | -3.16442055 |
| GLIS2 | -0.114780042 | 3.82565867 | -2.7437854 | 0.006176 | 0.017355 | -3.16551504 |
| NINJ1 | 0.088911134 | 3.952691345 | 2.742917362 | 0.006192 | 0.017396 | -3.16785722 |
| SMARCC2 | -0.080892932 | 4.069060173 | -2.74208308 | 0.006207 | 0.017435 | -3.17010763 |
| TBX19 | -0.08443024 | 2.538467237 | -2.74185916 | 0.006212 | 0.01744 | -3.17071154 |
| PRR5 | -0.075988499 | 2.743114585 | -2.74182179 | 0.006212 | 0.01744 | -3.1708123 |
| NIPA1 | 0.10046867 | 2.250614984 | 2.740974553 | 0.006228 | 0.017481 | -3.17309677 |
| GPAM | -0.075182701 | 2.514372736 | -2.74081092 | 0.006231 | 0.017485 | -3.17353791 |
| LRRC23 | -0.107478366 | 2.872019115 | -2.74064219 | 0.006235 | 0.017489 | -3.17399275 |
| NUP54 | 0.07519379 | 3.357427233 | 2.738486211 | 0.006275 | 0.017599 | -3.17980222 |
| PPP1R1B | 0.240081003 | 3.232776954 | 2.738172219 | 0.006281 | 0.017611 | -3.18064792 |
| ACO2 | 0.086768946 | 4.034816995 | 2.735852938 | 0.006325 | 0.017731 | -3.18689168 |
| NCAPD2 | 0.104646204 | 4.299043009 | 2.735617197 | 0.00633 | 0.017739 | -3.18752603 |
| CYB5B | 0.077902149 | 3.473166148 | 2.735095672 | 0.00634 | 0.017762 | -3.18892919 |
| APRT | 0.095474533 | 4.464954741 | 2.734411883 | 0.006353 | 0.017792 | -3.19076853 |
| RAB3GAP2 | -0.062066418 | 2.996264784 | -2.73437329 | 0.006354 | 0.017792 | -3.19087234 |
| MIER1 | 0.064368979 | 3.441486664 | 2.733983012 | 0.006361 | 0.017808 | -3.19192193 |
| CLUL1 | -0.145553455 | 2.12198086 | -2.73219566 | 0.006396 | 0.0179 | -3.19672687 |
| RTP4 | 0.16954558 | 3.438445545 | 2.73124914 | 0.006414 | 0.017947 | -3.19927016 |
| ASB16 | -0.100482879 | 1.48554957 | -2.72954796 | 0.006447 | 0.018034 | -3.20383901 |
| OAZ1 | 0.066716048 | 5.510423803 | 2.728636044 | 0.006464 | 0.018079 | -3.20628698 |
| TRIT1 | 0.084961815 | 3.750321369 | 2.727496392 | 0.006487 | 0.018137 | -3.20934517 |
| SALL1 | -0.181154622 | 1.531043737 | -2.72734585 | 0.00649 | 0.01814 | -3.20974905 |
| PRKCQ | -0.112373758 | 2.811256477 | -2.72671996 | 0.006502 | 0.01817 | -3.21142794 |
| MLXIPL | -0.156245489 | 2.806669211 | -2.72619535 | 0.006512 | 0.018194 | -3.2128349 |
| C15orf41 | -0.058809125 | 2.30178592 | -2.72584984 | 0.006519 | 0.018208 | -3.21376136 |
| TNFSF9 | 0.138353845 | 1.517140031 | 2.725091161 | 0.006534 | 0.018245 | -3.21579531 |
| CRNN | 0.125660837 | 1.078218985 | 2.724127804 | 0.006553 | 0.018294 | -3.21837719 |
| RADIL | -0.112206148 | 1.765881127 | -2.72214001 | 0.006592 | 0.018399 | -3.2237018 |
| SRP19 | 0.067503519 | 3.133106112 | 2.721693165 | 0.006601 | 0.018418 | -3.22489821 |
| DNAJB1 | 0.091919217 | 4.850601122 | 2.7216159 | 0.006602 | 0.018418 | -3.22510506 |
| TCP11L2 | -0.070898066 | 2.378490412 | -2.7215316 | 0.006604 | 0.018418 | -3.22533076 |
| SSBP2 | -0.086722045 | 2.934010904 | -2.72112433 | 0.006612 | 0.018436 | -3.22642096 |
| ARRDC4 | -0.110659903 | 2.676429567 | -2.71942053 | 0.006646 | 0.018526 | -3.23098011 |
| GEMIN7 | 0.081679308 | 3.155704403 | 2.718633854 | 0.006662 | 0.018565 | -3.23308422 |
| BMPER | -0.095113278 | 1.236588301 | -2.71825315 | 0.006669 | 0.018582 | -3.23410226 |
| PCOLCE | -0.152650943 | 4.046578721 | -2.71817678 | 0.006671 | 0.018582 | -3.23430647 |
| MED6 | 0.064937218 | 3.158787793 | 2.71805384 | 0.006673 | 0.018584 | -3.23463518 |
| ZNF250 | 0.088812645 | 3.179619925 | 2.717580775 | 0.006683 | 0.018605 | -3.23589992 |
| DALRD3 | -0.082832742 | 3.466146529 | -2.71748834 | 0.006685 | 0.018606 | -3.23614702 |
| FMNL1 | -0.088677364 | 3.001777 | -2.71716524 | 0.006691 | 0.018619 | -3.23701067 |
| TECTA | -0.07689586 | 1.677995297 | -2.7170356 | 0.006694 | 0.018621 | -3.23735717 |
| STARD8 | -0.072335634 | 2.183226167 | -2.71697005 | 0.006695 | 0.018621 | -3.23753236 |
| TOB1 | 0.093487235 | 4.212062782 | 2.716364299 | 0.006707 | 0.01865 | -3.23915116 |
| GRIK5 | -0.164682466 | 2.869327317 | -2.71568608 | 0.006721 | 0.018683 | -3.24096319 |
| CA10 | -0.100154766 | 0.866367807 | -2.71557979 | 0.006723 | 0.018685 | -3.24124714 |
| MPHOSPH8 | -0.069533423 | 3.670992612 | -2.71312418 | 0.006773 | 0.018818 | -3.24780394 |
| DTX3L | 0.101709751 | 4.354792398 | 2.713015982 | 0.006775 | 0.018819 | -3.24809271 |
| KCNG3 | 0.117849413 | 1.357389794 | 2.712733675 | 0.006781 | 0.018826 | -3.2488461 |
| ACOX1 | 0.071776539 | 3.38229915 | 2.71272901 | 0.006781 | 0.018826 | -3.24885855 |
| PRRG1 | 0.092175057 | 2.498333367 | 2.712388404 | 0.006788 | 0.01884 | -3.24976742 |
| FLRT3 | -0.160762092 | 2.138577786 | -2.71230929 | 0.006789 | 0.01884 | -3.24997852 |
| SRPX | -0.134597539 | 2.486689384 | -2.71221496 | 0.006791 | 0.018841 | -3.25023018 |
| SNRNP27 | 0.064581969 | 3.87223942 | 2.711934715 | 0.006797 | 0.018852 | -3.25097786 |
| RAB40C | -0.074866379 | 3.305061467 | -2.70973851 | 0.006842 | 0.018972 | -3.25683455 |
| SERINC1 | -0.088373392 | 4.459453625 | -2.70949112 | 0.006847 | 0.018981 | -3.25749397 |
| PIM3 | 0.092373442 | 3.950366331 | 2.709359159 | 0.00685 | 0.018984 | -3.25784569 |
| IFNAR1 | 0.075018639 | 3.759791976 | 2.706509666 | 0.006908 | 0.019141 | -3.26543647 |
| PDLIM1 | -0.103482691 | 4.807526776 | -2.70640101 | 0.00691 | 0.019143 | -3.26572576 |
| ZNF141 | -0.073146357 | 2.688299119 | -2.70548737 | 0.006929 | 0.01919 | -3.26815785 |
| IPO4 | 0.08575299 | 2.626249421 | 2.704666835 | 0.006946 | 0.019222 | -3.2703414 |
| GIN1 | -0.060391899 | 2.211350805 | -2.70463594 | 0.006947 | 0.019222 | -3.27042359 |
| ZNF208 | -0.137204496 | 2.119216768 | -2.70463041 | 0.006947 | 0.019222 | -3.27043833 |
| AHR | -0.109539333 | 3.983402358 | -2.70460162 | 0.006948 | 0.019222 | -3.27051492 |
| AFAP1 | -0.09769183 | 2.990751154 | -2.70339381 | 0.006973 | 0.019287 | -3.27372779 |
| RYR3 | -0.101244371 | 1.51472485 | -2.70302062 | 0.006981 | 0.019303 | -3.27472023 |
| RPL34 | -0.102346851 | 5.671480965 | -2.7027993 | 0.006985 | 0.019311 | -3.27530872 |
| PEX6 | -0.106599121 | 3.668461992 | -2.7015835 | 0.007011 | 0.019377 | -3.27854073 |
| GPR176 | -0.090809118 | 2.176316001 | -2.70144583 | 0.007014 | 0.01938 | -3.27890661 |
| WT1 | 0.148318083 | 4.830607178 | 2.700635897 | 0.007031 | 0.019422 | -3.28105879 |
| CD300LB | -0.100005811 | 1.485313089 | -2.69957275 | 0.007053 | 0.019479 | -3.28388283 |
| PKN3 | 0.095654759 | 2.721270616 | 2.699208587 | 0.007061 | 0.019495 | -3.28484991 |
| TBCD | 0.077693627 | 3.516303524 | 2.698839357 | 0.007068 | 0.019512 | -3.28583032 |
| KLHL25 | -0.08894495 | 2.489125044 | -2.69854856 | 0.007075 | 0.019524 | -3.28660237 |
| PRKDC | 0.08752112 | 4.205614641 | 2.697345361 | 0.0071 | 0.019589 | -3.28979591 |
| NDUFS1 | 0.060409762 | 3.624004544 | 2.696506473 | 0.007118 | 0.019633 | -3.29202168 |
| TNNT1 | 0.205274326 | 4.158088402 | 2.696328871 | 0.007121 | 0.019639 | -3.29249281 |
| NR2C2AP | 0.097918174 | 3.590924889 | 2.696207013 | 0.007124 | 0.019641 | -3.29281604 |
| KIF2A | 0.081074753 | 3.308671115 | 2.695852453 | 0.007132 | 0.019657 | -3.29375646 |
| KHDRBS1 | -0.04931396 | 4.848859132 | -2.69507043 | 0.007148 | 0.019693 | -3.29583023 |
| NEBL | -0.108946009 | 2.188756429 | -2.69506599 | 0.007148 | 0.019693 | -3.29584201 |
| HAS1 | -0.135908018 | 1.355274533 | -2.69438391 | 0.007163 | 0.019728 | -3.29765026 |
| APBA3 | -0.064873986 | 2.868979603 | -2.6940136 | 0.007171 | 0.019745 | -3.29863181 |
| BNIP2 | -0.06908775 | 3.485554971 | -2.69383305 | 0.007175 | 0.019751 | -3.29911032 |
| RPS4X | -0.089892598 | 6.865949896 | -2.6936607 | 0.007178 | 0.019756 | -3.29956708 |
| GNA12 | -0.072275019 | 3.518877719 | -2.69357376 | 0.00718 | 0.019756 | -3.29979745 |
| METTL5 | 0.069503686 | 3.825938676 | 2.692957637 | 0.007193 | 0.019788 | -3.30142998 |
| TRAPPC2L | 0.081467374 | 3.138263694 | 2.692788253 | 0.007197 | 0.019793 | -3.30187873 |
| DZIP3 | -0.074024 | 2.697140863 | -2.69264328 | 0.0072 | 0.019796 | -3.30226278 |
| CDH13 | 0.112422902 | 1.843481401 | 2.692397668 | 0.007205 | 0.019806 | -3.3029134 |
| BMP2 | -0.109477839 | 1.607472329 | -2.6921962 | 0.00721 | 0.019813 | -3.30344703 |
| LGR6 | -0.207831904 | 3.430791187 | -2.69028602 | 0.007251 | 0.019921 | -3.30850464 |
| ZNF318 | -0.074742102 | 3.143421063 | -2.68897228 | 0.007279 | 0.019994 | -3.311981 |
| EVL | -0.085854599 | 3.534103031 | -2.68804131 | 0.007299 | 0.020044 | -3.31444347 |
| ARV1 | 0.079937551 | 3.668629179 | 2.687828067 | 0.007304 | 0.020052 | -3.31500738 |
| USH1C | -0.126587255 | 1.36744805 | -2.68739583 | 0.007313 | 0.020073 | -3.31615028 |
| DZIP1 | -0.106502567 | 2.78704282 | -2.68646729 | 0.007334 | 0.020124 | -3.31860492 |
| DTYMK | 0.082269159 | 3.680708119 | 2.686126707 | 0.007341 | 0.020139 | -3.31950504 |
| GTF2B | 0.069394487 | 3.511692211 | 2.684915914 | 0.007368 | 0.020206 | -3.32270415 |
| TCTN2 | -0.088689613 | 3.101246346 | -2.68394825 | 0.007389 | 0.02026 | -3.32525984 |
| CTSF | -0.102905011 | 4.240105521 | -2.68326434 | 0.007404 | 0.020296 | -3.32706558 |
| CLCC1 | 0.066155264 | 3.204911988 | 2.682304015 | 0.007425 | 0.020344 | -3.32960036 |
| CAMTA2 | -0.070449365 | 3.349703082 | -2.68229287 | 0.007425 | 0.020344 | -3.32962976 |
| GLIS1 | -0.112971656 | 1.5797074 | -2.68188188 | 0.007434 | 0.020364 | -3.3307143 |
| KCNH8 | -0.137844088 | 1.509379099 | -2.68156493 | 0.007441 | 0.020376 | -3.33155056 |
| HOXD9 | 0.157050037 | 2.594417175 | 2.681522388 | 0.007442 | 0.020376 | -3.33166281 |
| KCNE4 | -0.106168849 | 2.206766747 | -2.68138144 | 0.007445 | 0.020379 | -3.33203467 |
| KCNAB1 | -0.085407013 | 1.573892289 | -2.68062274 | 0.007462 | 0.02042 | -3.33403593 |
| EML2 | 0.087303036 | 2.879423751 | 2.68046118 | 0.007466 | 0.020425 | -3.33446202 |
| PRSS21 | 0.220016505 | 3.537699045 | 2.680261365 | 0.00747 | 0.020432 | -3.33498897 |
| TPST1 | -0.086684567 | 3.16157567 | -2.67950599 | 0.007487 | 0.020473 | -3.33698066 |
| TTC7A | -0.073263987 | 3.243808769 | -2.678375 | 0.007512 | 0.020536 | -3.3399617 |
| CYP2J2 | 0.145199374 | 2.195973773 | 2.677347439 | 0.007535 | 0.020594 | -3.34266903 |
| COMMD7 | 0.064441124 | 4.240774532 | 2.676593893 | 0.007552 | 0.020635 | -3.34465376 |
| TCEAL2 | -0.181647534 | 2.048126621 | -2.67531693 | 0.00758 | 0.020708 | -3.34801584 |
| TMED10 | 0.077124848 | 5.038723532 | 2.675128918 | 0.007585 | 0.020715 | -3.34851072 |
| ING2 | 0.078842032 | 2.937784332 | 2.673402481 | 0.007624 | 0.020816 | -3.35305339 |
| PTPN7 | -0.090273309 | 2.139869101 | -2.67281471 | 0.007637 | 0.020847 | -3.3545993 |
| GPR183 | -0.151765508 | 2.630599159 | -2.67233311 | 0.007648 | 0.020871 | -3.35586569 |
| LILRA5 | 0.09939412 | 1.382728142 | 2.67208638 | 0.007653 | 0.020879 | -3.35651441 |
| COL23A1 | 0.177670183 | 2.950470079 | 2.672047807 | 0.007654 | 0.020879 | -3.35661583 |
| PTPRC | -0.121615753 | 2.806916974 | -2.67091238 | 0.00768 | 0.020944 | -3.35960034 |
| TGFBR2 | -0.099587297 | 3.795063256 | -2.67069964 | 0.007685 | 0.020952 | -3.3601594 |
| STAR | -0.163437621 | 1.26059112 | -2.67060695 | 0.007687 | 0.020952 | -3.36040296 |
| GTF3C2 | -0.060648015 | 4.073190766 | -2.67004958 | 0.0077 | 0.020982 | -3.36186739 |
| PROC | -0.100573054 | 1.360891063 | -2.66936095 | 0.007715 | 0.021019 | -3.36367629 |
| GCC1 | 0.058411448 | 2.984474557 | 2.669261132 | 0.007718 | 0.02102 | -3.36393845 |
| DMKN | -0.143704241 | 4.051067391 | -2.66911713 | 0.007721 | 0.021023 | -3.36431664 |
| TLL1 | -0.083231063 | 1.590367622 | -2.66905801 | 0.007722 | 0.021023 | -3.36447192 |
| UBE2E3 | -0.076059883 | 4.419593923 | -2.66817282 | 0.007742 | 0.021068 | -3.36679621 |
| HPSE2 | -0.121973213 | 1.140195462 | -2.66810566 | 0.007744 | 0.021068 | -3.36697251 |
| CRAT | 0.12393472 | 3.212755682 | 2.6680792 | 0.007745 | 0.021068 | -3.36704197 |
| HSPB8 | -0.146030192 | 2.569062308 | -2.667624 | 0.007755 | 0.021091 | -3.36823688 |
| CHAD | -0.112087669 | 1.366434033 | -2.66745099 | 0.007759 | 0.021093 | -3.36869099 |
| TESC | -0.123582323 | 1.568813223 | -2.66737992 | 0.007761 | 0.021093 | -3.3688775 |
| LIN9 | 0.077035547 | 2.572933844 | 2.667346561 | 0.007761 | 0.021093 | -3.36896506 |
| MBD4 | 0.060909342 | 3.842685041 | 2.666963894 | 0.00777 | 0.021112 | -3.36996931 |
| COLEC12 | -0.14627102 | 2.768551813 | -2.66687293 | 0.007772 | 0.021112 | -3.37020801 |
| OCEL1 | 0.100352877 | 3.530270721 | 2.666138503 | 0.007789 | 0.021151 | -3.37213492 |
| PCP4 | -0.28777201 | 2.659183529 | -2.66609033 | 0.00779 | 0.021151 | -3.37226129 |
| B3GAT3 | 0.0714828 | 3.896544821 | 2.664844664 | 0.007819 | 0.021223 | -3.3755283 |
| SERTAD1 | 0.102147105 | 3.060417469 | 2.664768097 | 0.007821 | 0.021223 | -3.37572906 |
| SLC25A6 | -0.099188405 | 6.003826459 | -2.66392622 | 0.00784 | 0.021268 | -3.37793614 |
| C9orf116 | 0.137051625 | 2.713908577 | 2.663876034 | 0.007841 | 0.021268 | -3.37806768 |
| FAM8A1 | -0.080857514 | 3.616005218 | -2.66379643 | 0.007843 | 0.021268 | -3.37827632 |
| SLC12A7 | 0.089441815 | 4.173635094 | 2.663644673 | 0.007847 | 0.021273 | -3.37867408 |
| MPZ | -0.094256835 | 1.844293763 | -2.66209008 | 0.007883 | 0.021365 | -3.38274738 |
| MCAT | 0.078763004 | 3.185567214 | 2.661824516 | 0.007889 | 0.021377 | -3.38344297 |
| BSN | -0.096709575 | 1.982681892 | -2.66160053 | 0.007894 | 0.021386 | -3.38402959 |
| GNLY | 0.161224479 | 2.201360893 | 2.661374202 | 0.007899 | 0.021395 | -3.38462232 |
| ITGA6 | -0.098884024 | 3.588026242 | -2.65989534 | 0.007934 | 0.021483 | -3.38849399 |
| PLXNA4 | -0.127149298 | 2.156050249 | -2.6596858 | 0.007939 | 0.021491 | -3.3890424 |
| FXYD7 | -0.172055505 | 1.846624471 | -2.65951496 | 0.007943 | 0.021496 | -3.38948948 |
| TTC21A | -0.078208236 | 2.309815158 | -2.65799366 | 0.007979 | 0.021588 | -3.39346945 |
| RHOU | -0.112993862 | 3.74813729 | -2.65781673 | 0.007983 | 0.021593 | -3.39393221 |
| SSU72 | 0.070752929 | 4.379239678 | 2.657746191 | 0.007984 | 0.021593 | -3.39411668 |
| DYRK1B | -0.105004979 | 3.971399447 | -2.65724984 | 0.007996 | 0.021619 | -3.3954146 |
| WDR77 | 0.088026551 | 3.829929116 | 2.656967992 | 0.008003 | 0.021632 | -3.39615153 |
| GNL3 | 0.070675901 | 4.101048984 | 2.656550871 | 0.008013 | 0.021649 | -3.39724198 |
| AP1M1 | -0.073040711 | 3.221625839 | -2.6565054 | 0.008014 | 0.021649 | -3.39736084 |
| WWC1 | 0.097171741 | 3.718842943 | 2.656449008 | 0.008015 | 0.021649 | -3.39750825 |
| ZNF689 | -0.069718651 | 2.738226758 | -2.65611244 | 0.008023 | 0.021665 | -3.39838796 |
| ZKSCAN1 | -0.073719462 | 3.823723132 | -2.65604404 | 0.008025 | 0.021665 | -3.39856673 |
| SAMD9 | 0.127288805 | 3.250278445 | 2.655561964 | 0.008036 | 0.02169 | -3.39982655 |
| TMEM209 | 0.073487284 | 3.337827284 | 2.654432992 | 0.008063 | 0.021757 | -3.40277602 |
| KRT14 | 0.228965506 | 2.340117887 | 2.653540187 | 0.008084 | 0.021804 | -3.40510762 |
| BLNK | -0.101421508 | 2.412382808 | -2.65353746 | 0.008084 | 0.021804 | -3.40511475 |
| CRYZL1 | -0.057012279 | 2.978857145 | -2.6530774 | 0.008095 | 0.021828 | -3.40631592 |
| NRXN2 | -0.12186404 | 2.089146494 | -2.65226829 | 0.008114 | 0.021875 | -3.40842792 |
| LIMK2 | 0.104471428 | 4.146997814 | 2.652163267 | 0.008117 | 0.021875 | -3.408702 |
| MRPL42 | 0.073189095 | 3.247486631 | 2.652097684 | 0.008118 | 0.021875 | -3.40887315 |
| PCDHB14 | -0.119429021 | 2.259907436 | -2.65191467 | 0.008123 | 0.021881 | -3.40935076 |
| CSNK1D | 0.067251168 | 4.118409969 | 2.651258014 | 0.008138 | 0.021918 | -3.41106411 |
| ANG | -0.125395628 | 1.88304574 | -2.65062587 | 0.008154 | 0.021954 | -3.41271312 |
| SARDH | -0.085196249 | 1.965743605 | -2.6497433 | 0.008175 | 0.022005 | -3.41501473 |
| CDC123 | 0.076350394 | 4.318959698 | 2.649304885 | 0.008185 | 0.022028 | -3.41615778 |
| STAP1 | -0.110272687 | 1.226519518 | -2.64816054 | 0.008213 | 0.022097 | -3.41914045 |
| SLC16A2 | -0.107795483 | 2.673650499 | -2.64740077 | 0.008231 | 0.022141 | -3.42112006 |
| INF2 | 0.105787512 | 4.015203379 | 2.646965568 | 0.008242 | 0.022163 | -3.42225373 |
| FBXO4 | 0.079572442 | 2.69110403 | 2.646906197 | 0.008243 | 0.022163 | -3.42240837 |
| PHF19 | 0.087240749 | 3.096213673 | 2.645635713 | 0.008274 | 0.02224 | -3.42571678 |
| POLR2D | 0.06667107 | 3.319070831 | 2.644722407 | 0.008296 | 0.022294 | -3.42809411 |
| DIP2A | -0.064164013 | 3.036363482 | -2.64447609 | 0.008302 | 0.022305 | -3.42873515 |
| AQP3 | 0.15129471 | 2.928722215 | 2.644402404 | 0.008304 | 0.022305 | -3.42892689 |
| XG | 0.13058678 | 1.583396203 | 2.642489102 | 0.008351 | 0.022425 | -3.43390399 |
| HPS5 | -0.06477503 | 2.883142453 | -2.64215292 | 0.008359 | 0.022441 | -3.43477814 |
| SAMD13 | -0.111484542 | 1.688080319 | -2.64204076 | 0.008362 | 0.022443 | -3.43506975 |
| RBM42 | 0.081607235 | 4.524351863 | 2.641766005 | 0.008368 | 0.022456 | -3.43578406 |
| CCR7 | -0.12567986 | 1.707631601 | -2.64049307 | 0.0084 | 0.022534 | -3.4390925 |
| DOK1 | -0.07439564 | 2.972796425 | -2.64020962 | 0.008407 | 0.022548 | -3.43982898 |
| IMMP1L | 0.082907169 | 2.866074093 | 2.639965332 | 0.008413 | 0.022558 | -3.44046366 |
| YWHAE | 0.080577946 | 5.631262325 | 2.639837031 | 0.008416 | 0.022561 | -3.44079698 |
| HSD11B1 | -0.136587425 | 1.766825442 | -2.63950325 | 0.008424 | 0.022578 | -3.44166401 |
| AEBP2 | -0.079707795 | 3.3638244 | -2.63937259 | 0.008427 | 0.022581 | -3.44200341 |
| SLC41A3 | -0.065811876 | 3.536428825 | -2.63900918 | 0.008436 | 0.0226 | -3.44294723 |
| FRK | 0.090490687 | 2.611519655 | 2.638910771 | 0.008439 | 0.022601 | -3.4432028 |
| SLCO1B3 | 0.158444629 | 1.200012175 | 2.638792101 | 0.008442 | 0.022603 | -3.44351097 |
| MKNK2 | 0.080523649 | 4.506704019 | 2.637579758 | 0.008472 | 0.022678 | -3.44665846 |
| SUCNR1 | 0.129463407 | 1.514776185 | 2.63668342 | 0.008494 | 0.022732 | -3.44898462 |
| CALM1 | 0.069468149 | 4.832117306 | 2.63658459 | 0.008496 | 0.022732 | -3.44924106 |
| ARHGEF5 | 0.086053615 | 3.028827599 | 2.636528047 | 0.008498 | 0.022732 | -3.44938777 |
| ARID1B | -0.066056635 | 3.622760594 | -2.63568538 | 0.008519 | 0.022782 | -3.45157381 |
| NUDT14 | 0.097558501 | 3.6260093 | 2.635070016 | 0.008534 | 0.022818 | -3.45316973 |
| NOC3L | -0.069785825 | 3.244300935 | -2.63470301 | 0.008543 | 0.022837 | -3.45412137 |
| DST | -0.078592797 | 2.901807119 | -2.63431368 | 0.008553 | 0.022857 | -3.45513077 |
| EMILIN2 | -0.104140307 | 2.978995985 | -2.63389879 | 0.008564 | 0.02288 | -3.45620627 |
| SLC38A5 | 0.138278392 | 2.360641513 | 2.633013096 | 0.008586 | 0.022933 | -3.45850164 |
| EPHX1 | 0.110349885 | 4.840902566 | 2.632058704 | 0.00861 | 0.022992 | -3.4609742 |
| RPLP2 | -0.092872873 | 6.226725225 | -2.63151792 | 0.008623 | 0.023021 | -3.46237484 |
| CTF1 | -0.123545643 | 2.977627446 | -2.63145835 | 0.008625 | 0.023021 | -3.46252909 |
| RAPSN | -0.110964496 | 1.399962226 | -2.63102771 | 0.008636 | 0.023039 | -3.46364422 |
| CRABP1 | 0.320539056 | 3.509144057 | 2.631022166 | 0.008636 | 0.023039 | -3.46365858 |
| ACTB | 0.068306221 | 7.587252061 | 2.630729088 | 0.008643 | 0.023054 | -3.46441739 |
| YTHDF3 | 0.068513338 | 3.926678997 | 2.63063727 | 0.008646 | 0.023054 | -3.4646551 |
| SLC4A5 | -0.064826579 | 1.747745412 | -2.63032252 | 0.008654 | 0.02307 | -3.46546989 |
| CPNE8 | -0.083247677 | 2.036838765 | -2.62987621 | 0.008665 | 0.023094 | -3.46662511 |
| PRRT3 | -0.110884821 | 2.797418892 | -2.62973415 | 0.008668 | 0.023098 | -3.46699276 |
| FUCA1 | -0.076737363 | 3.804720688 | -2.62955546 | 0.008673 | 0.023105 | -3.46745519 |
| HPDL | 0.145916726 | 2.385736892 | 2.629378177 | 0.008677 | 0.023111 | -3.46791396 |
| WDR66 | -0.092277736 | 2.015889953 | -2.62911774 | 0.008684 | 0.023123 | -3.46858784 |
| AHSA1 | 0.077246705 | 4.576181252 | 2.627371352 | 0.008728 | 0.023236 | -3.47310497 |
| OLFML2A | -0.124536953 | 2.959278274 | -2.62688209 | 0.008741 | 0.023264 | -3.47436993 |
| POLI | -0.065609101 | 2.602227943 | -2.62677133 | 0.008744 | 0.023266 | -3.47465626 |
| ADIPOR1 | 0.062802616 | 4.771450103 | 2.626456104 | 0.008752 | 0.023281 | -3.47547112 |
| SOX9 | -0.14275777 | 3.9467343 | -2.62602867 | 0.008763 | 0.023305 | -3.47657587 |
| HM13 | 0.069153413 | 4.3719278 | 2.625834228 | 0.008768 | 0.023312 | -3.47707836 |
| DPT | -0.16101678 | 1.733932726 | -2.62506669 | 0.008787 | 0.023359 | -3.47906156 |
| WNT2 | 0.154505987 | 1.856889134 | 2.623285622 | 0.008833 | 0.023475 | -3.48366134 |
| SFRP2 | -0.310991828 | 3.673016324 | -2.62312759 | 0.008837 | 0.02348 | -3.48406931 |
| FSD1L | 0.067460772 | 2.141983656 | 2.622897339 | 0.008843 | 0.02349 | -3.48466371 |
| XRN1 | 0.059937993 | 3.148092485 | 2.622371799 | 0.008857 | 0.023521 | -3.48602018 |
| TSNARE1 | 0.089681825 | 3.217605749 | 2.621823782 | 0.008871 | 0.023553 | -3.48743438 |
| CLIC2 | -0.097600007 | 2.2347416 | -2.62130606 | 0.008884 | 0.023583 | -3.48877014 |
| SKI | -0.088912365 | 4.242194194 | -2.62091786 | 0.008894 | 0.023603 | -3.48977155 |
| MIER2 | -0.06112789 | 3.013629364 | -2.62084821 | 0.008896 | 0.023603 | -3.4899512 |
| PHC3 | 0.067854891 | 3.211352783 | 2.620302603 | 0.00891 | 0.02363 | -3.49135837 |
| MCCC1 | 0.077569246 | 3.734518919 | 2.620285726 | 0.008911 | 0.02363 | -3.4914019 |
| FKBP4 | 0.087452277 | 4.645714006 | 2.620020602 | 0.008918 | 0.023643 | -3.49208557 |
| ZHX2 | -0.11234723 | 3.720276565 | -2.61906476 | 0.008942 | 0.023703 | -3.49454982 |
| ZNF57 | 0.07474486 | 2.275565829 | 2.618836235 | 0.008948 | 0.023713 | -3.49513885 |
| NID1 | -0.112069688 | 3.175368924 | -2.61854046 | 0.008956 | 0.023728 | -3.49590113 |
| ATF6B | -0.07279144 | 4.546096715 | -2.61820395 | 0.008965 | 0.023745 | -3.49676831 |
| CCL28 | 0.14953009 | 3.238696616 | 2.618117552 | 0.008967 | 0.023746 | -3.49699094 |
| TRIM62 | 0.078607859 | 2.833249347 | 2.617696835 | 0.008978 | 0.023769 | -3.49807492 |
| RUFY2 | -0.053350748 | 2.602041646 | -2.61756712 | 0.008982 | 0.023772 | -3.49840909 |
| SH3GLB1 | 0.064304513 | 3.927266958 | 2.616866579 | 0.009 | 0.023815 | -3.50021358 |
| TRIAP1 | 0.076690974 | 3.673526165 | 2.616511563 | 0.009009 | 0.023834 | -3.50112786 |
| SLAIN1 | 0.124263647 | 3.172837729 | 2.615403505 | 0.009038 | 0.023905 | -3.50398068 |
| SERPINB8 | 0.082498391 | 2.569566722 | 2.615251321 | 0.009042 | 0.02391 | -3.5043724 |
| EWSR1 | 0.065150571 | 4.281655774 | 2.61485806 | 0.009053 | 0.02393 | -3.50538456 |
| PTP4A1 | 0.082538133 | 4.009945554 | 2.614786163 | 0.009055 | 0.02393 | -3.50556958 |
| TMEM156 | -0.098668567 | 1.343705129 | -2.61472521 | 0.009056 | 0.02393 | -3.50572643 |
| ADAMTSL1 | -0.081871376 | 2.034698853 | -2.61404726 | 0.009074 | 0.023971 | -3.50747085 |
| CHCHD5 | 0.083907648 | 3.149186618 | 2.613554739 | 0.009087 | 0.024 | -3.50873789 |
| DNMT1 | 0.096667481 | 3.890395399 | 2.613042673 | 0.009101 | 0.02403 | -3.51005494 |
| NT5DC2 | 0.099404117 | 4.113245595 | 2.611886264 | 0.009131 | 0.024105 | -3.51302832 |
| SLC35D1 | 0.078193047 | 2.774577275 | 2.611722447 | 0.009136 | 0.024111 | -3.51344942 |
| PPM1K | -0.071445332 | 2.438733315 | -2.61159629 | 0.009139 | 0.024114 | -3.51377371 |
| ADCK2 | 0.079575736 | 3.626948428 | 2.611377183 | 0.009145 | 0.024123 | -3.51433687 |
| EIF2S3 | 0.079855877 | 4.924472126 | 2.610842713 | 0.009159 | 0.024155 | -3.5157104 |
| CNTNAP2 | -0.122219636 | 1.217971761 | -2.61069422 | 0.009163 | 0.02416 | -3.51609196 |
| ORAI2 | -0.076278138 | 3.0438863 | -2.61008735 | 0.009179 | 0.024197 | -3.51765114 |
| FAM20A | 0.157837453 | 3.481270476 | 2.6099129 | 0.009184 | 0.024203 | -3.51809927 |
| SYT13 | -0.14644003 | 2.376536189 | -2.60953154 | 0.009194 | 0.024224 | -3.51907882 |
[truncated: 235,378 more chars]
