# Supplementary material for: Characterization of hypoxia-responsive states in ovarian cancer to identify hot tumors and aid adjuvant therapy
Source: Discov Oncol. 2024 Jan 31;15:23. doi: 10.1007/s12672-024-00859-8 (PMC10831007; doi:10.1007/s12672-024-00859-8)

Figure S1 The flowchart of the present research.


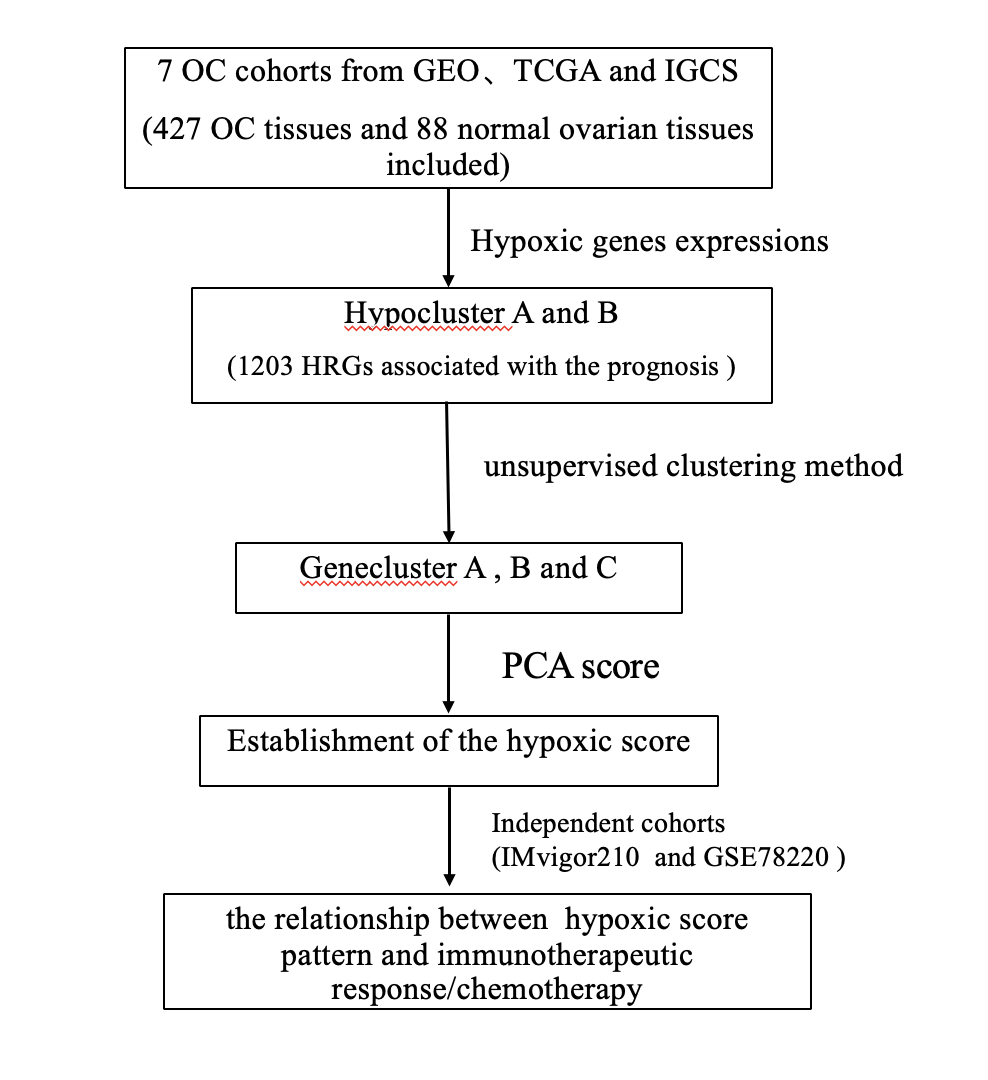


Figure S2 K-M survival curves of these fifteen hypoxia-genes


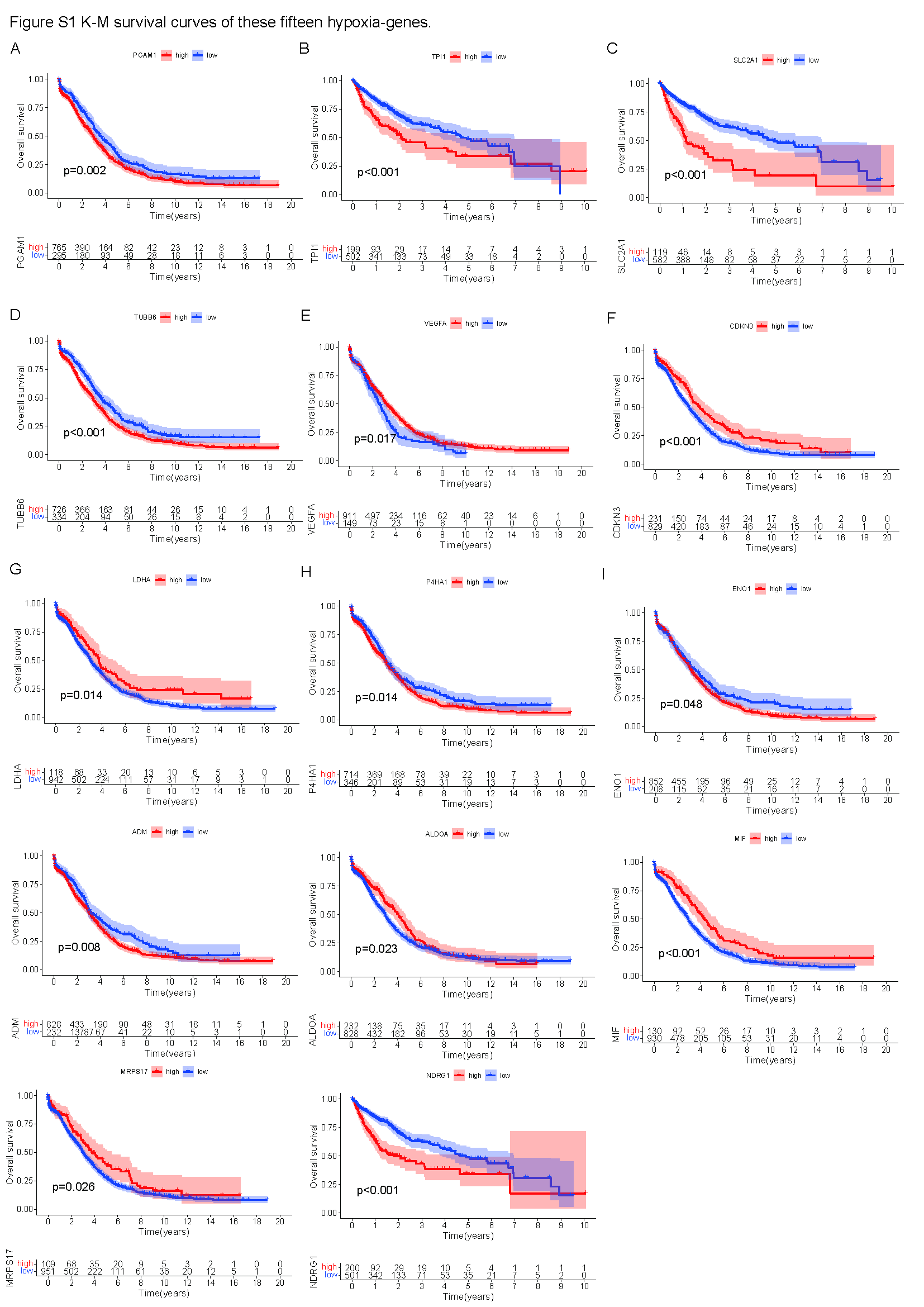

Supplement: Supplementary file 2 — Additional file 2: Figure S1. K-M survival curves of these fifteen hypoxia-genes. [file 12672_2024_859_MOESM2_ESM.doc]
